# High Site Selectivity in Electrophilic Aromatic Substitutions: Mechanism of C–H Thianthrenation

**DOI:** 10.1021/jacs.1c06281

**Published:** 2021-09-21

**Authors:** Fabio Juliá, Qianzhen Shao, Meng Duan, Matthew B. Plutschack, Florian Berger, Javier Mateos, Chenxi Lu, Xiao-Song Xue, K. N. Houk, Tobias Ritter

**Affiliations:** † Max-Planck-Institut für Kohlenforschung, Kaiser-Wilhelm Platz 1, D-45470 Mülheim an der Ruhr, Germany; ‡ Department of Chemistry and Biochemistry, University of California, Los Angeles, California 90095-1569 United States

## Abstract

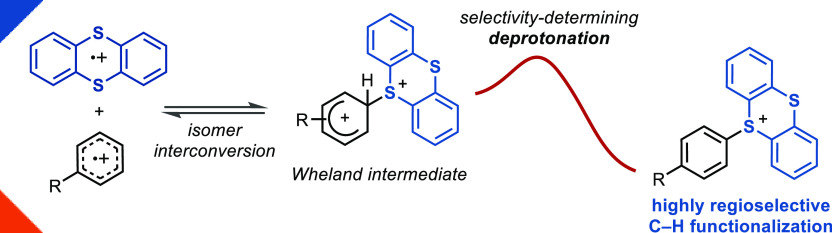

The introduction of thianthrene as a linchpin has proven to be a versatile strategy for the C–H functionalization of aromatic compounds, featuring a broad scope and fast diversification. The synthesis of aryl thianthrenium salts has displayed an unusually high *para* regioselectivity, notably superior to those observed in halogenation or borylation reactions for various substrates. We report an experimental and computational study on the mechanism of aromatic C–H thianthrenation reactions, with an emphasis on the elucidation of the reactive species and the nature of the exquisite site selectivity. Mechanisms involving a direct attack of arene to the isolated *O*-trifluoracetylthianthrene *S*-oxide (**TT^+^-TFA**) or to the thianthrene dication (**TT^2+^**) via electron transfer under acidic conditions are identified. A reversible interconversion of the different Wheland-type intermediates before a subsequent, irreversible deprotonation is proposed to be responsible for the exceptional *para* selectivity of the reaction.

## Introduction

Selective functionalization of aromatic C–H bonds is a longstanding challenge for synthetic chemists, despite the 150-year history^1^ of electrophilic aromatic substitution (S_E_Ar) chemistry.^2^ The past decades have witnessed a large growth of transition-metal-catalyzed C–H functionalization chemistry, in part also to control positional selectivity in arene functionalization.^3,4^ While some aspects of positional selectivity, such as *ortho*/*para* over *meta* selectivity in conventional S_E_Ar reactions^2,5,6^ or chelation-assisted *ortho* selectivity induced by coordinating directing groups in metal-catalyzed aromatic C–H functionalization,^7,8^ are well understood, highly selective reactions that are not dependent on specific directing groups or substitution patterns are scarce, and the source of selectivity is generally not well understood.^9,10^ We have investigated the origins of the unusually high positional selectivity observed in the thianthrenation of arenes and report here the discovery of guiding principles that will be of value for the design of similarly selective functionalizations.

**Scheme 1:**
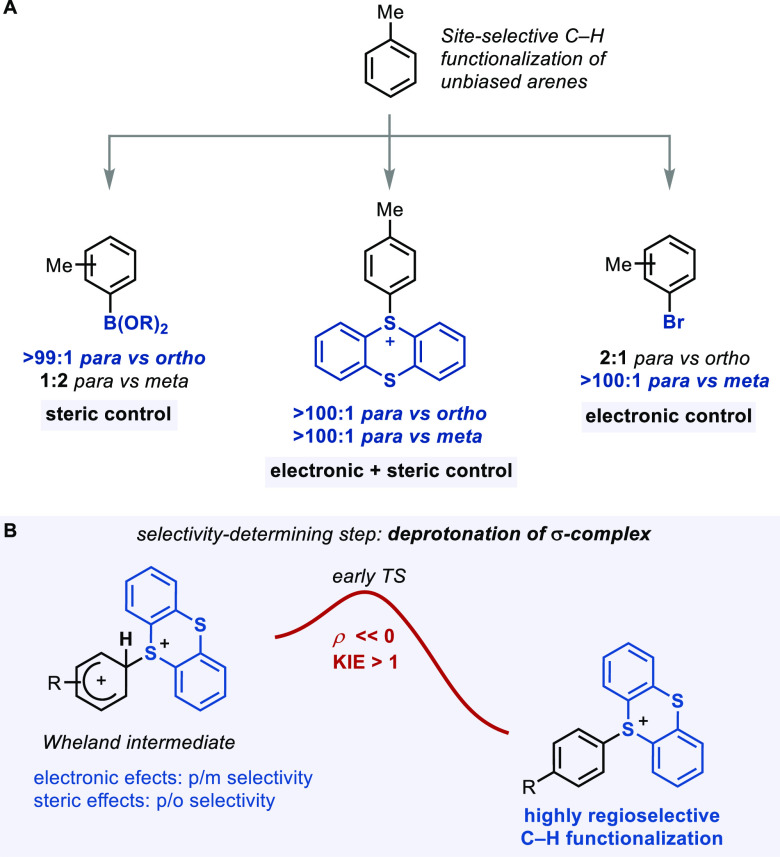
(A) Methods for Selective C–H Functionalization of Arenes to Introduce Linchpins and (B) Selectivity-Determining Deprotonation in Aromatic C–H Thianthrenations

The development of reactions that install a reactive linchpin in place of a C–H bond is highly sought after because it allows multiple diversification pathways.^11−14^ In particular, S_E_Ar reactions are among the most extensively studied and used reactions for arene C–H functionalization.^2,15,16^ We teach the regioselectivity of S_E_Ar reactions already at an early career stage to undergraduate students, yet many S_E_Ar reactions are rather unselective, especially when it comes to *para* vs *ortho* selectivity, and much effort has been placed to reliably predict the position of electrophilic attack.^17−20^ A powerful alternative to S_E_Ar chemistry is undirected C–H borylation, for which high regioselectivity can be achieved for substrates with bulky substituents or specific substitution patterns, such as 1,3-disubstituted benzenes.^21,22^ With the aim of achieving high levels of site selectivity, chemists have also relied on the use of coordinating directing groups to target the activation of *o*-, *m-*, and, to a lesser extent, *p*-C–H bonds with the aid of transition-metal catalysts.^8,23−26^ However, all the aforementioned strategies cannot provide a highly regioselective functionalization of arenes that lack the required, appropriately positioned substituents (Scheme 1A). The site-selective introduction of a versatile reactivity handle in a broad range of arenes remains a challenging task.

As part of a longstanding interest in regioselective aromatic C–H functionalizations,^9,27−31^ our group recently reported the use of thianthrene (**TT**) as a new type of functional linchpin.^32^ A remarkably high site selectivity for C–H functionalization was obtained with a strong preference for the formation of the *para* isomer in greater than 200:1 selectivity for ethylbenzene. Not only do thianthrenation reactions have a broad scope but also the resulting aryl sulfonium salts (**Ar–TT**^**+**^) are versatile intermediates that enable diversification via transition-metal cross-coupling reactions and photoredox catalysis for C–C,^32−35^ C–N,^36^ C–O,^37^ C–S,^38^ C–F,^39^ C–B,^32,40^ and C–Ge^41^ bond formation. In parallel, the groups of Procter and Alcarazo have reported the use of dibenzothiophene *S*-oxide (**DBTO**) to access the corresponding sulfonium salts (**Ar–DBT**^**+**^).^42,43^ With the aim of introducing ^18^F via nucleophlic substitution,^44^ we have also used **Ar–DBT**^**+**^ salts as linchpins, finding site selectivities somewhat lower than those observed for **Ar–TT**^**+**^ in their preparation from arenes (>50:1 in ethylbenzene). While the synthetic utility of aryl sulfonium salts is currently well recognized,^45−51^ the reasons behind the high selectivity on their formation from arenes still remain largely unexplored and are not well understood.

In this report we describe an experimental and theoretical investigation of the C–H thianthrenation of arenes and attempt to extract generalizable aspects to aid in the design of new, highly selective aromatic C–H functionalization chemistry. Our data are consistent with irreversible deprotonation of energetically accessible Wheland intermediates being the selectivity-determining step (Scheme 1B), while a reversible carbon–electrophile (C–S) bond-forming event can sample the energetically most favorable constitutional isomer (*ortho* vs *meta* vs *para*). Early transition states (TSs) of the ensuing deprotonation retain the order of relative energies, in agreement with the Evans–Polanyi principle, and thereby result in high *para* selectivity. Formation of the Wheland intermediates can contribute to the regioselectivity and disfavor isomers that display a larger barrier for formation of the Wheland intermediate than for the deprotonation of others. The regioselectivity is thus dictated by the distinct stability of the σ-complexes, which is governed by electronic (*para* vs *meta*) and steric (*para* vs *ortho*) effects. The present study provides a new analysis of how to attain high levels of regiocontrol in S_E_Ar.

## Results and Discussion

### Reactive Species

**Scheme 2:**
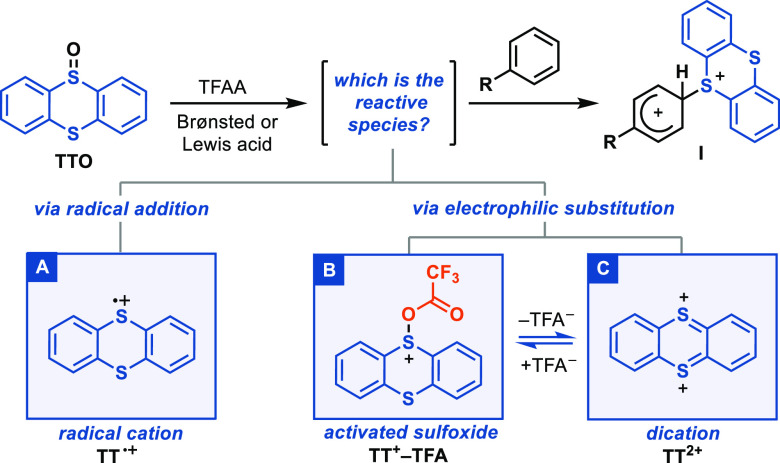
Elucidation of the Reactive Species in Thianthrenation of Arenes: Possible Reactive Species

Although they have been speculated upon, the mechanism and reactive species for thianthrenation have not been investigated in detail. In our protocol, the **TT** substituent and its tetrafluoro analogue **TFT** (2,3,7,8-tetrafluorothianthrene) are introduced by activation of the respective thianthrene *S*-oxide (**TTO** and **TFTO**) with trifluoroacetic anhydride (TFAA) and a Brønsted or Lewis acid (Scheme 2).^32^ A Hammett analysis showed that the rates of thianthrenation reactions are significantly accelerated by substituents that stabilize a positive charge in the transition state (ρ = −11), which is in line with the intermediacy of Wheland intermediates (**I**).^32^ We thus considered different potential electrophilic species derived from **TTO** to attack a given arene en route to the σ-complex **I**. At the outset, we considered the thianthrene radical cation (**TT**^**•+**^, box A), the trifluoroacetylated derivative **TT**^**+**^**–TFA** (box B), and the thianthrene dication (**TT**^**2+**^, box C).

#### Thianthrene Radical Cation (**TT**^**•+**^)

**Scheme 3:**
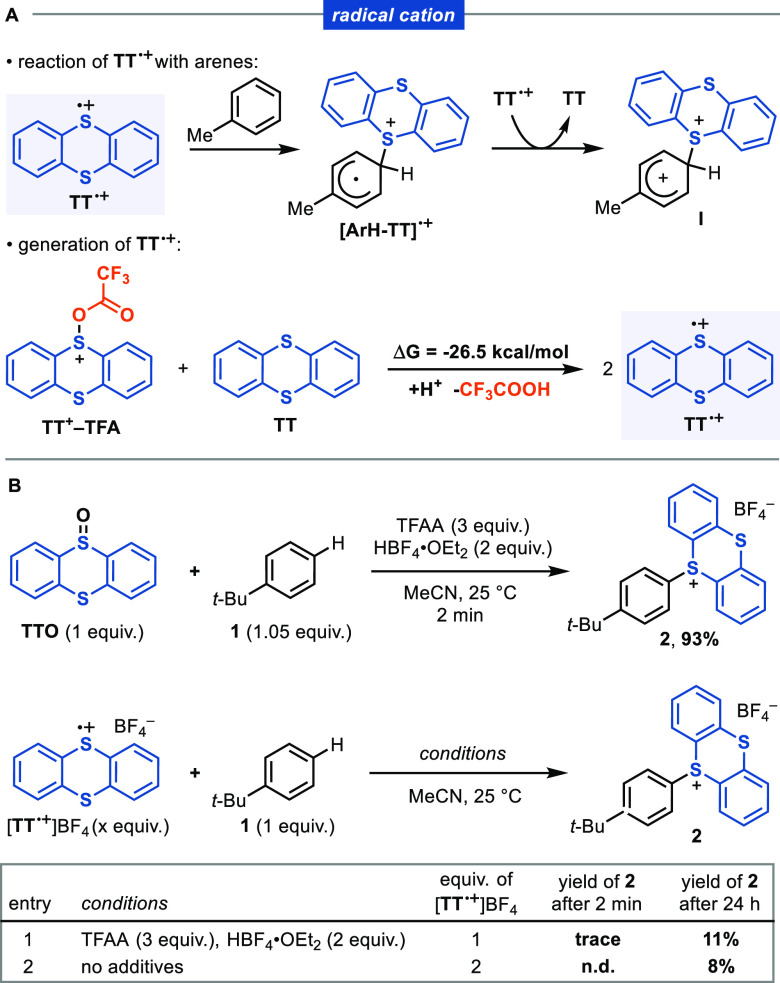
Assessment of Thianthrene Radical Cation (**TT**^**•+**^) as a Reactive Species

In our initial report, we suggested that radical cations, **TT**^**•+**^ or its tetrafluoro analogue **TFT**^**•+**^, might be responsible for the C–H functionalization of arenes.^32^ The Wheland intermediate **I** may be formed by radical addition and subsequent oxidation of the resultant adduct (Scheme 3A). This mechanistic hypothesis is in agreement with the seminal works of Shine^52,53^ and Parker^54^ on the mechanisms of reactions with [**TT**^**•+**^]ClO_4_. In Parker’s proposed mechanism for the thianthrenation of anisole, the arene and **TT**^**•+**^ form the complex **[ArH-TT]**^**•+**^, which has a lower oxidation potential than **TT**^**•+**^. Another 1 equiv of thianthrene radical cation should therefore be able to oxidize **[ArH-TT]**^**•+**^ to generate intermediate **I**. Indeed, Shine and co-workers reported the formation of aryl sulfonium salts by reaction of the salt [**TT**^**•+**^]ClO_4_ with highly electron rich arenes (e.g., anisole).^55^ Conversely, a low yield was obtained when less nucleophilic substrates were tested (e.g., alkylbenzenes), even when they were used in large excess.^56^ In contrast, our protocol based on **TTO** features faster reaction rates and significantly broader scope in comparison with Shine’s earlier observations, raising pertinent questions about the identity of the species accounting for the overall reactivity.

We previously proposed a comproportionation reaction between **TT** or **TFT** and their corresponding trifluoroacylated sulfoxides **TT**^**+**^**–TFA**/**TFT**^**+**^**–TFA** to form 2 equiv of **TT**^**•+**^/**TFT**^**•+**^ (Scheme 3A, bottom). Like its thianthrene analogue, **TFT**^**•+**^ is persistent, could be characterized by electron paramagnetic resonance (EPR) spectroscopy, and is formed in the reaction mixture.^32^ We have now calculated the **TT**/ **TT**^**+**^**–TFA** comproportionation to be thermodynamically favorable (Δ*G* = −26.5 kcal/mol) by density functional theory (DFT). The DFT calculations were carried out at the ωB97X-D/6-311++G(d,p)/SMD//ωB97X-D/6-31+G(d)/SMD level of theory. Details are given in the Supporting Information. When *tert*-butylbenzene (**1**) was treated with **TTO** and TFAA and HBF_4_, we found a fast and efficient reaction to afford aryl thianthrenium salt **2** in 93% yield in less than 2 min (Scheme 3B, top). In contrast, at the same temperature and in both the presence and absence of TFAA and HBF_4_, [**TT**^**•+**^]BF_4_ resulted in only about 10% of the product (Scheme 3B, bottom) after 24 h. In addition, computational studies indicated that the proposed intermediate **[ArH-TT]**^**•+**^is not stable, and the C–S bond spontaneously dissociates to arene and [**TT**^**•+**^] (see the Supporting Information). These results point against **TT**^**•+**^ being the main reactive species responsible for product formation.

#### Activated Thianthrene *S*-Oxides (**TT**^**+**^**–OH**, **TT**^**+**^**–TFA**)

**Scheme 4:**
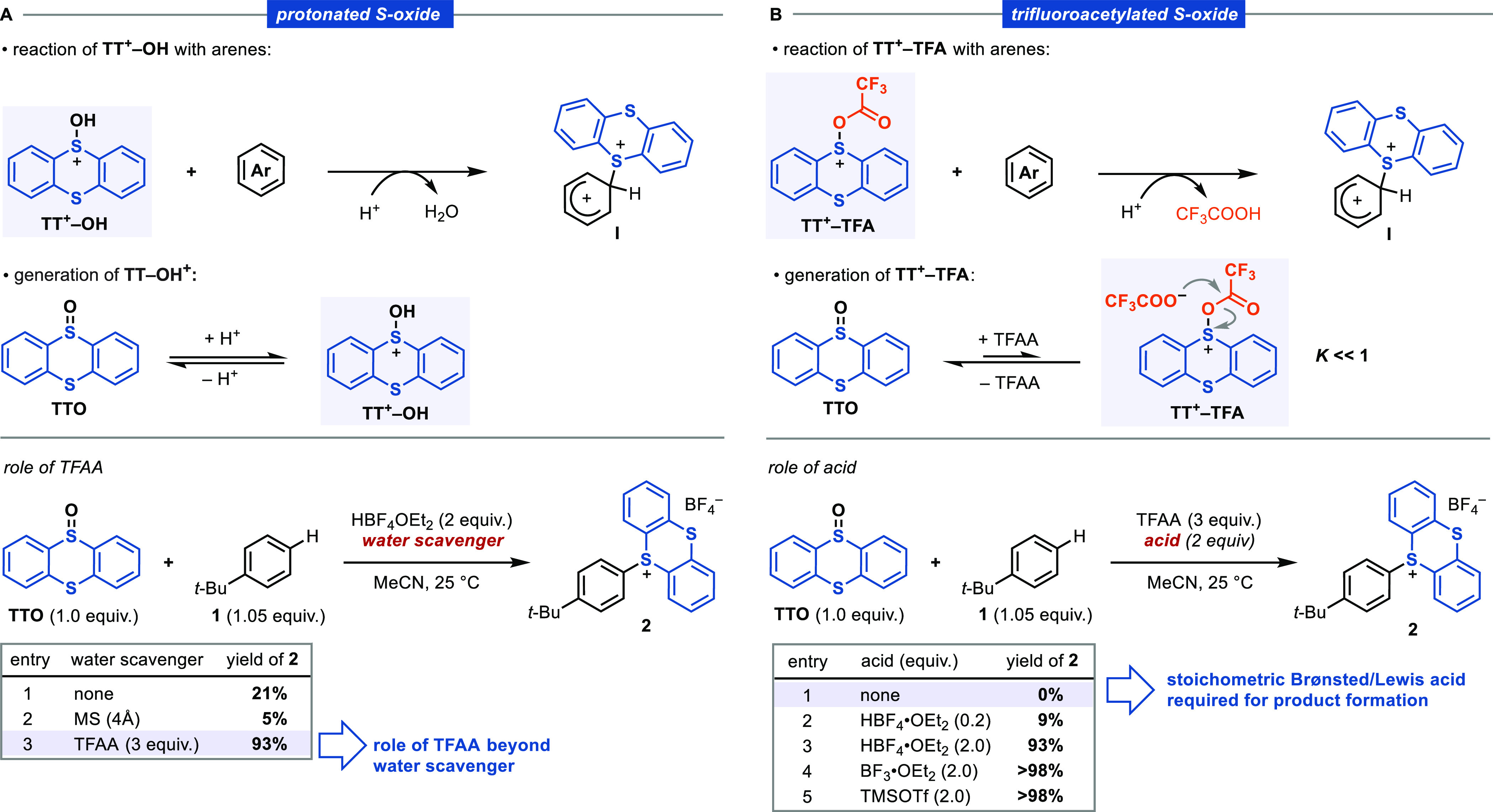
Assessment of Protonated (**TT**^**+**^**–OH**) and Trifluoroacetylated (**TT**^**+**^**–TFA**) Thianthrenium *S*-Oxide as Reactive Species

Sulfoxides are known to react with strong acids and acetylating reagents.^42,57,58^ Because our reaction conditions involve the use of HBF_4_·OEt_2_ and TFAA, we evaluated whether protonated (**TT**^**+**^**–OH**) or trifluoroacetylated (**TT**^**+**^**–TFA**) derivatives of **TTO** can react directly with arenes to afford **I** (Scheme 4). Protonated sulfoxides (R_2_S^**+**^–OH) have been previously reported to react with phenols and other electron-rich aromatics to afford sulfonium salts.^57,59,60^ The observed reactivity is dependent on the strength of the acid, which is required to shift the equilibrium toward the protonated species, and is typically used in combination with a dehydrating agent. The reaction between **TTO** and **1** under our optimized conditions but excluding TFAA resulted in only a 21% yield of **2** after 2 h (Scheme 4A). The marked difference in rate and yield without TFAA (see the Supporting Information for more details) could be related to the ability of TFAA to scavange H_2_O to shift the equilibrium toward **TT**^**+**^**–OH** and, therefore, accelerate the reaction. To probe this hypothesis, we conducted the same experiment but replaced TFAA with 4 Å molecular sieves, which resulted in attenuated reactivity (Scheme 4, entry 2). While other explanations are conceivable, overall, these results reveal that **TT**^**+**^**–OH** is only able to react slowly with unactivated arenes and thus cannot be responsible for the fast product formation observed under the standard reaction conditions (TFAA + HBF_4_·OEt_2_). More importantly, the results suggest that TFAA plays a key role in the reaction that goes beyond its ability to remove water, which is consistent with the relevance of **TT**^**+**^**–TFA**.

**Scheme 5:**
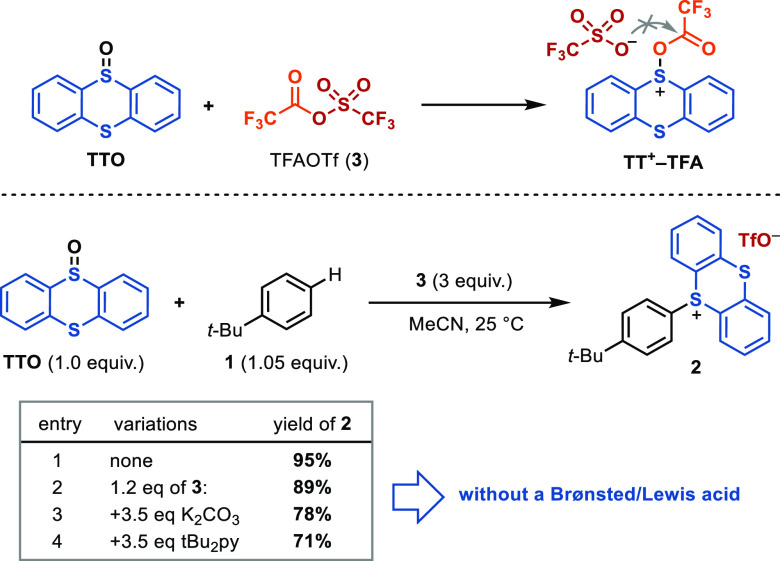
C–H Thianthrenations with Trifluoroacetyl Triflate **3**

The activation of sulfoxides by acylation has been known for a century and has been extensively applied in Pummerer rearrangements.^61−63^ A similar strategy was later applied to the C–H functionalization of aromatic sulfoxides, phenols, and heteroarenes in interrupted-Pummerer reactions.^64−66^ However, although trifluoroacetylated sulfoxides are frequently operating in Pummerer-type rearrangements, their reactivity toward arenes has rarely been described^67^ and is generally restricted to highly electron rich substrates.^68−73^ Recently, Procter and Alcarazo independently reported the formation of aryl sulfonium salts from a broad set of arenes by using dibenzothiophene *S*-oxide (**DBTO**) in combination with triflic anhydride (Tf_2_O).^42,43^ It was proposed that electrophilic intermediates of the type R_2_S^**+**^–OTf are responsible for the extended reactivity with aromatic substrates. Due to the lower basicity of **TTO** in comparison with other sulfoxides,^74^ its reaction with TFAA to form **TT**^**+**^**–TFA** is expected to be less favorable. Indeed, in contrast to observations with dimethyl sulfoxide^75^ or **DBTO**,^76^ NMR experiments did not show any detectable new species when **TTO** and TFAA were mixed at either −50 or 25 °C, consistent with an equilibrium constant much smaller than unity for the formation of **TT**^**+**^**–TFA** (see the Supporting Information). This observation is in agreement with the lack of conversion to **2** when **1** is treated with **TTO** and TFAA in the absence of acid (Scheme 4B). Furthermore, when the acid is used in substoichiometric amounts, lower yields are obtained. On the basis of a putative small equilibrium constant and the necessity for acid, we speculated that the main role of HBF_4_·OEt_2_ in our protocol is to stoichometrically protonate the trifluoroacetate (TFA^–^) released after reaction of **TTO** with TFAA to shift the equilibrium toward the formation of **TT**^**+**^**–TFA**. In fact, other reagents capable of trapping TFA^–^, such as BF_3_·OEt_2_ and TMSOTf, lead to quantitative formation of product **2**. We therefore employed the mixed anhydride of trifluoroacetic and triflic acid (TFAOTf, **3**) for acylation of **TTO**.^77,78^ Because the reaction between **TTO** and **3** would form **TT**^**+**^**–TFA** with a triflate counterion, which is less nucleophilic than trifluoroacetate, the acylation of **TTO** should proceed with a higher equilibrium constant with **3** (Scheme 5). Such a reaction should be able to access **TT**^**+**^**–TFA** in sufficient equilibrium quantities for C–H thianthrenation in the absence of acids, which we verified experimentally. With **3**, the reaction can even be executed under basic conditions. The lack of a strong Brønsted acid in the reaction mixtures at any time rules out a crucial contribution of **TT**^**+**^**–OH** in the thianthrenation of arenes. Conversely, these results are consistent with a reaction mechanism that proceeds through the formation of **TT**^**+**^**–TFA** as a key active intermediate.

#### Thianthrene Dication (**TT**^**2+**^)

Our group recently expanded the C–H thianthrenation strategy to olefins.^79^ Interestingly, we observed the formation of intermediates of [4 + 2] cycloadducts between the olefin and the thianthrene core with the apparent stereospecificity of a cycloaddition with respect to the double-bond geometry. This experimental outcome was not consistent with the operation of an open-shell mechanism that proceds via **TT**^**•+**^ and instead pointed toward the involvement of a different reactive intermediate, i.e. **TT**^**2+**^, for this transformation. Due to the similarity of the conditions employed for the C–H thianthrenation of alkenes and arenes, we decided to evaluate whether the involvement of **TT**^**2+**^ species could be responsible for the observed reactivity in arene functionalization.

A thianthrene dication was originally proposed by Shine as the intermediate undergoing electrophilic attack to the arene in the reaction between **TT**^**•+**^ and anisole.^55^ Later, Parker and co-workers performed mechanistic studies that ruled out this possibility.^54^**TT**^**2+**^ has been experimentally observed when **TTO** is dissolved in neat sulfuric acid to give deep red solutions^80^ or when electrochemical oxidation is carried out (*E* > +1.8 V vs SCE)^81^ in nucleophile-free solvents such as liquid SO_2_.^82,83^ We conducted open-circuit-potential measurements under our reaction conditions and observed the transient formation of highly oxidizing species (*E* = +1.54 V vs SCE), which are consistent with the intermediacy of **TT**^**2+**^ (see the Supporting Information).

**Scheme 6:**
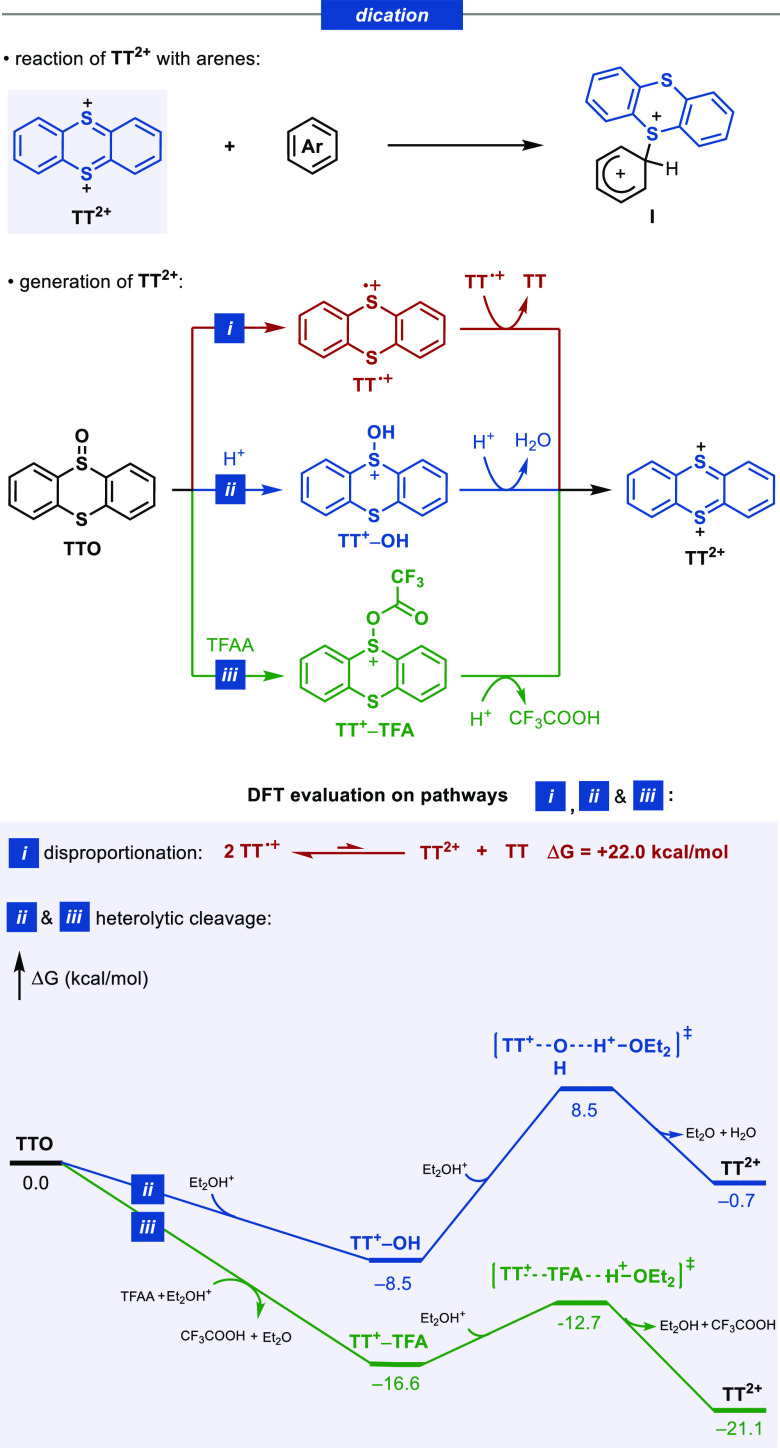
Assesment of a Thianthrene Dication (**TT**^**2+**^) as a Reactive Species

The drastic conditions for the generation of **TT**^**2+**^ are a consequence of its high reactivity toward nucleophiles, which could rationalize a fast reactivity with a broad range of arenes. Under our reaction conditions there are at least three possible pathways that could lead to the formation of **TT**^**2+**^, which are depicted in Scheme 6. It was proposed that **TT**^**2+**^ could be formed by the disproportionation of **TT**^**•+**^ (Scheme 6, pathway *i*).^52^ Parker et al. evaluated this possibility in the reaction of **TT**^**•+**^ with water and determined that the equilibrium constant for disproportionation was small (*K*_dis_ = 2.3 × 10^–9^),^84^ and therefore, disproportionation to **TT**^**2+**^ is not kinetically competent for product formation.

We have computed with DFT the Gibbs free energy for the disproportionation of **TT**^**•+**^ and found it uphill by +22.0 kcal/mol, in line with the results obtained by Parker. These experimental and computational data rule out an effective generation of **TT**^**2+**^ via pathway *i*. Because our conditions involve HBF_4_·OEt_2_ and TFAA, we also considered a dissociative, heterolytic route from **TT**^**+**^**–OH** or **TT**^**+**^**–TFA** (pathways *ii* and *iii*, respectively). Protonation of **TTO** with HBF_4_·OEt_2_ to give **TT**^**+**^**–OH** is favorable, using the protonated solvent as the acid. Subsequent dissociation facilitated by protonation in the transition state has a barrier of 17.0 kcal/mol (Scheme 6, pathway *ii*). The formation of **TT**^**+**^**–TFA** in acid is more favorable, and the corresponding acid-promoted dissociation transition state for generation of **TT**^**2+**^ through the intermediacy of **TT**^**+**^**–TFA** has a lower barrier (Scheme 6, pathway *iii*, same TS as **TS-II** in Figure 2 shown later), which is a very favorable pathway for the formation of **TT**^**2+**^ under our reaction conditions.

#### From **TT**^**+**^**–TFA** to **I**: NMR Studies and Computational Evaluation of the Reaction of **TT**^**+**^**–TFA** and **TT**^**2+**^ with Arenes

**Figure 1:**
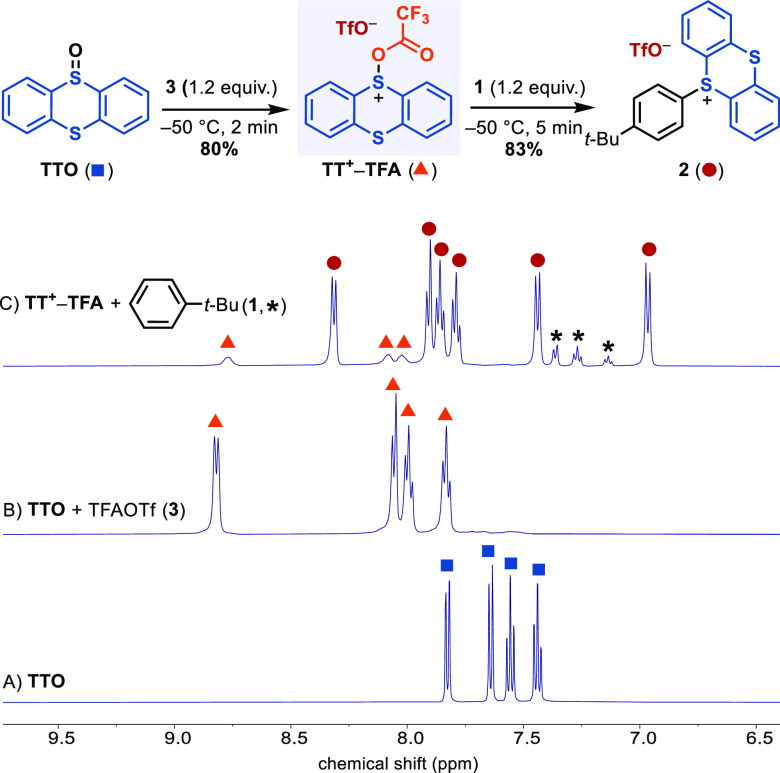
^1^H NMR studies on the characterization of **TT**^**+**^**–TFA** and its reactivity toward arenes. All spectra were measured at −50 °C in CD_2_Cl_2_.

With the aim of evaluating the potential role of **TT**^**+**^**–TFA** and **TT**^**2+**^ in the thianthrenation of **1** with **TTO**, we carried out NMR experiments to detect the intermediate species and assess its reactivity toward **1** (Figure 1). Upon addition of **3** to a solution of **TTO** (blue squares) in CD_2_Cl_2_ at −50 °C, the starting material was immediately consumed, giving rise to a new species (orange triangles). The ^1^H NMR signals corresponding to the thianthrene core in the new product are considerably more deshielded in comparison with those of **TTO**, and the existence of four sets of signals rules out a major presence of symmetrical **TT**^**2+**^. An analysis of the ^19^F NMR spectrum revealed two new singlets that integrate for three fluorine resonances each at −72.7 and −79.2 ppm, respectively, and are consistent with assignment to CF_3_COO– and CF_3_SO_3_– fragments, respectively. Identical ^1^H and ^19^F spectra were obtained when **TTO** was treated with TFAA in the presence of TfOH (see the Supporting Information). These data are consistent with the structure of **TT**^**+**^**–TFA**. We next examined the reactivity of **TT**^**+**^**–TFA** toward arenes. While maintaining the sample at −50 °C, we added 1.2 equiv of the arene **1** to a solution of **TT**^**+**^**–TFA** and observed nearly complete conversion of **TT**^**+**^**–TFA** to the aryl thianthrenium salt **2** in less than 5 min at −50 °C. On the basis of these data and using the Eyring equation, we estimate an upper limit for the barrier of C–H thianthrenation of **1** to be ∼15 kcal/mol from **TT**^**+**^**–TFA** (Δ*G*_223_^⧧^ = 15 kcal/mol, *t*_1/2_ = 1.25 min). These experiments confirm the chemical and kinetic competence of **TT**^**+**^**–TFA** to undergo aromatic C–H thianthrenation under the reaction conditions in the absence of added acid.

**Scheme 7:**
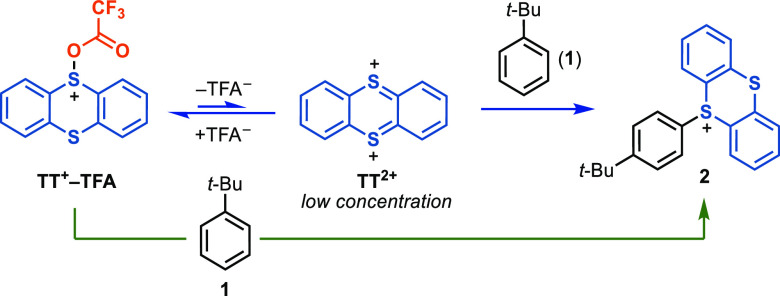
Possible Reaction Pathways from **TT**^**+**^**–TFA** to **2**

NMR studies allowed us to determine experimentally a fast reaction of **TT**^**+**^**–TFA** with arenes at low temperatures; however, the mechanism of the process remained unclear. **TT**^**2+**^ could not be detected under our experimental conditions, but its participation as an intermediate via a slow generation from **TT**^**+**^**–TFA** and a subsequent fast reaction with arenes could prevent the accumulation of this species because it would be consumed upon generation (Scheme 7).

**Figure 2:**
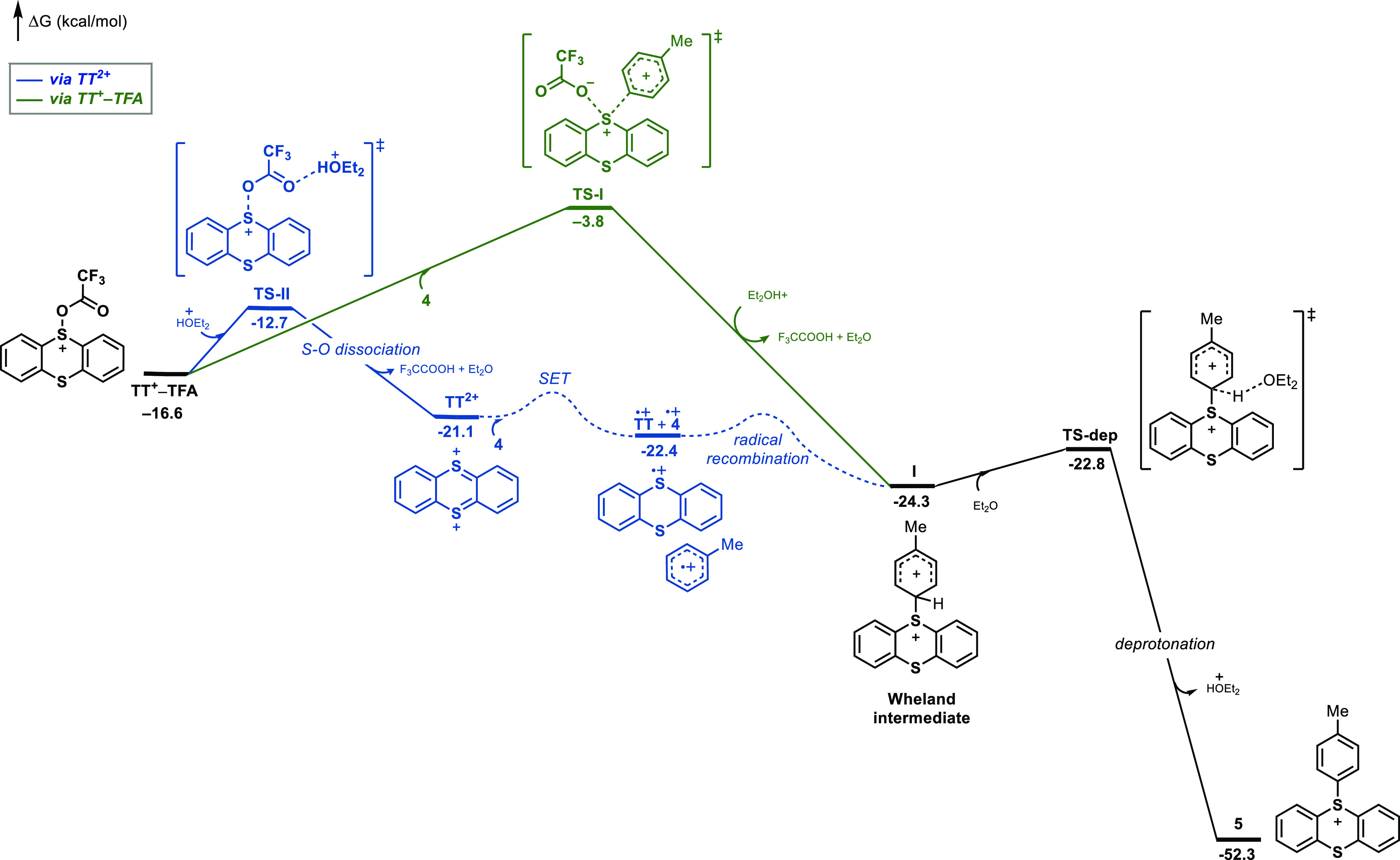
DFT evaluation of the reaction profile of aromatic C–H thianthrenation of toluene. Gibbs free energies are all relative to **TTO.** The transition states represented by the dashed lines have not been located computationally.

Experimental differentiation of the two pathways shown in Scheme 7 is challenging due to the kinetic indistinguishability, low solubility at −50 °C, and the preclusion to study the reaction order in TFA^–^ due to its deleterious reaction with **TT**^**+**^**–TFA** to form **TTO**. For this reason, we studied both reactivity profiles with toluene (**4**) by DFT (Figure 2). The reaction *via* nucleophilic substitution of TFA^–^ by the arene at the sulfur atom of **TT**^**+**^**–TFA** (green pathway) involves a barrier of 12.8 kcal/mol (**TS-I**, relative to **TT**^**+**^**-TFA**) to produce the Wheland intermediate **I**. The computed barrier is found within the experimentally determined higher limit from **TT**^**+**^**–TFA** and **1** to product (Figure 1). The alternative pathway (in blue) based on **TT**^**2+**^ was also evaluated. The heterolytic cleavage of the S–OOCCF_3_ bond accompanied by protonation by Et_2_OH^+^ to generate **TT**^**2+**^ has a barrier of only +3.9 kcal/mol (**TS-II**). The subsequent pathway **TT**^**2+**^ → **I** can proceed through single-electron transfer (SET) and radical recombination or polar electrophilic addition; computationally, polar electrophilic addition is slightly less favorable (see the Supporting Information). Although there is no potential energy barrier shown, the electron transfer between **TT**^**2+**^ and **4** in solution requires diffusion of the species together and solvent reorganization treated by Marcus theory, which has an intrinsic barrier on the order of 3–6 kcal/mol. Furthermore, the dication is present in only low concentrations, leading to a more significant free energy of activation. Similarly, radical combination in the conversion of the radical pair to the Wheland intermediate will have a free energy barrier due to unfavorable entropy. Because of the conversion of open-shell to closed-shell species in this step, and the necessity of variational transition-state calculations to locate such transition states, we have not attempted to locate the transition states for conversions of radical pairs to closed-shell intermediates and the reverse.

The proton transfer to the TFA leaving group could also facilitate the reaction via nucleophilic substitution at sulfur, but in attempts to compute such a transition state, we find that acid causes the loss of trifluoracetic acid, which is not accelerated by a neighboring aromatic molecule. Only the S_N_1 type dissociation to **TT^2+^** followed by a reaction with arene could be identified under acidic conditions, thus being consistent with **TT**^**2+**^ acting as the reactive species under acidic reaction conditions.

In the absence of acid, as under the previously investigated basic condition with TFAOTf (Scheme 5 and Figure 1), the S_N_2 mechanism via **TS-I** is favored, with a barrier of 12.8 kcal/mol. Moreover, while the barriers to form **TT**^**2+**^ via S–OOCCF_3_ bond cleavage and the Wheland intermediate **I** via S_N_2 in basic conditions are similar, it is expected that the contribution of both pathways is also substrate-dependent, where more nucleophilic arenes favor the reaction with **TT**^**+**^**–TFA** and more electron deficient arenes react preferentially with **TT**^**2+**^ (see the Supporting Information for the energetics under basic conditions). Subsequent deprotonation of the σ-complex **I** (**TS-dep**) is selectivity-determining in the reaction,^85^ in good agreement with the experimentally determined kinetic isotope effect of *k*_H_/*k*_D_ = 1.9 observed in intermolecular competition experiments.^32^ On consideration of all of the above, experimental and computational studies predict a fast reaction of **TT**^**+**^**–TFA** with arenes either by a direct path (under basic conditions; see the Supporting Information) or *via* the intermediacy of **TT**^**2+**^ and identify **TT**^**+**^**–TFA** as the key reactive species for C–H thianthrenation.

### Origin of Regioselectivity in C–H Thianthrenation

#### Site Selectivity in Different Thianthrenation Protocols

First, we analyzed the regioselectivity of the thianthrenation reaction of toluene (**4**) under the standard reaction conditions, which is expected to proceed through **TT**^**2+**^ or **TT**^**+**^**–TFA** (Table 1). High *para* selectivity (>100:1 with respect to both *meta* and *ortho* isomers) was observed for the formation of aryl sulfonium salt **5**. Likewise, in the absence of TFAA but with excess sulfuric acid, conditions that are expected to go through **TT**^**2+**^,^80^ an almost identical *para* selectivity was observed. Moreover, the reaction of **4** with independently prepared **TT**^**•+**^ provides the product again with virtually identical *para* selectivity. A similar outcome was observed when TFAOTf was used as the acetylating reagent under basic conditions, likely involving **TT**^**+**^**–TFA**. Given the high selectivity for all four independent reactions, it is conceivable that the selectivity is determined at a common, post-Wheland-intermediate step in the reaction profile: i.e., deprotonation of the σ-complex **I**.

**Table 1:**
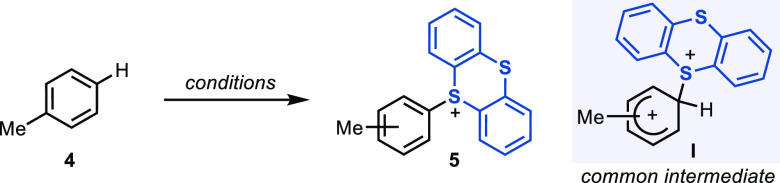
Regioselectivity on the Thianthrenation of Toluene under Different Conditions

entry	conditions	proposed	*p*/*o*	*p*/*m*
1	**TTO**, TFAA, HBF_4_·OEt_2_, MeCN	**TT**^**2+**^**/TT**^**+**^**-TFA**	106	132
2	**TTO**, neat H_2_SO_4_	**TT**^**2+**^	122	132
3	[**TT**^**•+**^]BF_4_, MeCN	**TT**^**•+**^	114	127
4	**TTO**, TFAOTf, K_2_CO_3_, MeCN	**TT**^**+**^**-TFA**	75	125

#### Analysis of Thianthrenation vs Other S_E_Ar Reactions: Source of *p*/*m* and *p*/*o* Regioselectivity

In previous preliminary studies on the thianthrenation of arenes with **TFTO**,^32^ a Hammett analysis indicated a significant development of positive charge on the aromatic ring (ρ = −11) in the C–S bond-forming transition state, in line with the involvement of cationic Wheland intermediates of the type **I**. To investigate the possible correlation between ρ and site selectivity, we compared both values with those displayed by other S_E_Ar reactions (Table 2).^5,86−88^

**Table 2:** Comparison of Hammett Parameters and Regioselectivity for Different S_E_Ar Reactions^a^

reaction	ρ	*p*/*m* in toluene	*p*/*o* in toluene
bromination	–12	220	2
**thianthrenation**^b^	**–11**	**206**	**144**
chlorination	–9	82	0.66
acetylation	–9	54	162
nitration	–6	17	0.54
electroiodination	–6	12	1
mercuration	–4	5	2
alkylation	–2	1.8	1.7

a Data obtained from refs (5 and 86−88).

b Data for reactions with **TFTO**.

**Figure 3:**
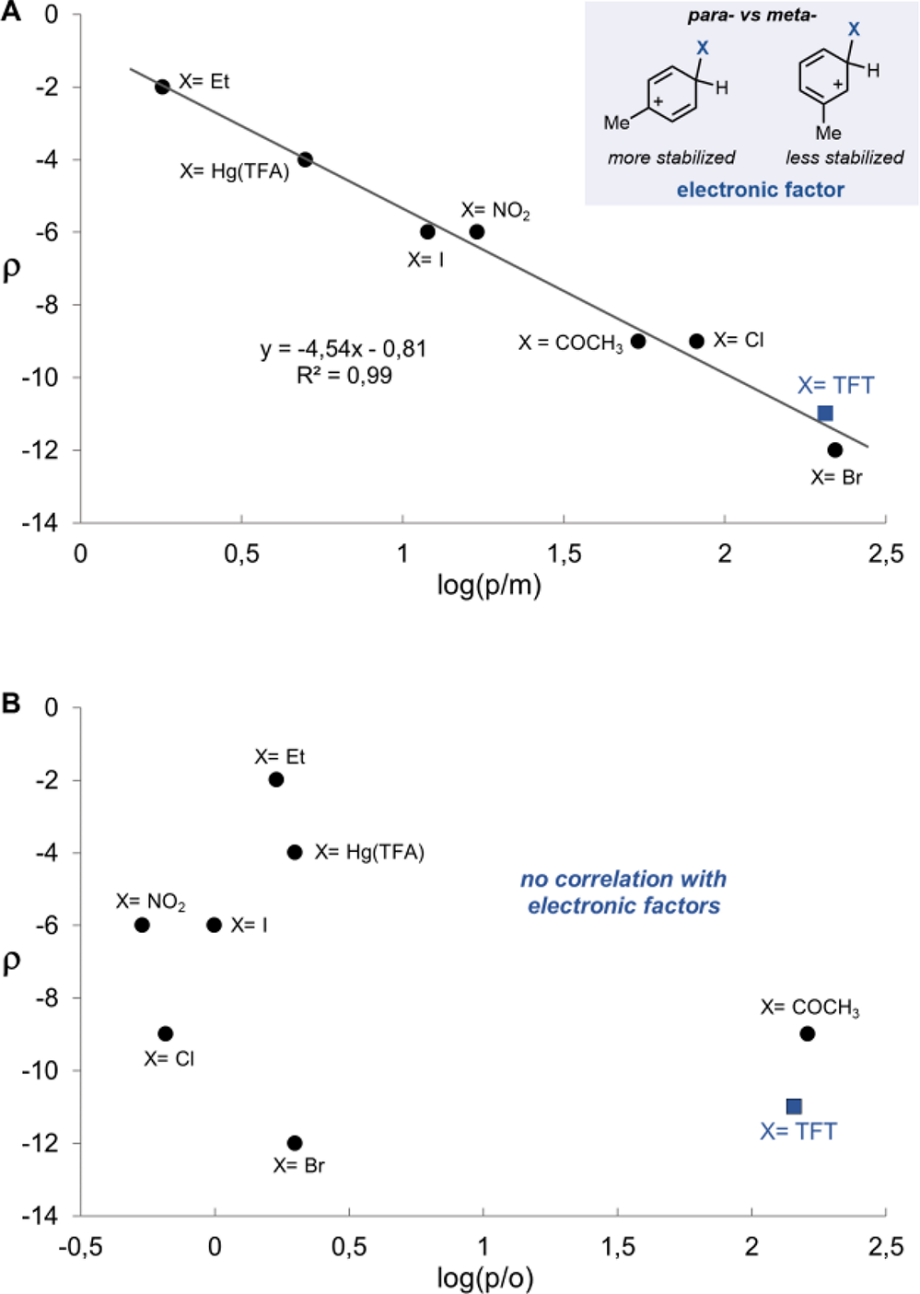
(A) Hammett parameter vs *para*/*meta* selectivity in S_E_Ar presented in Table 2. (B) Hammett parameter vs *para*/*ortho* selectivity in S_E_Ar presented in Table 2.

S_E_Ar reactions of monosubstituted arenes with electron-donating substituents commonly display selectivity to afford *ortho*- and *para*- over *meta*-substituted products, due to the better stabilization of the cationic charge on the Wheland intermediate.^2^ Accordingly, it is reasonable that a higher σ-complex character in the transition state, evidenced by more negative ρ values, will result in higher *p*/*m* selectivities. On the basis of an analysis developed by Brown and Stock in the 1960s,^89,90^ we propose here an intuitive linear free energy relationship to correlate the Hammett value ρ for any given S_E_Ar with its *para* vs *meta* selectivity, as shown in Figure 3A. The predictive power of this analysis lies in the ability to predict the extent of *para* over *meta* selectivity solely on the basis of the Hammett value. In contrast, the Hammett value of a given S_E_Ar reaction does not display a similar correlation with *para* versus *ortho* selectivity, as can be seen in Figure 3B, which may be the reason that Hammett values are not commonly considered when regioselective S_E_Ar reactions are targeted. In fact, a similar observation was reported by Houk and Perrin following an analysis of *p*/*o* selectivities analogous to that of *p*/*m* by Brown and Stock.^91^*We therefore summarize that para vs meta selectivity is electronic in nature and can be predicted by the Hammett value ρ, while the para vs ortho selectivity is not electronic in nature and does not correlate with the Hammett value ρ.*

Arene bromination, for example, is typically considered a highly selective reaction with respect to *para* vs *meta* differenciation (*p*/*m* = 220), a consequence of the late TS for the electrophilic addition (ρ = −12) that strongly resembles the arenium intermediate. Similarly, the large negative Hammett value (ρ = −11) that we measured for thianthrenation is well correlated with the observed excellent *para vs meta* selectivity (*p*/*m* = 206). The accurate prediction by the free linear energy relationship thus establishes the prevalent relevance of the electronic stabilization in the product-determining transition state to determine *para* vs *meta* selectivity in thianthrenations.

**Scheme 8:**
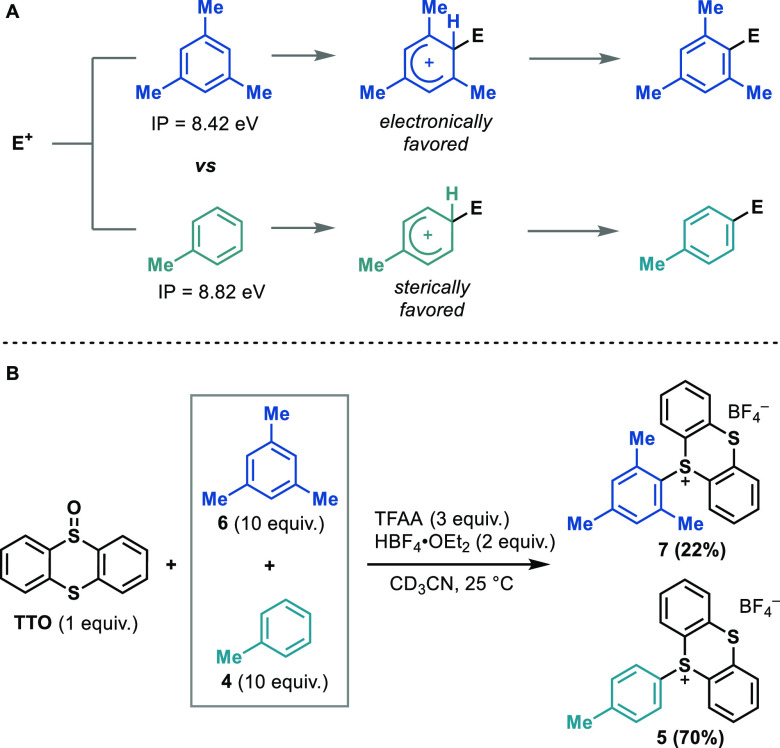
Steric Effects in Thianthrenations: Intermolecular Competition Experiment between Toluene and Mesitylene

Bromination and thianthrenation have similar ρ values and *p*/*m* selectivities yet substantially different *para vs ortho* selectivities: namely, 144:1 for thianthrenation and 2:1 for bromination. While electronic effects can explain the *para*/*meta* selectivity, they cannot rationalize the excellent *para*/*ortho* differentiation. An obvious difference between highly selective thianthrenation and *para-*/*ortho*-unselective halogenation reactions is the size of the thianthrene heterocycle in comparison to monatomic halides. Steric effects in S_E_Ar reactions have been addressed in the past^92^ and can alter the regioselectivity.^6,93^ To analyze the influence of steric effects in aromatic thianthrenation, we performed a competition experiment between toluene and mesitylene (Scheme 8). Due to a lower ionization potential^94^ and higher nucleophilicity,^95^ mesitylene reacts more quickly than toluene in most S_E_Ar reactions.^96^ However, thianthrenation is selective for toluene over mesitylene in an intermolecular competition experiment (Scheme 8B). When they are taken together with the observed primary kinetic isotope effect, these results support the hypothesis of product-determining deprotonation being slower at the more highly substituted mesitylene-based Wheland intermediate. To address whether a similar steric effect can also rationalize the *p*/*o* selectivity of thianthrenation, we next evaluated by DFT the product-determining deprotonation of the different *ortho*, *meta*, and *para* Wheland intermediates in more detail.

#### Computational Study of the Selectivity-Determining Deprotonation of Wheland Intermediates

**Figure 4:**
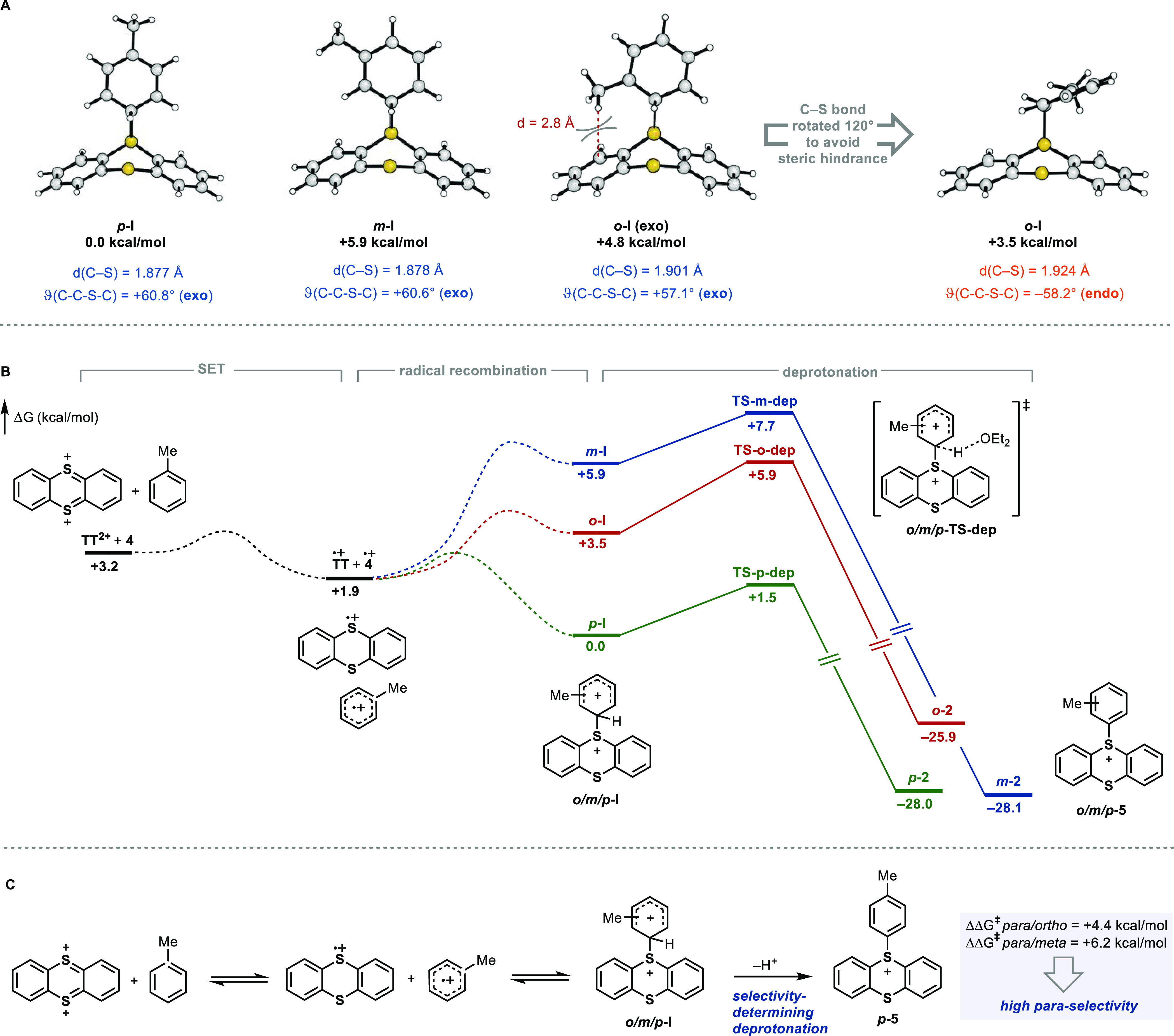
(A) Structural analysis of isomeric σ-complex intermediates. Distances (*d*) are given in Å and dihedral angles (θ) in deg. (B) Reaction profile. Free energies are all relative to *p*-I. (C) Reversible addition and selectivity-determining deprotonation of Wheland intermediates. Transition states indicated by dashed lines have not been located computationally.

Of the three different σ-complexes for thianthrenation of toluene (***o***-/***m-***/***p*****-I**) the *para* isomer (***p*****-I**) is computed to be the most stable (Figure 4A). The optimized structure of ***p*****-I** presents the thianthrene moiety in an exocyclic arrangement, while the proaryl substituent adopts a flagpole position with respect to the thianthrene heterocycle, as discussed previously.^39^ A virtually identical arrangement is found for the *meta* isomer. No steric effects that would explain the ***p*****-I** to ***m*****-I** energy difference of 5.9 kcal/mol could be identified; the energy difference can be explained by conventional hyperconjugative effects as discussed above (Hammett value of ρ = −11). For the ***o*****-I** structure, a significantly different arrangement was calculated that we refer to as an *endo* conformation, in which a rotation of the proarene with respect to the thianthrene moiety of about 120° avoids an eclipsing interaction between thianthrene and the methyl group of toluene in the *exo* conformation. Computationally, the *exo* conformer ***o*****-I-exo** lies 1.3 kcal/mol higher in energy than the *endo* isomer, which itself lies 3.5 kcal/mol higher in energy than the *para* isomer ***p*****-I**. These observations reflect the energetic cost of steric hindrance in the *ortho*-Wheland isomer. Computation of the transition states for subsequent deprotonation (**TS-dep-*****o*****/*****-m*****/*****-p***, Figure 4B) reveal early transition states that resemble the Wheland intermediates in geometry and relative energies, in line with Hammond’s postulate.^97^ Accordingly, the relatively high energy differences observed in ***o-*****/*****m-*****/*****p*****-I** are also evident at **TS-dep-*****o-*****/*****m-*****/*****p***, in agreement with the >100:1 selectivity observed experimentally. Due to the lack of similar interactions in halogenation reactions, the energy difference between the *ortho* and *para* σ-complexes is much smaller (ΔΔ*G* ≈ 0.2 kcal/mol)^20^ and results in low *o*/*p* selectivity. The exquisite positional selectivity for thianthrenation is rare in aromatic C–H functionalization and can thus be rationalized through electronic and steric control to determine both *p*/*m* and *p*/*o* selectivity, respectively.

In line with the observed KIE, calculations predict that the formation of the Wheland intermediates is reversible; homolytic cleavage of the C–S bond regenerates **TT**^**•+**^ and **4**^**•+**^ (Figure 4B). This process enables the small amounts of *o* and *m* isomers potentially generated kinetically to all be funneled to the most stable ***p*****-I**, before irreversible deprotonation affords the final aryl thianthrenium product ***p*****-5** (Figure 4C). Formation of the Wheland intermediate may also contribute to the selectivity if the formation of the *meta* isomer is too high in energy to occur, while reversible Wheland intermediate formation is unambiguously established by the primary KIE. The formation of the different Wheland intermediates may occur via different pathways (e.g., through **TT**^**+**^**–TFA**, **TT**^**2+**^, or **TT**^**•+**^; Table 1) but s*electivity in all cases is identical and is determined in the comparatively slow deprotonation step by the order of the energies of the energetically accessible Wheland intermediates*.

#### Comparison with Other Sulfoxides: Importance of a Stable Radical Cation for the Equilibration of Arenium Intermediates

**Scheme 9:**
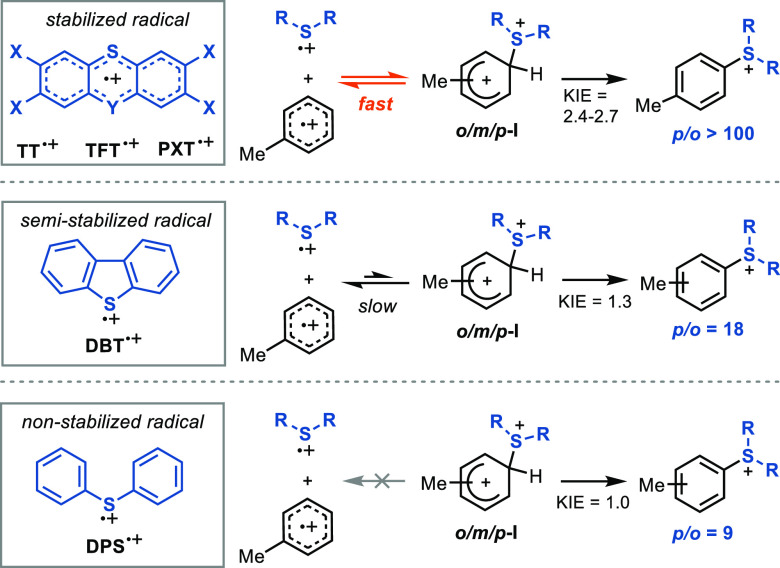
Reversible Homolytic Cleavage in C–H Functionalization of Arenes with Different Sulfoxides

According to our calculations, the facile dissociation of **I** into **TT**^**•+**^ and ArH^**•+**^ is responsible for the fast equilibrium of the different isomers of the Wheland intermediate before an irreversible, product-determining deprotonation. The ease of homolytic cleavage of the C–S bond can be rationalized by the stability of the persistent radical **TT**^**•+**^.^98−100^ Its high stability sets **TT**^**•+**^ apart from most other R_2_S^•+^ reagents and may contribute to its higher selectivity in S_E_Ar reactions. For a quantitative comparison, we evaluated the reactivity of different sulfoxides R_2_SO in C–H functionalization and performed cyclic voltammetry to evaluate the behavior of the corresponding R_2_S^•+^ (Table 3). All thianthrene analogues (**TFT**, **TT**, and **PTX**) display a reversible single-electron oxidation, consistent with persistent radical formation, and a primary KIE, consistent with reversible Wheland intermediate formation. All of these reactions are characterized by excellent *para* selectivity (>100:1 in all cases) over both *ortho* and *meta* positions. On the other hand, the related sulfides **DBT** and **DPS** do not form persistent radicals, display a significantly lower KIE if any, and are characterized by a markedly lower *p*/*o* selectivity. In view of these results, we propose that the reversible formation of the C–S bond contributes to the site selectivity in C–H functionalizations mediated by sulfoxides (Scheme 9). For this process to be efficient, it is thus important that homolytic cleavage results in a low-energy (persistent) radical cation on the sulfur electrophile.

**Table 3:**
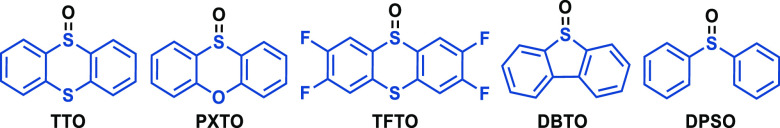
Comparison of Site Selectivity and KIE for the Reactions of Different Sulfoxides with Toluene

sulfoxide	*p*/*o*^a^ in **5**	*p*/*m*^a^ in **5**	*k*_H_/*k*_D_^b^	*E*(R_2_S^**•+**^/R_2_S) (V) vs SCE	reversible?
**TTO**	106	133	2.7	+1.24	Y
**TFTO**	144	206	2.4	+1.42	Y
**PXTO**	99	190	2.7	+1.20	Y
**DBTO**	18	114	1.3	+1.55	N
**DPSO**	9	86	1.0	+1.47	N

a Activation method: TFAA + HBF_4_·OEt_2_.

b From an intermolecular competition of **4/4-*****d***_**8**_.

### Lessons Learned from Thianthrenation: Guidelines toward the Design of Highly *para* Selective C–H Functionalizations

**Figure 5:**
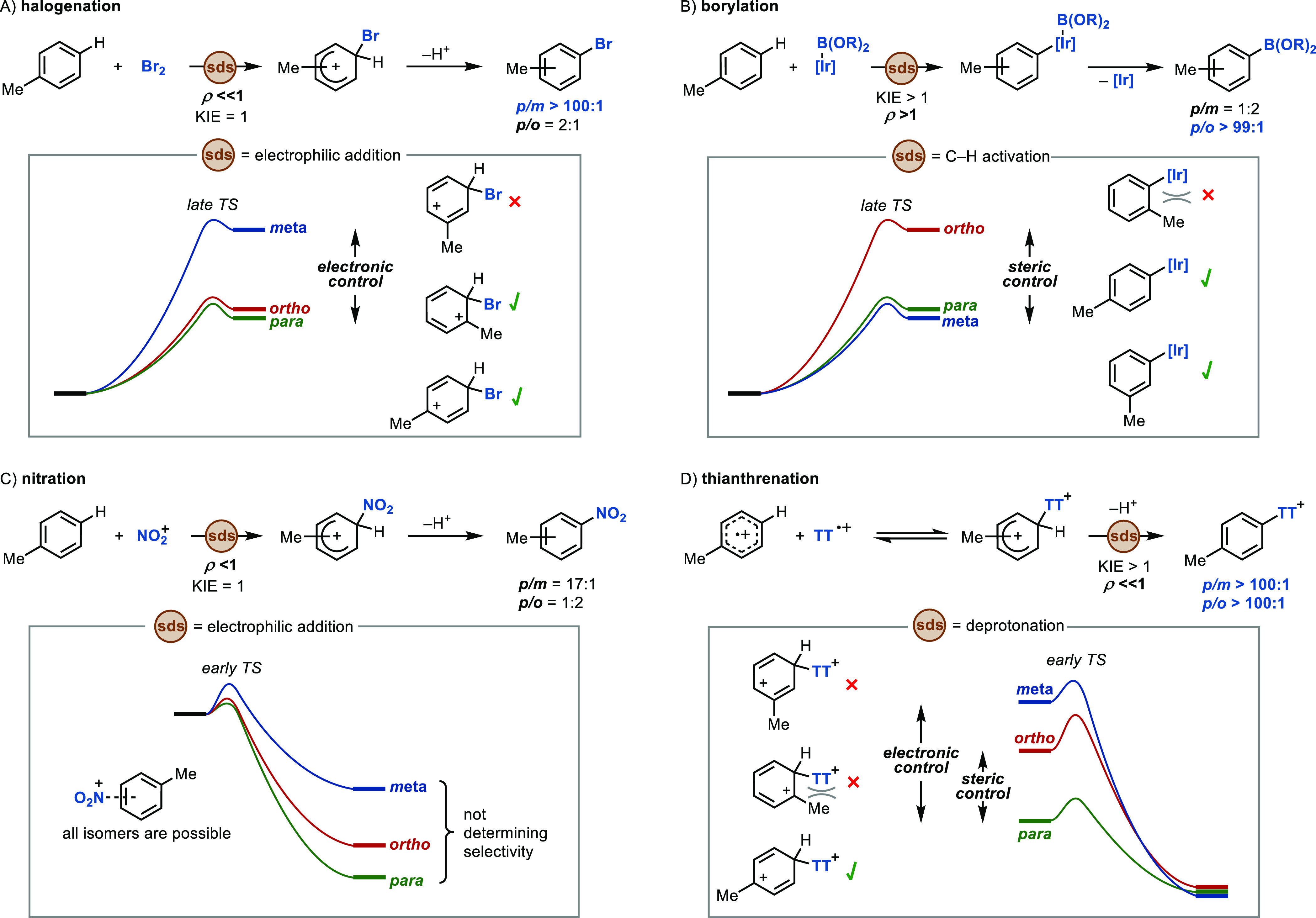
Analysis of site selectivity on toluene for representative C–H functionalizations. Data from (A)–(C) are extracted from refs (20 and 101). sds = selectivity-determining step.

In light of the data discussed above, we attempt to rationalize the regioselectivity obtained in different C–H functionalizations with special emphasis on the distinct features found in thianthrenations (Figure 5). With the aim of providing valuable tools to facilitate the development of future selective transformations, we summarize below the most important aspects that we consider relevant to achieve high site selectivity in aromatic C–H functionalizations:

#### Message #1: Strong Dependence of the Energy of Wheland Intermediates on Electronic (ρ ≪ 0) and Steric Effects

In general, halogenations exhibit a reaction profile in which the electrophilic addition to the arene is the rate- and product-determining step (Figure 5A).^2^ Due to the character of the late TS of addition, site selectivity in halogenations is largely determined by the relative stability of Wheland intermediates.^2,5,15^ Brominations typically display excellent *p*/*m* selectivity due to the different stabilizations of the positive charge (*p* ≈ *o* ≫ *m*) in the arenium intermediate (electronic control). In contrast, due to the small effective size of monatomic halogens, similar energies can be found for the *ortho* and *para* σ-complexes (ΔΔ*G* ≈ 0.2 kcal/mol).^20^ While good *para* vs *ortho* selectivities can be attained in electronically or sterically biased subtrates, the lack of steric control ultimately results in low *o*/*p* selectivity on a variety of substrates such as toluene.

Undirected aromatic C–H borylations (Figure 5B) proceed via a different mechanism, typically involving a metal-mediated C–H activation with a late TS as the rate-determining step.^101^ The dissimilar mechanistic features of borylation in comparison with S_E_Ar reactions result in distinct site selectivity. Accordingly, borylations are largely unaffected by electronic properties (e.g., *ρ =* 3.3^102^) but are highly sensitive to steric requirements. In fact, the C–H borylation of mesitylene is considered challenging and was not possible until recently.^103^ These features result in reactions that favor *para* and *meta* over *ortho* isomers (ΔΔ*G*^⧧^_*para-ortho*_ = +2.5 kcal/mol) but afford, in general, low *p*/*m* selectivity (ΔΔ*G*^⧧^_*para-meta*_ = −0.1 kcal/mol).^101^

Thianthrenation reactions (Figure 5D), on the other hand, involve Wheland intermediates that are affected by both electronic and steric factors, which provides a large energy difference between isomeric σ-complexes that ensures high levels of both *p*/*m* and *p*/*o* selectivity. This aspect constitutes a distinguishing feature of thianthrenation in comparison to all other linchpin-introducing arene functionalization reactions.

#### Message #2: Transition State Resembles Wheland Intermediate in the Selectivity-Determining Step: Late TS for Electrophilic Attack or Early TS for Deprotonation

As found in other S_E_Ar reactions, isomeric Wheland intermediates in nitrations display different energies that could be sufficient to induce site selectivity (ΔΔ*G*^⧧^_*para-ortho*_ = +2.8 kcal/mol, ΔΔ*G*^⧧^_*para-meta*_ = +5.4 kcal/mol).^20^ However, nitrations are often rather unselective reactions (Figure 5C). The explanation for this apparent contradiction is rooted in the mechanism of nitration, in which electrophilic addition of NO_2_^+^ is the selectivity-determining step. According to the Hammond postulate, the high exothermicity of this step results in an early TS with little resemblance to the arenium intermediate (as indicated by the small ρ value). Nitration thus exemplifies how the energetic differentiation of σ-complexes is not sufficient to achieve high regioselectivity if the TS for the product-determining step is not early (for electrophilic additions) or late (for deprotonations). The energy difference of the σ-complexes in thianthrenation plays an important role in selectivity due to the early TS found in the deprotonation of the arenium intermediates (Figure 5D).

#### Message #3: Reversible Electrophilic Addition

**Scheme 10:**
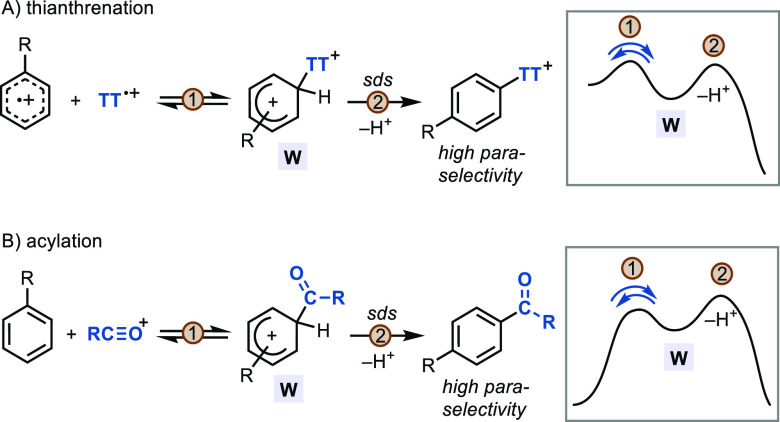
Reversible Addition of Electrophile in Highly *para* Selective C–H Functionalization

The low barrier of homolytic and heterolytic cleavage of the C–S bond in the σ-complexes of thianthrenation (Figure 4B) enable a fast isomerization to the most stable arenium before irreversible deprotonation (Scheme 10A). Acetylations also have an irreversible, selectivity-determining deprotonation step (KIE = 2.1)^87^ and a similar predicted energy profile (Scheme 10B).^104^ In this case, a heterolytic cleavage of the C–COMe bond from the arenium intermediate to give an acylium ion and the aromatic substrate requires only +1.6 kcal/mol, while the irreversibly deprotonated TS is located +6.5 kcal/mol higher in energy than the σ-complex. As can be seen in Table 2, high selectivities for both *p*/*m* and *p*/*o* can be found in some Friedel–Crafts acylations (>95% *para* isomer in toluene^87,105^).

## Conclusions

We have investigated the mechanism of aromatic C–H thianthrenation and the source of their unusually high site selectivity by experiments and theory. The reaction proceeds through the formation of reactive electrophilic species derived from thianthrene *S*-oxide: i.e., the thianthrene dication and trifluoroacetylated derivative that are studied here in detail for the first time. According to our results, both reactive species can be generated under the reaction conditions and participate in electrophilic addition to the aromatic substrates with relatively low barriers, which enables a fast reaction even at low temperature. The formation and subsequent deprotonation of the arenium intermediates were identified as the selectivity-determining steps. The high *para* selectivity was rationalized and explained by a combination of a polar contribution that favors the *para* over the *meta* isomer and by a steric effect that favors the *para* over the *ortho* isomer in the different constitutional Wheland isomer intermediates, combined with a reversible interconversion between them before product-determining deprotonation. We introduced the analysis of a linear free energy relationship, in which the selectivity of an S_E_Ar reaction can be predicted simply on the basis of the Hammett value of the transformation, in comparison to thianthrenation with the metrics of other S_E_Ar reactions, and extracted valuable conclusions that should aid in the development of other selective C–H functionalizations.

## Supporting Information Available

Experimental procedures, characterization data, and details of mechanistic and computational studies (PDF)
Cartesian coordinates for the calculated structures (XYZ)

## Supplementary Material

ja1c06281_si_001.pdf

ja1c06281_si_002.xyz
