# Supplementary material for: High Site Selectivity in Electrophilic Aromatic Substitutions: Mechanism of C–H Thianthrenation
Source: J Am Chem Soc. 2021 Sep 21;143(39):16041–54. doi: 10.1021/jacs.1c06281 (PMC8499029; doi:10.1021/jacs.1c06281)
Supplement: Supplementary file 1 — ja1c06281_si_001.pdf [file ja1c06281_si_001.pdf]

SUPPORTING INFORMATION

# High Site-Selectivity in Electrophilic Aromatic Substitutions: Mechanism of C–H Thiantrenation

Fabio Juliá[a], Qianzhen Shao[b], Meng Duan[b], Matthew B. Plutschack[a], Florian Berger[a], Javier Mateos[a], Chenxi Lu[b], Xiao-Song Xue[b], K. N. Houk[b]*, and Tobias Ritter[a]*

[a] Max-Planck-Institut für Kohlenforschung, Kaiser-Wilhelm Platz 1, D-45470 Mülheim an der Ruhr, Germany.

[b] Department of Chemistry and Biochemistry, University of California, Los Angeles, California 90095-1569 USA

*[a] e-mail: ritter@kofo.mpg.de

*[b] e-mail: houk@chem.ucla.edu

# TABLE OF CONTENTS

TABLE OF CONTENTS ..... 1

MATERIALS AND METHODS ..... 5

EXPERIMENTAL DATA ..... 6

Experimental procedures ..... 6

General procedure for thianthrenation of arenes with thianthrene-S-oxide/TFAA/$HBF_4OEt_2$ ..... 6

General procedure for thianthrenation of arenes with thianthrene-S-oxide/TFAOTf ..... 7

General procedure for thianthrenation of arenes with thianthrenium radical cation tetrafluoroborate ..... 7

General procedure for thianthrenation of arenes with thianthrene-S-oxide in neat $H_2SO_4$ ..... 8

Characterization of new sulfonium salts ..... 8

*tert*-butylbenzene derived thianthrenium salt **2**, *para*-isomer ..... 8

indole derived thianthrenium salt **S1** ..... 9

Preparation of *ortho*-, *meta*- and *para*-isomers of tolyl sulfonium salts ..... 10

Toluene derived thianthrenium salt **5**, *para*-isomer ..... 10

Toluene derived thianthrenium salt **5**, *meta*-isomer ..... 10

Toluene derived thianthrenium salt **5**, *ortho*-isomer ..... 11

Toluene derived tetrafluorothianthrenium salt **S2**, *para*-isomer ..... 12

Toluene derived tetrafluorothianthrenium salt **S2**, *meta*-isomer ..... 13

Toluene derived tetrafluorothianthrenium salt **S2**, *ortho*-isomer ..... 13

Toluene derived phenoxathiinium salt **S3**, *para*-isomer ..... 14

Toluene derived phenoxathiinium salt **S3**, *meta*-isomer ..... 15

Toluene derived phenoxathiinium salt **S3**, *ortho*-isomer ..... 16

Toluene derived dibenzothiophenium salt **S4**, *para*-isomer ..... 16

Toluene derived dibenzothiophenium salt **S4**, *meta*-isomer ..... 17

Toluene derived dibenzothiophenium salt **S4**, *ortho*-isomer ..... 18

Toluene derived diphenylsulfonium salt **S5**, *para*- and *ortho*-isomers ..... 19

Toluene derived diphenylsulfonium salt **S5**, *meta*-isomer ..... 20

Aromatic thianthrenations in the absence of TFAA ..... 20

Table S1. Yields of **2** with different acids ..... 20

Reaction profile of the thianthrenation of toluene in the absence of TFAA ..... 21

NMR studies on acylated derivatives of thianthrene-S-oxide ..... 22

Characterization and reactivity of **$TT^+$-TFA** ..... 22

Generation of **$TT^+$-TFA** using TFAA and TfOH ..... 23

Generation of **$TT^+$-OAc** using $Ac_2O$ and TfOH ..... 25

Reactivity modulation of activated thianthrene-S-oxide ........ 26

Site selectivity on thrianthrenations ........ 27

Selectivity determination on thianthrenation with thianthrene-S-oxide/TFAA/$HBF_4OEt_2$ ........ 27

Selectivity determination on thianthrenation with thianthrene-S-oxide in $H_2SO_4$ ........ 29

Selectivity determination on thianthrenation with thianthrenium radical cation ........ 30

Selectivity determination on thianthrenation with thianthrene-S-oxide/TFAOTf/$K_2CO_3$ ........ 32

Site selectivity on C–H functionalization with other sulfoxides ........ 33

Selectivity determination on C–H functionalization with tetrafluorothianthrene-S-oxide/TFAA/$HBF_4OEt_2$ ........ 33

Selectivity determination on C–H functionalization with phenoxathiin-S-oxide/TFAA/$HBF_4OEt_2$ ........ 35

Selectivity determination on C–H functionalization with dibenzothiophene-S-oxide/TFAA/$HBF_4OEt_2$ 36

Selectivity determination on C–H functionalization with diphenylsulfoxide/TFAA/$HBF_4OEt_2$ ........ 38

Brown-Stock plot ........ 39

Table S2. ........ 39

Kinetic Isotope Effect (KIE) determinations ........ 40

KIE on toluene for thianthrene-S-oxide ........ 40

KIE on toluene for tetrafluorothianthrene-S-oxide ........ 41

KIE on toluene for phenoxtathiin-S-oxide ........ 42

KIE on toluene for dibenzothiophene-S-oxide ........ 43

KIE on toluene for diphenylsulfoxide ........ 44

Kinetic isotope effect on benzene for thianthrene-S-oxide under different conditions ........ 44

Mesitylene vs toluene competition experiments ........ 45

Experiments with deuterium-labeled thianthrene-S-oxide ........ 47

Preparation of **TTO-*d₂*** ........ 47

Thianthrenation of *tert*-butylbenzene with **TTO-*d₂*** ........ 48

Cyclic voltammetry ........ 50

Open-circuit measurements ........ 52

COMPUTATIONAL DATA ........ 53

Computational Methods ........ 53

Intrinsic instability of **[ArH-TT]**$^{\bullet+}$ ........ 53

Radical recombination vs Polar electrophilic addition ........ 53

**TT**$^{2+}$ vs **TT**$^{+}$**-TFA** in basic condition ........ 54

ωB97X-D geometries for all optimized compounds and transition states ........ 55

SPECTROSCOPIC DATA ........ 1

$^{1}$H NMR of *tert*-butylbenzene derived thianthrenium salt **2**, *para*-isomer ........ 1

$^{13}$C NMR of *tert*-butylbenzene derived thianthrenium salt **2**, *para*-isomer ........ 1

$^{1}$H NMR of toluene derived thianthrenium salt **5**, *para*-isomer ........ 2

$^{13}$C NMR of toluene derived thianthrenium salt **5**, *para*-isomer ........ 3

$^{1}$H NMR of toluene derived thianthrenium salt **5**, *meta*-isomer ........ 4

$^{13}$C NMR of toluene derived thianthrenium salt **5**, *meta*-isomer ........ 5

$^{1}$H NMR of toluene derived thianthrenium salt **5**, *ortho*-isomer ........ 6

$^{13}$C NMR of toluene derived thianthrenium salt **5**, *ortho*-isomer ........ 7

$^{1}$H NMR of indole derived thianthrenium salt **S1** ........ 8

$^{13}$C NMR of indole derived thianthrenium salt **S1** ........ 9

$^{1}$H NMR of toluene derived tetrafluorothianthrenium salt **S2**, *para*-isomer ........ 10

$^{13}$C NMR of toluene derived tetrafluorothianthrenium salt **S2**, *para*-isomer ........ 11

$^{19}$F NMR of toluene derived tetrafluorothianthrenium salt **S2**, *para*-isomer ........ 12

$^{1}$H NMR of toluene derived tetrafluorothianthrenium salt **S2**, *meta*-isomer ........ 13

$^{13}$C NMR of toluene derived tetrafluorothianthrenium salt **S2**, *meta*-isomer ........ 14

$^{19}$F NMR of toluene derived tetrafluorothianthrenium salt **S2**, *meta*-isomer ........ 15

$^{1}$H NMR of toluene derived tetrafluorothianthrenium salt **S2**, *ortho*- + *para*-isomer ........ 16

$^{13}$C NMR of toluene derived tetrafluorothianthrenium salt **S2**, *ortho*- + *para*-isomer ........ 17

$^{19}$F NMR of toluene derived tetrafluorothianthrenium salt **S2**, *ortho*- + *para*-isomer ........ 18

$^{1}$H NMR of toluene derived phenoxathiinium salt **S3**, *para*-isomer ........ 19

$^{13}$C NMR of toluene derived phenoxathiinium salt **S3**, *para*-isomer ........ 20

$^{1}$H NMR of toluene derived phenoxathiinium salt **S3**, *meta*-isomer ........ 21

$^{13}$C NMR of toluene derived phenoxathiinium salt **S3**, *meta*-isomer ........ 22

$^{1}$H NMR of toluene derived phenoxathiinium salt **S3**, *ortho*-isomer ........ 23

$^{13}$C NMR of toluene derived phenoxathiinium salt **S3**, *ortho*-isomer ........ 24

$^{1}$H NMR of toluene derived dibenzothiophenium salt **S4**, *para*-isomer ........ 25

$^{13}$C NMR of toluene derived dibenzothiophenium salt salt **S4**, *para*-isomer ........ 26

$^{1}$H NMR of toluene derived dibenzothiophenium salt **S4**, *meta*-isomer ........ 27

$^{13}$C NMR of toluene derived dibenzothiophenium salt **S4**, *meta*-isomer ........ 28

$^{1}$H NMR of toluene derived dibenzothiophenium salt **S4**, *ortho*-isomer ........ 29

$^{13}$C NMR of toluene derived dibenzothiophenium salt **S4**, *ortho*-isomer ........ 30

$^{1}$H NMR of toluene derived diphenylsulfonium salt **S5**, *para*-isomer ........ 31

$^{13}$C NMR of toluene derived diphenylsulfonium salt **S5**, *para*-isomer ........ 32

$^{1}$H NMR of toluene derived diphenylsulfonium salt **S5**, *meta*-isomer ........ 33

$^{13}$C NMR of toluene derived diphenylsulfonium salt **S5**, *meta*-isomer ........ 34

$^{1}$H NMR of toluene derived diphenylsulfonium salt **S5**, *ortho*-isomer ........ 35

$^{13}$C NMR of toluene derived diphenylsulfonium salt **S5**, *ortho*-isomer ........ 36

$^{1}$H NMR of **TTO-$d_2$** ........ 37

$^{13}$C NMR of **TTO-$d_2$** ........ 38

$^{2}$H NMR of **TTO-$d_2$** ........ 39

$^{1}$H NMR of *tert*-butylbenzene derived $d_2$-thianthrenium salt **2-$d_2$** ........ 40

$^{13}$C NMR of *tert*-butylbenzene derived $d_2$-thianthrenium salt **2-$d_2$** ........ 41

$^{2}$H NMR of *tert*-butylbenzene derived $d_2$-thianthrenium salt **2-$d_2$** ........ 42

References ........ 44

# MATERIALS AND METHODS

All air- and moisture-insensitive reactions were carried out under an ambient atmosphere and monitored by thin-layer chromatography (TLC). High-resolution mass spectra were obtained using Q Exactive Plus from Thermo. Concentration under reduced pressure was performed by rotary evaporation at 25–40 °C at an appropriate pressure. Purified compounds were further dried under vacuum ($10^{-6}$ – $10^{-3}$ bar). Yields refer to purified and spectroscopically pure compounds, unless otherwise stated.

### Solvents

Dichloromethane, and methanol were purchased from *Sigma-Aldrich* and used as received. Anhydrous solvents were obtained from *Phoenix Solvent Drying Systems*. All deuterated solvents were purchased from *Euriso-Top*. Anhydrous acetonitrile-$d_3$ was dried by storage over molecular sieves.

### Chromatography

Thin layer chromatography (TLC) was performed using EMD TLC plates pre-coated with 250 µm thickness silica gel 60 $F_{254}$ plates and visualized by fluorescence quenching under UV light and $KMnO_4$ stain. Flash column chromatography was performed using silica gel (40–63 µm particle size) purchased from Geduran®. Preparatory high-performance liquid chromatographic separation was executed on Shimadzu Prominence Preparative HPLC system with an YMC-Triart C18 HPLC column.

### Spectroscopy and Instruments

NMR spectra were recorded on a *Bruker Ascend*™ 500 spectrometer operating at 500 MHz, 471 MHz and 126 MHz, for 1H, 19F and 13C acquisitions, respectively. Chemical shifts are reported in ppm with the solvent residual peak as the internal standard. For $^1$H NMR: $CDCl_3$, δ 7.26; $CD_3CN$, δ 1.96; $CD_2Cl_2$, δ 5.32; For $^{13}$C NMR: CDCl3, δ 77.16; CD3CN, δ 1.32; CD2Cl2, δ 53.84. $^{19}$F NMR spectra were referenced using a unified chemical shift scale based on the $^1$H resonance of tetramethylsilane (1% v/v solution in the respective solvent). Data is reported as follows: s = singlet, d = doublet, t = triplet, q = quartet, quin = quintet, sext = sextet, sept = septet, m = multiplet, bs = broad singlet; coupling constants in Hz; integration.

### Starting materials

All substrates were used as received from commercial suppliers or prepared according to published procedures, unless otherwise stated. Chemicals were purchased from *Sigma-Aldrich*, *Chempur*, *TCI*, or *Alfa Aesar*. Thianthrene-S-oxide (**TTO**), 2,3,7,8-Tetrafluorothianthrene-S-oxide (**TFTO**), dibenzothiophene-S-oxide (**DBTO**), phenoxatiine-S-oxide (**PXTO**) and thianthrenium tetrafluoroborate (**TT**$^{\bullet+}$$BF_4$) were prepared according to the literature.[1]

# EXPERIMENTAL DATA

## Experimental procedures

### General procedure for thianthrenation of arenes with thianthrene-S-oxide/TFAA/$HBF_4OEt_2$

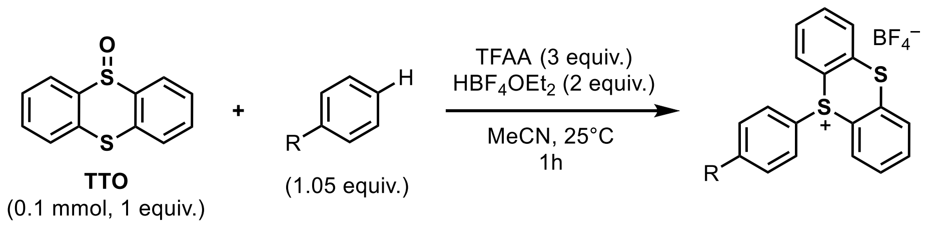


Under an ambient atmosphere, a 4 ml glass-vial equipped with a stir bar was charged with thianthrene-S-oxide (23.2 mg, 0.100 mmol, 1.0 equiv.), dry MeCN (1 mL, c = 0.1 M) and the arene (0.105 mmol, 1.05 equiv.). TFAA (42 µL, 0.30 mmol, 3.0 equiv.) and then $HBF_4{\cdot}OEt_2$ (27 µL, 0.20 mmol, 2.0 equiv.) were subsequently added to the vial while stirring the reaction mixture. The vial was sealed with a screw-cap and the mixture was stirred at 25 °C for 1 h. The solution was diluted with 10 ml DCM and poured onto 10 ml of saturated aqueous $NaHCO_3$ solution in a separatory funnel. After the layers were separated, the DCM layer was washed with aqueous $NaBF_4$ solution (10 ml, 5 % w/w). The organic phase was dried over $Mg_2SO_4$, filtered, and the solvent was removed under reduced pressure. The resultant residue was dissolved in $CD_3CN$ (0.5 mL), internal standard $CH_2Br_2$ (14 µL, 0.20 mmol, 2.0 equiv.) was added and the mixture was analyzed by $^1H$ NMR spectroscopy. Reactions using $CD_3CN$ as solvent were carried out using the above protocol at 0.05 mmol scale and analyzed by $^1H$ NMR spectroscopy without previous aqueous work up.

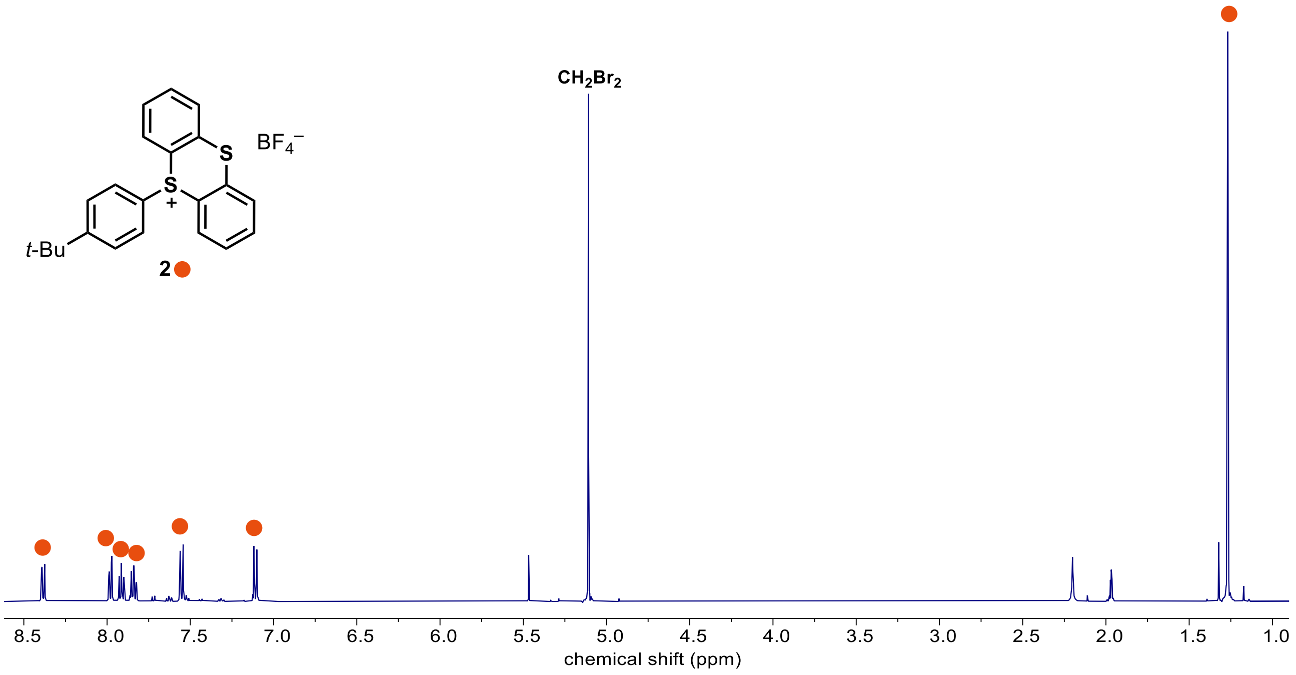


**Figure S1.** Representative $^1H$ NMR spectrum ($CD_3CN$, 500 MHz) of the analyzed reaction mixture.

## General procedure for thianthrenation of arenes with thianthrene-S-oxide/TFAOTf

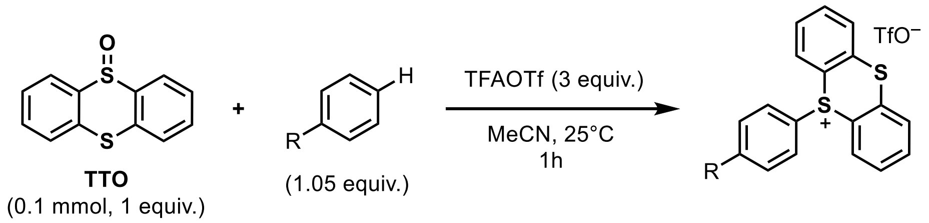


Under an ambient atmosphere, a 4 ml glass-vial equipped with a stir bar was charged with thianthrene-S-oxide (23.2 mg, 0.1 mmol, 1.00 equiv.), dry MeCN (1 mL, c = 0.1 M) and the arene (0.105 mmol, 1.05 equiv.). TFAOTf (74 mg, 0.30 mmol, 3.0 equiv.) was added to the vial while stirring the reaction mixture. The vial was sealed with a screw-cap and the mixture was stirred at 25 °C for 1 h. The solution was diluted with 10 ml DCM and poured onto 10 ml of saturated aqueous $NaHCO_3$ solution in a separatory funnel. The organic phase was dried over $Mg_2SO_4$, filtered, and the solvent was removed under reduced pressure. The resultant residue was dissolved in $CD_3CN$ (0.5 mL), internal standard $CH_2Br_2$ (14 µL, 0.20 mmol, 2.0 equiv.) was added and the mixture was analyzed by $^1H$ NMR spectroscopy. Reactions in $CD_3CN$ were carried out using the above protocol at 0.05 mmol scale and analyzed by $^1H$ NMR spectroscopy without the need for aqueous work up.

## General procedure for thianthrenation of arenes with thianthrenium radical cation tetrafluoroborate

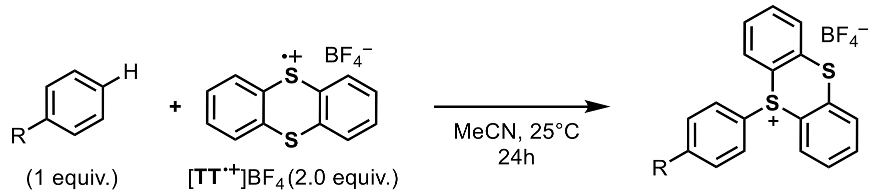


In a nitrogen-filled glove box, a 4 mL vial equipped with a stirbar was charged with thianthrene radical cation tetrafluoroborate (60.6 mg, 0.200 mmol, 2.00 equiv.) and dry MeCN (1 mL, c = 0.1 M). The vial was sealed with a septum-cap, removed from the glove box, and the arene (0.1 mmol, 1.0 equiv.) was added via the septum. The mixture was stirred at 25 °C for 24 h. The solution was diluted with 10 ml DCM and poured onto 10 ml of saturated aqueous $NaHCO_3$ solution in a separatory funnel. The organic phase was dried over $Mg_2SO_4$, filtered, and the solvent was removed under reduced pressure. The resultant residue was dissolved in $CD_3CN$ (0.5 mL), internal standard $CH_2Br_2$ (14 µL, 0.20 mmol, 2.0 equiv.) was added and the mixture was analyzed by $^1H$ NMR spectroscopy.

### General procedure for thianthrenation of arenes with thianthrene-S-oxide in neat $H_2SO_4$

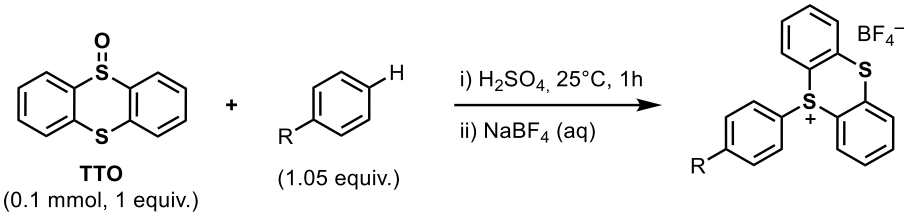


Following a described protocol,[2] under an ambient atmosphere a 4 ml glass-vial equipped with a stir bar was charged with thianthrene-S-oxide (23.2 mg, 0.100 mmol, 1.00 equiv.), $H_2SO_4$ (1 mL, c = 0.1 M) and the arene (0.105 mmol, 1.05 equiv.). The vial was sealed with a screw-cap and the mixture was stirred at 25 °C for 1 h. The solution was then poured onto ice. Once the ice melted, 10 mL of DCM were added and the mixture was transferred to a separatory funnel. The DCM layer was washed with a saturated aqueous $NaHCO_3$ solution (10 mL) and aqueous $NaBF_4$ solution (2x10 ml, 5 % w/w). The organic phase was then dried over $Mg_2SO_4$, filtered, and the solvent was removed under reduced pressure. The resultant residue was dissolved in $CD_3CN$ (0.5 mL), internal standard $CH_2Br_2$ (14 µL, 0.20 mmol, 2.0 equiv.) was added and the mixture was analyzed by $^1H$ NMR spectroscopy.

## Characterization of new sulfonium salts

### *tert*-butylbenzene derived thianthrenium salt 2, *para*-isomer

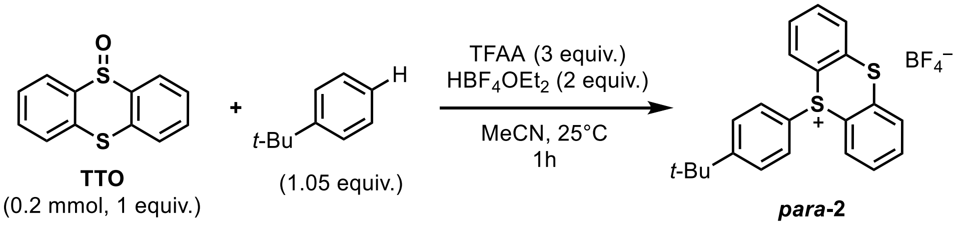


Under an ambient atmosphere, a 4 mL vial equipped with a stir bar was charged with thianthrene-S-oxide (46 mg, 0.20 mmol, 1.0 equiv.), dry MeCN (2 mL, c = 0.1 M) and *tert*-butylbenzene (32 µL, 0.21 mmol, 1.05 equiv.). TFAA (84 µL, 0.30 mmol, 3.0 equiv.) and then $HBF_4{\cdot}OEt_2$ (56 µL, 0.20 mmol, 2.0 equiv.) were subsequently added to the vial while stirring the reaction mixture. The mixture was stirred at 25 °C for 1 h and then diluted with 20 ml DCM and poured onto 20 ml of saturated aqueous $NaHCO_3$ solution in a separatory funnel. After the layers were separated, the DCM layer was washed with aqueous $NaBF_4$ solution (20 ml, 5 % w/w). The organic phase was dried over $Mg_2SO_4$, filtered, and the solvent was removed under reduced pressure. The residue was purified by chromatography on silica gel eluting with DCM/MeOH (20:1, v/v) to afford ***para*-2** (79 mg, 0.18 mmol, 91 %) as a colorless solid.

**NMR Spectroscopy:**

**$^1$H NMR** (500 MHz, $CD_3CN$, 23 °C, δ): 8.39 (dd, *J* = 7.9, 1.4 Hz, 2H), 7.99 (dd, *J* = 7.9, 1.4 Hz, 2H), 7.92 (td, *J* = 7.7, 1.4 Hz, 2H), 7.84 (td, *J* = 7.7, 1.4 Hz, 2H), 7.56 (d, *J* = 8.9 Hz, 2H), 7.11 (d, *J* = 8.9 Hz, 2H), 1.27 (s, 9H).

**$^{13}$C NMR** (125 MHz, $CD_3CN$, 23 ºC, δ): 157.6, 136.9, 135.5, 135.3, 131.0, 130.4, 128.3, 128.2, 120.9, 119.0, 35.3, 30.4.

**$^{19}$F NMR** (470 MHz, $CD_3CN$, 23 ºC, δ): –151.62 (s), –151.68 (s) ppm

**HRMS-ESI(m/z)** calc'd for $C_{22}H_{21}S_2$ $[M–BF_4]^+$, 349.1079; found, 349.1080; deviation: 0.5 ppm.

### indole derived thianthrenium salt S1

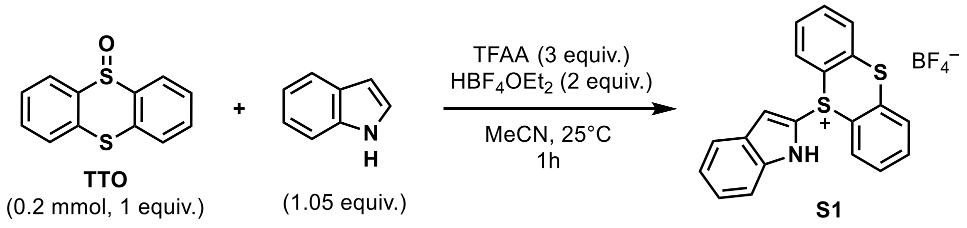


Under an ambient atmosphere, a 4 mL vial equipped with a stir bar was charged with thianthrene-S-oxide (46 mg, 0.20 mmol, 1.0 equiv.), dry MeCN (2 mL, c = 0.1 M) and indole (24 mg, 0.21 mmol, 1.05 equiv.). TFAA (84 μL, 0.30 mmol, 3.0 equiv.) and then $HBF_4·OEt_2$ (56 μL, 0.20 mmol, 2.0 equiv.) were subsequently added to the vial while stirring the reaction mixture. The mixture was stirred at 25 °C for 1 h and then diluted with 20 ml DCM and poured onto 20 ml of saturated aqueous $NaHCO_3$ solution in a separatory funnel. After the layers were separated, the DCM layer was washed with aqueous $NaBF_4$ solution (20 ml, 5 % w/w). The organic phase was dried over $Mg_2SO_4$, filtered, and the solvent was removed under reduced pressure. The residue was purified by chromatography on silica gel eluting with DCM/MeOH (20:1, v/v) to afford **S1** (59 mg, 0.14 mmol, 70 %) as a pale-yellow solid.

**NMR Spectroscopy:**

**$^1$H NMR** (500 MHz, $CD_3CN$, 23 ºC, δ): 11.02 (bs, 1H), 8.47 (d, *J* = 3.4 Hz, 1H), 8.01 (dd, *J* = 7.8, 1.2 Hz, 2H), 7.86 (dt, *J* = 8.4, 0.9 Hz, 1H), 7.75 (td, *J* = 7.7, 1.3 Hz, 2H), 7.52 (ddd, *J* = 8.5, 7.5, 1.2 Hz, 2H), 7.46 (ddd, *J* = 8.3, 7.1, 1.1 Hz, 1H), 7.41 (dd, *J* = 8.2, 1.3 Hz, 2H), 7.15 (ddd, *J* = 8.1, 7.2, 0.9 Hz, 1H), 6.90 (d, *J* = 8.1 Hz, 1H).

**$^{13}$C NMR** (75 MHz, $CD_3CN$, 23 ºC, δ): 141.0, 139.3, 134.0, 133.3, 131.5, 130.6, 129.4, 126.0, 125.8, 124.1, 123.5, 120.0, 115.6, 83.5.

**$^{19}$F NMR** (470 MHz, $CD_3CN$, 23 ºC, δ): –151.67 (bs), –151.73 (bs) ppm

**HRMS-ESI(m/z)** calc'd for $C_{20}H_{14}N_1S_2$ $[M–BF_4]^+$, 332.0562; found, 332.0563; deviation: 0.3 ppm.

## Preparation of *ortho*-, *meta*- and *para*-isomers of tolyl sulfonium salts

### Toluene derived thianthrenium salt 5, *para*-isomer

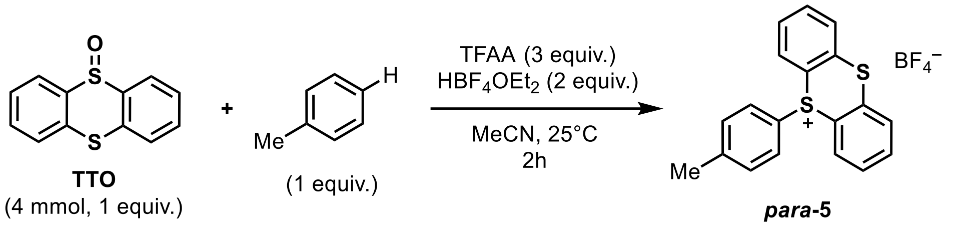


Under an ambient atmosphere, a 100mL round bottom flask equipped with a stir bar was charged with thianthrene-S-oxide (929 mg, 4.00 mmol, 1.00 equiv.), dry MeCN (40 mL, c = 0.10 M) and toluene (0.43 mL, 4.00 mmol, 1.00 equiv.). TFAA (1.67 mL, 12.0 mmol, 3.00 equiv.) and then $HBF_4·OEt_2$ (1.08 mL, 8.00 mmol, 2.00 equiv.) were subsequently added to the vial while stirring the reaction mixture. The flask was capped and the mixture was stirred at 25 °C for 2 h. The solution was diluted with 100 ml DCM and poured onto 100 ml of saturated aqueous $NaHCO_3$ solution in a separatory funnel. After the layers were separated, the DCM layer was washed with aqueous $NaBF_4$ solution (100 ml, 5 % w/w). The organic phase was dried over $Mg_2SO_4$, filtered, and the solvent was removed under reduced pressure. The residue was purified by chromatography on silica gel eluting with DCM/*i*-PrOH (20:1, v/v) to afford ***para*-5** (1.11 g, 2.80 mmol, 70 %) as a colorless solid.

**NMR Spectroscopy:**

**$^1$H NMR** (500 MHz, $CD_3CN$, 23 ºC, δ): 8.37 (dd, *J* = 7.9, 1.4 Hz, 2H), 7.98 (dd, *J* = 7.9, 1.4 Hz, 2H), 7.91 (td, *J* = 7.7, 1.4 Hz, 2H), 7.84 (td, *J* = 7.7, 1.4 Hz, 2H), 7.33 (d, *J* = 8.7 Hz, 2H), 7.05 (d, *J* = 8.7 Hz, 2H), 2.37 (s, 3H).

**$^{13}$C NMR** (125 MHz, $CD_3CN$, 23 ºC, δ): 145.1, 137.0, 135.6, 135.4, 131.8, 131.2, 130.5, 128.5, 120.9, 119.3, 20.9.

**$^{19}$F NMR** (470 MHz, $CD_3CN$, 23 ºC, δ): –151.26 (bs), –151.31 (bs).

**HRMS-ESI(m/z)** calc'd for $C_{19}H_{15}S_2$ $[M–BF_4]^+$, 307.0610; found, 307.0607; deviation: 0.9 ppm.

### Toluene derived thianthrenium salt 5, *meta*-isomer

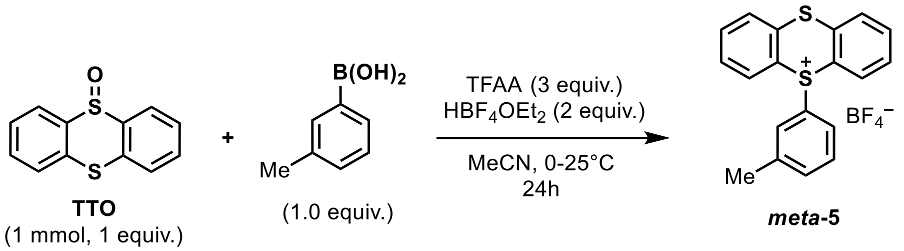


Under an ambient atmosphere, a 50mL round bottom flask equipped with a stir bar was charged with thianthrene-S-oxide (232 mg, 1.00 mmol, 1.00 equiv.), dry MeCN (10 mL, c = 0.10 M) and 3-methylboronic

acid (136 mg, 1.00 mmol, 1.00 equiv.) and the mixture was cooled down to 0°C. TFAA (420 µL, 3.00 mmol, 3.00 equiv.) and then $HBF_4 \cdot OEt_2$ (270 µL, 2.00 mmol, 2.00 equiv.) were subsequently added to the vial while stirring the reaction mixture. The flask was capped and the mixture was stirred at 0 °C for 1 h, then at 25 °C for 24 h. The solution was diluted with 60 ml DCM and poured onto 60 ml of saturated aqueous $NaHCO_3$ solution in a separatory funnel. After the layers were separated, the DCM layer was washed with aqueous $NaBF_4$ solution (50 ml, 5 % w/w). The organic phase was dried over $Mg_2SO_4$, filtered, and the solvent was removed under reduced pressure. The residue was purified by chromatography on silica gel eluting with DCM/*i*-PrOH (20:1, v/v) to afford ***meta*-5** (374 mg, 3.80 mmol, 95 %) as a colorless solid.

**NMR Spectroscopy:**

**$^1$H NMR** (500 MHz, $CD_3CN$, 23 ºC, δ): 8.40 (dd, *J* = 7.9, 1.4 Hz, 2H), 7.96 (dd, *J* = 8.0, 1.4 Hz, 2H), 7.91 (td, *J* = 7.7, 1.4 Hz, 2H), 7.84 (td, *J* = 7.7, 1.6 Hz, 2H), 7.43 (d, *J* = 7.7 Hz, 1H), 7.37 (t, *J* = 7.9 Hz, 1H), 7.00 (s, 1H), 6.92 (d, *J* = 8.0 Hz, 1H), 2.28 (s, 3H).

**$^{13}$C NMR** (125 MHz, $CD_3CN$, 23 ºC, δ): 142.2, 137.2, 135.7, 135.6, 134.4, 131.2, 130.9, 130.5, 128.5, 125.6, 124.2, 119.1, 20.9

**$^{19}$F NMR** (470 MHz, $CD_3CN$, 23 ºC, δ): –151.26 (bs), –151.31 (bs).

**HRMS-ESI(m/z)** calc'd for $C_{19}H_{15}S_2$ $[M–BF_4]^+$, 307.0610; found, 307.0606; deviation: 1.2 ppm.

## Toluene derived thianthrenium salt 5, *ortho*-isomer

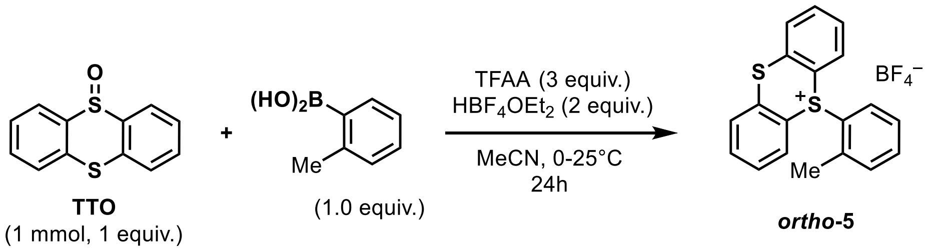


***ortho*-5**

Under an ambient atmosphere, a 50mL round bottom flask equipped with a stir bar was charged with thianthrene-S-oxide (232 mg, 1.00 mmol, 1.00 equiv.), dry MeCN (10 mL, c = 0.10 M) and 2-methylboronic acid (136 mg, 1.00 mmol, 1.00 equiv.) and the mixture was cooled down to 0°C. TFAA (420 µL, 3.00 mmol, 3.00 equiv.) and then $HBF_4 \cdot OEt_2$ (270 µL, 2.00 mmol, 2.00 equiv.) were subsequently added to the vial while stirring the reaction mixture. The flask was capped and the mixture was stirred at 0 °C for 1 h, then at 25 °C for 24 h. The solution was diluted with 60 ml DCM and poured onto 60 ml of saturated aqueous $NaHCO_3$ solution in a separatory funnel. After the layers were separated, the DCM layer was washed with aqueous $NaBF_4$ solution (50 ml, 5 % w/w). The organic phase was dried over $Mg_2SO_4$, filtered, and the solvent was removed under reduced pressure. The residue was purified by chromatography on silica gel eluting with DCM/*i*-PrOH (20:1, v/v) to afford ***ortho*-5** (267 mg, 0.680 mmol, 68 %) as a colorless solid.

**NMR Spectroscopy:**

**$^1$H NMR** (500 MHz, $CD_3CN$, 23 ºC, δ): 8.24 (dd, *J* = 8.0, 1.4 Hz, 2H), 7.98 (dd, *J* = 7.9, 1.4 Hz, 2H), 7.86

(td, $J$ = 7.8, 1.4 Hz, 2H), 7.78 (td, $J$ = 7.5, 1.4 Hz, 2H), 7.59 (td, $J$ = 7.5, 1.2 Hz, 1H), 7.50 (d, $J$ = 7.4 Hz, 1H), 7.31 (t, $J$ = 8.0 Hz, 1H), 7.02 (dd, $J$ = 8.3, 1.2 Hz, 1H), 2.66 (s, 3H).

**$^{13}$C NMR** (125 MHz, $CD_3CN$, 23 ºC, δ): 140.9, 137.6, 135.3, 134.8, 134.7, 134.3, 131.5, 130.6, 130.2, 128.4, 121.8, 118.6, 20.4.

**$^{19}$F NMR** (470 MHz, $CD_3CN$, 23 ºC, δ): –151.27 (bs), –151.32 (bs).

**HRMS-ESI(m/z)** calc'd for $C_{19}H_{15}S_2$ $[M–BF_4]^+$, 307.0610; found, 307.0605; deviation: 1.4 ppm.

## Toluene derived tetrafluorothianthrenium salt S2, *para*-isomer

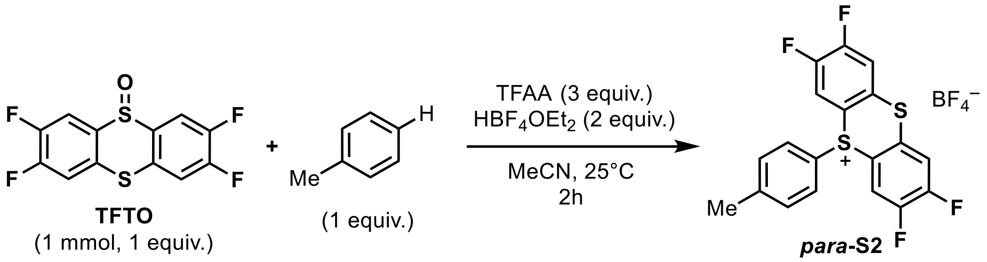


Under an ambient atmosphere, a 50mL round bottom flask equipped with a stir bar was charged with tetrafluorothianthrene-S-oxide (304 mg, 1.00 mmol, 1.00 equiv.), dry MeCN (10 mL, c = 0.10 M) and toluene (106 µL, 1.00 mmol, 1.00 equiv.). TFAA (420 µL, 3.00 mmol, 3.00 equiv.) and then $HBF_4·OEt_2$ (270 µL, 2.00 mmol, 2.00 equiv.) were subsequently added to the vial while stirring the reaction mixture. The flask was capped and the mixture was stirred at 25 °C for 2 h. The solution was diluted with 60 ml DCM and poured onto 60 ml of saturated aqueous $NaHCO_3$ solution in a separatory funnel. After the layers were separated, the DCM layer was washed with aqueous $NaBF_4$ solution (50 ml, 5 % w/w). The organic phase was dried over $Mg_2SO_4$, filtered, and the solvent was removed under reduced pressure. The residue was purified by chromatography on silica gel eluting with DCM/*i*-PrOH (20:1, v/v) to afford ***para*-S2** (437 mg, 0.940 mmol, 94 %) as a colorless solid.

**NMR Spectroscopy:**

**$^{1}$H NMR** (500 MHz, $CD_3CN$, 23 ºC, δ): 8.45 (dd, $J$ = 9.1, 7.2 Hz, 2H), 7.98 (dd, $J$ = 10.0, 7.1 Hz, 2H), 7.37 (d, $J$ = 8.7 Hz, 2H), 7.16 (d, $J$ = 8.5 Hz, 2H), 2.39 (s, 3H).

**$^{13}$C NMR** (125 MHz, $CD_3CN$, 23 ºC, δ): 154.53 (dd, $J$ = 261.6, 13.2 Hz), 151.32 (dd, $J$ = 255.5, 13.5 Hz), 145.80, 134.91 (dd, $J$ = 8.5, 4.0 Hz), 132.01, 128.99, 125.26 (dd, $J$ = 22.1, 2.4 Hz), 120.89 (d, $J$ = 22.0 Hz), 119.72, 115.28 (dd, $J$ = 7.2, 3.6 Hz), 21.08.

**$^{19}$F NMR** (470 MHz, $CD_3CN$, 23 ºC, δ): –125.59 (ddd, $J$ = 20.7, 10.3, 7.5 Hz), –133.88 (ddd, $J$ = 20.2, 9.2, 7.3 Hz), –150.90 (bs), –150.95 (bs).

**HRMS-ESI(m/z)** calc'd for $C_{19}H_{11}F_4S_2$ $[M–BF_4]^+$, 379.0233; found, 379.0227; deviation: 1.6 ppm.

### Toluene derived tetrafluorothianthrenium salt S2, *meta*-isomer

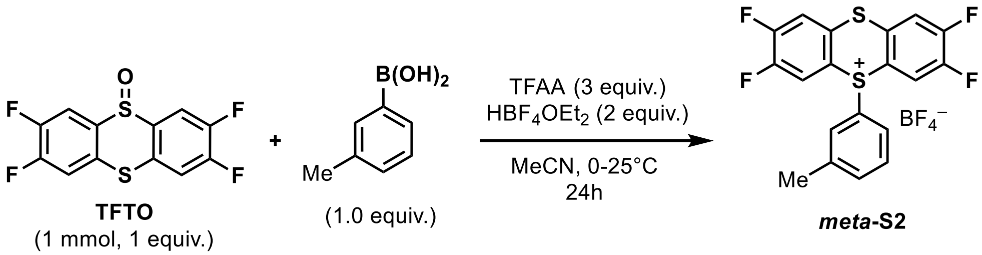


Under an ambient atmosphere, a 50mL round bottom flask equipped with a stir bar was charged with tetrafluorothianthrene-S-oxide (304 mg, 1.00 mmol, 1.00 equiv.), dry MeCN (10 mL, c = 0.10 M) and 3-methylboronic acid (136 mg, 1.00 mmol, 1.00 equiv.) and the mixture was cooled down to 0°C. TFAA (420 µL, 3.00 mmol, 3.00 equiv.) and then $HBF_4·OEt_2$ (270 µL, 2.00 mmol, 2.00 equiv.) were subsequently added to the vial while stirring the reaction mixture. The flask was capped and the mixture was stirred at 0 °C for 1 h, then at 25 °C for 24 h. The solution was diluted with 60 ml DCM and poured onto 60 ml of saturated aqueous $NaHCO_3$ solution in a separatory funnel. After the layers were separated, the DCM layer was washed with aqueous $NaBF_4$ solution (50 ml, 5 % w/w). The organic phase was dried over $Mg_2SO_4$, filtered, and the solvent was removed under reduced pressure. The residue was purified by chromatography on silica gel eluting with DCM/*i*-PrOH (20:1, v/v) to afford ***meta*-S2** (338 mg, 0.720 mmol, 72 %) as a colorless solid.

**NMR Spectroscopy:**

**$^{1}$H NMR** (500 MHz, $CD_3CN$, 23 ºC, δ): 8.50 – 8.40 (m, 2H), 8.05 – 7.91 (m, 2H), 7.49 (d, *J* = 7.7 Hz, 1H), 7.41 (t, *J* = 7.9 Hz, 1H), 7.07 (s, 1H), 7.02 (d, *J* = 8.2 Hz, 1H), 2.32 (s, 3H).

**$^{13}$C NMR** (125 MHz, $CD_3CN$, 23 ºC, δ): 154.4 (dd, *J* = 261.6, 13.1 Hz), 151.2 (dd, *J* = 255.6, 13.8 Hz), 142.4, 134.9 (dd, *J* = 8.5, 4.2 Hz), 134.8, 130.9, 128.7, 125.9, 125.3 (dd, *J* = 22.3, 2.4 Hz), 122.9, 120.8 (d, *J* = 21.9 Hz), 114.9 (dd, *J* = 7.4, 3.6 Hz), 20.8.

**$^{19}$F NMR** (470 MHz, $CD_3CN$, 23 ºC, δ): –125.49 (dt, *J* = 18.8, 8.8 Hz), –133.84 (dt, *J* = 20.5, 8.4 Hz),–150.91 (bs), –150.96 (bs).

**HRMS-ESI(m/z)** calc'd for $C_{19}H_{11}F_4S_2$ $[M–BF_4]^+$, 379.0233; found, 379.0229; deviation: 1.1 ppm.

### Toluene derived tetrafluorothianthrenium salt S2, *ortho*-isomer

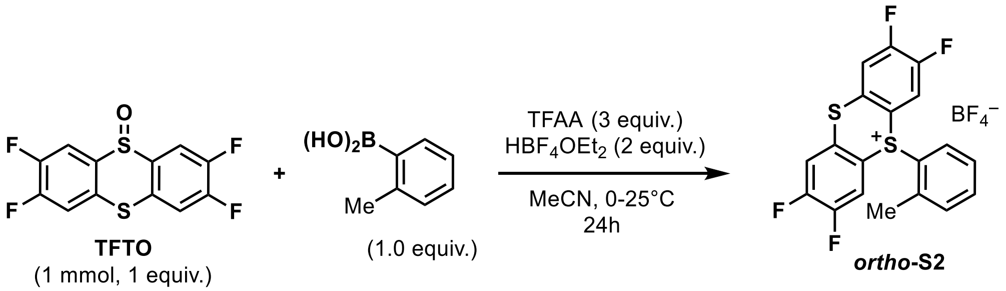


Under an ambient atmosphere, a 50mL round bottom flask equipped with a stir bar was charged with

tetrafluorothianthrene-S-oxide (304 mg, 1.00 mmol, 1.00 equiv.), dry MeCN (10 mL, c = 0.10 M) and 2-methylboronic acid (136 mg, 1.00 mmol, 1.00 equiv.) and the mixture was cooled down to 0°C. TFAA (420 µL, 3.00 mmol, 3.00 equiv.) and then $HBF_4{\cdot}OEt_2$ (270 µL, 2.00 mmol, 2.00 equiv.) were subsequently added to the vial while stirring the reaction mixture. The flask was capped and the mixture was stirred at 0 °C for 1 h, then at 25 °C for 24 h. The solution was diluted with 60 ml DCM and poured onto 60 ml of saturated aqueous $NaHCO_3$ solution in a separatory funnel. After the layers were separated, the DCM layer was washed with aqueous $NaBF_4$ solution (50 ml, 5 % w/w). The organic phase was dried over $Mg_2SO_4$, filtered, and the solvent was removed under reduced pressure. The residue was purified by chromatography on silica gel eluting with DCM/*i*-PrOH (20:1, v/v) to afford ***ortho*-S2** (192 mg, 410 µmol, 41 %) as a colorless solid.

**NMR Spectroscopy:**

**$^1$H NMR** (500 MHz, $CD_3CN$, 23 ºC, δ): 8.33 (dd, *J* = 9.2, 7.2 Hz, 2H), 8.02 (dd, *J* = 9.9, 7.1 Hz, 2H), 7.65 (td, *J* = 7.5, 1.2 Hz, 1H), 7.55 (d, *J* = 7.2 Hz, 1H), 7.40 – 7.34 (m, 1H), 7.10 (dd, *J* = 8.4, 1.2 Hz, 1H), 2.69 (s, 3H).

**$^{13}$C NMR** (125 MHz, $CD_3CN$, 23 ºC, δ): 154.1 (dd, *J* = 261.7, 13.3 Hz), 151.3 (dd, *J* = 255.6, 13.8 Hz), 141.2, 135.6 (dd, *J* = 8.6, 3.9 Hz), 135.2, 134.5, 130.4, 128.5, 124.4 (dd, *J* = 22.1, 2.4 Hz), 121.0 (d, *J* = 21.8 Hz), 120.4, 114.5 (dd, *J* = 7.1, 3.5 Hz), 20.5.

**$^{19}$F NMR** (470 MHz, $CD_3CN$, 23 ºC, δ): –125.85 (ddd, *J* = 20.2, 9.6, 7.0 Hz), –133.55 (ddd, *J* = 20.1, 9.2, 7.2 Hz), –150.57 (bs), –150.62 (bs).

**HRMS-ESI(m/z)** calc'd for $C_{19}H_{11}F_4S_2$ $[M–BF_4]^+$, 379.0233; found, 379.0233; deviation: 0.0 ppm.

### Toluene derived phenoxathiinium salt S3, *para*-isomer

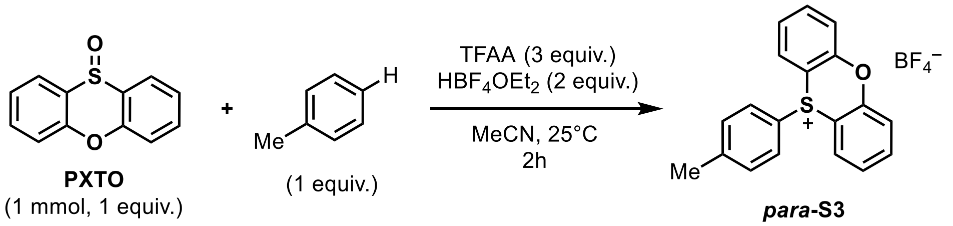


Under an ambient atmosphere, a 50mL round bottom flask equipped with a stir bar was charged with phenoxathiine-S-oxide (216 mg, 1.00 mmol, 1.00 equiv.), dry MeCN (10 mL, c = 0.10 M) and toluene (106 µL, 1.00 mmol, 1.00 equiv.). TFAA (420 µL, 3.00 mmol, 3.00 equiv.) and then $HBF_4{\cdot}OEt_2$ (270 µL, 2.00 mmol, 2.00 equiv.) were subsequently added to the vial while stirring the reaction mixture. The flask was capped and the mixture was stirred at 25 °C for 2 h. The solution was diluted with 60 ml DCM and poured onto 60 ml of saturated aqueous $NaHCO_3$ solution in a separatory funnel. After the layers were separated, the DCM layer was washed with aqueous $NaBF_4$ solution (50 ml, 5 % w/w). The organic phase was dried over $Mg_2SO_4$, filtered, and the solvent was removed under reduced pressure. The residue was purified by chromatography on silica gel eluting with DCM/*i*-PrOH (20:1, v/v) to afford ***para*-S3** (270 mg, 0.710 mmol, 71 %) as a colorless solid.

**NMR Spectroscopy:**

**$^{1}$H NMR** (500 MHz, $CD_3CN$, 23 ºC, δ): 8.02 (dd, *J* = 8.1, 1.6 Hz, 2H), 7.91 (ddd, *J* = 8.7, 7.3, 1.6 Hz, 2H), 7.68 (dd, *J* = 8.5, 1.2 Hz, 2H), 7.60 – 7.54 (m, 4H), 7.42 (d, *J* = 8.3 Hz, 2H), 2.38 (s, 3H).

**$^{13}$C NMR** (125 MHz, $CD_3CN$, 23 ºC, δ): 151.9, 147.1, 137.4, 132.7, 131.8, 129.5, 128.6, 127.9, 121.1, 106.3, 21.1.

**$^{19}$F NMR** (470 MHz, $CD_3CN$, 23 ºC, δ): –151.46 (bs), –151.51 (bs).

**HRMS-ESI(m/z)** calc'd for $C_{19}H_{15}OS$ $[M–BF_4]^+$, 291.0838; found, 291.0835; deviation: 0.9 ppm.

## Toluene derived phenoxathiinium salt S3, *meta*-isomer

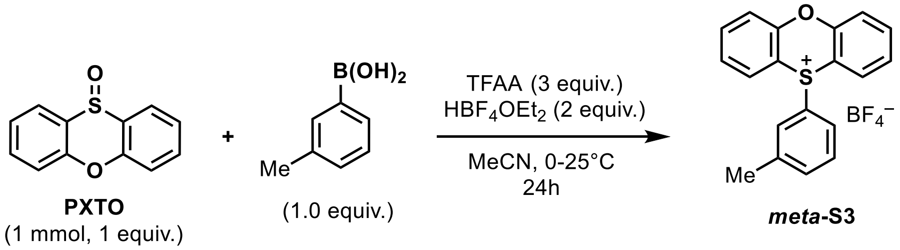


Under an ambient atmosphere, a 50mL round bottom flask equipped with a stir bar was charged with phenoxathiine-S-oxide (216 mg, 1.00 mmol, 1.00 equiv.), dry MeCN (10 mL, c = 0.10 M) and 3-methylboronic acid (136 mg, 1.00 mmol, 1.00 equiv.). TFAA (420 µL, 3.00 mmol, 3.00 equiv.) and then $HBF_4{\cdot}OEt_2$ (270 µL, 2.00 mmol, 2.00 equiv.) were subsequently added to the vial while stirring the reaction mixture. The flask was capped and the mixture was stirred at 25 °C for 2 h. The solution was diluted with 60 ml DCM and poured onto 60 ml of saturated aqueous $NaHCO_3$ solution in a separatory funnel. After the layers were separated, the DCM layer was washed with aqueous $NaBF_4$ solution (50 ml, 5 % w/w). The organic phase was dried over $Mg_2SO_4$, filtered, and the solvent was removed under reduced pressure. The residue was purified by chromatography on silica gel eluting with DCM/*i*-PrOH (20:1, v/v) to afford ***meta*-S3** (120 mg, 0.320 mmol, 32 %) as a colorless solid.

**NMR Spectroscopy:**

**$^{1}$H NMR** (500 MHz, $CD_3CN$, 23 ºC, δ): 8.05 (dd, *J* = 8.1, 1.6 Hz, 2H), 7.92 (ddd, *J* = 8.8, 7.4, 1.6 Hz, 2H), 7.68 (dd, *J* = 8.5, 1.2 Hz, 2H), 7.58 (ddd, *J* = 8.3, 7.4, 1.2 Hz, 2H), 7.52 – 7.41 (m, 4H), 2.34 (s, 3H).

**$^{13}$C NMR** (125 MHz, $CD_3CN$, 23 ºC, δ): 151.8, 143.0, 137.3, 135.8, 131.8, 131.8, 131.6, 129.3, 127.7, 126.3, 120.9, 105.8, 20.7.

**$^{19}$F NMR** (470 MHz, $CD_3CN$, 23 ºC, δ): –151.06 (bs), –151.11 (bs).

**HRMS-ESI(m/z)** calc'd for $C_{19}H_{15}OS$ $[M–BF_4]^+$, 291.0838; found, 291.0835; deviation: 1.2 ppm.

### Toluene derived phenoxathiinium salt S3, *ortho*-isomer

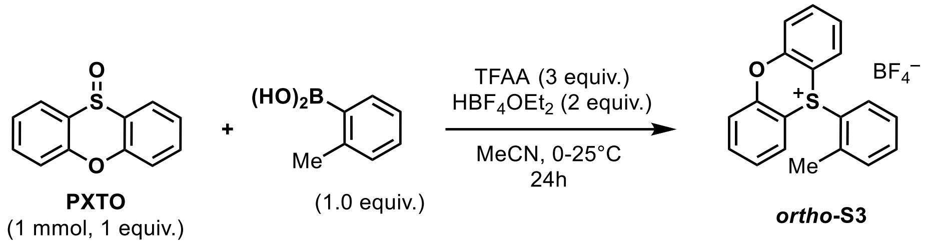


Under an ambient atmosphere, a 50mL round bottom flask equipped with a stir bar was charged with phenoxathiine-S-oxide (216 mg, 1.00 mmol, 1.00 equiv.), dry MeCN (10 mL, c = 0.10 M) and 2-methylboronic acid (136 mg, 1.00 mmol, 1.00 equiv.). TFAA (420 µL, 3.00 mmol, 3.00 equiv.) and then $HBF_4 \cdot OEt_2$ (270 µL, 2.00 mmol, 2.00 equiv.) were subsequently added to the vial while stirring the reaction mixture. The flask was capped and the mixture was stirred at 25 °C for 2 h. The solution was diluted with 60 ml DCM and poured onto 60 ml of saturated aqueous $NaHCO_3$ solution in a separatory funnel. After the layers were separated, the DCM layer was washed with aqueous $NaBF_4$ solution (50 ml, 5 % w/w). The organic phase was dried over $Mg_2SO_4$, filtered, and the solvent was removed under reduced pressure. The residue was purified by chromatography on silica gel eluting with DCM/*i*-PrOH (20:1, v/v) to afford ***ortho*-S3** (177 mg, 0.470 mmol, 47 %) as a colorless solid.

**NMR Spectroscopy:**

$^1$**H NMR** (500 MHz, $CD_3CN$, 23 ºC, δ): 7.97 (dd, *J* = 8.1, 1.5 Hz, 2H), 7.92 (ddd, *J* = 8.8, 7.4, 1.6 Hz, 2H), 7.71 (dd, *J* = 8.5, 1.3 Hz, 2H), 7.60 – 7.52 (m, 4H), 7.36 (dddd, *J* = 8.1, 7.2, 1.6, 0.7 Hz, 1H), 7.20 (dd, *J* = 8.4, 1.1 Hz, 1H), 2.94 (s, 3H).

$^{13}$**C NMR** (125 MHz, $CD_3CN$, 23 ºC, δ): 152.4, 140.8, 137.4, 135.2, 133.6, 131.7, 130.7, 130.3, 129.9, 127.9, 121.2, 105.8, 19.9.

$^{19}$**F NMR** (470 MHz, $CD_3CN$, 23 ºC, δ): –151.54 (bs), –151.59 (bs).

**HRMS-ESI(m/z)** calc'd for $C_{19}H_{15}OS$ $[M–BF_4]^+$, 291.0838; found, 291.0835; deviation: 1.1 ppm.

### Toluene derived dibenzothiophenium salt S4, *para*-isomer

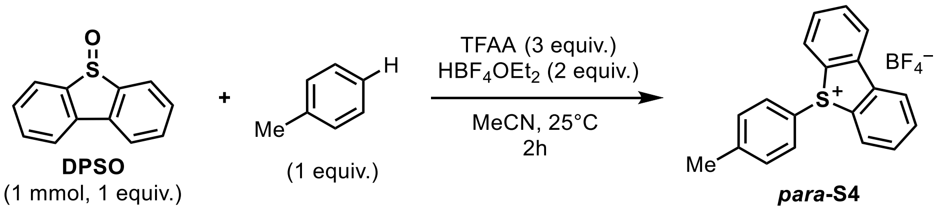


Under an ambient atmosphere, a 50mL round bottom flask equipped with a stir bar was charged with dibenzothiophene-S-oxide (200 mg, 1.00 mmol, 1.00 equiv.), dry MeCN (10 mL, c = 0.10 M) and toluene (106 µL, 1.00 mmol, 1.00 equiv.). TFAA (420 µL, 3.00 mmol, 3.00 equiv.) and then $HBF_4 \cdot OEt_2$ (270 µL, 2.00 mmol, 2.00 equiv.) were subsequently added to the vial while stirring the reaction mixture. The flask was capped

and the mixture was stirred at 25 °C for 2 h. The solution was diluted with 60 ml DCM and poured onto 60 ml of saturated aqueous $NaHCO_3$ solution in a separatory funnel. After the layers were separated, the DCM layer was washed with aqueous $NaBF_4$ solution (50 ml, 5 % w/w). The organic phase was dried over $Mg_2SO_4$, filtered, and the solvent was removed under reduced pressure. The residue was purified by chromatography on silica gel eluting with DCM/*i*-PrOH (20:1, v/v) to afford ***para*-S4** (248 mg, 0.680 mmol, 68 %) as a colorless solid.

**NMR Spectroscopy:**

**$^1$H NMR** (500 MHz, $CD_3CN$, 23 ºC, δ): 8.36 (dd, *J* = 7.9, 1.2 Hz, 2H), 8.08 (dd, *J* = 8.0, 0.9 Hz, 2H), 7.96 (td, *J* = 7.6, 1.0 Hz, 2H), 7.73 (td, *J* = 7.8, 1.2 Hz, 2H), 7.48 (d, *J* = 8.4 Hz, 2H), 7.42 (d, *J* = 8.4 Hz, 2H), 2.40 (s, 3H).

**$^{13}$C NMR** (125 MHz, $CD_3CN$, 23 ºC, δ): 147.6, 139.9, 135.0, 132.8, 132.8, 132.2, 131.0, 128.4, 125.1, 123.5, 21.2.

**$^{19}$F NMR** (470 MHz, $CD_3CN$, 23 ºC, δ): –151.11 (bs), –151.16 (bs).

**HRMS-ESI(m/z)** calc'd for $C_{19}H_{15}S$ $[M–BF_4]^+$, 275.0889; found, 275.0885; deviation: 1.6 ppm.

## Toluene derived dibenzothiophenium salt S4, *meta*-isomer

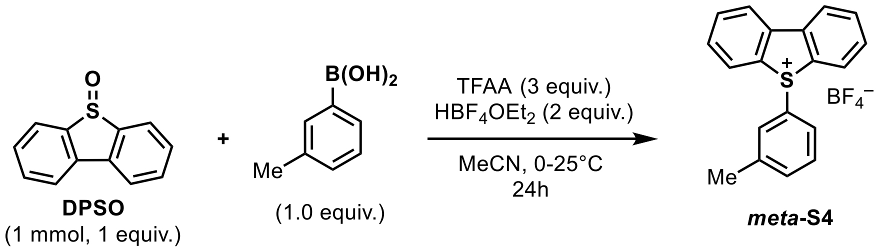


Under an ambient atmosphere, a 50mL round bottom flask equipped with a stir bar was charged with dibenzothiophene-S-oxide (200 mg, 1.00 mmol, 1.00 equiv.), dry MeCN (10 mL, c = 0.10 M) and 3-methylboronic acid (136 mg, 1.00 mmol, 1.00 equiv.) and the mixture was cooled down to 0°C. TFAA (420 µL, 3.00 mmol, 3.00 equiv.) and then $HBF_4·OEt_2$ (270 µL, 2.00 mmol, 2.00 equiv.) were subsequently added to the vial while stirring the reaction mixture. The flask was capped and the mixture was stirred at 0 °C for 1 h, then at 25 °C for 24 h. The solution was diluted with 60 ml DCM and poured onto 60 ml of saturated aqueous $NaHCO_3$ solution in a separatory funnel. After the layers were separated, the DCM layer was washed with aqueous $NaBF_4$ solution (50 ml, 5 % w/w). The organic phase was dried over $Mg_2SO_4$, filtered, and the solvent was removed under reduced pressure. The residue was purified by chromatography on silica gel eluting with DCM/*i*-PrOH (20:1, v/v) to afford ***meta*-S4** (162 mg, 0.450 mmol, 45 %) as a colorless solid.

**NMR Spectroscopy:**

**$^1$H NMR** (500 MHz, $CD_3CN$, 23 ºC, δ): 8.09 (d, *J* = 8.1 Hz, 2H), 7.97 (t, *J* = 7.7 Hz, 2H), 7.75 (t, *J* = 7.8 Hz, 2H), 7.58 (d, *J* = 7.6 Hz, 1H), 7.51 (t, *J* = 7.8 Hz, 1H), 7.46 (d, *J* = 8.1 Hz, 1H), 7.37 (s, 1H), 2.33 (s, 3H).

**$^{13}$C NMR** (125 MHz, $CD_3CN$, 23 ºC, δ): 143.2, 140.0, 136.5, 135.0, 132.4, 132.2, 131.9, 130.7, 128.4, 128.3, 126.9, 125.2, 20.8.

**$^{19}$F NMR** (470 MHz, $CD_3CN$, 23 ºC, δ): –151.33 (bs), –151.38 (bs).

**HRMS-ESI(m/z)** calc'd for $C_{19}H_{15}S$ $[M–BF_4]^+$, 275.0889; found, 275.0888; deviation: 0.4 ppm.

### Toluene derived dibenzothiophenium salt S4, *ortho*-isomer

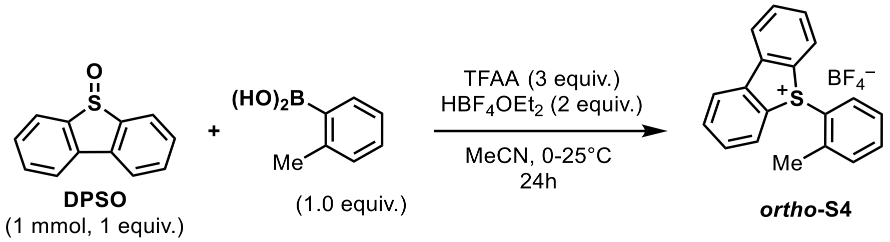


Under an ambient atmosphere, a 50mL round bottom flask equipped with a stir bar was charged with dibenzothiophene-S-oxide (200 mg, 1.00 mmol, 1.00 equiv.), dry MeCN (10 mL, c = 0.10 M) and 2-methylboronic acid (136 mg, 1.00 mmol, 1.00 equiv.) and the mixture was cooled down to 0°C. TFAA (420 μL, 3.00 mmol, 3.00 equiv.) and then $HBF_4{\cdot}OEt_2$ (270 μL, 2.00 mmol, 2.,0 equiv.) were subsequently added to the vial while stirring the reaction mixture. The flask was capped and the mixture was stirred at 0 °C for 1 h, then at 25 °C for 24 h. The solution was diluted with 60 ml DCM and poured onto 60 ml of saturated aqueous $NaHCO_3$ solution in a separatory funnel. After the layers were separated, the DCM layer was washed with aqueous $NaBF_4$ solution (50 ml, 5 % w/w). The organic phase was dried over $Mg_2SO_4$, filtered, and the solvent was removed under reduced pressure. The residue was purified by chromatography on silica gel eluting with DCM/*i*-PrOH (20:1, v/v) to afford ***ortho*-S4** (296 mg, 0.820 mmol, 82 %) as a colorless solid.

**NMR Spectroscopy:**

**$^1$H NMR** (500 MHz, $CD_3CN$, 23 ºC, δ): 8.39 (d, *J* = 7.9 Hz, 2H), 8.08 (d, *J* = 8.0 Hz, 2H), 7.98 (t, *J* = 7.6 Hz, 2H), 7.76 (t, *J* = 7.7 Hz, 2H), 7.67 – 7.60 (m, 2H), 7.28 – 7.22 (m, 1H), 6.74 (d, *J* = 8.2 Hz, 1H), 2.97 (s, 3H).

**$^{13}$C NMR** (125 MHz, $CD_3CN$, 23 ºC, δ): 143.6, 140.1, 135.6, 135.0, 133.7, 132.3, 132.2, 130.0, 128.8, 128.4, 126.3, 125.3, 20.0.

**$^{19}$F NMR** (470 MHz, $CD_3CN$, 23 ºC, δ): –151.61 (bs), –151.66 (bs).

**HRMS-ESI(m/z)** calc'd for $C_{19}H_{15}S$ $[M–BF_4]^+$, 275.0889; found, 275.0887; deviation: 0.9 ppm.

### Toluene derived diphenylsulfonium salt S5, *para*- and *ortho*-isomers

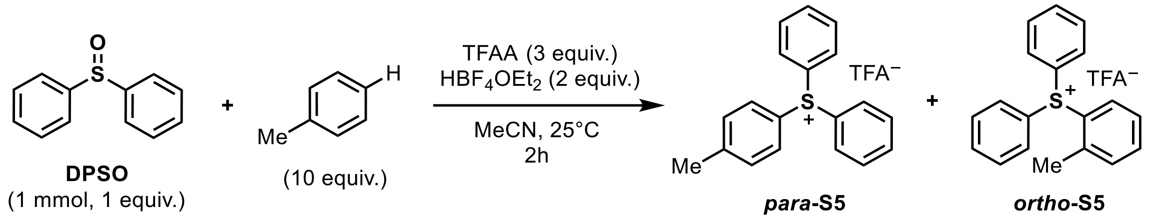


Under an ambient atmosphere, a 50mL round bottom flask equipped with a stir bar was charged with diphenylsulfoxide (202 mg, 1.00 mmol, 1.00 equiv.), dry MeCN (10 mL, c = 0.10 M) and toluene (1.06 mL, 10.0 mmol, 10.0 equiv.). TFAA (420 µL, 3.00 mmol, 3.00 equiv.) and then $HBF_4{\cdot}OEt_2$ (300 µL, 2.00 mmol, 2.00 equiv.) were subsequently added to the vial while stirring the reaction mixture. The flask was capped and the mixture was stirred at 25 °C for 2 h. The solution was diluted with 60 ml DCM and poured onto 60 ml of saturated aqueous $NaHCO_3$ solution in a separatory funnel. After the layers were separated, the DCM layer was washed with aqueous $NaBF_4$ solution (50 ml, 5 % w/w). The organic phase was dried over $Mg_2SO_4$, filtered, and the solvent was removed under reduced pressure. The residue was purified by preparatory high-performance liquid chromatography on C18-reversed phase with a solvent mixture of methanol/water (1% TFA) (20/80 (v/v)) to afford 42 mg of ***ortho*-S5** (0.11 mmol, 11 %) and 98 mg of ***para*-S5** (0.25 mmol, 25 %) as off-white solids.

**Data for *para*-S5:**

**NMR Spectroscopy:**

**$^1$H NMR** (500 MHz, $CD_3CN$, 23 ºC, δ): 7.86 (t, *J* = 7.4 Hz, 2H), 7.76 (t, *J* = 7.8 Hz, 4H), 7.69 (d, *J* = 7.6 Hz, 4H), 7.64 – 7.56 (m, 4H), 2.50 (s, 3H).

**$^{13}$C NMR** (125 MHz, $CD_3CN$, 23 ºC, δ): 147.0, 135.0, 132.6, 131.9, 131.6, 131.3, 125.3, 121.1, 21.0.

**$^{19}$F NMR** (470 MHz, $CD_3CN$, 23 ºC, δ): –76.51 (bs).

**HRMS-ESI(m/z)** calc'd for $C_{19}H_{17}S$ $[M–BF_4]^+$, 277.1045; found, 277.1044; deviation: 0.5 ppm.

**Data for *ortho*-S5:**

**NMR Spectroscopy:**

**$^1$H NMR** (500 MHz, $CD_3CN$, 23 ºC, δ): 7.89 (t, *J* = 7.4 Hz, 2H), 7.80 – 7.74 (m, 5H), 7.65 (dd, *J* = 13.9, 7.7 Hz, 5H), 7.54 (t, *J* = 7.7 Hz, 1H), 7.17 (d, *J* = 8.0 Hz, 1H), 2.55 (s, 3H).

**$^{13}$C NMR** (125 MHz, $CD_3CN$, 23 ºC, δ): 141.6, 135.4, 135.2, 133.6, 132.3, 131.9, 130.8, 129.6, 124.2, 123.5, 19.5.

**$^{19}$F NMR** (470 MHz, $CD_3CN$, 23 ºC, δ): –75.98 (bs).

**HRMS-ESI(m/z)** calc'd for $C_{19}H_{17}S$ $[M–BF_4]^+$, 277.1045; found, 277.1043; deviation: 0.7 ppm.

### Toluene derived diphenylsulfonium salt S5, *meta*-isomer

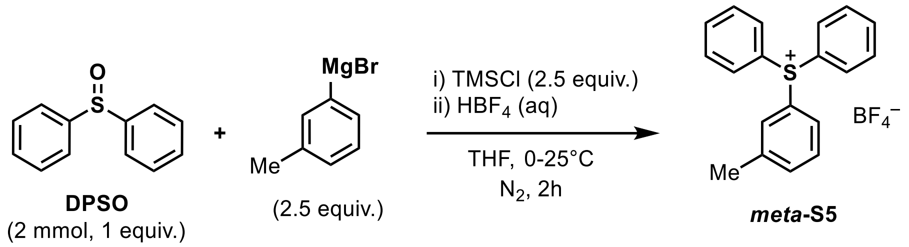


This product was prepared following a modified method to that reported in the literature.[3] Diphenylsulfoxide (202 mg, 1.00 mmol, 1.00 equiv.), was added to a solution of the Grignard reagent, pre-formed by reaction between Mg (121 mg, 5.00 mmol, 2.50 equiv.) and the 3-bromotoluene (606 µL, 5.00 mmol, 2.50 equiv.) in dry THF (10 mL). The mixture was then cooled to 0°C and TMSCl (630 µL, 5.00 mmol, 2.50 equiv.) was added dropwise. The cooling bath was removed and the mixture stirred at room temperature for 2 hours, then quenched slowly with an aqueous solution of $HBF_4$ (48% in $H_2O$, 4 mL) while stirring at 0°C. The resultant solution was diluted with 80 ml DCM and poured onto 80 ml water in a separatory funnel. After the layers were separated, the DCM layer was washed with aqueous $NaBF_4$ solution (50 ml, 5 % w/w). The organic phase was dried over $Mg_2SO_4$, filtered, and the solvent was removed under reduced pressure. The residue was purified by chromatography on silica gel eluting with DCM/*i*-PrOH (20:1, v/v) to afford ***meta*-S5** (592 mg, 1.62 mmol, 81 %) as a colorless solid after stored overnight in a desiccator.

**NMR Spectroscopy:**

**$^1$H NMR** (500 MHz, $CD_3CN$, 23 ºC, δ): 7.86 (tt, *J* = 7.4, 1.2 Hz, 2H), 7.79 – 7.73 (m, 4H), 7.71 – 7.67 (m, 5H), 7.64 (t, *J* = 7.8 Hz, 1H), 7.53 (s, 1H), 7.50 (d, *J* = 7.9 Hz, 1H), 2.43 (s, 3H).

**$^{13}$C NMR** (125 MHz, $CD_3CN$, 23 ºC, δ): 143.1, 136.1, 135.2, 132.1, 131.9, 131.8, 131.7, 128.8, 125.1, 124.7, 20.9.

**$^{19}$F NMR** (470 MHz, $CD_3CN$, 23 ºC, δ): –151.60 (bs), –151.66 (bs).

**HRMS-ESI(m/z)** calc'd for $C_{19}H_{17}S$ $[M–BF_4]^+$, 277.1045; found, 277.1048; deviation: 1.0 ppm.

## Aromatic thianthrenations in the absence of TFAA

### Table S1. Yields of 2 with different acids

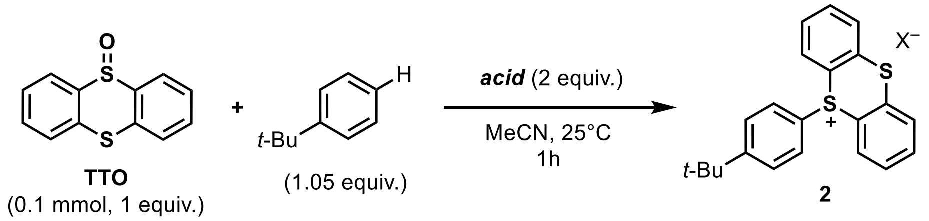


| Entry | Acid | Yield of **2** |
|---|---|---|

| | | |
|---|---|---|
| 1 | None | **-** |
| 2 | TFA | **-** |
| 3 | $HBF_4OEt_2$ | **21%** |
| 4 | TfOH | **57%** |
| 5 | $BF_3OEt_2$ | **-** |
| 6 | TMSOTf | **4%** |
| 7 | $Sc(OTf)_3$ | **-** |

## Reaction profile of the thianthrenation of toluene in the absence of TFAA

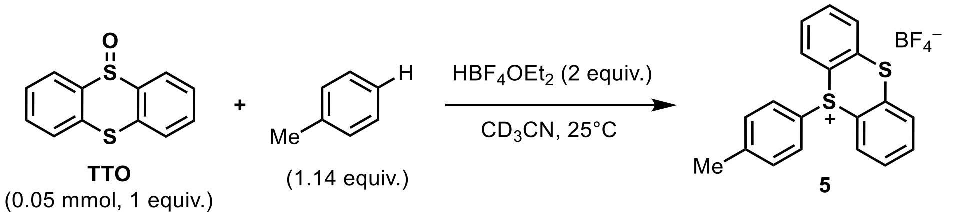


Under an ambient atmosphere, a 2 mL vial was charged with thianthrene-S-oxide (11.6 mg, 0.050 mmol, 1.00 equiv.), $CD_3CN$ (0.5 mL, c = 0.1 M), toluene (6 µL, 0.06 mmol, 1.1 equiv.), $CH_2Br_2$ (7 µL, 0.1 mmol, 2 equiv.) and the mixture was transferred to an NMR tube. $HBF_4{\cdot}OEt_2$ (13.5 µL, 0.100 mmol, 2.00 equiv.) was added and the evolution of the reaction mixture was monitored by $^1H$ NMR spectroscopy.

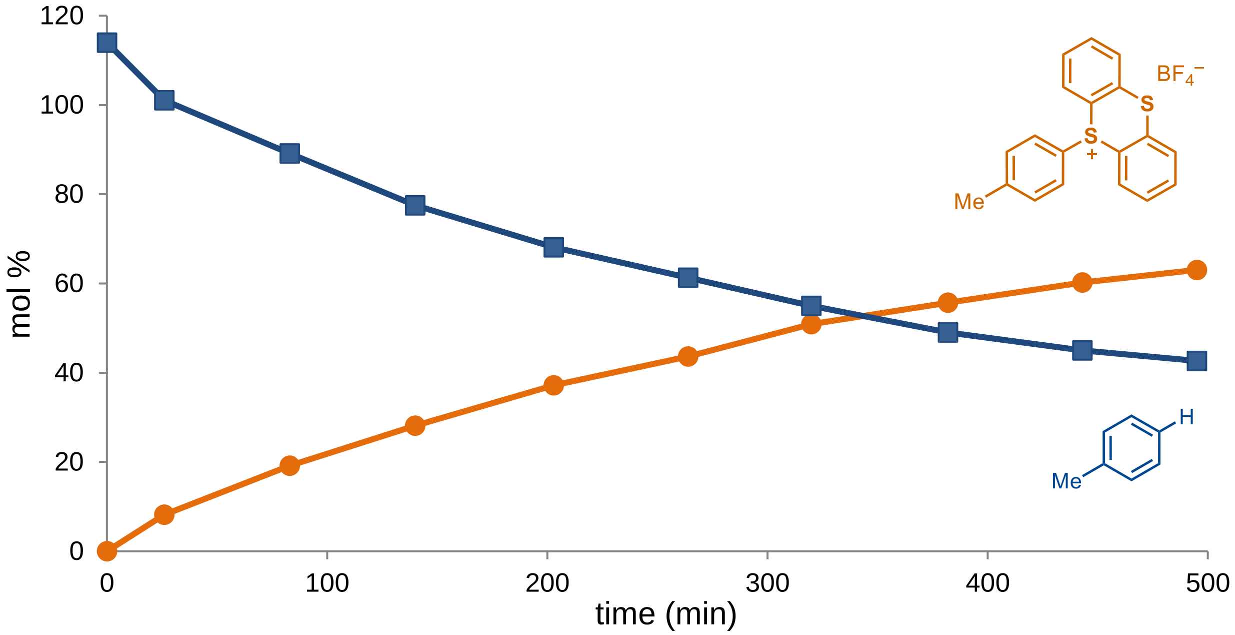


**Figure S2.** Reaction profile of thianthrenation of toluene with $HBF_4OEt_2$ in $CD_3CN$ followed by $^1H$ NMR.

## NMR studies on acylated derivatives of thianthrene-S-oxide

### Characterization and reactivity of $TT^+$-TFA

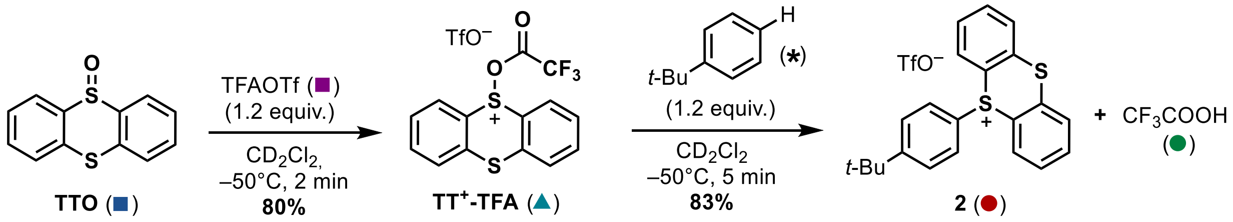


Under an ambient atmosphere, an NMR tube was charged with thianthrene-S-oxide (11.6 mg, 0.050 mmol, 1.00 equiv.) and dry $CD_2Cl_2$ (0.5 mL, c = 0.1 M) and capped with a septum. The tube was immersed in an ethanol/dry ice bath (T ~ –70°C). After 5 minutes, while maintaining the sample in the cooling bath, TFAOTf (15 μL, 0.060 mmol, 1.2 equiv.) was added with a microsyringe through the septum and the mixture was immediately transferred to the NMR spectrometer pre-cooled at –50°C. The mixture was then analyzed by $^1H$ and $^{19}F$ NMR spectroscopy.

To study the reactivity with arenes, the sample was taken from the spectrometer and immediately inmmersed in the ethanol/dry ice bath. After 2 minutes, while maintaining the sample in the cooling bath, *tert*-butylbenzene (9 μL, 0.06 mmol, 1.2 equiv.) was added with a microsyringe through the septum and the mixture was immediately transferred to the NMR spectrometer pre-cooled at –50°C. The mixture was then analyzed by $^1H$ and $^{19}F$ NMR spectroscopy.

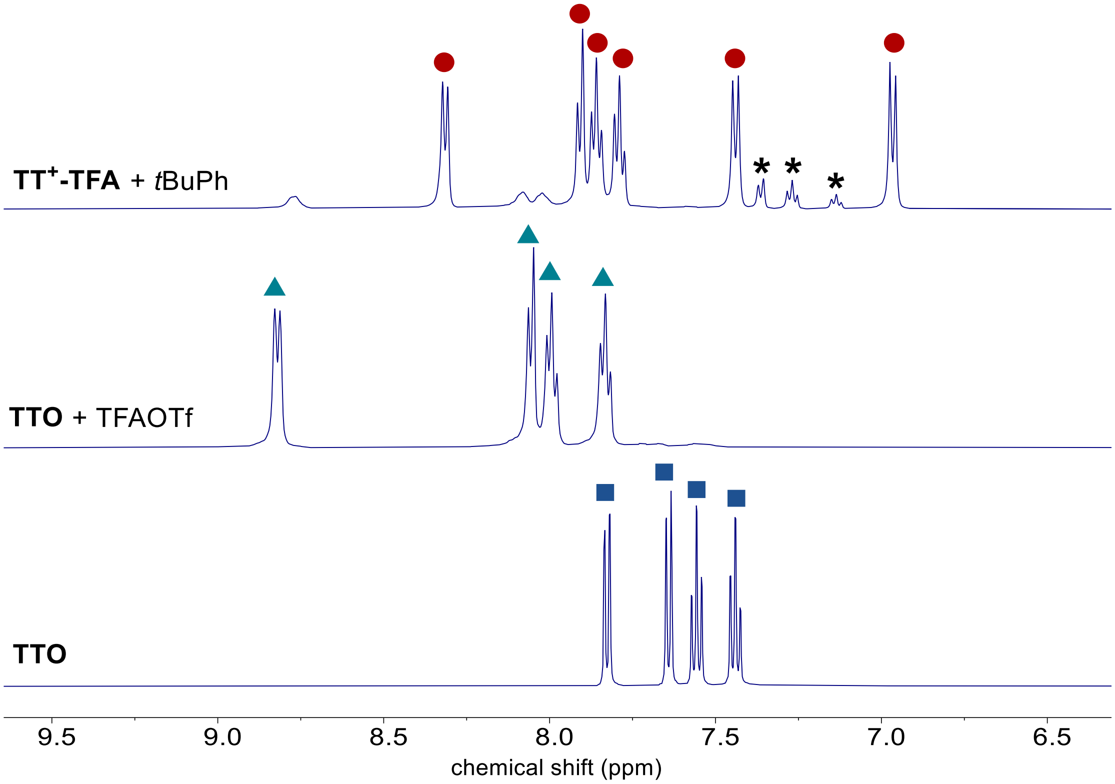

**Figure S3.** Generation and reactivity of **TT⁺-TFA** in $CD_3CN$ followed by $^1H$ NMR.

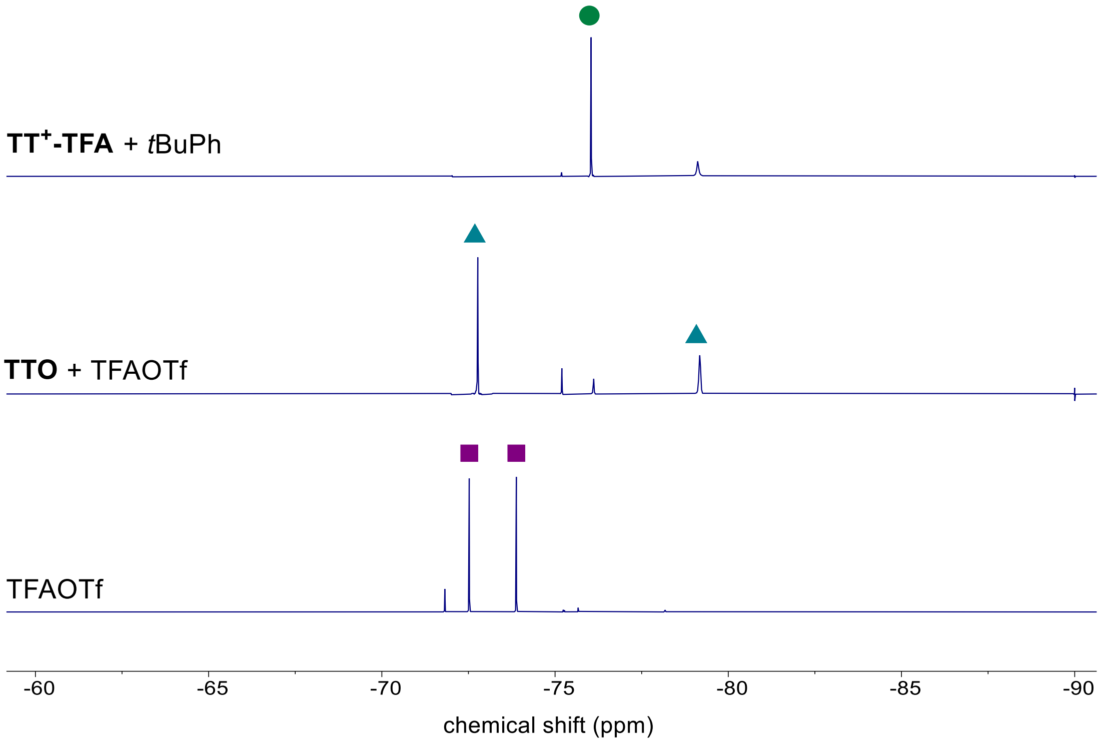


**Figure S4.** Generation and reactivity of **TT⁺-TFA** in $CD_3CN$ followed by $^{19}F$ NMR.

**NMR Spectroscopy data for TT⁺-TFA:**

**$^1$H NMR** (500 MHz, $CD_2Cl_2$, -50 ºC, δ): 8.58 (d, $J$ = 7.2 Hz, 2H), 8.00 (d, $J$ = 7.2 Hz, 2H), 7.91 (d, $J$ = 7.4 Hz, 2H), 7.75 (t, $J$ = 7.3 Hz, 2H).

**$^{19}$F NMR** (470 MHz, $CD_2Cl_2$, -50 ºC, δ): -79.4.

## Generation of TT⁺-TFA using TFAA and TfOH

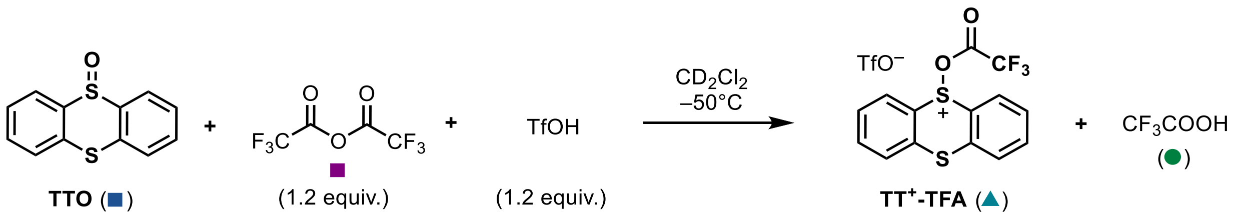


Under an ambient atmosphere, an NMR tube was charged with thianthrene-S-oxide (11.6 mg, 0.050 mmol, 1.00 equiv.) and dry $CD_2Cl_2$ (0.5 mL, c = 0.1 M) and capped with a septum. The tube was immersed in an ethanol/dry ice bath (T ~ –70°C). After 5 minutes, while maintaining the sample in the cooling bath, TFAA (8.3 μL, 0.060 mmol, 1.2 equiv.) was added with a microsyringe through the septum and the mixture was immediately transferred to the NMR spectrometer pre-cooled at –50°C. The mixture was then analyzed by $^1H$ and $^{19}F$ NMR spectroscopy both at –50°C and 25°C .

The sample was taken from the spectrometer inmmersed in the ethanol/dry ice bath. After 2 minutes, while maintaining the sample in the cooling bath, trifluoromethanesulfonic acid (5.3 µL, 0.060 mmol, 1.2 equiv.) was added with a microsyringe through the septum and the mixture was immediately transferred to the NMR spectrometer pre-cooled at –50°C. The mixture was then analyzed by $^{1}H$ and $^{19}F$ NMR spectroscopy.

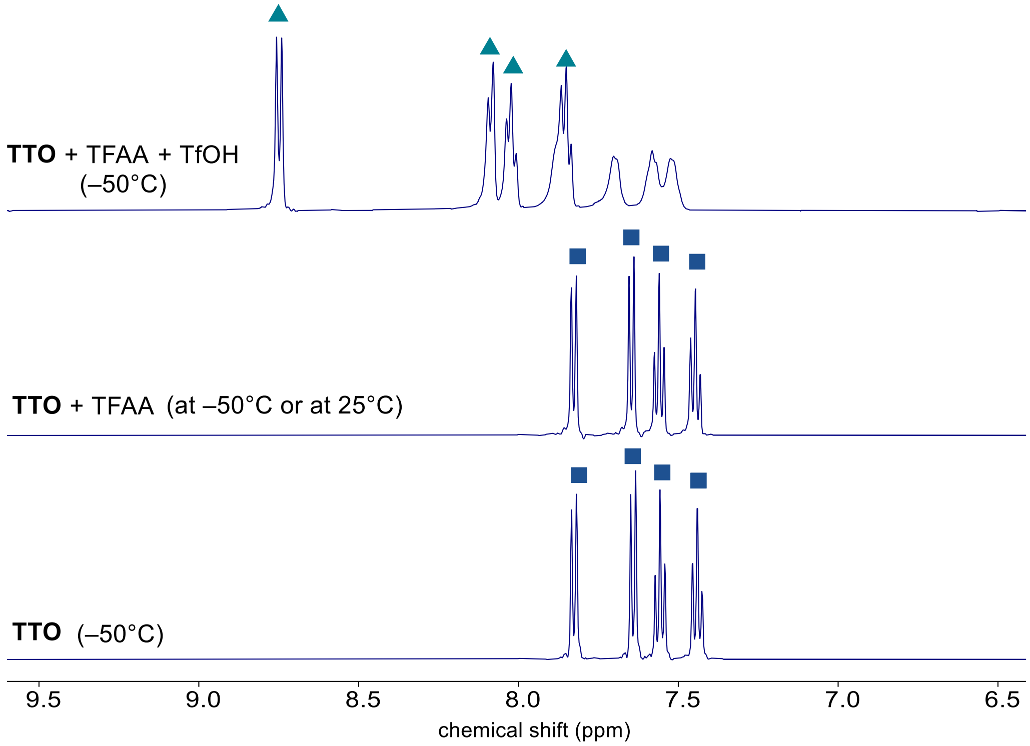


**Figure S5.** Generation of **TT⁺-TFA** from **TTO** + TFAA + TfOH in $CD_3CN$ followed by $^{1}H$ NMR.

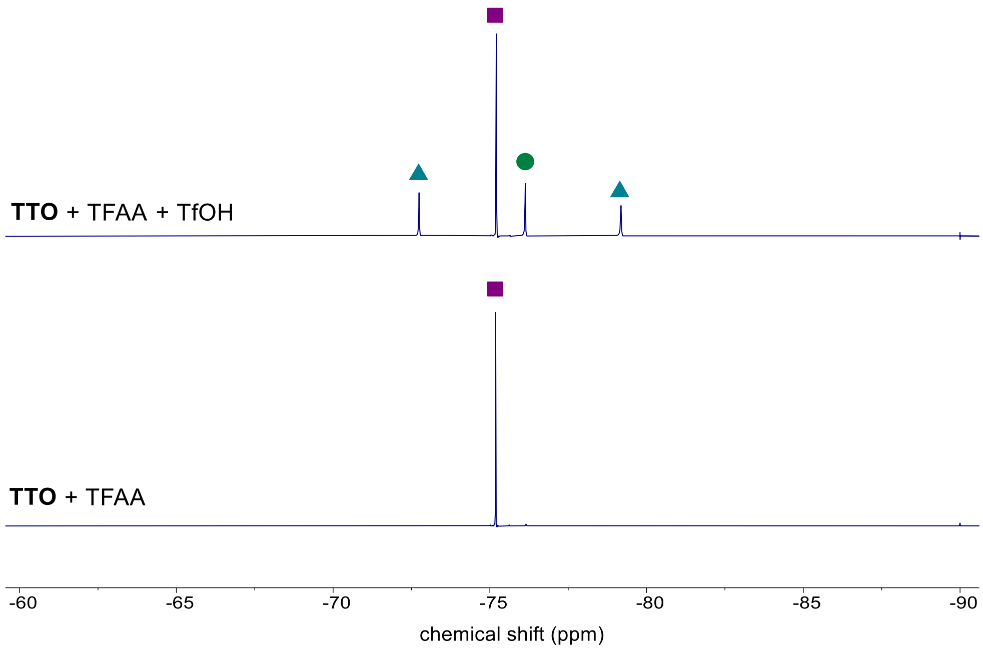

**Figure S6.** Generation of **TT⁺-TFA** from **TTO** + TFAA + TfOH in $CD_3CN$ followed by $^{19}F$ NMR.

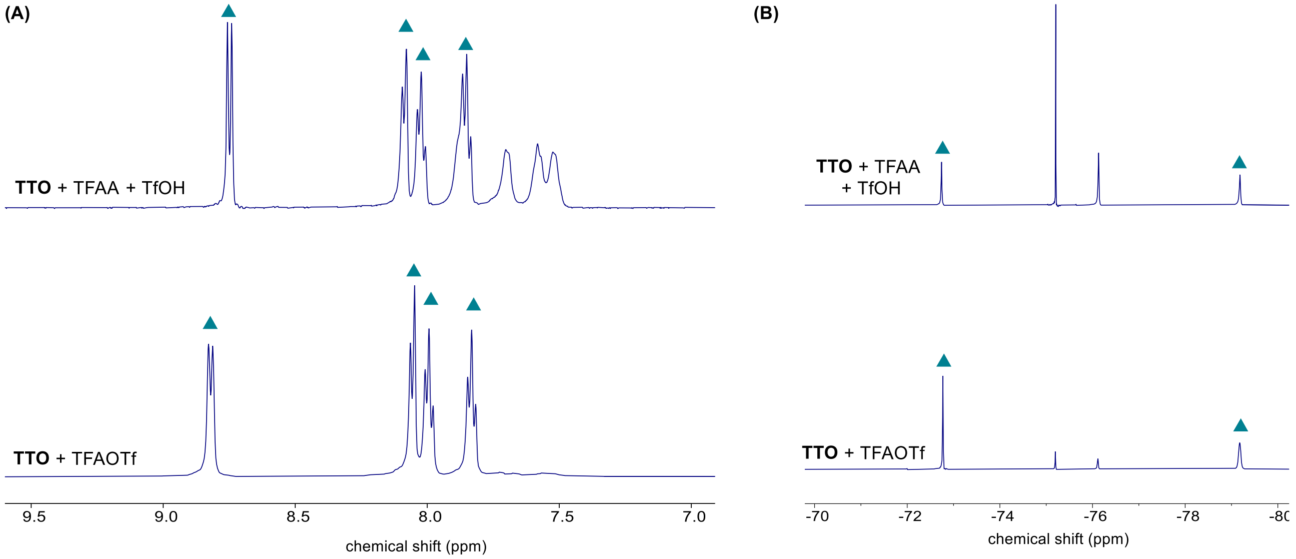


**Figure S7.** Comparison of the $^{1}H$ (A) and $^{19}F$ (B) NMR spectra of **TT⁺-TFA** generated from **TTO**+TFAA+TfOH or **TTO**+TFAOTf.

## Generation of TT⁺-OAc using $Ac_2O$ and TfOH

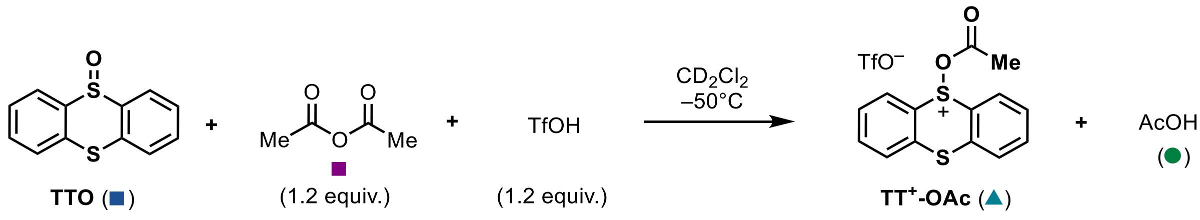


Under an ambient atmosphere, an NMR tube was charged with thianthrene-S-oxide (11.6 mg, 0.050 mmol, 1.00 equiv.) and dry $CD_2Cl_2$ (0.5 mL, c = 0.1 M) and capped with a septum. The tube was immersed in an ethanol/dry ice bath (T ~ –70°C). After 5 minutes, while maintaining the sample in the cooling bath, $Ac_2O$ (5.7 μL, 0.060 mmol, 1.2 equiv.) was added with a microsyringe through the septum and the mixture was immediately transferred to the NMR spectrometer pre-cooled at –50°C. The mixture was then analyzed by $^{1}H$ NMR spectroscopy.

The sample was taken from the spectrometer inmmersed in the ethanol/dry ice bath. After 2 minutes, while maintaining the sample in the cooling bath, trifluoromethanesulfonic acid (5.3 μL, 0.060 mmol, 1.2 equiv.) was added with a microsyringe through the septum and the mixture was immediately transferred to the NMR spectrometer pre-cooled at –50°C. The mixture was then analyzed by $^{1}H$ and $^{19}F$ NMR spectroscopy.

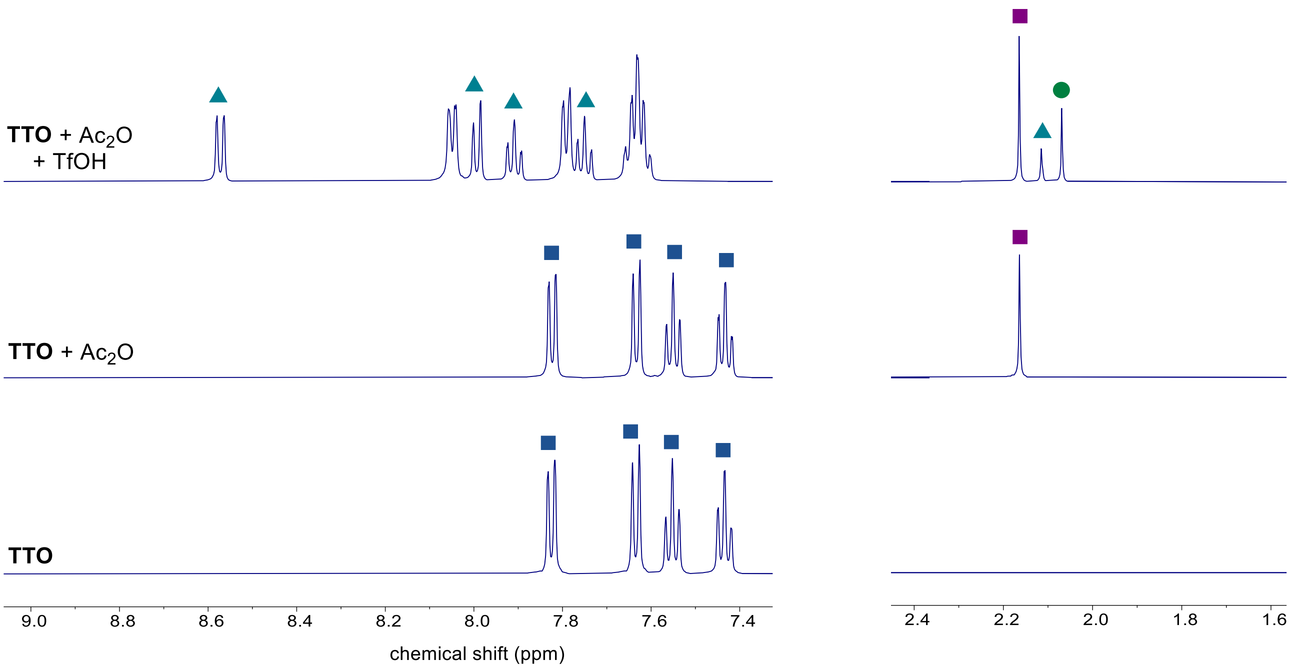


**Figure S8.** Generation of **TT⁺-OAc** from **TTO** + $Ac_2O$ + TfOH in $CD_3CN$ followed by $^1H$ NMR.

**NMR Spectroscopy data for TT⁺-OAc:**

**$^1$H NMR** (500 MHz, $CD_2Cl_2$, -50 °C, δ): 8.57 (dd, *J* = 8.1, 1.3 Hz, 2H), 7.99 (dd, *J* = 8.1, 1.2 Hz, 2H), 7.91 (td, *J* = 7.7, 1.4 Hz, 2H), 7.75 (td, *J* = 7.8, 7.4, 1.2 Hz, 2H), 2.12 (s, 3H)

## Reactivity modulation of activated thianthrene-S-oxide

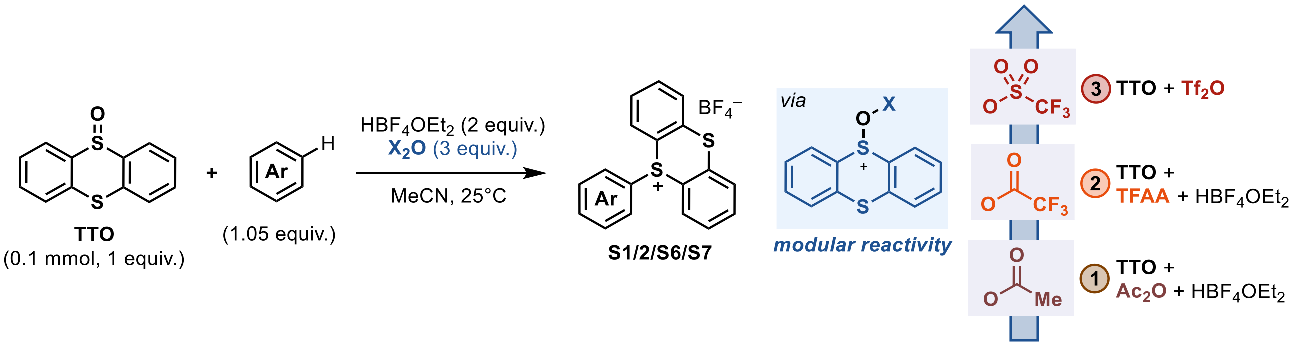


Under an ambient atmosphere, a 4 ml glass-vial equipped with a stir bar was charged with thianthrene-S-oxide (23.2 mg, 0.100 mmol, 1.00 equiv.), dry MeCN (1 mL, c = 0.1 M) and the arene (0.105 mmol, 1.05 equiv.). A suitable anhydride $X_2O$ (0.3 mmol, 3.0 equiv.) and then $HBF_4{\cdot}OEt_2$ (27 µL, 0.20 mmol, 2.0 equiv.) were subsequently added to the vial while stirring the reaction mixture. When using $Tf_2O$ the acid was not required. The vial was sealed with a screw-cap and the mixture was stirred at 25 °C for 2 h. The solution was diluted with 10 ml DCM and poured onto 10 ml of saturated aqueous $NaHCO_3$ solution in a separatory funnel. After the layers were separated, the DCM layer was washed with aqueous $NaBF_4$ solution (10 ml, 5 % w/w). The organic phase was dried over $Mg_2SO_4$, filtered, and the solvent was removed under reduced pressure.

The resultant residue was dissolved in $CD_3CN$ (0.5 mL), internal standard $CH_2Br_2$ (14 µL, 0.20 mmol, 2.0 equiv.) was added and the mixture was analyzed by $^1H$ NMR spectroscopy. NMR yields of the different thianthrenium salts are given in Figure S9.

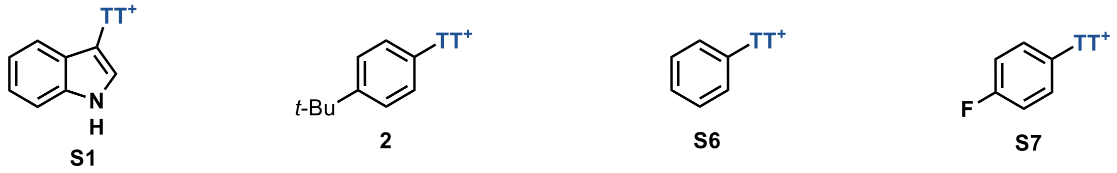


| $X_2O$ | yield (S1) | yield (2) | yield (S6) | yield (S7) |
|---|---|---|---|---|
| $Ac_2O$ | 47% | 43% | trace | n.d. |
| TFAA | 62% | 93% | 24% | 18% |
| $Tf_2O$ | 33% | 92% | 73% | 55% |

**Figure S9.** Influence of the anhydride activator on the yields for thianthrenation reactions with different arenes.

## Site selectivity on thrianthrenations

### Selectivity determination on thianthrenation with thianthrene-S-oxide/TFAA/$HBF_4OEt_2$

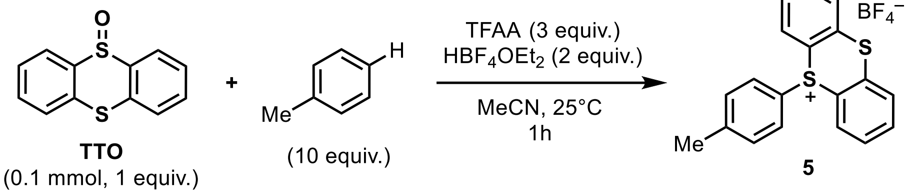


Under an ambient atmosphere, a 4 ml glass-vial equipped with a stir bar was charged with thianthrene-S-oxide (23.2 mg, 0.100 mmol, 1.00 equiv.), dry MeCN (1 mL, c = 0.1 M) and toluene (106 µL, 1.00 mmol, 10.0 equiv.). TFAA (42 µL, 0.30 mmol, 3.0 equiv.) and then $HBF_4{\cdot}OEt_2$ (27 µL, 0.20 mmol, 2.0 equiv.) were subsequently added to the vial while stirring the reaction mixture. The vial was sealed with a screw-cap and the mixture was stirred at 25 °C for 1 h. The solution was diluted with 10 ml DCM and poured onto 10 ml of saturated aqueous $NaHCO_3$ solution in a separatory funnel. After the layers were separated, the DCM layer was washed with aqueous $NaBF_4$ solution (10 ml, 5 % w/w). The organic phase was dried over $Mg_2SO_4$, filtered, and the solvent was removed under reduced pressure. The resultant residue was dissolved in $CD_3CN$ (0.5 mL) and analyzed by $^1H$ NMR spectroscopy.

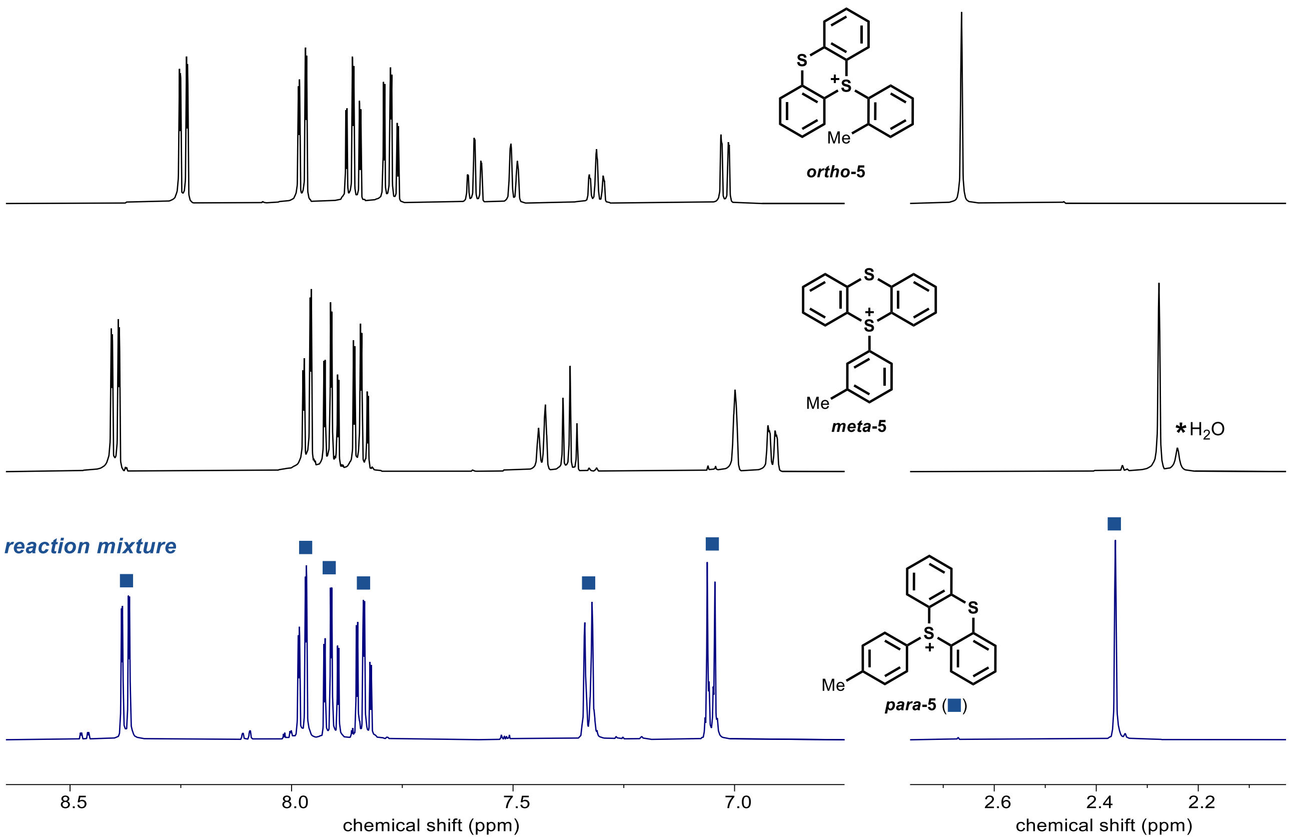


**Figure S10.** $^1$H NMR of reaction mixture, in comparison to *ortho* and *meta* isomers of **5**, $CD_3CN$, 500 MHz, 298 K.

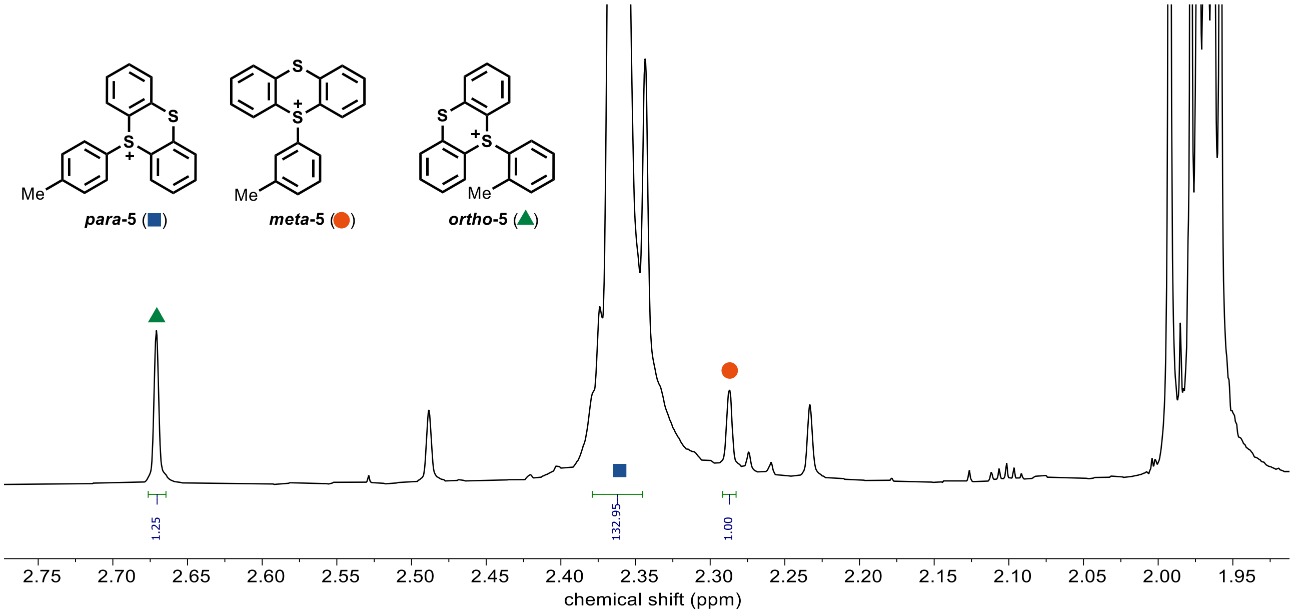


**Figure S11.** Determination of the selectivity in the reaction mixture, $CD_3CN$, 500 MHz, 298 K.

## Selectivity determination on thianthrenation with thianthrene-S-oxide in $H_2SO_4$

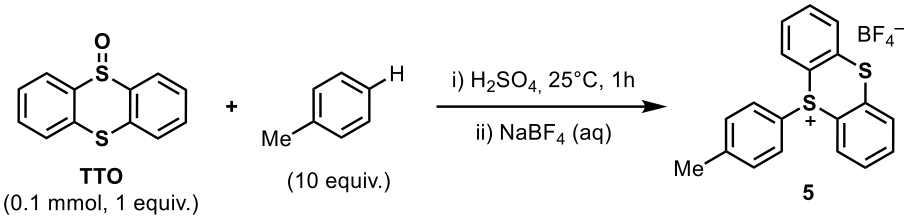


Under an ambient atmosphere, a 4 ml glass-vial equipped with a stir bar was charged with thianthrene-S-oxide (23.2 mg, 0.10 mmol, 1.00 equiv.), $H_2SO_4$ (1 mL, c = 0.1 M) and toluene (106 µL, 1.00 mmol, 10.0 equiv.). The vial was sealed with a screw-cap and the mixture was stirred at 25 °C for 1 h. The solution was then poured onto ice. Once the ice melted, 10 mL of DCM were added and the mixture was transferred to a separatory funnel. The DCM layer was washed with a saturated aqueous $NaHCO_3$ solution (10 mL) and aqueous $NaBF_4$ solution (2x10 ml, 5 % w/w). The organic phase was then dried over $Mg_2SO_4$, filtered, and the solvent was removed under reduced pressure. The resultant residue was dissolved in $CD_3CN$ (0.5 mL) and analyzed by $^1H$ NMR spectroscopy.

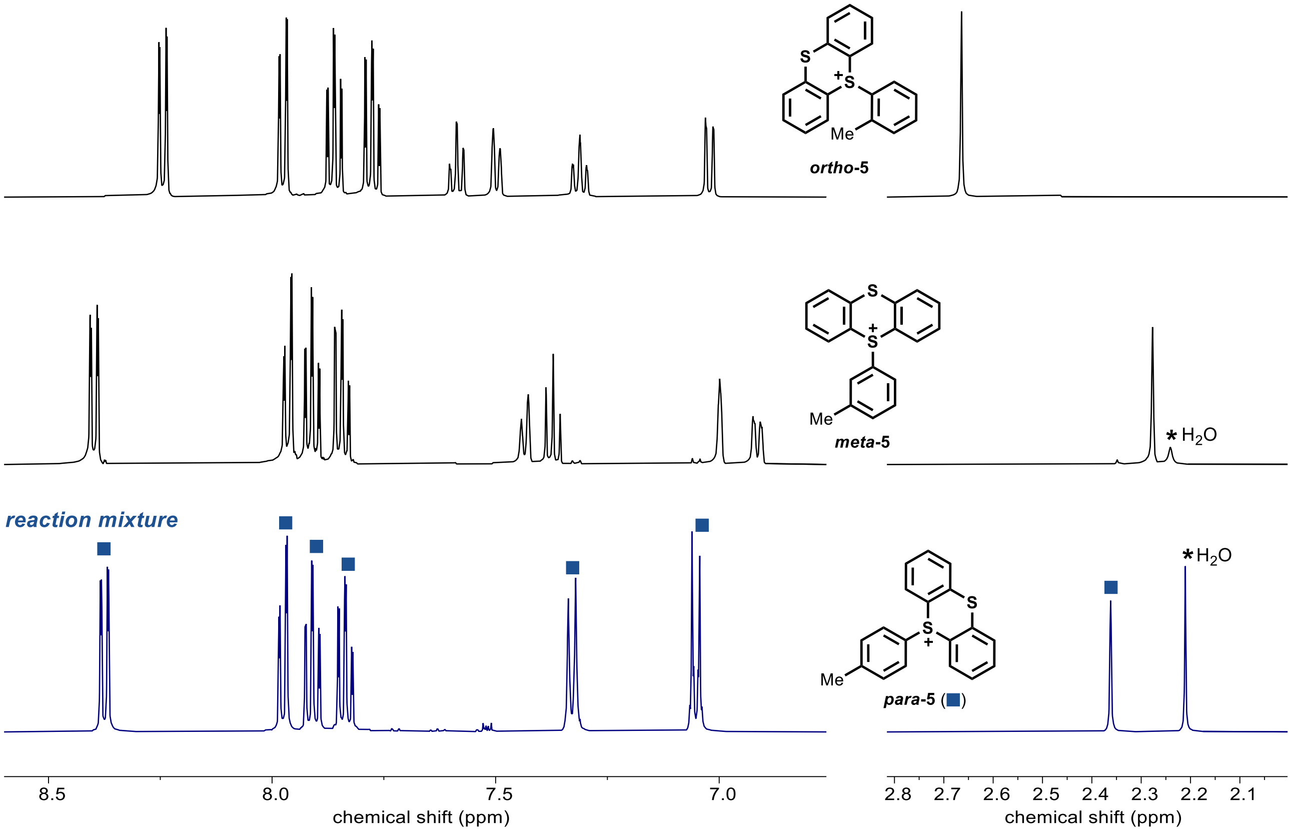


**Figure S12.** $^1H$ NMR of reaction mixture, in comparison to *ortho* and *meta* isomers of **5**, $CD_3CN$, 500 MHz, 298 K.

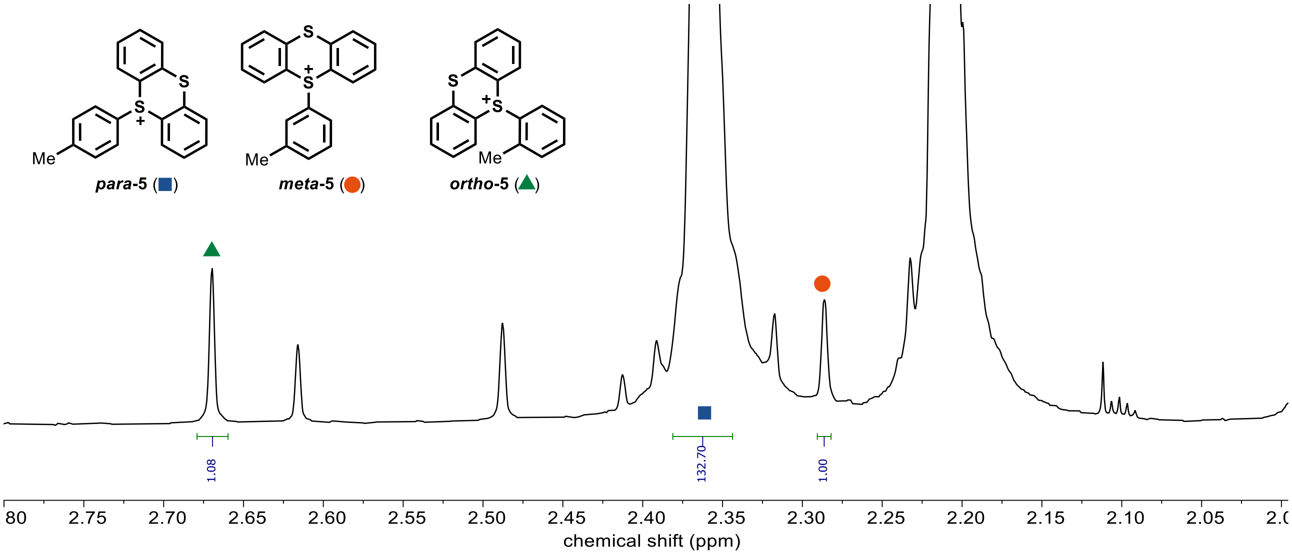


**Figure S13.** Determination of the selectivity in the reaction mixture, $CD_3CN$, 500 MHz, 298 K.

## Selectivity determination on thianthrenation with thianthrenium radical cation

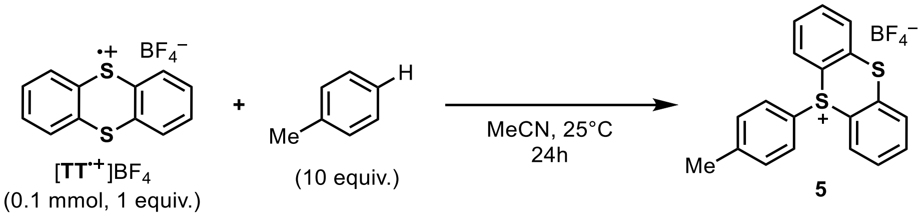


In a nitrogen-filled glove box, a 4 mL vial equipped with a stirbar was charged with thianthrene radical cation tetrafluoroborate (30.3 mg, 0.100 mmol, 1.00 equiv.) and dry MeCN (1 mL, c = 0.1 M). The vial was sealed with a septum-cap, removed from the glove box, and toluene (106 µL, 1.0 mmol, 10 equiv.) was added via the septum. The mixture was stirred at 25 °C for 24 h. The solution was diluted with 10 ml DCM and poured onto 10 ml of saturated aqueous $NaHCO_3$ solution in a separatory funnel. After the layers were separated, the DCM layer was washed with aqueous $NaBF_4$ solution (10 ml, 5 % w/w). The organic phase was dried over $Mg_2SO_4$, filtered, and the solvent was removed under reduced pressure. The resultant residue was dissolved in $CD_3CN$ (0.5 mL) and analyzed by $^1H$ NMR spectroscopy.

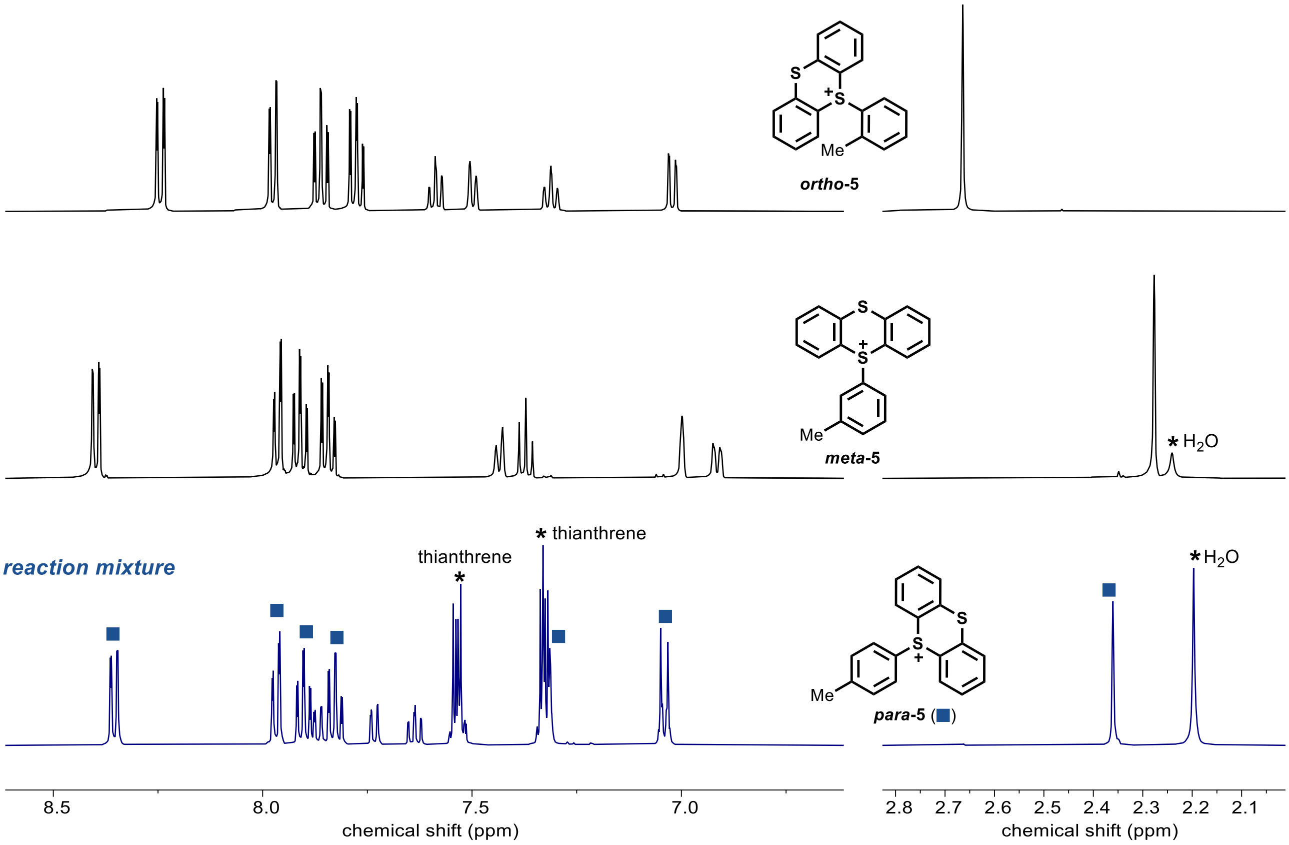


**Figure S14.** [1]H NMR of reaction mixture, in comparison to *ortho* and *meta* isomers of **5**, $CD_3CN$, 500 MHz, 298 K.

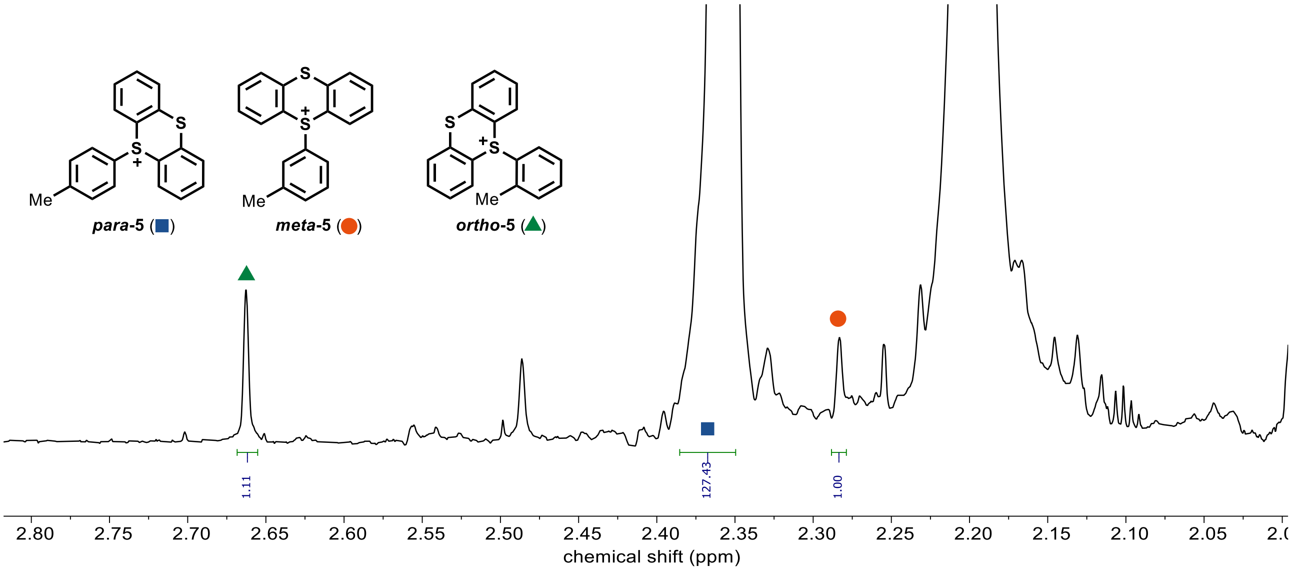


**Figure S15.** Determination of the selectivity in the reaction mixture, $CD_3CN$, 500 MHz, 298 K.

### Selectivity determination on thianthrenation with thianthrene-S-oxide/TFAOTf/$K_2CO_3$

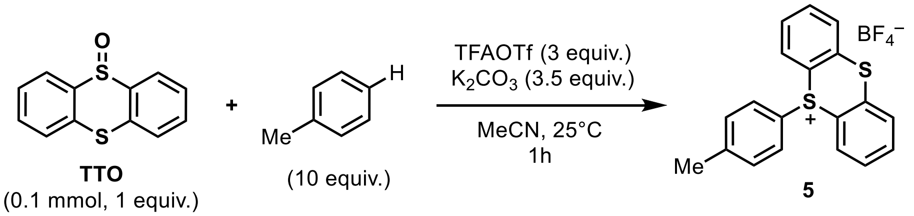


Under an ambient atmosphere, a 4 ml glass-vial equipped with a stir bar was charged with thianthrene-S-oxide (23.2 mg, 0.100 mmol, 1.00 equiv.), $K_2CO_3$ (48.4 mg, 0.350 mmol, 3.50 equiv), dry MeCN (1 mL, c = 0.1 M) and toluene (106 µL, 1.00 mmol, 10.0 equiv.). TFAOTf (74 mg, 0.30 mmol, 3.0 equiv.) was then added to the vial while stirring the reaction mixture. The vial was sealed with a screw-cap and the mixture was stirred at 25 °C for 1 h. The solution was diluted with 10 ml DCM and poured onto 10 ml of saturated aqueous $NaHCO_3$ solution in a separatory funnel. After the layers were separated, the DCM layer was washed with aqueous $NaBF_4$ solution (10 ml, 5 % w/w). The organic phase was dried over $Mg_2SO_4$, filtered, and the solvent was removed under reduced pressure. The resultant residue was dissolved in $CD_3CN$ (0.5 mL) and analyzed by $^1$H NMR spectroscopy.

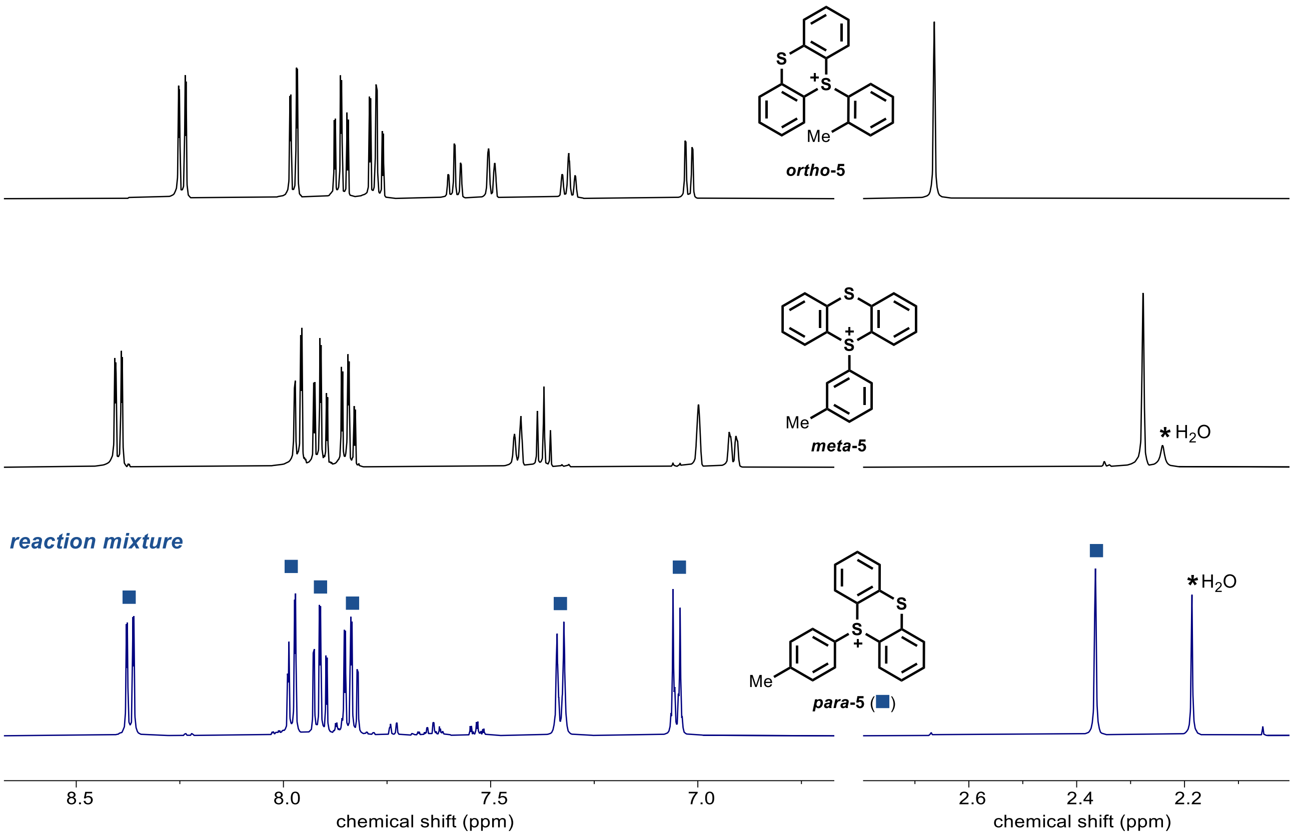


**Figure S16.** $^1$H NMR of reaction mixture, in comparison to *ortho* and *meta* isomers of **5**, $CD_3CN$, 500 MHz, 298 K.

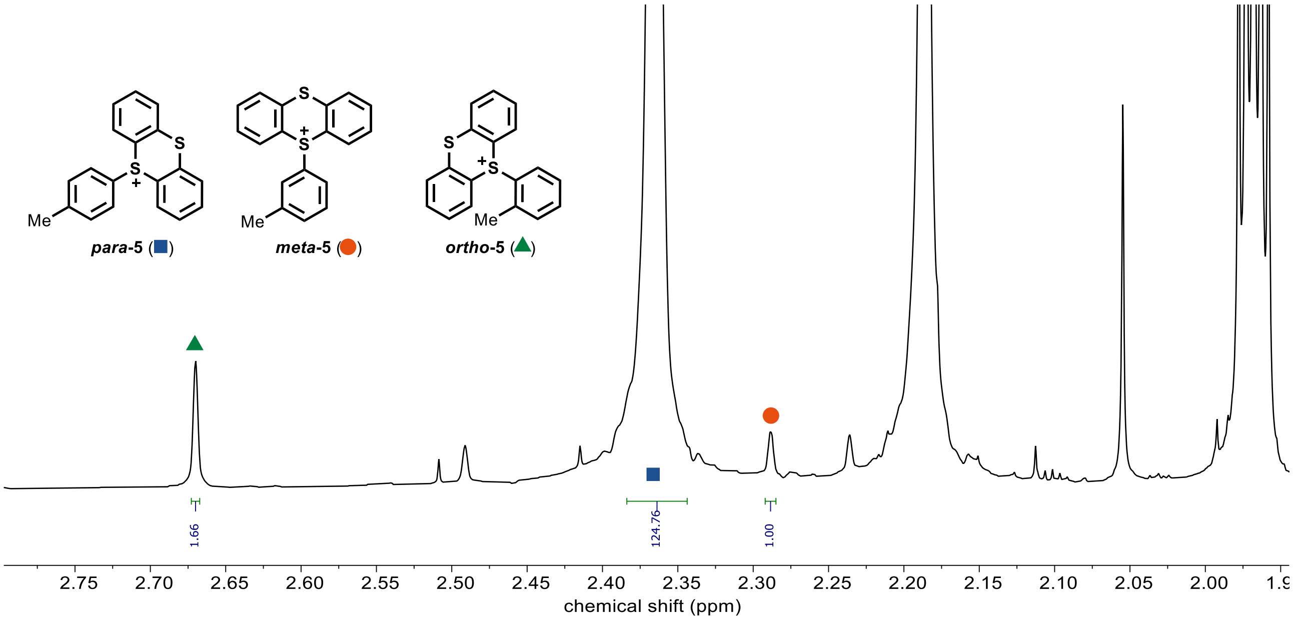


**Figure S17.** Determination of the selectivity in the reaction mixture, $CD_3CN$, 500 MHz, 298 K.

## Site selectivity on C–H functionalization with other sulfoxides

### Selectivity determination on C–H functionalization with tetrafluorothianthrene-S-oxide/TFAA/$HBF_4OEt_2$

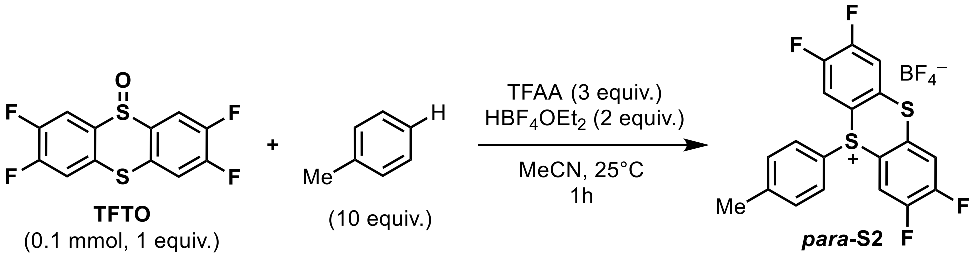


Under an ambient atmosphere, a 4 ml glass-vial equipped with a stir bar was charged with tetrafluorothianthrene-S-oxide (30.4 mg, 0.100 mmol, 1.0 equiv.), dry MeCN (1 mL, c = 0.1 M) and toluene (106 µL, 1.00 mmol, 10.0 equiv.). TFAA (42 µL, 0.30 mmol, 3.0 equiv.) and then $HBF_4{\cdot}OEt_2$ (27 µL, 0.20 mmol, 2.0 equiv.) were subsequently added to the vial while stirring the reaction mixture. The vial was sealed with a screw-cap and the mixture was stirred at 25 °C for 1 h. The solution was diluted with 10 ml DCM and poured onto 10 ml of saturated aqueous $NaHCO_3$ solution in a separatory funnel. After the layers were separated, the DCM layer was washed with aqueous $NaBF_4$ solution (10 ml, 5 % w/w). The organic phase was dried over $Mg_2SO_4$, filtered, and the solvent was removed under reduced pressure. The resultant residue was dissolved in $CD_3CN$ (0.5 mL) and analyzed by $^1H$ NMR spectroscopy.

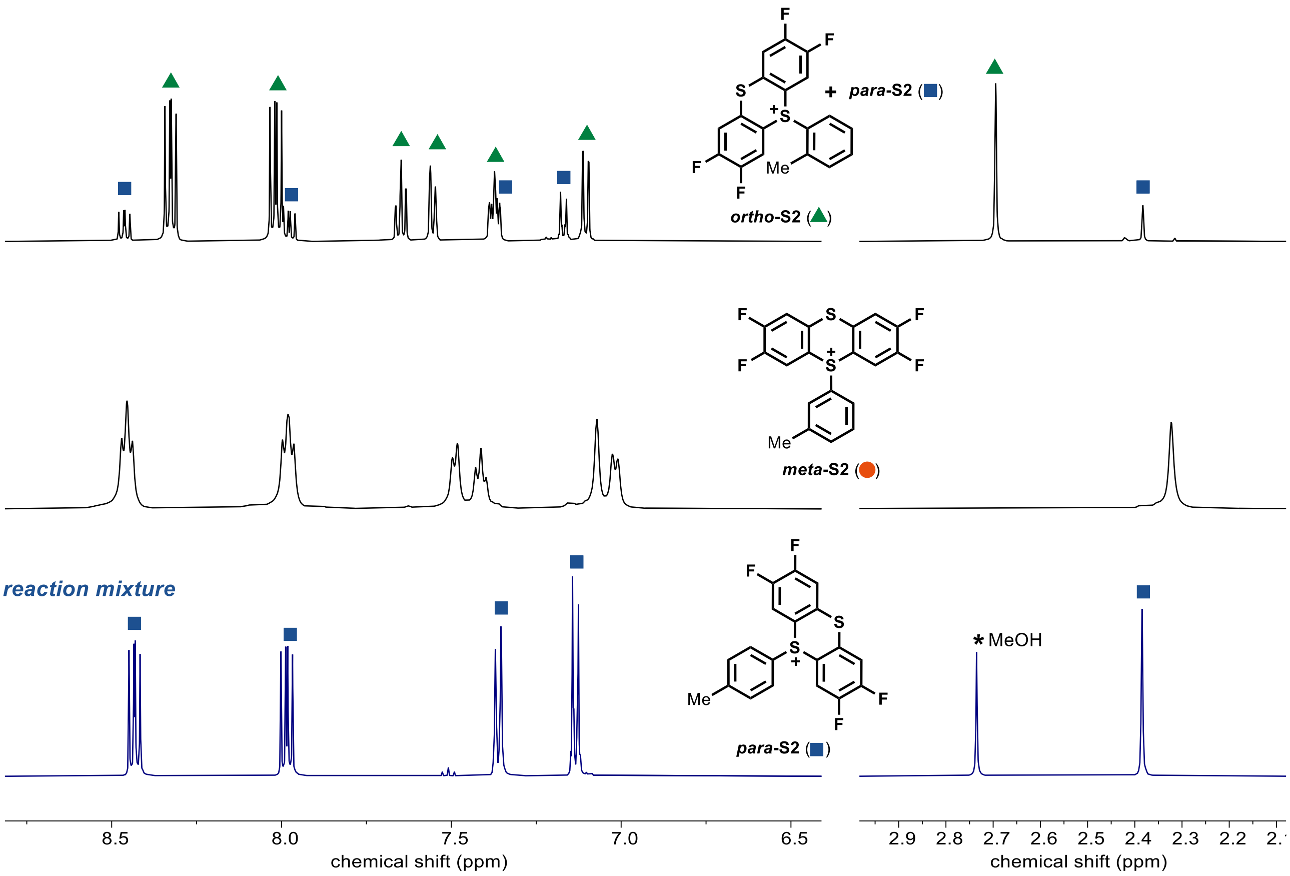


**Figure S18.** $^{1}$H NMR of reaction mixture, in comparison to *ortho* and *meta* isomers of **S2**, $CD_3CN$, 500 MHz, 298 K.

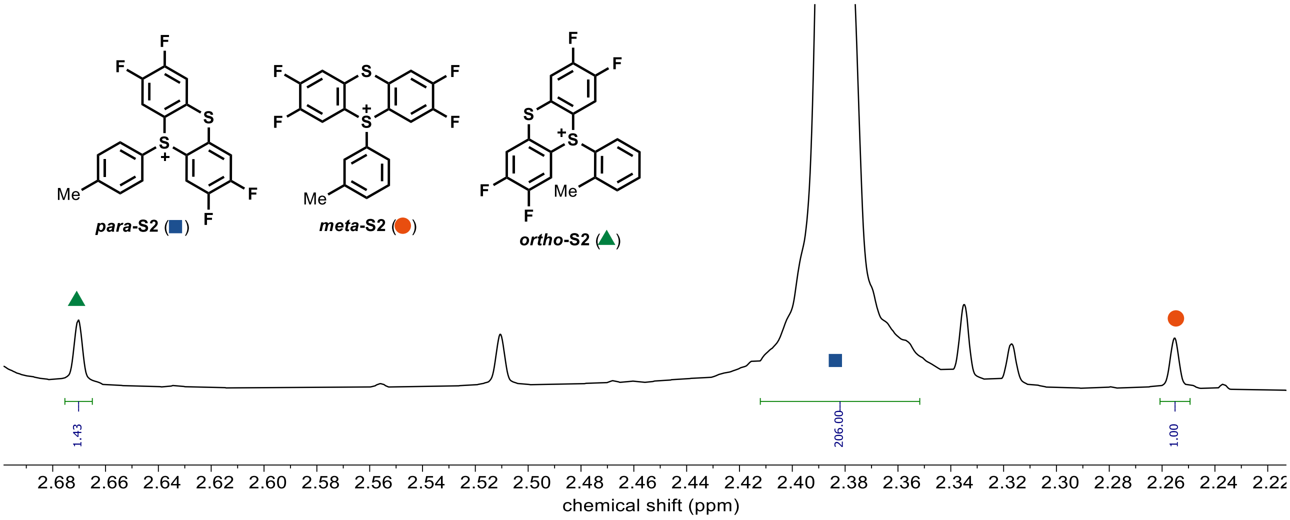


**Figure S19.** Determination of the selectivity in the reaction mixture, $CD_3CN$, 500 MHz, 298 K.

## Selectivity determination on C–H functionalization with phenoxathiin-S-oxide/TFAA/$HBF_4OEt_2$

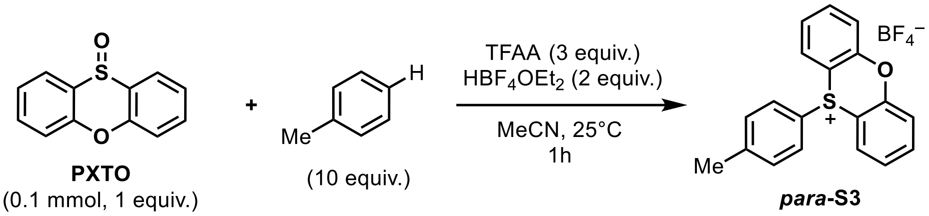


Under an ambient atmosphere, a 4 ml glass-vial equipped with a stir bar was charged with phenoxathiine-S-oxide (21.6 mg, 0.100 mmol, 1.0, equiv.), dry MeCN (1 mL, c = 0.1 M) and toluene (106 µL, 1.00 mmol, 10.0 equiv.). TFAA (42 µL, 0.30 mmol, 3.0 equiv.) and then $HBF_4·OEt_2$ (27 µL, 0.20 mmol, 2.0 equiv.) were subsequently added to the vial while stirring the reaction mixture. The vial was sealed with a screw-cap and the mixture was stirred at 25 °C for 1 h. The solution was diluted with 10 ml DCM and poured onto 10 ml of saturated aqueous $NaHCO_3$ solution in a separatory funnel. After the layers were separated, the DCM layer was washed with aqueous $NaBF_4$ solution (10 ml, 5 % w/w). The organic phase was dried over $Mg_2SO_4$, filtered, and the solvent was removed under reduced pressure. The resultant residue was dissolved in $CD_3CN$ (0.5 mL) and analyzed by $^1H$ NMR spectroscopy.

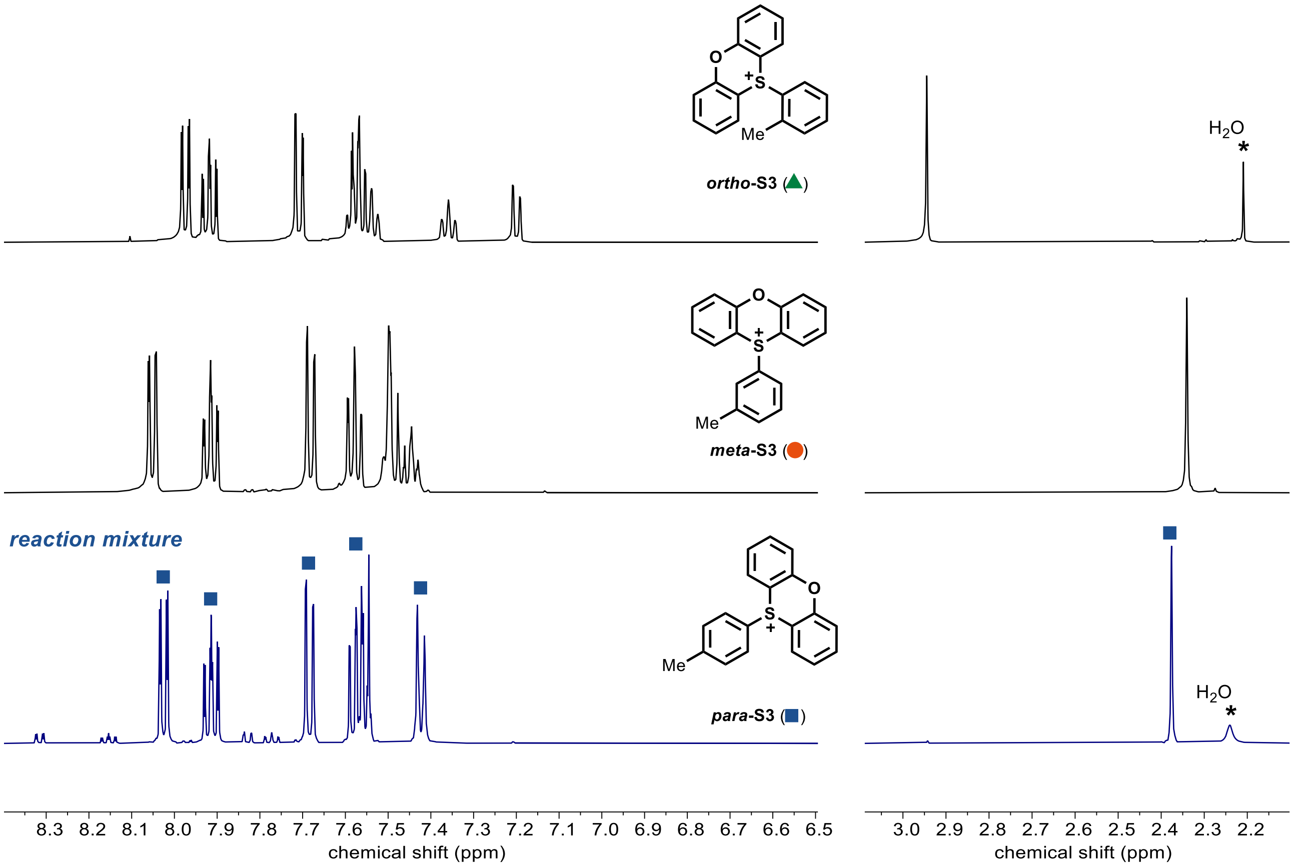


**Figure S20.** $^1H$ NMR of reaction mixture, in comparison to *ortho* and *meta* isomers of **S3**, $CD_3CN$, 500 MHz, 298 K.

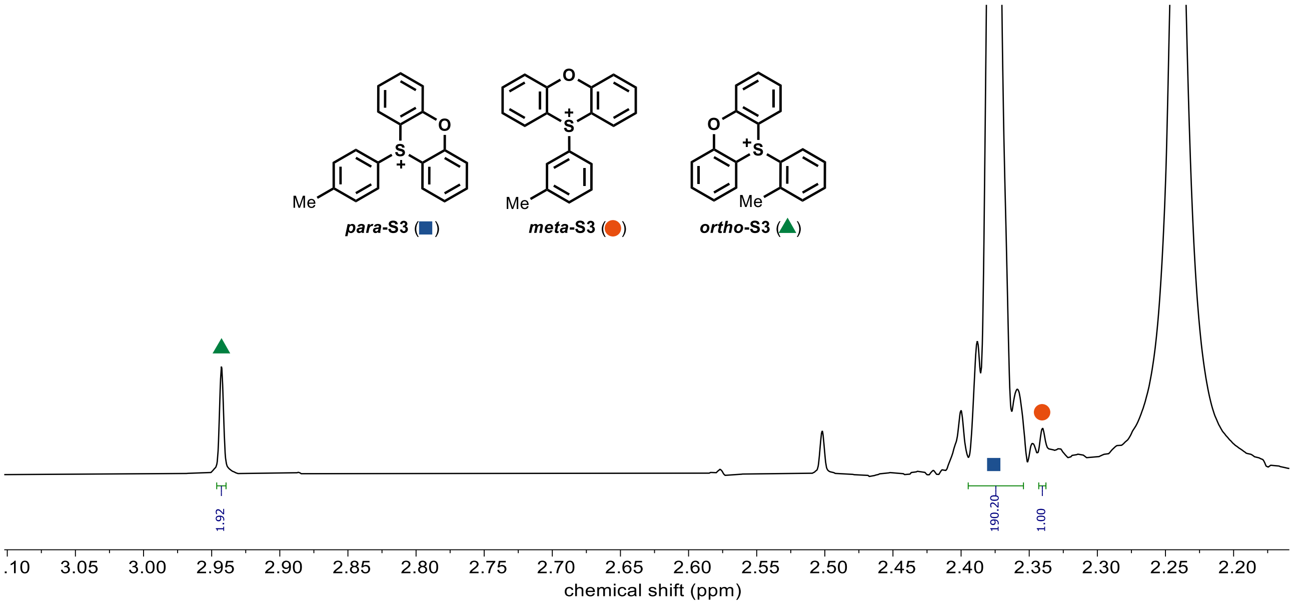


**Figure S21.** Determination of the selectivity in the reaction mixture, $CD_3CN$, 500 MHz, 298 K.

## Selectivity determination on C–H functionalization with dibenzothiophene-S-oxide/TFAA/$HBF_4OEt_2$

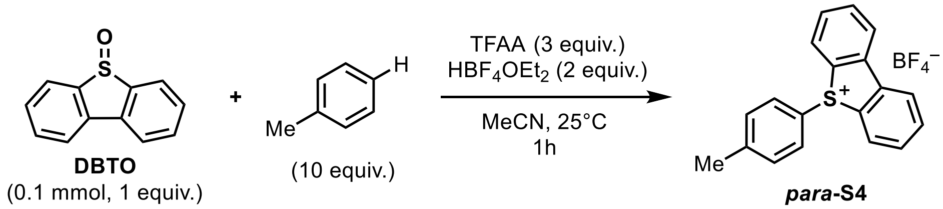


Under an ambient atmosphere, a 4 ml glass-vial equipped with a stir bar was charged with dibenzothiophene-S-oxide (20 mg, 0.10 mmol, 1.00 equiv.), dry MeCN (1 mL, c = 0.1 M) and toluene (106 µL, 1.00 mmol, 10.0 equiv.). TFAA (42 µL, 0.3 mmol, 3.0 equiv.) and then $HBF_4{\cdot}OEt_2$ (27 µL, 0.2 mmol, 2.0 equiv.) were subsequently added to the vial while stirring the reaction mixture. The vial was sealed with a screw-cap and the mixture was stirred at 25 °C for 1 h. The solution was diluted with 10 ml DCM and poured onto 10 ml of saturated aqueous $NaHCO_3$ solution in a separatory funnel. After the layers were separated, the DCM layer was washed with aqueous $NaBF_4$ solution (10 ml, 5 % w/w). The organic phase was dried over $Mg_2SO_4$, filtered, and the solvent was removed under reduced pressure. The resultant residue was dissolved in $CD_3CN$ (0.5 mL) and analyzed by $^1H$ NMR spectroscopy.

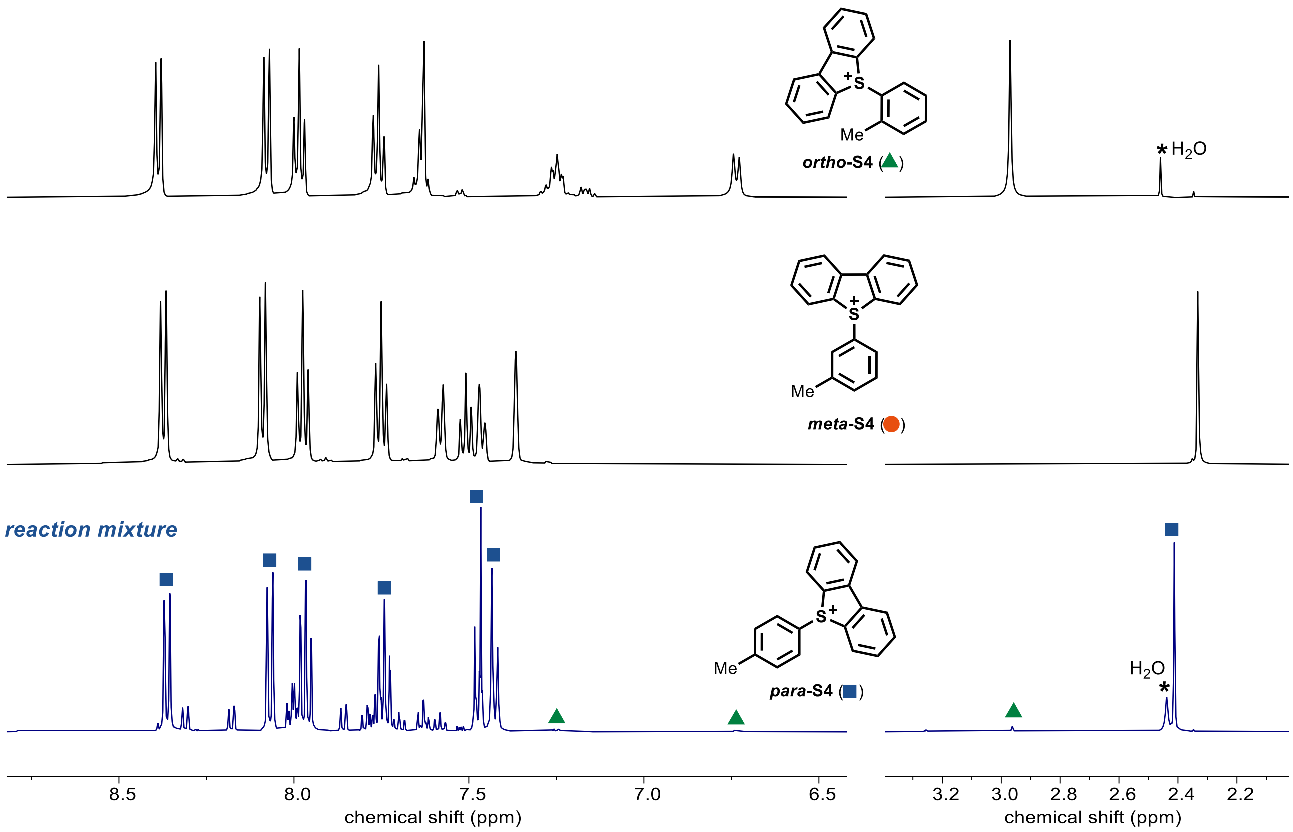


**Figure S22.** [1]H NMR of reaction mixture, in comparison to *ortho* and *meta* isomers of **S4**, $CD_3CN$, 500 MHz, 298 K.

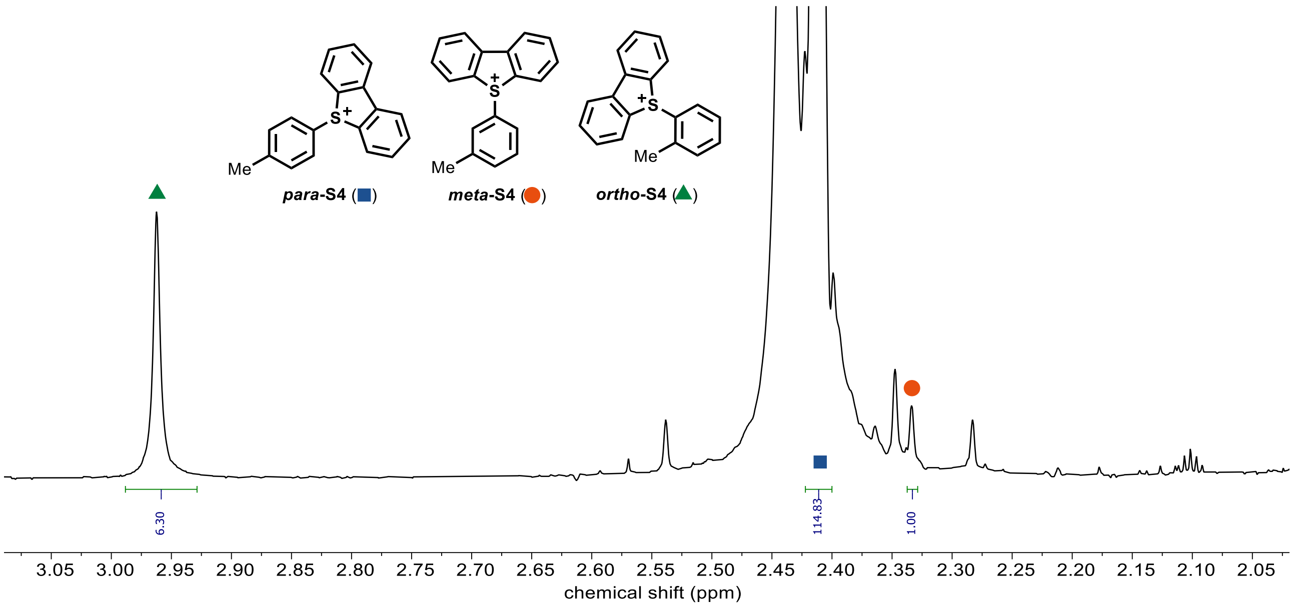


**Figure S23.** Determination of the selectivity in the reaction mixture, $CD_3CN$, 500 MHz, 298 K.

### Selectivity determination on C–H functionalization with diphenylsulfoxide/TFAA/$HBF_4OEt_2$

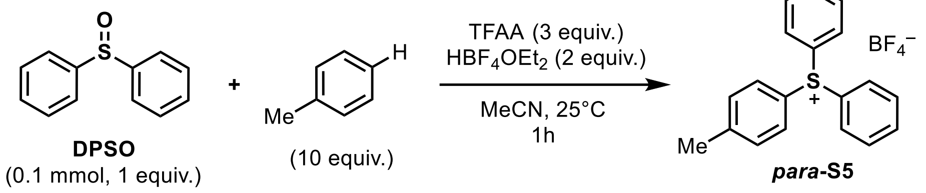


Under an ambient atmosphere, a 4 ml glass-vial equipped with a stir bar was charged with diphenylsulfoxide (20 mg, 0.10 mmol, 1.0 equiv.), dry MeCN (1 mL, c = 0.1 M) and toluene (106 µL, 1.00 mmol, 10.0 equiv.). TFAA (42 µL, 0.30 mmol, 3.0 equiv.) and then $HBF_4\cdot OEt_2$ (27 µL, 0.20 mmol, 2.0 equiv.) were subsequently added to the vial while stirring the reaction mixture. The vial was sealed with a screw-cap and the mixture was stirred at 25 °C for 1 h. The solution was diluted with 10 ml DCM and poured onto 10 ml of saturated aqueous $NaHCO_3$ solution in a separatory funnel. After the layers were separated, the DCM layer was washed with aqueous $NaBF_4$ solution (10 ml, 5 % w/w). The organic phase was dried over $Mg_2SO_4$, filtered, and the solvent was removed under reduced pressure. The resultant residue was dissolved in $CD_3CN$ (0.5 mL) and analyzed by $^1H$ NMR spectroscopy.

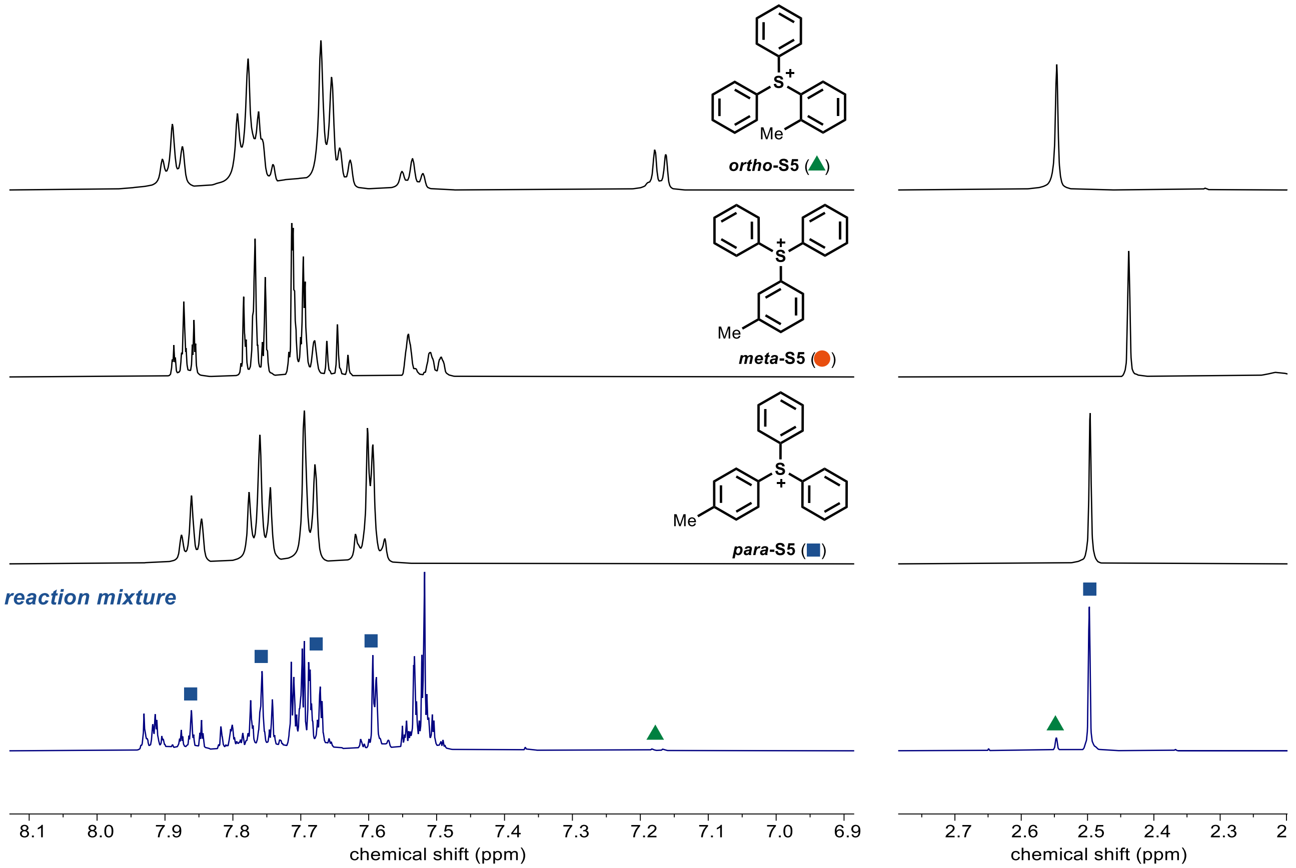


**Figure S24.** $^1H$ NMR of reaction mixture, in comparison to *ortho* and *meta* isomers of **S5**, $CD_3CN$, 500 MHz, 298 K.

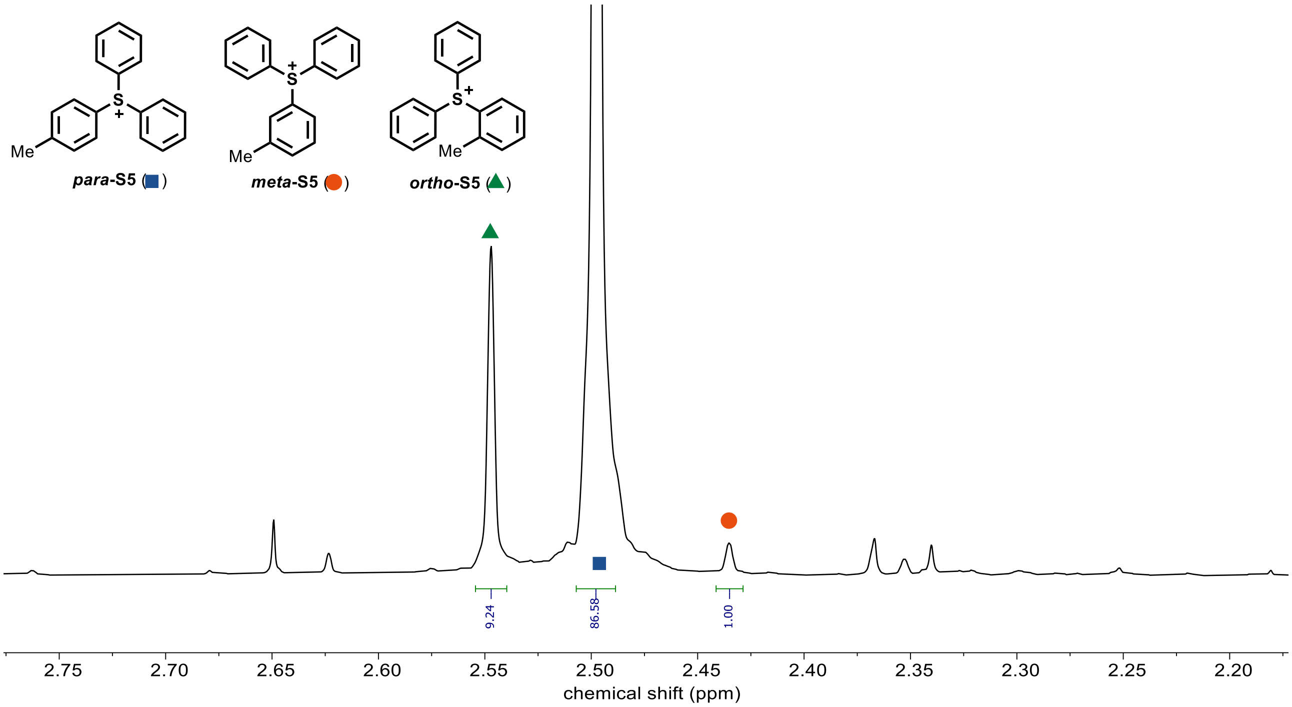


**Figure S25.** Determination of the selectivity in the reaction mixture, $CD_3CN$, 500 MHz, 298 K.

## Brown-Stock plot

As originally described by Brown and Stock,[4] a linear free energy relationship between the relative rate of *p*-Tol–H versus Ph–H and the relative rate of *p*-Tol–H versus *m*-Tol–H is generally observed for electrophilic aromatic substitution (EAS). We compared the selectivity-relationship data of thianthrenation[1] with those of the other EAS,[5-8] finding a relatively good fitting.

**Table S2.**

| reaction | $k_{p\text{-tol}}$ / $k_{benzene}$ | *p/m* in toluene |
|---|---|---|
| Thianthrenation (**TFTO**) | 11200 | 206 |
| Bromination | 2420 | 220 |
| Chlorination | 820 | 82 |
| Benzoylation | 831 | 83 |
| Acetylation | 749 | 54 |
| Nitration | 46 | 17 |
| Iodination | 64 | 12 |
| Alkylation | 10 | 1.8 |

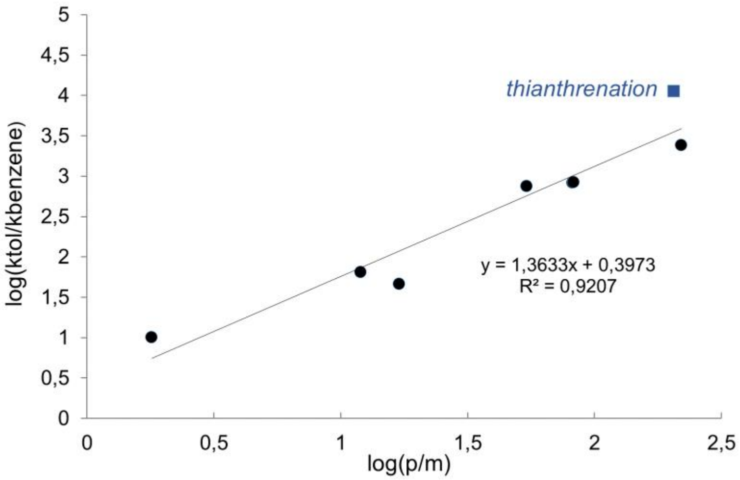


**Figure S26.** Stock-Brown analysis of EAS discussed in this manuscript. Data obtained from refs 1,5-8.

## Kinetic Isotope Effect (KIE) determinations

### KIE on toluene for thianthrene-S-oxide

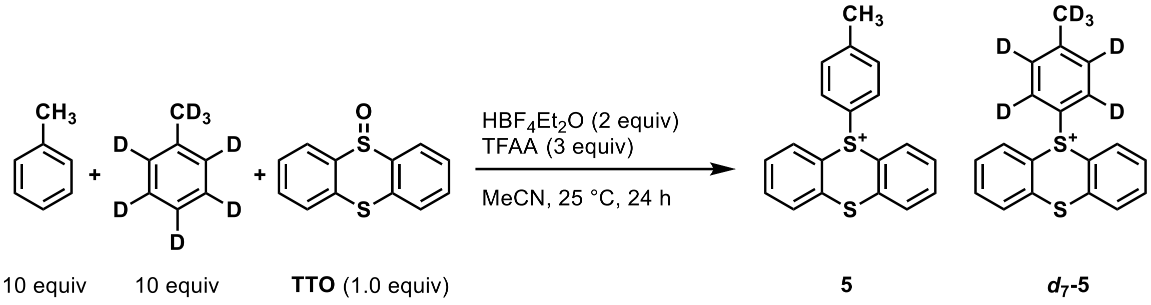


Under an ambient atmosphere, a 4 mL vial was charged with thianthrene-*S*-oxide (23.2 mg, 0.100 mmol, 1.00 equiv.), dry MeCN (1 mL, c = 0.1 M), toluene-$d_8$ (106 µL, 1.00 mmol, 10.0 equiv.) and toluene (106 µL, 1.00 mmol, 10.0 equiv.). Then $HBF_4{\cdot}OEt_2$ (27 µL, 0.20 mmol, 2.0 equiv.) followed by trifluoroacetic anhydride (42 µL, 0.30 mmol, 3.0 equiv.). The reaction mixture was stirred at 25 °C for 24 h, at which point, the solvent was removed via rotary evaporator, the residue was dissolved in dichloromethane, and loaded onto a silica gel column. The column was eluted with 20 mL of dichloromethane to separate all non-sulfonium salts, and then with a 20 mL of a dichloromethane/*i*-PrOH mixture (7:3) to wash the sulfonium salts from the column. The solution containing the mixture of sulfonium salts was concentrated via rotary evaporator and then analyzed by $^1H$ NMR spectroscopy and electrospray ionization mass spectrometry.

$k_H / k_D$ = 2.6 (determined from $^1H$ spectrum, Fig. S27)

$k_H / k_D$ = 2.6 (determined by ESI-MS)

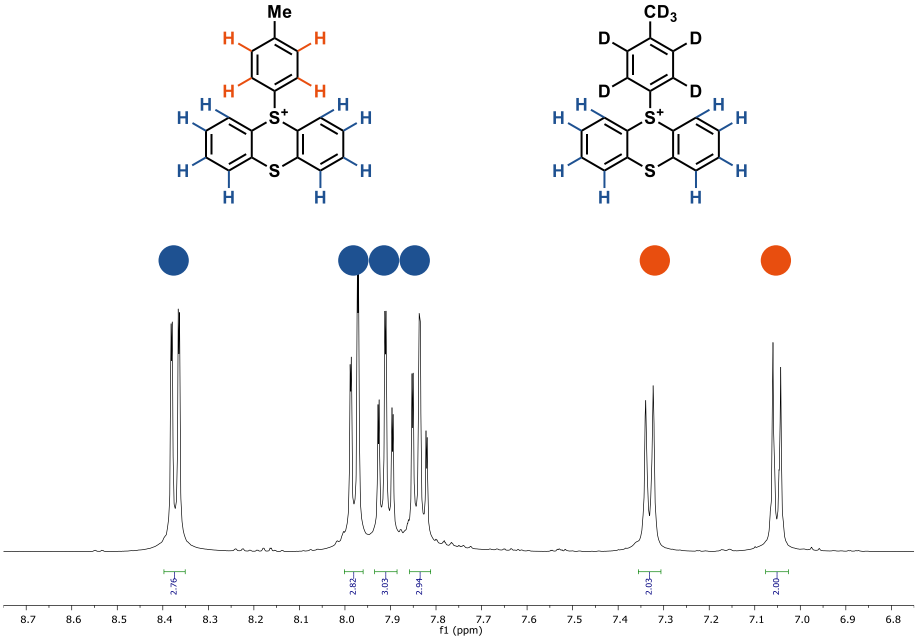


**Figure S27.** $^1H$ NMR of intermolecular competition experiment for the functionalization of toluene versus toluene-$d_8$ with thianthrene-*S*-oxide, $CD_3CN$, 500 MHz, 298 K.

## KIE on toluene for tetrafluorothianthrene-S-oxide

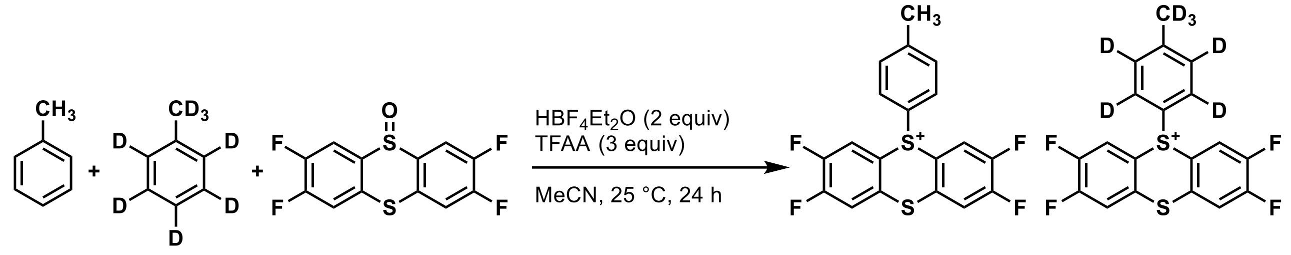


10 equiv 10 equiv **TFTO** (1.0 equiv) **S2** ***d*₇-S2**

Under an ambient atmosphere, a 4 mL vial was charged with tetrafluorothianthrene-*S*-oxide (30 mg, 0.10 mmol, 1.0 equiv.), dry MeCN (1 mL, c = 0.1 M), toluene-$d_8$ (106 µL, 1.00 mmol, 10.0 equiv.) and toluene (106 µL, 1.00 mmol, 10.0 equiv.). Then $HBF_4{\cdot}OEt_2$ (27 µL, 0.20 mmol, 2.0 equiv.) followed by trifluoroacetic anhydride (42 µL, 0.30 mmol, 3.0 equiv.). The reaction mixture was stirred at 25 °C for 24 h, at which point, the solvent was removed via rotary evaporator, the residue was dissolved in dichloromethane, and loaded onto a silica gel column. The column was eluted with 20 mL of dichloromethane to separate all non-sulfonium salts, and then with a 20 mL of a dichloromethane/*i*-PrOH mixture (7:3) to wash the sulfonium salts from the column. The solution containing the mixture of sulfonium salts was concentrated via rotary evaporator and then analyzed by $^1$H NMR spectroscopy and electrospray ionization mass spectrometry.

$k_H / k_D$ = 2.2 (determined from $^1$H spectrum, Fig. S28)

$k_H / k_D$ = 2.3 (determined by ESI-MS)

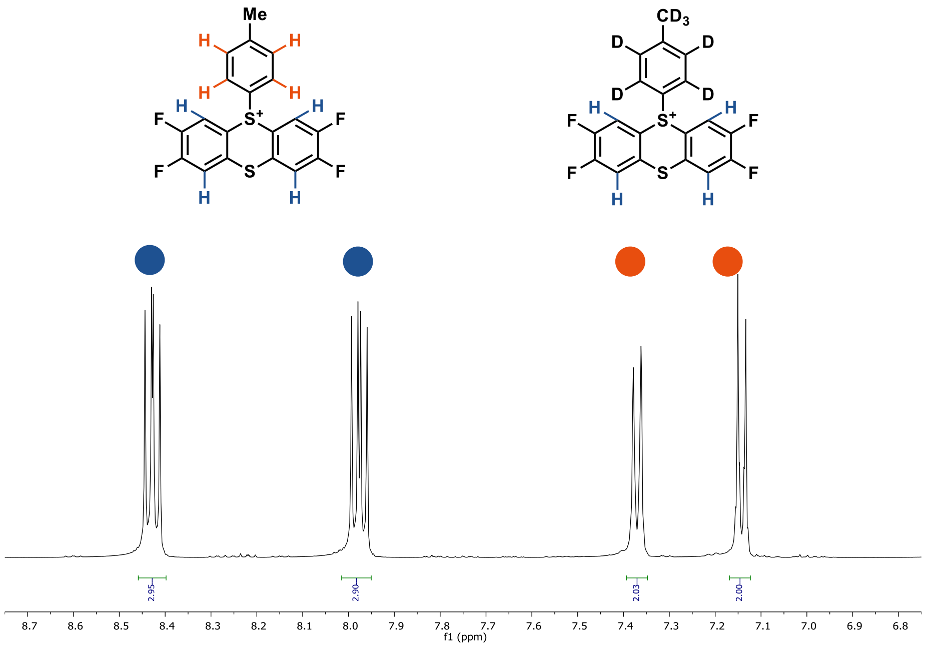


**Figure S28.** $^1$H NMR of intermolecular competition experiment for the functionalization of toluene versus toluene-$d_8$ with tetrafluorothianthrene-*S*-oxide, $CD_3CN$, 500 MHz, 298 K.

## KIE on toluene for phenoxtathiin-S-oxide

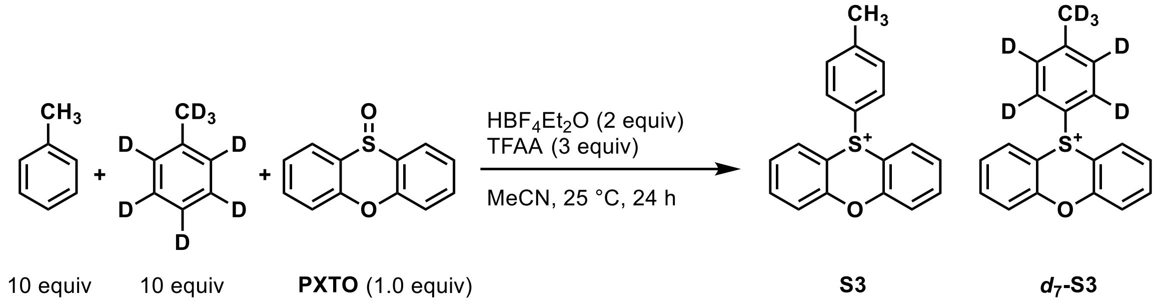


Under an ambient atmosphere, a 4 mL vial was charged with phenoxathiine-*S*-oxide (22 mg, 0.10 mmol, 1.0 equiv.), dry MeCN (1 mL, c = 0.1 M), toluene-$d_8$ (106 µL, 1.00 mmol, 10.0 equiv.) and toluene (106 µL, 1.00 mmol, 10.0 equiv.). Then $HBF_4·OEt_2$ (27 µL, 0.20 mmol, 2.0 equiv.) followed by trifluoroacetic anhydride (42 µL, 0.30 mmol, 3.0 equiv.). The reaction mixture was stirred at 25 °C for 24 h, at which point, the solvent was removed via rotary evaporator, the residue was dissolved in dichloromethane, and loaded onto a silica gel column. The column was eluted with 20 mL of dichloromethane to separate all non-sulfonium salts, and then with a 20 mL of a dichloromethane/*i*-PrOH mixture (7:3) to wash the sulfonium salts from the column. The solution containing the mixture of sulfonium salts was concentrated via rotary evaporator and then analyzed by $^1$H NMR spectroscopy and electrospray ionization mass spectrometry.

$k_H / k_D$ = 2.4 (determined from $^1$H spectrum, Fig. S29)

$k_H / k_D$ = 2.6 (determined by ESI-MS)

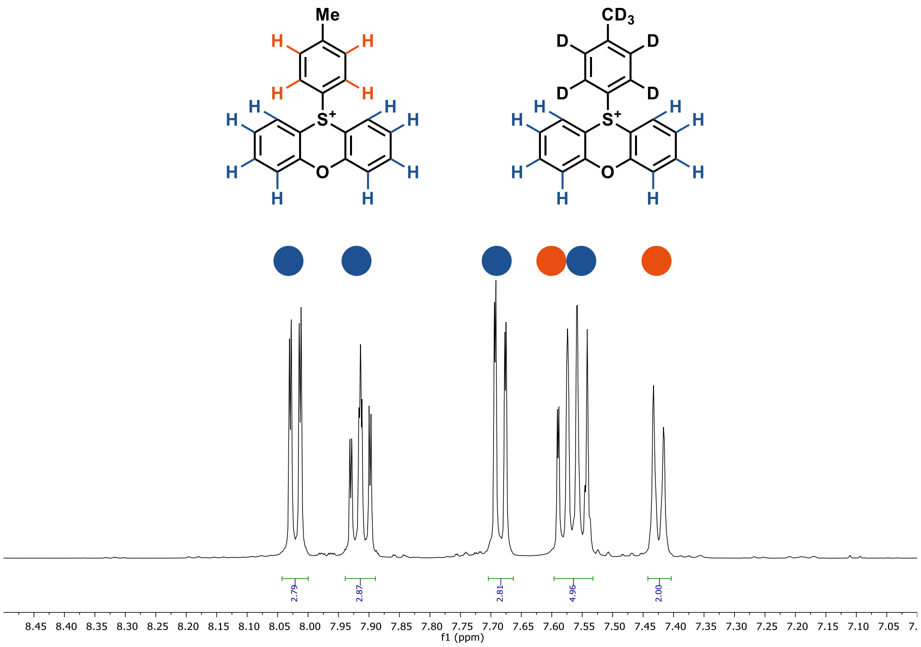


**Figure S29.** $^1$H NMR of intermolecular competition experiment for the functionalization of toluene versus toluene-$D_8$ with phenoxathiin-10-oxide, $CD_3CN$, 500 MHz, 298 K.

## KIE on toluene for dibenzothiophene-S-oxide

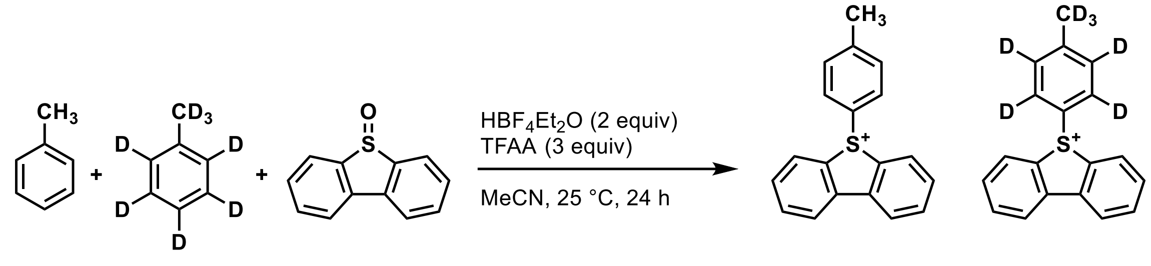


Under an ambient atmosphere, a 4 mL vial was charged with dibenzothiophene-*S*-oxide (20 mg, 0.10 mmol, 1.0 equiv.), dry MeCN (1 mL, c = 0.1 M), toluene-$d_8$ (106 µL, 1.00 mmol, 10.0 equiv.) and toluene (106 µL, 1.00 mmol, 10.0 equiv.). Then $HBF_4 \cdot OEt_2$ (27 µL, 0.20 mmol, 2.0 equiv.) followed by trifluoroacetic anhydride (42 µL, 0.30 mmol, 3.0 equiv.). The reaction mixture was stirred at 25 °C for 24 h, at which point, the solvent was removed via rotary evaporator, the residue was dissolved in dichloromethane, and loaded onto a silica gel column. The column was eluted with 20 mL of dichloromethane to separate all non-sulfonium salts, and then with a 20 mL of a dichloromethane/*i*-PrOH mixture (7:3) to wash the sulfonium salts from the column. The solution containing the mixture of sulfonium salts was concentrated via rotary evaporator and then analyzed by $^1$H NMR spectroscopy and electrospray ionization mass spectrometry.

$k_H / k_D$ = 1.2 (determined from $^1$H spectrum, Fig. S30)

$k_H / k_D$ = 1.3 (determined by ESI-MS)

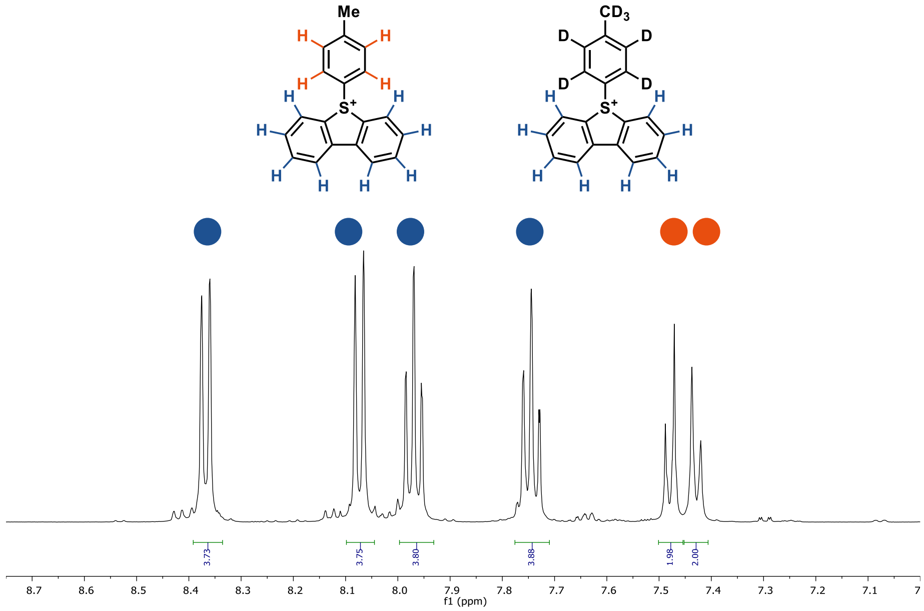


**Figure S30.** $^1$H NMR of intermolecular competition experiment for the functionalization of toluene versus toluene-$d_8$ with dibenzothiophene-*S*-oxide, $CD_3CN$, 500 MHz, 298 K.

### KIE on toluene for diphenylsulfoxide

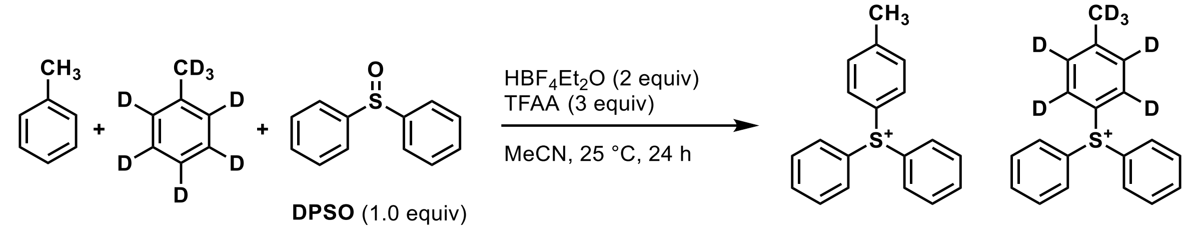


10 equiv 10 equiv **DPSO** (1.0 equiv) **S5** ***d*$_7$-S5**

Under an ambient atmosphere, a 4 mL vial was charged with diphenylsulfoxide (20 mg, 0.10 mmol, 1.00 equiv.), dry MeCN (1 mL, c = 0.1 M), toluene-*d*$_8$ (106 µL, 1.00 mmol, 10.0 equiv.) and toluene (106 µL, 1.00 mmol, 10.0 equiv.). Then $HBF_4{\cdot}OEt_2$ (27 µL, 0.2 mmol, 2.0 equiv.) followed by trifluoroacetic anhydride (42 µL, 0.3 mmol, 3.0 equiv.). The reaction mixture was stirred at 25 °C for 24 h, at which point, the solvent was removed via rotary evaporator, the residue was dissolved in dichloromethane, and loaded onto a silica gel column. The column was eluted with 20 mL of dichloromethane to separate all non-sulfonium salts, and then with a 20 mL of a dichloromethane/*i*-PrOH mixture (7:3) to wash the sulfonium salts from the column. The solution containing the mixture of sulfonium salts was concentrated via rotary evaporator and then analyzed by electrospray ionization mass spectrometry. KIE values could not be calculated accurately from $^1$H NMR spectrum due to the close proximity of the aromatic signals.

$k_H / k_D$ = 1.0 (determined by ESI-MS)

### Kinetic isotope effect on benzene for thianthrene-S-oxide under different conditions

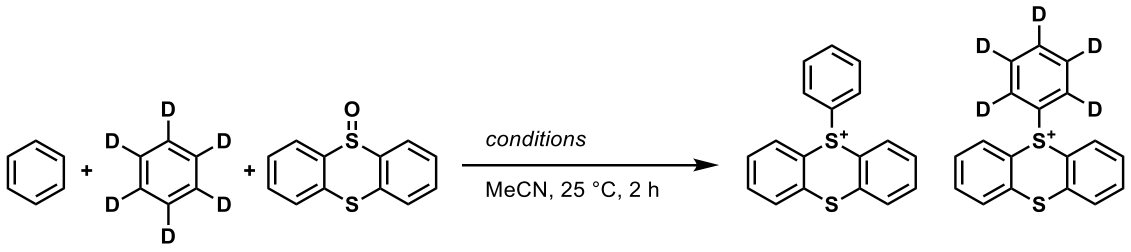


10 equiv 10 equiv **TTO** (1.0 equiv) **S6** ***d*$_7$-S6**

Under an ambient atmosphere, a 2 mL vial was charged with thianthrene-*S*-oxide (11.6 mg, 0.050 mmol, 1.00 equiv.), dry $CD_3CN$ (0.5 mL, c = 0.1 M), benzene-*d*$_6$ (44 µL, 0.50 mmol, 10 equiv.) and benzene (45 µL, 0.50 mmol, 10 equiv.) and base, if required. Then TFAA (21 µL, 0.15 mmol, 3.0 equiv.) and $HBF_4{\cdot}OEt_2$ (13 µL, 0.10 mmol, 2.0 equiv.) or TFAOTf (37 mg, 0.15 mmol, 3.0 equiv.) were added while stirring. The reaction mixture was stirred at 25 °C for 2 h and then analyzed by $^1$H NMR spectroscopy and electrospray ionization mass spectrometry.

**Table S3.**

| Entry | conditions | $k_H$ / $k_D$ [a] | $k_H$ / $k_D$ [b] |
|---|---|---|---|
| 1 | TFAA (3.0 equiv.) + $HBF_4OEt_2$ (2.0 equiv.) | **2.1** | **2.0** |
| 2 | TFAOTf (3.0 equiv.) | **1.6** | **1.5** |
| 3 | TFAOTf (3.0 equiv.) + $K_2CO_3$ (3.5 equiv.) | **1.8** | **1.9** |
| 4 | TFAOTf (3.0 equiv.) + 2,6-*t*-$Bu_2$py (3.5 equiv.) | **1.6** | **1.5** |

[a] Determined from $^1$H spectrum. [b] Determined by ESI-MS

## Mesitylene vs toluene competition experiments

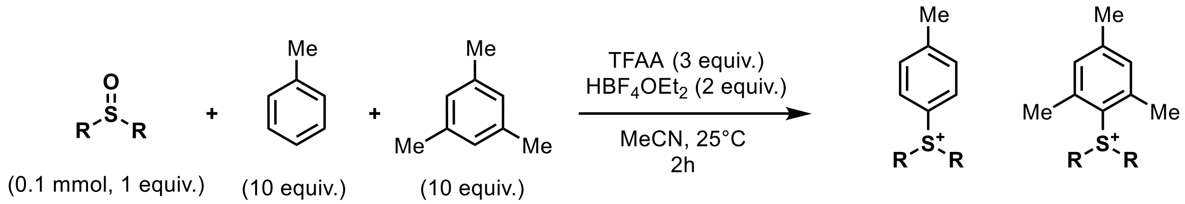


Under an ambient atmosphere, a 4 ml glass-vial equipped with a stir bar was charged with the sulfoxide (0.1 mmol, 1.0 equiv.), dry MeCN (1 mL, c = 0.1 M) and toluene (106 µL, 1.00 mmol, 10.0 equiv.). TFAA (42 µL, 0.30 mmol, 3.0 equiv.) and then $HBF_4 \cdot OEt_2$ (27 µL, 0.20 mmol, 2.0 equiv.) were subsequently added to the vial while stirring the reaction mixture. The vial was sealed with a screw-cap and the mixture was stirred at 25 °C for 2 h. The solution was diluted with 10 ml DCM and poured onto 10 ml of saturated aqueous $NaHCO_3$ solution in a separatory funnel. After the layers were separated, the DCM layer was washed with aqueous $NaBF_4$ solution (10 ml, 5 % w/w). The organic phase was dried over $Mg_2SO_4$, filtered, and the solvent was removed under reduced pressure. The resultant residue was dissolved in $CD_3CN$ (0.5 mL) and analyzed by $^1$H NMR spectroscopy.

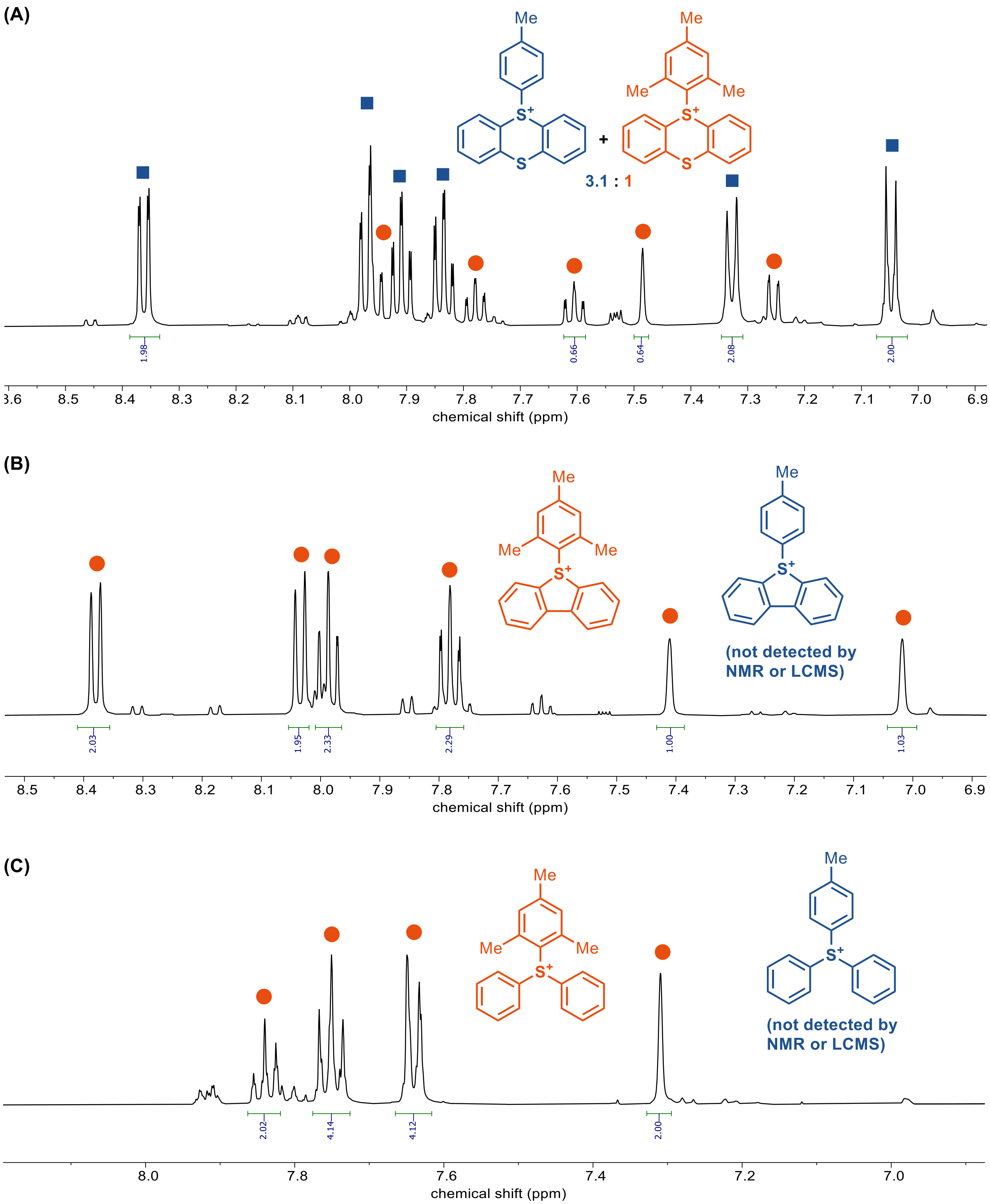


**Figure S31.** $^{1}H$ NMR of intermolecular competition experiments for the functionalization of toluene versus mesytilene with **TTO**, **DBTO** and **DPSO** in $CD_3CN$, 500 MHz, 298 K.

# Experiments with deuterium-labeled thianthrene-S-oxide

## Preparation of TTO-*d*$_2$

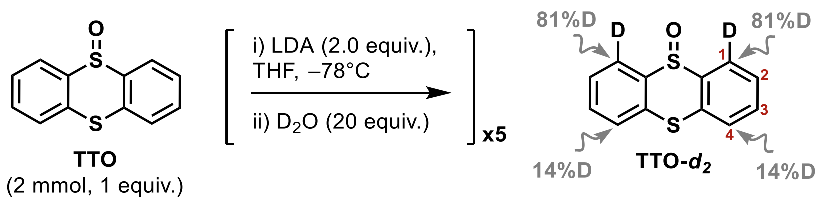


| iteration | %D in position 1 | %D in position 4 |
|---|---|---|
| 1 | **44%** | 3% |
| 2 | **57%** | 4% |
| 3 | **66%** | 7% |
| 4 | **76%** | 8% |
| 5 | **81%** | 14% |

Under nitrogen atmosphere, a flame-dried two-necked 50mL round bottom flask equipped with a stir bar was charged with thianthrene-S-oxide (466 mg, 2.00 mmol, 1.00 equiv.) and dry THF (8 mL, c = 0.25 M). The solution was cooled down to –78°C and LDA (2M in THF, 2.0 mL, 4.0 mmol, 2.0 equiv.) was added dropwise. The mixture was stirred at this temperature for 1 hour and then quenched with $D_2O$ (0.72 mL, 40 mmol, 20 equiv.) and warmed up to room temperature. The solution was diluted with 80 ml ethyl acetate and poured onto 60 ml of water in a separatory funnel. After the layers were separated, the organic layer was washed with brine, dried over $Mg_2SO_4$, filtered, and the solvent was removed under reduced pressure. This protocol was repeated 4 more times to achieve the desired level of deuteration (determined by $^1$H NMR). The obtained residue was purified by chromatography on silica gel eluting with DCM/EtOAc (100:0 to 9:1, v/v) to afford **TTO-*d*$_2$** (190 mg, 0.810 mmol, 41%) as a colorless solid.

**NMR Spectroscopy:**

**$^1$H NMR** (500 MHz, $CDCl_3$, 23 ºC, δ): 7.94 (dd, *J* = 7.7, 1.2 Hz, 0.4H), 7.64 (dd, *J* = 7.7, 1.2 Hz, 2H), 7.60 – 7.53 (m, 2H), 7.47 – 7.41 (m, 2H).

**$^{13}$C NMR** (125 MHz, $CDCl_3$, 23 ºC, δ): 141.4, 141.3, 129.8, 129.7, 129.0, 129.0, 128.4, 128.3, 124.5, 124.2 (t, $J_{C–D}$ = 25 Hz).

**HRMS-EI(m/z)** calc'd for $C_{12}H_6D_2OS_2$ $[M]^+$, 234.0136; found, 234.0137. deviation: 0.2 ppm.

### Thianthrenation of *tert*-butylbenzene with TTO-$d_2$

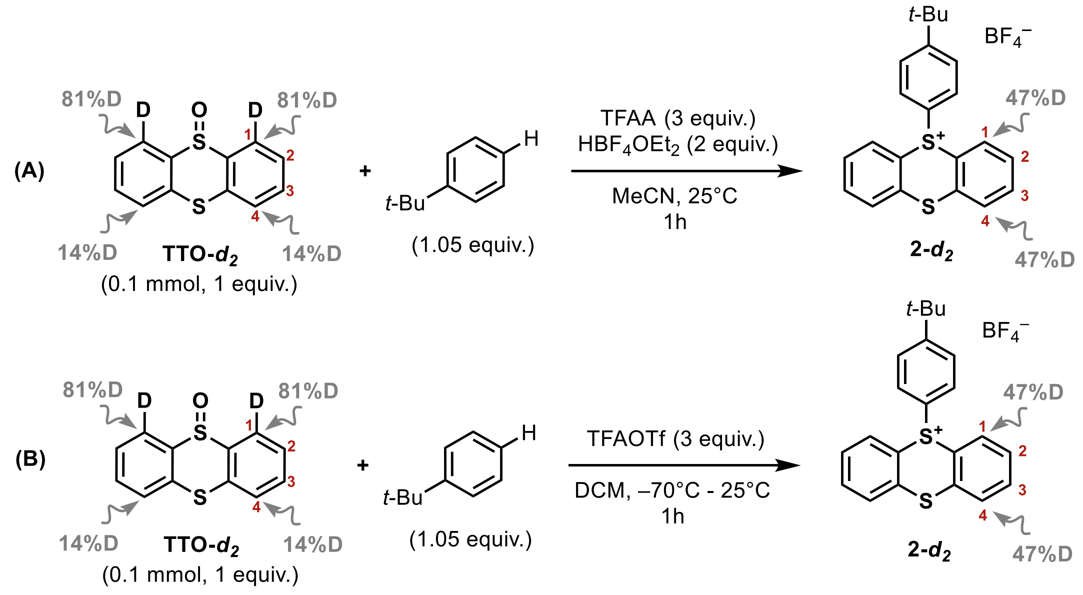


*Conditions A*: Under an ambient atmosphere, a 4 mL vial was charged with thianthrene-*S*-oxide-$d_2$ (23.4 mg, 0.100 mmol, 1.00 equiv.), dry MeCN (1 mL, c = 0.1 M) and *tert*-butylbenzene (16 µL, 0.105 mmol, 1.05 equiv.). Then $HBF_4 \cdot OEt_2$ (27 µL, 0.20 mmol, 2.0 equiv.) followed by trifluoroacetic anhydride (42 µL, 0.30 mmol, 3.0 equiv.). The reaction mixture was stirred at 25 °C for 1 h after which it was diluted with 10 ml DCM and poured onto 10 ml of saturated aqueous $NaHCO_3$ solution in a separatory funnel. After the layers were separated, the DCM layer was washed with aqueous $NaBF_4$ solution (10 ml, 5 % w/w). The organic phase was dried over $Mg_2SO_4$, filtered, and the solvent was removed under reduced pressure. The residue was purified by chromatography on silica gel eluting with DCM/*i*-PrOH (20:1, v/v) to afford **2-*d*₂** (40 mg, 0.092 mmol, 92 %) as a colorless solid.

*Conditions B*: Under an ambient atmosphere, a 10 mL round-bottom flask was charged with thianthrene-*S*-oxide-$d_2$ (23.4 mg, 0.100 mmol, 1.00 equiv.), dry DCM (1 mL, c = 0.1 M) and *tert*-butylbenzene (16 µL, 0.105 mmol, 1.05 equiv.). The mixture was cooled down to –78°C and then TFAOTf (49 mg, 0.20 mmol, 2.0 equiv.) was added. The reaction mixture was stirred at this temperature for 10 minutes and then 1 hout at 25 °C. It was diluted with 10 ml DCM and poured onto 10 ml of saturated aqueous $NaHCO_3$ solution in a separatory funnel. After the layers were separated, the DCM layer was washed with aqueous $NaBF_4$ solution (10 ml, 5 % w/w). The organic phase was dried over $Mg_2SO_4$, filtered, and the solvent was removed under reduced pressure. The residue was purified by chromatography on silica gel eluting with DCM/*i*-PrOH (20:1, v/v) to afford **2-$d_2$** (42 mg, 0.096 mmol, 96 %) as a colorless solid.

**NMR Spectroscopy:**

**$^1$H NMR** (500 MHz, $CDCl_3$, 23 ºC, δ): 8.49 (d, *J* = 7.8 Hz, 1H), 7.85 – 7.79 (m, 3H), 7.76 – 7.71 (m, 2H), 7.42 (d, *J* = 8.9 Hz, 2H), 7.11 (d, *J* = 8.9 Hz, 2H), 1.21 (s, 9H).

**$^{13}$C NMR** (125 MHz, $CDCl_3$, 23 ºC, δ): 157.4, 136.5 (m), 134.9 (d, *J* = 16 Hz), 130.4, 130.2 (d, *J* = 17

Hz), 128.0, 127.9, 120.4, 118.8 (d, $J$ = 15 Hz), 35.2, 30.9.

**$^{19}$F NMR** (470 MHz, $CDCl_3$, 23 ºC, δ): –151.53 (s), –151.58 (s).

**HRMS-ESI(m/z)** calc'd for $C_{22}H_{19}D_2S_2$ [M-BF4]$^+$, 351.1205; found, 351.1204. deviation: 0.1 ppm.

Discussion

With the aim of assess whether **$TT^+$-TFA** or **$TT^{2+}$** was operating as the reactive species in the thianthrenation of arenes with **TTO**, we studied the reactivity of deuterium-labeled thianthrene-S-oxide (**TTO-*d₂***) (Figure S30). We hypothesized that a reaction involving an attack of the arene to the sulfur in **$TT^+$-TFA** will result in product **I**, with the aryl group bounded exclusively to the sulfur that previously formed a S–O bond (pathway A: via acylated sulfoxide). Contrarily, a prior heterolytic dissociation of the S–O bond to generate **$TT^{2+}$** and trifluoroacetate would result in a statistical mixture of **I** and **II**, as a result of the symmetrical character of **$TT^{2+}$** (pathway B: via dication). Reactions with $TFAA/HBF_4OEt_2$ in MeCN at 25°C and TFAOTf in DCM at –70°C both resulted in a equimolar mixture of **I** and **II**, suggesting in principle that pathway B is operating.

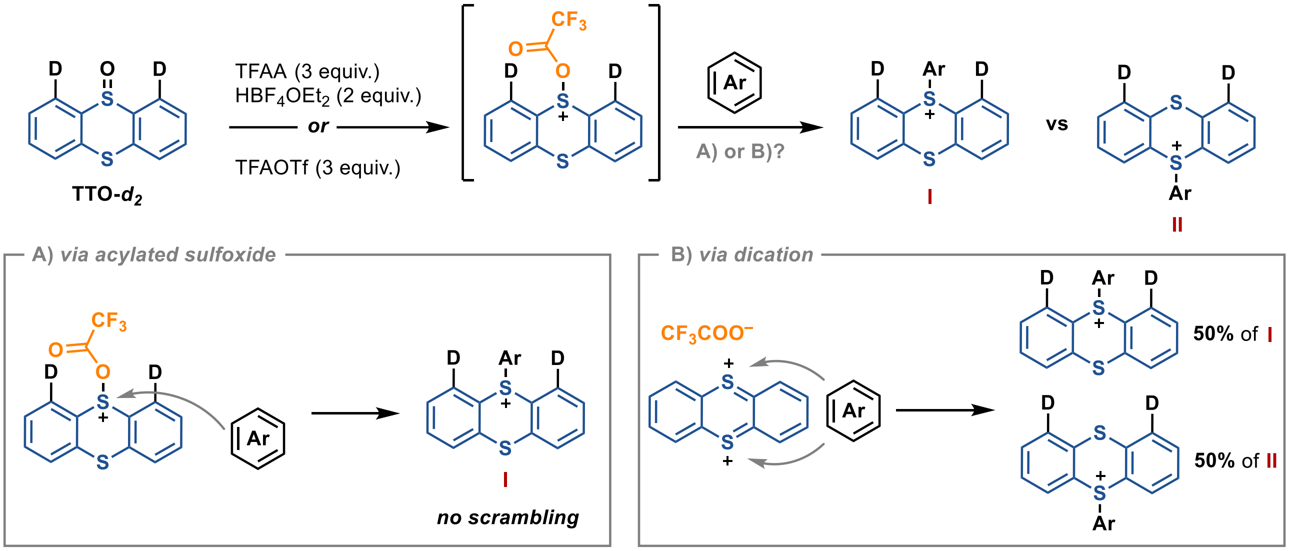


**Figure S32.** Mechanistic hypothesis for the interpretation of the results of thianthrenation using **TTO-$d_2$**.

In order evaluate an alternative mechanism conducting to the scrambling of the deuterated thianthrene, we considered an intramolecular exchange of the TFA moiety between the two sulfur atoms of thianthrene core (Figure S31). This pathway resulted to be more favourable (ΔG‡= +10.2 kcal/mol) than heterolytic S–O dissociation conducting to the dication (ΔG= +15.8 kcal/mol) by 5.6 kcal/mol. This intramolecular process should then occur considerably faster, accounting for the formation of **I**+**II** under the studied reaction conditions and therefore did not allow us to experimentally confirm the operation of **$TT^{2+}$** as the reactive species in aromatic thianthrenations.

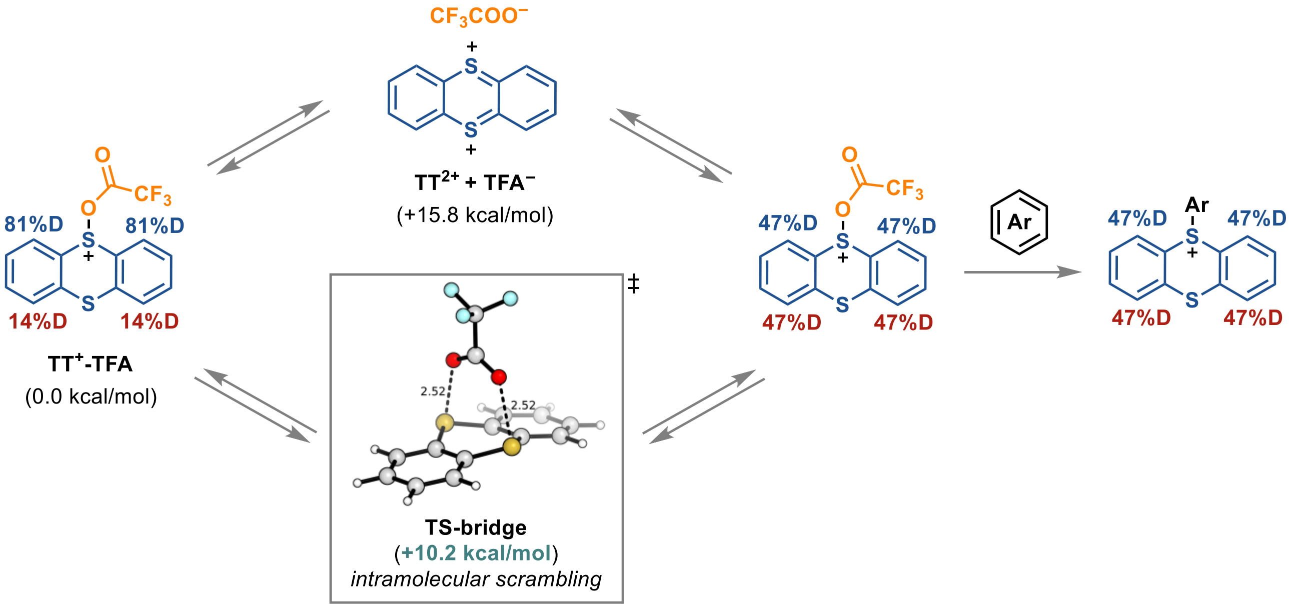


**Figure S33.** DFT studies on the deuterium scrambling during thianthrenation reactions using **TTO-*d*₂**.

## Cyclic voltammetry

Cyclic voltammetry was conducted using an Autolab PGSTAT204 potentiostat equipped with a glassy carbon working electrode, a Ag/AgCl reference electrode and a Pt counter electrode. Samples containing 10mM of the substrate and 0.1 M of tetrabutylammonium hexafluorophosphate were prepared in dry acetonitrile and degassed before measurements. Voltammograms were measured at 0.1 V/s scan rate. Potentials values were converted to SCE substracting 0.047 V according to tabulated conversions. Potentials for irreversible waves were estimated at half the maximum current, as previously described by Nicewicz.[9]

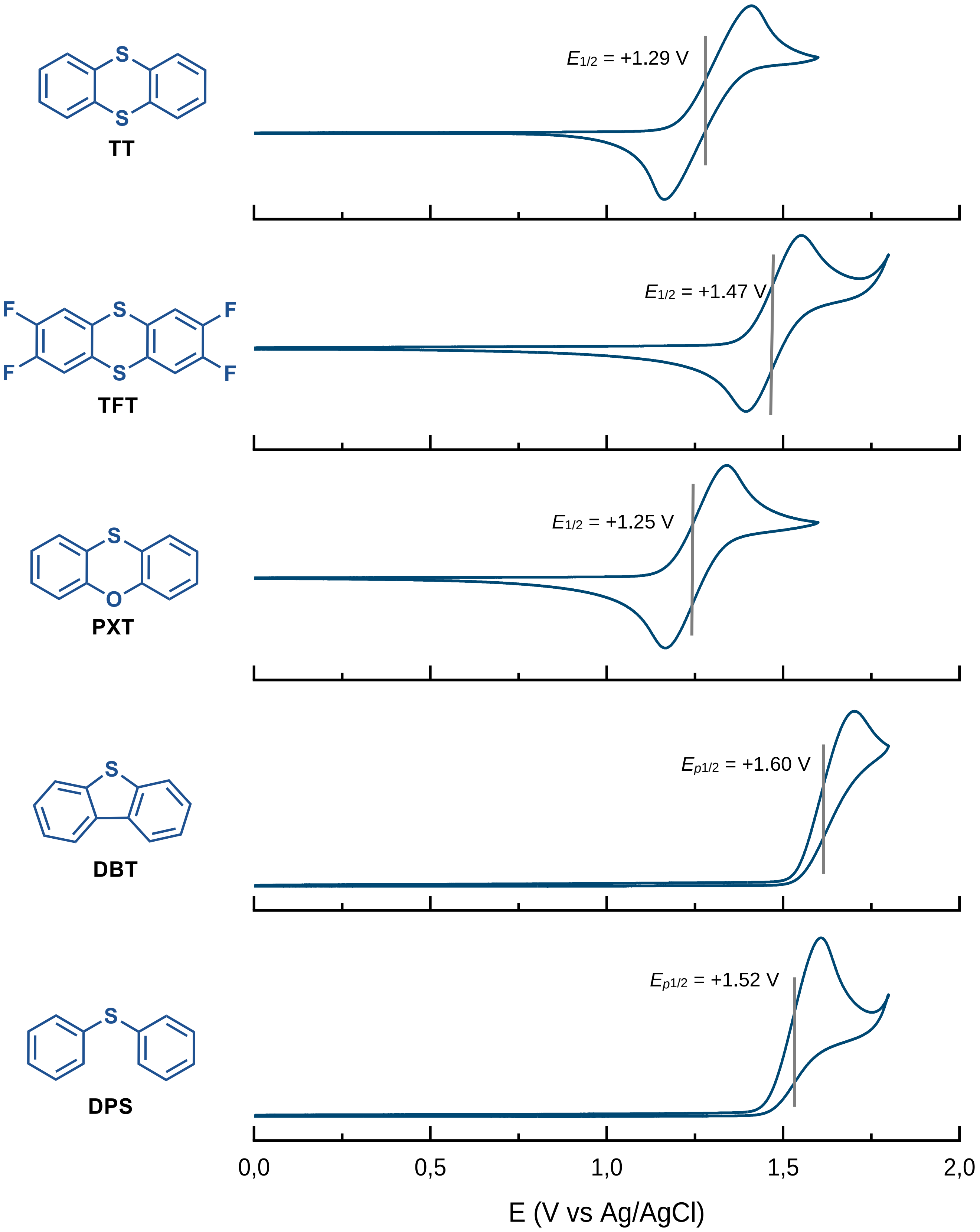


**Figure S34.** Cyclic voltammograms of different aryl sulfides. Y axis (intensity in mA) was omitted for clarity.

Discussion:

Similarly to that observed for **TT**, cyclic voltammograms in acetonitrile of **TFT** and **PTX** present reversible waves, suggesting a large chemical stability of the corresponding 1e-oxidized species. **TFT**$^{•+}$ and **PTX**$^{•+}$ can benefit from analogous conjugative effects described for **TT**$^{•+}$, which ought to contribute to the stability of these radical cations.[10] **DBT** presents an irreversible oxidation in acetonitrile. While some conjugation in the two benzenoid rings could be possible, studies on **DBT**$^{•+}$ have revealed a large spin density located at the sulfur atom,[11] resulting in a lower stabilization by delocalization in comparison with **TT**$^{•+}$. Finally, there are few reports studying **DPS**$^{•+}$,[10] which is described as a very reactive species with the spin density largely based on the sulfur atom. In line with its low stability, the electrochemical oxidation of **DPS** in acetonitrile was found irreversible.

## Open-circuit measurements

When a solution of toluene and **TTO** is stirred at 25 °C in acetonitrile, a constant potential of +0.55 V is required to maintain a current of 0 A between the reaction mixture and the electrode. The addition of $HBF_4•Et_2O$ increases the necessary potential to approximately +1.25 V (Figure S33, point 1), consistent with the reported value for **TT**$^{•+}$ of 1.26 V. Visibly, there is only a small amount of radical cation produced with $HBF_4•Et_2O$. When TFAA is added to the reaction mixture, a spike in the applied potential is observed (Figure S33, point 2), denoting the formation of a strong oxidant (E = +1.56 V ) which is rapidly consumed. Analysis of the reaction mixture by $^1$H NMR spectroscopy revealed 90% yield of aryl thianthrenium salt during this experiment.

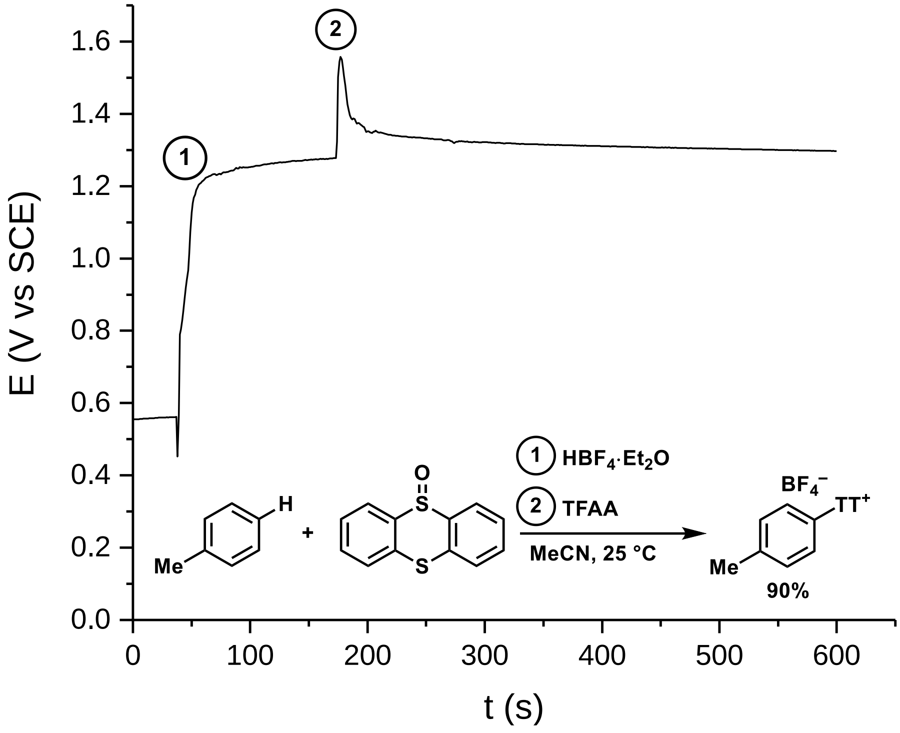


**Figure S35.** Open-circuit experiments on thianthrenation

# COMPUTATIONAL DATA

## Computational Methods

Density-functional theory (DFT) calculations were applied with the Gaussian 16 package[12].Geometry optimizations and frequency calculations were performed at ωB97X-D/6-31+G(d)-SMD(acetonitrile) the level of theory[13-14]. Normal vibrational mode analysis was carried out for all stationary points to confirm them as minima (zero imaginary frequencies) or transition structures (a single imaginary frequency), and to derive the thermochemical corrections for the enthalpies and free energies. Single-point energies were computed at ωB97X-D/6-311++G(d,p)-SMD(acetonitrile) level of theory using the optimized structures. Thermochemistries were corrected with the Head-Gordon and Grimme corrections using GoodVibes version 3.0.1[15], with quasiharmonic approximations to entropy[16] and enthalpy[17] and corrected for 298.15K.

## Intrinsic instability of $[ArH\text{-}TT]^{\bullet+}$

$[ArH\text{-}TT]^{\bullet+}$ cannot be located with spin and charge reset on optimized $[ArH\text{-}TT]^{2+}$ initial geometry. S-C bond scan shows electronic energy goes up as Toluene and $TT^{\bullet+}$ are brought together.

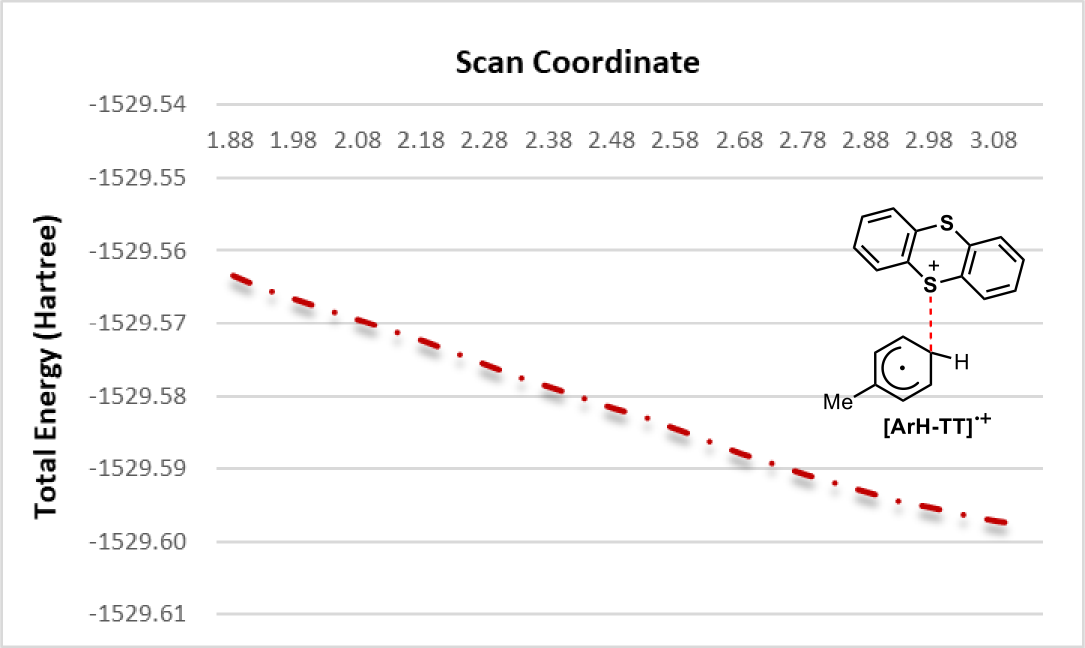


**Figure S36.** S-C bond scan of $[ArH\text{-}TT]^{\bullet+}$. Scanned bond is noted in red.

## Radical recombination vs Polar electrophilic addition

**$TT^{2+}$ → I** can proceed through single-electron transfer (SET) + radical recombination or polar electrophilic addition. The former pathway happens in barrierless manner with a free energy high point corresponding to the fragment aggression. (Left) The later pathway involves a TS with a 0.6 kcal/mol higher free energy (Right), which make it unfavorable. In the other way, the geometry change is basically identical in these two process, which means the polar electrophilic addition or TS-III can only exist in imaginary DFT calculations

with the restriction of the electron spin. The SET can happen in the thermodynamic favored manner before S-C distance is close enough to proceed the polar electrophilic addition.

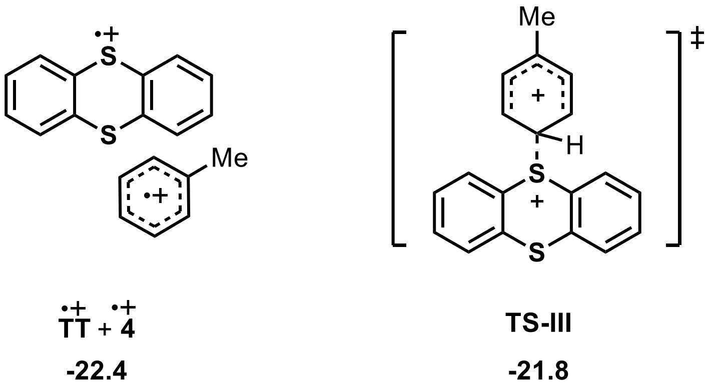


**Figure S37.** Key free energies of radical recombination vs polar electrophilic addition. Energy zero point is **TTO** (same as Figure 2)

Figure S35 does not include the final deprotonation part since the deprotonation but TFA⁻ involves a 3-component ion-pair complex. This kind of highly charged non-bonding interaction complex is hard to search the conformation and even optimize due to the limitation of the method and the computational power. We tried with CREST-NCI mode from XTB but fail with getting the final reasonable geometry. The TS - precomplex pair also has a question of ZPE and entropy, since we are optimizing on the electronic energy surface. The C-H bond is stretched in the TS and causes a reduction of the ZPE. This effect makes most of our optimized TSs have a lower enthalpy than the precomplex

## $TT^{2+}$ vs $TT^{+}$-TFA in basic condition

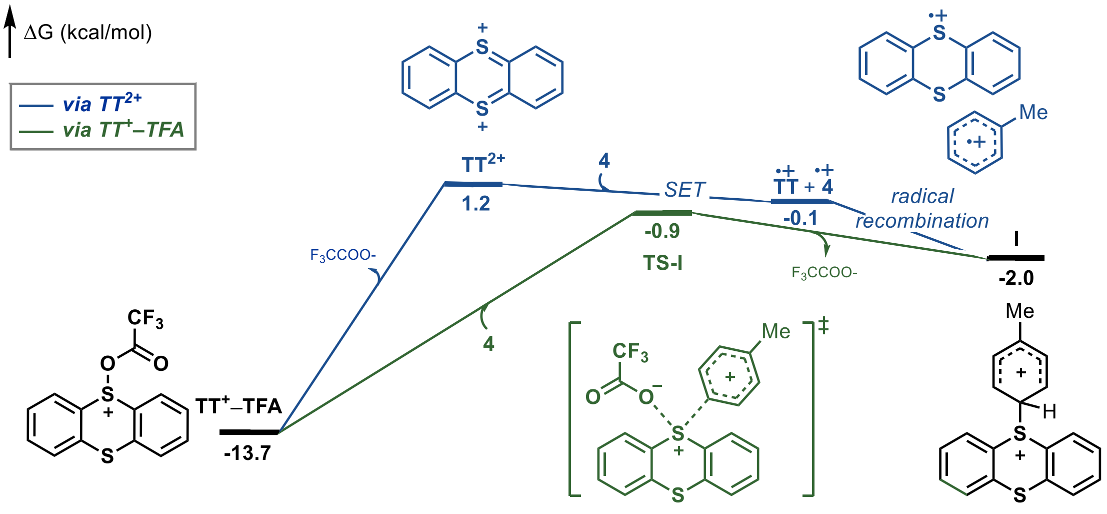


**Figure S38.** Energy profile from **TT⁺-TFA** to **I** in basic condition with TFAOTf.

## Benchmark with different density functionals

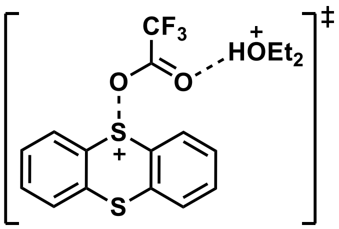


TS-II

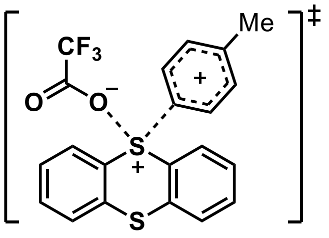


TS-I

| Methods | $\Delta\Delta G_{TS}{}^{a}$ (kcal/mol) |
|---|---|
| ωB97X-D/6-311++G(d,p) | 8.9 |
| M06-2X/6-311++G(d,p) | 7.5 |
| MN15/6-311++G(d,p) | 8.2 |
| PBE0-D3BJ/6-311++G(d,p) | 5.4 |
| B3LYP-D3BJ/6-311++G(d,p) | 6.6 |
| **(Optimization&Frequency: ωB97XD/6-31+G(d))** | |

[a]$G_{TS}$ is obtained by subtracting $\Delta G_{TS\text{-}II}$ from $\Delta G_{TS\text{-}I}$. Transition state structures are presented at the top. Optimization and frequency calculation are done at ωB97X-D/6-31+G(d). The SMD solvent model of MeCN is applied in both optimization and single point energy calculations.

## ωB97X-D geometries for all optimized compounds and transition states

**4 E= -271.546421 G= -271.444781**

C -1.903558 -0.001113 0.008690

C -1.200877 1.204281 0. 002159

C 0.194557 1.202689 -0.009394

C 0.913848 0.001378 -0.012391

C 0.196443 -1.201501 -0.009319

C -1.198576 -1.205575 0.002284

H -2.990437 -0.002059 0.015083

H -1.739513 2.148651 0.002883

H 0.734200 2.147628 -0.017283

H 0.737867 -2.145498 -0.017053

H -1.735536 -2.150901 0.003166

C 2.421881 0.000557 0.009019

H 2.827770 -0.857183 -0.537697

H 2.827755 0.915687 -0.434482

H 2.795588 -0.060621 1.039093

**5 E= -1529.199965 G= -1528.957406**

| | | | |
|---|---|---|---|
| C | -2.206531 | -3.626950 | 0.528222 |
| C | -1.924234 | -2.517095 | 1.321126 |
| C | -1.363340 | -1.374075 | 0.749659 |
| C | -1.097437 | -1.383996 | -0.624166 |
| C | -1.407232 | -2.477231 | -1.431621 |
| C | -1.962048 | -3.608809 | -0.844059 |
| C | -1.088139 | 1.389548 | -0.624714 |
| C | -1.353989 | 1.382310 | 0.749102 |
| C | -1.907434 | 2.529354 | 1.319803 |
| H | -2.135595 | 2.549709 | 2.381283 |
| C | -2.182999 | 3.640273 | 0.526031 |
| C | -1.939207 | 3.619393 | -0.846353 |
| C | -1.391486 | 2.483948 | -1.433049 |
| H | -2.152891 | -2.535133 | 2.382544 |
| H | -1.203032 | -2.448988 | -2.498065 |
| H | -1.187869 | 2.453371 | -2.499536 |
| S | -0.302763 | 0.000006 | -1.399186 |
| S | -0.992826 | 0.003099 | 1.802497 |
| C | 3.304569 | -1.214228 | 0.007211 |
| C | 1.994305 | -1.223581 | -0.465669 |
| C | 1.361238 | -0.006100 | -0.684766 |
| C | 2.003627 | 1.209338 | -0.464513 |
| C | 3.310959 | 1.190277 | 0.007486 |
| C | 3.980569 | -0.015501 | 0.252288 |
| H | 3.803701 | -2.162200 | 0.188683 |
| H | 1.505463 | 2.158078 | -0.640924 |
| H | 1.490609 | -2.169358 | -0.642400 |
| H | 3.818192 | 2.134054 | 0.190591 |
| C | 5.392490 | -0.007519 | 0.773179 |
| H | -2.172084 | 4.483345 | -1.459922 |
| H | -2.612397 | 4.524624 | 0.986511 |
| H | -2.199853 | -4.471930 | -1.456905 |
| H | -2.641533 | -4.508221 | 0.989338 |
| H | 6.037922 | 0.610912 | 0.139787 |
| H | 5.427465 | 0.413995 | 1.784623 |
| H | 5.809271 | -1.018058 | 0.810227 |

**CF3COOH E= -526.817188 G= -526.807215**

| | | | |
|---|---|---|---|
| O | 1.514749 | -1.040829 | -0.002892 |
| C | 0.946989 | 0.152595 | -0.005417 |
| O | 1.495656 | 1.221297 | -0.002360 |
| C | -0.594018 | -0.000397 | -0.001625 |
| F | -1.183282 | 1.189829 | -0.083637 |
| F | -1.003042 | -0.748652 | -1.035789 |
| F | -1.001367 | -0.598228 | 1.128149 |
| H | 2.488139 | -0.943463 | 0.005761 |

**Et2OH+ E= -234.083500 G= -233.959701**

| | | | |
|---|---|---|---|
| O | 0.000000 | -0.231859 | -0.073279 |
| C | 2.424060 | -0.402764 | -0.025172 |
| H | 2.424059 | -0.981889 | -0.952724 |
| H | 3.345086 | 0.187704 | 0.014120 |
| H | 2.422514 | -1.080674 | 0.835163 |
| C | 1.267606 | 0.557275 | 0.022329 |
| H | 1.220377 | 1.218867 | -0.841606 |
| H | 1.209730 | 1.126798 | 0.951700 |
| C | -1.267607 | 0.557276 | 0.022324 |
| H | -1.209730 | 1.126804 | 0.951691 |

H -1.220378 1.218861 -0.841616

C -2.424060 -0.402764 -0.025170

H -3.345086 0.187703 0.014118

H -2.424058 -0.981897 -0.952717

H -2.422514 -1.080668 0.835170

H -0.000001 -0.940874 0.607062

**Et2O E= -233.661814 G= -233.551257**

O -0.000000 0.269261 -0.000061

C 2.382941 0.405585 0.000050

H 2.384754 1.044907 0.890038

H 3.304594 -0.187031 -0.000116

H 2.384720 1.045341 -0.889627

C 1.179200 -0.513862 -0.000155

H 1.191616 -1.165455 0.888421

H 1.191671 -1.165143 -0.888959

C -1.179199 -0.513861 0.000266

H -1.191651 -1.165669 -0.888152

H -1.191637 -1.164928 0.889228

C -2.382941 0.405584 -0.000115

H -3.304594 -0.187032 0.000215

H -2.384694 1.045547 0.889414

H -2.384782 1.044701 -0.890251

**F3CCOO- E= -526.366161 G= -526.367816**

C -1.047870 0.011503 -0.007134

O -1.531689 -1.135710 -0.003563

C 0.522399 0.013134 -0.002540

F 1.012012 -0.546564 1.132628

F 1.076197 1.238958 -0.088111

F 1.032219 -0.705613 -1.031887

O -1.584689 1.132104 -0.003390

**H2O E= -76.441105 G= -76.434258**

O 0.000000 -0.000000 0.118003

H 0.000000 0.766163 -0.472011

H -0.000000 -0.766163 -0.472011

**I E= -1529.574361 G= -1529.32118**

C -2.384281 3.653391 -0.059284

C -2.447978 2.535907 -0.887442

C -1.733846 1.382820 -0.559780

C -0.960453 1.392605 0.607322

C -0.913886 2.498013 1.458642

C -1.631760 3.636366 1.114692

C -0.962069 -1.391810 0.607430

C -1.735452 -1.381240 -0.559674

C -2.450876 -2.533567 -0.887211

H -3.067095 -2.552906 -1.781128

C -2.388388 -3.651056 -0.058958

C -1.635785 -3.634802 1.114978

C -0.916636 -2.497222 1.458807

H -3.064139 2.555832 -1.781385

H -0.317996 2.471558 2.366138

H -0.320626 -2.471342 2.366248

S 0.030199 -0.000173 1.060435

S -1.821297 0.000809 -1.665608

C 3.497545 1.233699 -0.264497

C 2.143554 1.255508 -0.204550
C 1.370163 -0.000781 -0.253844
C 2.141571 -1.258193 -0.205707
C 3.495819 -1.238629 -0.266408
C 4.204790 -0.003208 -0.286116
H 4.060708 2.160776 -0.278942
H 1.602969 -2.201606 -0.187505
H 0.823543 -0.000262 -1.220926
H 1.606284 2.199670 -0.186123
H 4.057219 -2.166678 -0.281939
C 5.678565 -0.001506 -0.358257
H -1.607304 -4.504544 1.762582
H -2.952244 -4.538830 -0.327668
H -1.602371 4.506112 1.762249
H -2.947148 4.541766 -0.328084
H 5.944654 0.054588 -1.426942
H 6.107045 0.880075 0.125204
H 6.110483 -0.922148 0.040124

**m-2 E= -1529.199888 G= -1528.957526**

C -4.138667 -1.260939 0.540536
C -3.187112 -0.604956 1.318034
C -1.984970 -0.186708 0.745741
C -1.772445 -0.446949 -0.612723
C -2.732963 -1.071624 -1.406739
C -3.923048 -1.485429 -0.818257
C 0.126478 1.571794 -0.683140
C -0.097163 1.818564 0.675991
C 0.268604 3.063539 1.189668
H 0.096150 3.288383 2.238021
C 0.837111 4.022744 0.354761
C 1.024284 3.771004 -1.003495
C 0.662383 2.537454 -1.533598
H -3.385124 -0.405389 2.366971
H -2.547388 -1.245968 -2.462585
H 0.808416 2.322693 -2.588203
S -0.230927 -0.019670 -1.380896
S -0.790053 0.616827 1.779684
C 1.555059 -3.284690 0.218489
C 0.592470 -2.432907 -0.321791
C 0.968030 -1.121487 -0.582507
C 2.253664 -0.643319 -0.347179
C 3.211033 -1.501928 0.197275
C 2.842643 -2.824427 0.474883
H 1.289203 -4.313605 0.440888
H 2.519810 0.387612 -0.564898
H -0.413832 -2.792040 -0.514832
H 1.452207 4.529763 -1.650244
H 1.117220 4.985559 0.770854
H -4.676467 -1.982294 -1.420424
H -5.067725 -1.581362 1.001811
H 3.579437 -3.502375 0.898645
C 4.604836 -1.014000 0.491411
H 4.757578 0.003405 0.120072
H 4.793231 -1.015524 1.571266
H 5.351603 -1.667786 0.027887

**m-I E= -1529.565904 G= -1529.311864**

| | | | |
|---|---|---|---|
| C | 3.888143 | -2.137186 | -0.124858 |
| C | 3.368019 | -1.132838 | -0.937117 |
| C | 2.179586 | -0.494511 | -0.581068 |
| C | 1.538702 | -0.896845 | 0.597246 |
| C | 2.067046 | -1.883772 | 1.431448 |
| C | 3.251035 | -2.507377 | 1.059234 |
| C | 0.153958 | 1.516709 | 0.643172 |
| C | 0.802582 | 1.899827 | -0.537203 |
| C | 0.843360 | 3.256879 | -0.857977 |
| H | 1.346585 | 3.584666 | -1.762566 |
| C | 0.255167 | 4.190008 | -0.008449 |
| C | -0.361955 | 3.794180 | 1.178070 |
| C | -0.414911 | 2.447556 | 1.514555 |
| H | 3.893891 | -0.836807 | -1.839895 |
| H | 1.556904 | -2.165816 | 2.347801 |
| H | -0.900065 | 2.123688 | 2.430730 |
| S | -0.004904 | -0.186945 | 1.083660 |
| S | 1.534029 | 0.748139 | -1.667056 |
| C | -2.435741 | -2.939676 | -0.320672 |
| C | -1.232853 | -2.308204 | -0.206365 |
| C | -1.183891 | -0.839528 | -0.224181 |
| C | -2.458885 | -0.107646 | -0.192094 |
| C | -3.654997 | -0.762447 | -0.298138 |
| C | -3.620313 | -2.179151 | -0.349539 |
| H | -2.490776 | -4.021126 | -0.365813 |
| H | -2.428471 | 0.978581 | -0.151214 |
| H | -0.702099 | -0.570090 | -1.195176 |
| H | -0.303801 | -2.870464 | -0.171097 |
| H | -0.802684 | 4.530939 | 1.841035 |
| H | 0.297720 | 5.242313 | -0.271104 |
| H | 3.673299 | -3.279934 | 1.692849 |
| H | 4.814180 | -2.623455 | -0.415092 |
| H | -4.568057 | -2.709922 | -0.404302 |
| C | -4.970838 | -0.042400 | -0.342292 |
| H | -5.616017 | -0.387042 | 0.472474 |
| H | -4.831800 | 1.037409 | -0.250720 |
| H | -5.484097 | -0.253510 | -1.286215 |

**o-2 E= -1529.198106 G= -1528.953991**

| | | | |
|---|---|---|---|
| C | -1.487966 | 3.818982 | 0.606813 |
| C | -0.628896 | 3.008540 | 1.343490 |
| C | -0.164317 | 1.808103 | 0.802720 |
| C | -0.596904 | 1.442838 | -0.475523 |
| C | -1.416781 | 2.273223 | -1.238956 |
| C | -1.867817 | 3.466390 | -0.687700 |
| C | 1.599033 | -0.214948 | -0.758524 |
| C | 2.039232 | 0.192082 | 0.507337 |
| C | 3.391964 | 0.039611 | 0.815463 |
| H | 3.764244 | 0.355679 | 1.785367 |
| C | 4.265836 | -0.504097 | -0.122832 |
| C | 3.815307 | -0.882584 | -1.386443 |
| C | 2.472487 | -0.733171 | -1.713731 |
| H | -0.300787 | 3.318533 | 2.331191 |
| H | -1.714343 | 1.981094 | -2.241653 |
| H | 2.105202 | -1.027902 | -2.692287 |
| S | -0.113443 | -0.108100 | -1.206261 |
| S | 0.958180 | 0.828286 | 1.761044 |
| C | -0.802128 | -3.349608 | 1.164245 |

| | | | |
|---|---|---|---|
| C | -0.165099 | -2.315689 | 0.487584 |
| C | -0.932674 | -1.409459 | -0.241913 |
| C | -2.327634 | -1.514540 | -0.356902 |
| C | -2.931349 | -2.563579 | 0.345221 |
| C | -2.188149 | -3.466360 | 1.098912 |
| H | 0.915583 | -2.237904 | 0.530228 |
| H | -4.010558 | -2.673290 | 0.282873 |
| H | -2.692773 | -4.270838 | 1.625629 |
| H | -0.210811 | -4.057798 | 1.735920 |
| C | -3.176083 | -0.574368 | -1.170049 |
| H | 4.504709 | -1.292764 | -2.116838 |
| H | 5.314601 | -0.615675 | 0.134389 |
| H | -2.515919 | 4.115305 | -1.267335 |
| H | -1.840673 | 4.749149 | 1.041409 |
| H | -4.155030 | -1.023142 | -1.356978 |
| H | -3.338236 | 0.370615 | -0.639784 |
| H | -2.729371 | -0.342245 | -2.143941 |

**o-I E= -1529.570925 G= -1529.315597**

| | | | |
|---|---|---|---|
| C | -2.199539 | 3.062386 | -0.788496 |
| C | -1.070952 | 2.951082 | 0.014153 |
| C | -0.292076 | 1.790367 | -0.021108 |
| C | -0.686056 | 0.755175 | -0.879187 |
| C | -1.805041 | 0.877159 | -1.712975 |
| C | -2.564165 | 2.035248 | -1.660636 |
| C | 1.815764 | -0.480134 | -0.519546 |
| C | 2.204879 | 0.589899 | 0.296783 |
| C | 3.564958 | 0.734067 | 0.586805 |
| H | 3.900032 | 1.560428 | 1.206737 |
| C | 4.490251 | -0.167284 | 0.074731 |
| C | 4.088725 | -1.219467 | -0.750855 |
| C | 2.746689 | -1.377349 | -1.057529 |
| H | -0.782810 | 3.774460 | 0.661208 |
| H | -2.079815 | 0.071951 | -2.387698 |
| H | 2.420648 | -2.188169 | -1.703093 |
| S | 0.142458 | -0.796055 | -0.954944 |
| S | 1.106136 | 1.744564 | 1.049090 |
| C | -1.424728 | -0.581529 | 2.457167 |
| C | -0.373948 | -1.112080 | 1.777640 |
| C | -0.591477 | -1.817774 | 0.500872 |
| C | -1.994201 | -2.091948 | 0.079360 |
| C | -3.011578 | -1.520539 | 0.801394 |
| C | -2.729972 | -0.743527 | 1.936194 |
| H | 0.027380 | -2.723964 | 0.436650 |
| H | 0.640878 | -1.056584 | 2.157434 |
| H | -4.042927 | -1.677457 | 0.503575 |
| H | -3.558725 | -0.275494 | 2.459861 |
| H | -1.276951 | -0.042532 | 3.385760 |
| C | -2.239759 | -2.920008 | -1.129990 |
| H | 4.818964 | -1.909992 | -1.158661 |
| H | 5.541306 | -0.035611 | 0.311533 |
| H | -3.434996 | 2.136579 | -2.299174 |
| H | -2.789853 | 3.972160 | -0.745973 |
| H | -1.486824 | -3.706651 | -1.237821 |
| H | -3.239052 | -3.360037 | -1.089008 |
| H | -2.201417 | -2.297708 | -2.036315 |

**p-2 E= -1529.199966 G= -1528.957406**

| | | | |
|---|---|---|---|
| C | -2.206531 | -3.626950 | 0.528222 |
| C | -1.924234 | -2.517095 | 1.321126 |
| C | -1.363340 | -1.374075 | 0.749659 |
| C | -1.097437 | -1.383996 | -0.624166 |
| C | -1.407232 | -2.477231 | -1.431621 |
| C | -1.962048 | -3.608809 | -0.844059 |
| C | -1.088139 | 1.389548 | -0.624714 |
| C | -1.353989 | 1.382310 | 0.749102 |
| C | -1.907434 | 2.529354 | 1.319803 |
| H | -2.135595 | 2.549709 | 2.381283 |
| C | -2.182999 | 3.640273 | 0.526031 |
| C | -1.939207 | 3.619393 | -0.846353 |
| C | -1.391486 | 2.483948 | -1.433049 |
| H | -2.152891 | -2.535133 | 2.382544 |
| H | -1.203032 | -2.448988 | -2.498065 |
| H | -1.187869 | 2.453371 | -2.499536 |
| S | -0.302763 | 0.000006 | -1.399186 |
| S | -0.992826 | 0.003099 | 1.802497 |
| C | 3.304569 | -1.214228 | 0.007211 |
| C | 1.994305 | -1.223581 | -0.465669 |
| C | 1.361238 | -0.006100 | -0.684766 |
| C | 2.003627 | 1.209338 | -0.464513 |
| C | 3.310959 | 1.190277 | 0.007486 |
| C | 3.980569 | -0.015501 | 0.252288 |
| H | 3.803701 | -2.162200 | 0.188683 |
| H | 1.505463 | 2.158078 | -0.640924 |
| H | 1.490609 | -2.169358 | -0.642400 |
| H | 3.818192 | 2.134054 | 0.190591 |
| C | 5.392490 | -0.007519 | 0.773179 |
| H | -2.172084 | 4.483345 | -1.459922 |
| H | -2.612397 | 4.524624 | 0.986511 |
| H | -2.199853 | -4.471930 | -1.456905 |
| H | -2.641533 | -4.508221 | 0.989338 |
| H | 6.037922 | 0.610912 | 0.139787 |
| H | 5.427465 | 0.413995 | 1.784623 |
| H | 5.809271 | -1.018058 | 0.810227 |

**p-I E= -1529.574361 G= -1529.321180**

| | | | |
|---|---|---|---|
| C | -2.384281 | 3.653391 | -0.059284 |
| C | -2.447978 | 2.535907 | -0.887442 |
| C | -1.733846 | 1.382820 | -0.559780 |
| C | -0.960453 | 1.392605 | 0.607322 |
| C | -0.913886 | 2.498013 | 1.458642 |
| C | -1.631760 | 3.636366 | 1.114692 |
| C | -0.962069 | -1.391810 | 0.607430 |
| C | -1.735452 | -1.381240 | -0.559674 |
| C | -2.450876 | -2.533567 | -0.887211 |
| H | -3.067095 | -2.552906 | -1.781128 |
| C | -2.388388 | -3.651056 | -0.058958 |
| C | -1.635785 | -3.634802 | 1.114978 |
| C | -0.916636 | -2.497222 | 1.458807 |
| H | -3.064139 | 2.555832 | -1.781385 |
| H | -0.317996 | 2.471558 | 2.366138 |
| H | -0.320626 | -2.471342 | 2.366248 |
| S | 0.030199 | -0.000173 | 1.060435 |
| S | -1.821297 | 0.000809 | -1.665608 |
| C | 3.497545 | 1.233699 | -0.264497 |
| C | 2.143554 | 1.255508 | -0.204550 |

| | | | |
|---|---|---|---|
| C | 1.370163 | -0.000781 | -0.253844 |
| C | 2.141571 | -1.258193 | -0.205707 |
| C | 3.495819 | -1.238629 | -0.266408 |
| C | 4.204790 | -0.003208 | -0.286116 |
| H | 4.060708 | 2.160776 | -0.278942 |
| H | 1.602969 | -2.201606 | -0.187505 |
| H | 0.823543 | -0.000262 | -1.220926 |
| H | 1.606284 | 2.199670 | -0.186123 |
| H | 4.057219 | -2.166678 | -0.281939 |
| C | 5.678565 | -0.001506 | -0.358257 |
| H | -1.607304 | -4.504544 | 1.762582 |
| H | -2.952244 | -4.538830 | -0.327668 |
| H | -1.602371 | 4.506112 | 1.762249 |
| H | -2.947148 | 4.541766 | -0.328084 |
| H | 5.944654 | 0.054588 | -1.426942 |
| H | 6.107045 | 0.880075 | 0.125204 |
| H | 6.110483 | -0.922148 | 0.040124 |

**TFAA E= -977.162681 G= -977.147683**

| | | | |
|---|---|---|---|
| O | 1.398821 | 1.907923 | -0.000372 |
| C | 1.201136 | 0.737866 | -0.000188 |
| O | 0.000001 | 0.088033 | 0.000045 |
| C | 2.313050 | -0.342613 | 0.000043 |
| F | 2.206176 | -1.117249 | 1.084829 |
| F | 3.507406 | 0.236604 | -0.000225 |
| F | 2.206014 | -1.117916 | -1.084250 |
| C | -1.201135 | 0.737865 | 0.000147 |
| O | -1.398820 | 1.907923 | -0.000080 |
| C | -2.313050 | -0.342613 | 0.000014 |
| F | -3.507406 | 0.236606 | -0.000620 |
| F | -2.205687 | -1.117934 | -1.084230 |
| F | -2.206505 | -1.117231 | 1.084848 |

**TFAOTf E= -1412.391577 G= -1412.377863**

| | | | |
|---|---|---|---|
| O | -1.285586 | 1.469549 | 1.286016 |
| O | 0.276624 | -0.045554 | -0.016947 |
| C | -2.215659 | -0.728392 | -0.004029 |
| F | -1.987681 | -1.406383 | -1.112676 |
| F | -3.473581 | -0.321569 | 0.036897 |
| F | -1.935563 | -1.463322 | 1.055335 |
| C | 1.458434 | 0.625707 | -0.018544 |
| O | 1.599775 | 1.807012 | -0.021928 |
| C | 2.613390 | -0.407632 | -0.001304 |
| F | 3.777152 | 0.222278 | -0.092725 |
| F | 2.581143 | -1.104303 | 1.139026 |
| F | 2.494884 | -1.259467 | -1.022776 |
| S | -1.154397 | 0.823216 | 0.007935 |
| O | -1.312543 | 1.504661 | -1.248570 |

**TfO- E= -961.622424 G= -961.624347**

| | | | |
|---|---|---|---|
| O | -1.241218 | -0.637971 | 1.283988 |
| O | -1.241826 | 1.430966 | -0.089450 |
| C | 0.948266 | 0.000038 | -0.000090 |
| F | 1.435082 | 0.554971 | -1.120772 |
| F | 1.434337 | -1.248201 | 0.079763 |
| F | 1.434647 | 0.693110 | 1.041024 |
| S | -0.914235 | 0.000048 | -0.000031 |
| O | -1.241760 | -0.792984 | -1.194425 |

**TS-dep E= -1763.252195 G= -1762.870041**

| | | | |
|---|---|---|---|
| C | 4.501600 | -1.539351 | 0.789291 |
| C | 4.060460 | -0.687143 | -0.217827 |
| C | 2.800496 | -0.089890 | -0.130954 |
| C | 2.012053 | -0.371979 | 0.988384 |
| C | 2.461443 | -1.198892 | 2.020687 |
| C | 3.712893 | -1.790585 | 1.912012 |
| C | 0.385291 | 1.836606 | 0.387697 |
| C | 1.202886 | 2.111599 | -0.716064 |
| C | 1.122906 | 3.382618 | -1.290068 |
| H | 1.747534 | 3.629800 | -2.143591 |
| C | 0.260961 | 4.338072 | -0.760443 |
| C | -0.516979 | 4.059278 | 0.362826 |
| C | -0.453130 | 2.802952 | 0.948557 |
| H | 4.700360 | -0.476257 | -1.069742 |
| H | 1.836919 | -1.384761 | 2.890041 |
| H | -1.058114 | 2.567978 | 1.819390 |
| S | 0.365004 | 0.253682 | 1.168346 |
| S | 2.300886 | 0.948206 | -1.472482 |
| C | -0.818617 | -1.771171 | -2.151219 |
| C | -0.604867 | -0.683672 | -1.347441 |
| C | -0.658521 | -0.829731 | 0.092686 |
| C | -0.730111 | -2.173067 | 0.636697 |
| C | -0.937792 | -3.235931 | -0.194409 |
| C | -0.981616 | -3.059461 | -1.598396 |
| H | -1.783768 | -0.348204 | 0.348822 |
| H | -0.515505 | 0.305702 | -1.781748 |
| H | -0.850380 | -1.644629 | -3.228441 |
| O | -3.072854 | 0.257620 | 0.662387 |
| C | -3.782947 | -0.703116 | 1.476259 |
| H | -4.843862 | -0.437770 | 1.450857 |
| H | -3.661933 | -1.700334 | 1.033193 |
| C | -3.247526 | -0.638345 | 2.888581 |
| H | -3.368444 | 0.368179 | 3.301584 |
| H | -3.810452 | -1.339419 | 3.513786 |
| H | -2.190975 | -0.918300 | 2.946078 |
| C | -3.696202 | 0.545729 | -0.610371 |
| H | -4.776666 | 0.595838 | -0.447563 |
| H | -3.488988 | -0.284217 | -1.298539 |
| C | -3.167702 | 1.863616 | -1.129780 |
| H | -3.622696 | 2.069014 | -2.104639 |
| H | -3.425170 | 2.678986 | -0.446690 |
| H | -2.080699 | 1.847992 | -1.258234 |
| H | -0.675614 | -2.320311 | 1.712320 |
| H | -1.050801 | -4.232699 | 0.219694 |
| C | -1.225098 | -4.230316 | -2.482173 |
| H | 5.482398 | -1.996192 | 0.702250 |
| H | 4.070769 | -2.441311 | 2.702716 |
| H | 0.215422 | 5.319857 | -1.221088 |
| H | -1.173360 | 4.815029 | 0.780645 |
| H | -2.309501 | -4.406294 | -2.528006 |
| H | -0.869939 | -4.050083 | -3.499434 |
| H | -0.767231 | -5.135683 | -2.073781 |

**TS-III E= -1529.570306 G= -1529.317147**

| | | | |
|---|---|---|---|
| C | -2.687703 | -2.347229 | 0.680812 |
| C | -1.553731 | -2.461752 | -0.098744 |

| | | | |
|---|---|---|---|
| C | -0.489131 | -1.564818 | 0.074367 |
| C | -0.626969 | -0.515255 | 1.033434 |
| C | -1.807770 | -0.413627 | 1.816731 |
| C | -2.812862 | -1.333036 | 1.651600 |
| C | 2.016931 | 0.224373 | 0.607578 |
| C | 2.170863 | -0.821105 | -0.325998 |
| C | 3.440190 | -1.058677 | -0.875489 |
| H | 3.580710 | -1.864963 | -1.589511 |
| C | 4.510059 | -0.266141 | -0.505429 |
| C | 4.351909 | 0.779010 | 0.420815 |
| C | 3.115909 | 1.029940 | 0.973040 |
| H | -1.473462 | -3.263821 | -0.826378 |
| H | -1.896828 | 0.380688 | 2.551618 |
| H | 2.984788 | 1.838308 | 1.686764 |
| S | 0.524130 | 0.700544 | 1.314217 |
| S | 0.904163 | -1.860035 | -0.896255 |
| C | -1.289293 | 0.902181 | -1.834344 |
| C | -0.153413 | 1.600570 | -1.490672 |
| C | -0.175620 | 2.475370 | -0.373046 |
| C | -1.403978 | 2.732773 | 0.295603 |
| C | -2.529594 | 2.029032 | -0.055991 |
| C | -2.488790 | 1.077777 | -1.107468 |
| H | 0.694217 | 3.089775 | -0.154150 |
| H | 0.771532 | 1.464334 | -2.042791 |
| H | -1.273742 | 0.201809 | -2.664404 |
| H | -1.427461 | 3.452511 | 1.107866 |
| H | -3.493412 | -3.061490 | 0.546797 |
| H | -3.707766 | -1.270803 | 2.260368 |
| H | 5.486348 | -0.460403 | -0.937277 |
| H | 5.202421 | 1.389418 | 0.703773 |
| H | -3.465118 | 2.188450 | 0.472242 |
| C | -3.707829 | 0.293431 | -1.436712 |
| H | -4.020361 | -0.293172 | -0.560261 |
| H | -4.542285 | 0.968259 | -1.664607 |
| H | -3.547346 | -0.383932 | -2.277958 |

**TS-II E= -2018.509145 G= -2018.216443**

| | | | |
|---|---|---|---|
| C | 0.750968 | 4.186499 | 0.459378 |
| C | 1.184927 | 3.090519 | 1.160437 |
| C | 1.418389 | 1.869777 | 0.473657 |
| C | 1.195804 | 1.775015 | -0.925423 |
| C | 0.757969 | 2.916385 | -1.615292 |
| C | 0.535368 | 4.093633 | -0.930420 |
| C | 2.139273 | -0.833676 | -0.880032 |
| C | 2.368159 | -0.713412 | 0.516187 |
| C | 2.978430 | -1.770928 | 1.241525 |
| H | 3.144660 | -1.666374 | 2.310171 |
| C | 3.351488 | -2.914050 | 0.582311 |
| C | 3.120847 | -3.034319 | -0.803088 |
| C | 2.532088 | -2.016233 | -1.525868 |
| H | 1.352550 | 3.148582 | 2.232405 |
| H | 0.591655 | 2.869365 | -2.687489 |
| H | 2.376223 | -2.127322 | -2.594761 |
| S | 1.424188 | 0.365542 | -1.888210 |
| S | 1.880928 | 0.589129 | 1.480268 |
| O | -0.742977 | -0.378512 | 0.212432 |
| C | -0.867465 | -0.357390 | 1.438539 |
| O | 0.022603 | -0.054626 | 2.290032 |

| | | | |
|---|---|---|---|
| C | -2.242068 | -0.699086 | 2.078130 |
| F | -2.107583 | -1.577821 | 3.074450 |
| F | -3.071289 | -1.218918 | 1.168181 |
| F | -2.802856 | 0.414408 | 2.569068 |
| H | 3.417587 | -3.942055 | -1.318423 |
| H | 3.823195 | -3.724264 | 1.126802 |
| H | 0.193224 | 4.965530 | -1.478438 |
| H | 0.574821 | 5.123829 | 0.975216 |
| O | -2.253275 | -0.607136 | -1.853812 |
| C | -2.732853 | 1.744672 | -1.544697 |
| H | -1.938422 | 2.002731 | -2.250736 |
| H | -3.523949 | 2.497103 | -1.624233 |
| H | -2.340826 | 1.776452 | -0.523175 |
| C | -3.334524 | 0.403062 | -1.875963 |
| H | -3.726254 | 0.357251 | -2.891943 |
| H | -4.094258 | 0.080741 | -1.161059 |
| C | -2.649426 | -1.998375 | -2.164246 |
| H | -3.433316 | -2.278111 | -1.457559 |
| H | -3.052453 | -1.945395 | -3.175468 |
| C | -1.423394 | -2.869780 | -2.069937 |
| H | -1.712604 | -3.888176 | -2.348098 |
| H | -0.645189 | -2.531463 | -2.760394 |
| H | -1.023485 | -2.900102 | -1.051576 |
| H | -1.744151 | -0.563773 | -0.979860 |

**TS-I E= -2055.956702 G= -2055.687334**

| | | | |
|---|---|---|---|
| C | 1.692322 | -2.910486 | 1.159265 |
| C | 0.999679 | -2.148052 | 0.238767 |
| C | 0.867261 | -0.762034 | 0.438920 |
| C | 1.413780 | -0.139981 | 1.573177 |
| C | 2.120081 | -0.930054 | 2.489296 |
| C | 2.259523 | -2.293286 | 2.279669 |
| C | 0.100480 | 2.247561 | 0.905332 |
| C | -0.428975 | 1.649194 | -0.242179 |
| C | -1.376498 | 2.308344 | -1.038662 |
| H | -1.788438 | 1.810964 | -1.911770 |
| C | -1.793957 | 3.579475 | -0.687954 |
| C | -1.271265 | 4.188773 | 0.457333 |
| C | -0.339624 | 3.533196 | 1.247719 |
| H | 0.550220 | -2.614535 | -0.632987 |
| H | 2.550040 | -0.472325 | 3.375654 |
| H | 0.050620 | 4.016758 | 2.139006 |
| S | 1.333496 | 1.566561 | 1.951393 |
| S | 0.004915 | 0.057475 | -0.824810 |
| O | -3.029241 | -0.443525 | -1.731807 |
| C | -2.947852 | -0.801531 | -0.557118 |
| O | -1.971139 | -0.750404 | 0.242484 |
| C | -4.212985 | -1.443325 | 0.095467 |
| F | -3.998691 | -2.748975 | 0.371246 |
| F | -5.295838 | -1.379949 | -0.693280 |
| F | -4.534242 | -0.841962 | 1.258613 |
| C | 3.641663 | 0.633953 | -0.698659 |
| C | 2.594162 | 1.327328 | -1.249542 |
| C | 1.681939 | 0.665708 | -2.141446 |
| C | 2.037471 | -0.641292 | -2.631953 |
| C | 3.081229 | -1.319055 | -2.070304 |
| C | 3.866383 | -0.715373 | -1.050429 |
| H | 1.050641 | 1.288615 | -2.774703 |

| | | | |
|---|---|---|---|
| H | 2.415524 | 2.366759 | -0.992791 |
| H | 4.296570 | 1.109217 | 0.024914 |
| H | 2.808011 | -2.885770 | 3.005065 |
| H | 1.794992 | -3.979397 | 1.007324 |
| H | -1.599465 | 5.183716 | 0.741019 |
| H | -2.529664 | 4.092517 | -1.298048 |
| H | 1.416366 | -1.102383 | -3.394006 |
| H | 3.316842 | -2.330720 | -2.385696 |
| C | 4.898023 | -1.519459 | -0.347795 |
| H | 5.394717 | -2.215118 | -1.030990 |
| H | 5.634523 | -0.891566 | 0.158878 |
| H | 4.388886 | -2.129510 | 0.415742 |

**TS-m-dep E= -1763.243086 G= -1762.860198**

| | | | |
|---|---|---|---|
| C | 3.857041 | -2.535132 | 0.965272 |
| C | 3.634678 | -1.726760 | -0.145996 |
| C | 2.594806 | -0.795685 | -0.135318 |
| C | 1.797174 | -0.706696 | 1.009719 |
| C | 2.032763 | -1.490134 | 2.140445 |
| C | 3.070597 | -2.413280 | 2.110387 |
| C | 0.915076 | 1.810450 | 0.203446 |
| C | 1.755013 | 1.718250 | -0.914925 |
| C | 2.112775 | 2.897903 | -1.569293 |
| H | 2.768820 | 2.858629 | -2.433758 |
| C | 1.645362 | 4.123878 | -1.104132 |
| C | 0.840540 | 4.200907 | 0.031978 |
| C | 0.476504 | 3.039433 | 0.700545 |
| H | 4.278150 | -1.810957 | -1.016844 |
| H | 1.407623 | -1.389095 | 3.022632 |
| H | -0.143685 | 3.087346 | 1.590454 |
| S | 0.388033 | 0.365678 | 1.075544 |
| S | 2.339588 | 0.187962 | -1.584722 |
| C | -1.284149 | -1.080545 | -2.345386 |
| C | -0.888465 | -0.130915 | -1.430766 |
| C | -0.832241 | -0.491985 | -0.032963 |
| C | -0.968218 | -1.888158 | 0.337515 |
| C | -1.321875 | -2.829902 | -0.595425 |
| C | -1.467435 | -2.403420 | -1.936965 |
| H | -1.926835 | -0.039492 | 0.336747 |
| H | -0.736021 | 0.902161 | -1.728770 |
| H | -1.736993 | -3.145139 | -2.684783 |
| H | -1.411946 | -0.809805 | -3.387293 |
| O | -3.274381 | 0.569544 | 0.706339 |
| C | -4.108994 | 0.477064 | -0.466129 |
| H | -4.949934 | 1.164018 | -0.329665 |
| H | -3.533801 | 0.811753 | -1.340676 |
| C | -4.602122 | -0.941766 | -0.621378 |
| H | -5.203948 | -1.238558 | 0.243583 |
| H | -5.227003 | -1.005103 | -1.518468 |
| H | -3.777455 | -1.650103 | -0.734047 |
| C | -3.057566 | 1.924221 | 1.152015 |
| H | -3.996630 | 2.473279 | 1.034814 |
| H | -2.310754 | 2.394981 | 0.498949 |
| C | -2.637850 | 1.908865 | 2.604438 |
| H | -2.445062 | 2.935510 | 2.933007 |
| H | -3.432661 | 1.487663 | 3.227890 |
| H | -1.726492 | 1.325982 | 2.773379 |
| H | -0.854715 | -2.169707 | 1.381637 |

| | | | |
|---|---|---|---|
| C | -1.559512 | -4.266337 | -0.229675 |
| H | 4.669783 | -3.254384 | 0.940604 |
| H | 3.263322 | -3.033349 | 2.979356 |
| H | 1.934008 | 5.030924 | -1.625899 |
| H | 0.499035 | 5.161970 | 0.401033 |
| H | -1.303552 | -4.455008 | 0.815932 |
| H | -2.613420 | -4.523644 | -0.382863 |
| H | -0.960134 | -4.926004 | -0.865280 |

**TS-o-dep E= -1763.246675 G= -1762.863061**

| | | | |
|---|---|---|---|
| C | 4.448287 | -1.821603 | 0.505643 |
| C | 4.014219 | -0.923327 | -0.464261 |
| C | 2.776417 | -0.291407 | -0.331089 |
| C | 1.991847 | -0.598571 | 0.785698 |
| C | 2.445342 | -1.456856 | 1.790506 |
| C | 3.678752 | -2.076752 | 1.640194 |
| C | 0.516533 | 1.738478 | 0.396232 |
| C | 1.341715 | 2.046688 | -0.693858 |
| C | 1.374348 | 3.370095 | -1.138271 |
| H | 2.008478 | 3.642299 | -1.976677 |
| C | 0.611237 | 4.342882 | -0.499254 |
| C | -0.174037 | 4.025936 | 0.608699 |
| C | -0.216442 | 2.717347 | 1.070531 |
| H | 4.647284 | -0.696411 | -1.317013 |
| H | 1.840297 | -1.642478 | 2.673014 |
| H | -0.811053 | 2.457377 | 1.941665 |
| S | 0.377818 | 0.091969 | 1.024323 |
| S | 2.300201 | 0.856994 | -1.588539 |
| C | -0.942838 | -1.241924 | -2.565445 |
| C | -0.705347 | -0.352064 | -1.552918 |
| C | -0.719050 | -0.801785 | -0.172958 |
| C | -0.837536 | -2.227961 | 0.120388 |
| C | -1.075330 | -3.076602 | -0.945040 |
| C | -1.097335 | -2.603065 | -2.257930 |
| H | -1.871303 | -0.436529 | 0.173855 |
| H | -0.624059 | 0.709474 | -1.759280 |
| H | -1.234423 | -4.132715 | -0.751437 |
| H | -1.256207 | -3.311122 | -3.065849 |
| H | -0.997765 | -0.900778 | -3.592784 |
| C | -0.808933 | -2.755411 | 1.517830 |
| O | -3.271770 | -0.075401 | 0.462710 |
| C | -3.899164 | 0.257702 | -0.796099 |
| H | -4.720500 | 0.949847 | -0.590577 |
| H | -3.165498 | 0.782701 | -1.421514 |
| C | -4.408973 | -1.004116 | -1.450968 |
| H | -5.189706 | -1.467278 | -0.839122 |
| H | -4.832760 | -0.756628 | -2.430119 |
| H | -3.606366 | -1.731664 | -1.602447 |
| C | -3.442008 | 0.931598 | 1.484376 |
| H | -4.516364 | 1.119996 | 1.574221 |
| H | -2.952025 | 1.855626 | 1.156152 |
| C | -2.888726 | 0.419166 | 2.792848 |
| H | -3.069363 | 1.169069 | 3.569899 |
| H | -3.384483 | -0.511493 | 3.086206 |
| H | -1.809592 | 0.241245 | 2.755256 |
| H | 5.413233 | -2.303842 | 0.384933 |
| H | 4.037288 | -2.752870 | 2.408951 |
| H | 0.651430 | 5.365897 | -0.859669 |

| | | | |
|---|---|---|---|
| H | -0.746437 | 4.794498 | 1.116913 |
| H | -1.504451 | -3.593905 | 1.608195 |
| H | 0.192693 | -3.139485 | 1.748181 |
| H | -1.070359 | -2.001727 | 2.265704 |

**TS-p-dep E= -1763.252195 G= -1762.870041**

| | | | |
|---|---|---|---|
| C | 4.501600 | -1.539351 | 0.789291 |
| C | 4.060460 | -0.687143 | -0.217827 |
| C | 2.800496 | -0.089890 | -0.130954 |
| C | 2.012053 | -0.371979 | 0.988384 |
| C | 2.461443 | -1.198892 | 2.020687 |
| C | 3.712893 | -1.790585 | 1.912012 |
| C | 0.385291 | 1.836606 | 0.387697 |
| C | 1.202886 | 2.111599 | -0.716064 |
| C | 1.122906 | 3.382618 | -1.290068 |
| H | 1.747534 | 3.629800 | -2.143591 |
| C | 0.260961 | 4.338072 | -0.760443 |
| C | -0.516979 | 4.059278 | 0.362826 |
| C | -0.453130 | 2.802952 | 0.948557 |
| H | 4.700360 | -0.476257 | -1.069742 |
| H | 1.836919 | -1.384761 | 2.890041 |
| H | -1.058114 | 2.567978 | 1.819390 |
| S | 0.365004 | 0.253682 | 1.168346 |
| S | 2.300886 | 0.948206 | -1.472482 |
| C | -0.818617 | -1.771171 | -2.151219 |
| C | -0.604867 | -0.683672 | -1.347441 |
| C | -0.658521 | -0.829731 | 0.092686 |
| C | -0.730111 | -2.173067 | 0.636697 |
| C | -0.937792 | -3.235931 | -0.194409 |
| C | -0.981616 | -3.059461 | -1.598396 |
| H | -1.783768 | -0.348204 | 0.348822 |
| H | -0.515505 | 0.305702 | -1.781748 |
| H | -0.850380 | -1.644629 | -3.228441 |
| O | -3.072854 | 0.257620 | 0.662387 |
| C | -3.782947 | -0.703116 | 1.476259 |
| H | -4.843862 | -0.437770 | 1.450857 |
| H | -3.661933 | -1.700334 | 1.033193 |
| C | -3.247526 | -0.638345 | 2.888581 |
| H | -3.368444 | 0.368179 | 3.301584 |
| H | -3.810452 | -1.339419 | 3.513786 |
| H | -2.190975 | -0.918300 | 2.946078 |
| C | -3.696202 | 0.545729 | -0.610371 |
| H | -4.776666 | 0.595838 | -0.447563 |
| H | -3.488988 | -0.284217 | -1.298539 |
| C | -3.167702 | 1.863616 | -1.129780 |
| H | -3.622696 | 2.069014 | -2.104639 |
| H | -3.425170 | 2.678986 | -0.446690 |
| H | -2.080699 | 1.847992 | -1.258234 |
| H | -0.675614 | -2.320311 | 1.712320 |
| H | -1.050801 | -4.232699 | 0.219694 |
| C | -1.225098 | -4.230316 | -2.482173 |
| H | 5.482398 | -1.996192 | 0.702250 |
| H | 4.070769 | -2.441311 | 2.702716 |
| H | 0.215422 | 5.319857 | -1.221088 |
| H | -1.173360 | 4.815029 | 0.780645 |
| H | -2.309501 | -4.406294 | -2.528006 |
| H | -0.869939 | -4.050083 | -3.499434 |
| H | -0.767231 | -5.135683 | -2.073781 |

**TT2++4 E= -1529.570563 G= -1529.317662**

| | | | |
|---|---|---|---|
| C | 3.453439 | -1.204795 | 1.108704 |
| C | 2.362436 | -0.499706 | 1.544157 |
| C | 1.115359 | -0.661834 | 0.883501 |
| C | 1.011553 | -1.545843 | -0.240829 |
| C | 2.165428 | -2.215053 | -0.690674 |
| C | 3.355753 | -2.050591 | -0.018807 |
| C | -1.740785 | -1.149233 | -0.276299 |
| C | -1.624233 | -0.272450 | 0.838124 |
| C | -2.782153 | 0.286762 | 1.428320 |
| H | -2.685318 | 0.960252 | 2.274997 |
| C | -4.017809 | -0.034084 | 0.923696 |
| C | -4.133525 | -0.898468 | -0.185332 |
| C | -3.019400 | -1.448726 | -0.780697 |
| H | 2.436125 | 0.168149 | 2.397298 |
| H | 2.106620 | -2.880928 | -1.546449 |
| H | -3.125324 | -2.119004 | -1.628493 |
| S | -0.429617 | -1.906367 | -1.085844 |
| S | -0.157439 | 0.249960 | 1.522400 |
| C | 1.888866 | 2.622818 | 0.365680 |
| C | 2.573611 | 1.842866 | -0.554589 |
| C | 1.879078 | 1.248454 | -1.625085 |
| C | 0.512145 | 1.450568 | -1.778558 |
| C | -0.202035 | 2.214780 | -0.849048 |
| C | 0.505242 | 2.781110 | 0.243293 |
| H | 2.420581 | 0.643666 | -2.346444 |
| H | 3.642892 | 1.686110 | -0.453795 |
| H | -0.037294 | 3.388484 | 0.963787 |
| H | 2.412505 | 3.082490 | 1.198054 |
| H | -0.011419 | 1.011840 | -2.624447 |
| C | -1.672382 | 2.444769 | -1.012786 |
| H | -5.116071 | -1.138967 | -0.577291 |
| H | -4.909048 | 0.383487 | 1.378617 |
| H | 4.401530 | -1.099922 | 1.624033 |
| H | 4.233758 | -2.588454 | -0.360578 |
| H | -1.843480 | 3.203868 | -1.787419 |
| H | -2.182813 | 1.533028 | -1.345955 |
| H | -2.133889 | 2.800375 | -0.087441 |

**TT2+ E= -1258.000428 G= -1257.871308**

| | | | |
|---|---|---|---|
| C | 3.786800 | -0.709740 | 0.000305 |
| C | 2.613103 | -1.418779 | -0.000217 |
| C | 1.384047 | -0.720210 | -0.000350 |
| C | 1.384048 | 0.720212 | -0.000048 |
| C | 2.613104 | 1.418779 | 0.000319 |
| C | 3.786800 | 0.709739 | 0.000553 |
| C | -1.384047 | 0.720211 | -0.000217 |
| C | -1.384048 | -0.720212 | -0.000184 |
| C | -2.613104 | -1.418779 | 0.000182 |
| H | -2.622144 | -2.505061 | 0.000269 |
| C | -3.786800 | -0.709739 | 0.000529 |
| C | -3.786800 | 0.709740 | 0.000314 |
| C | -2.613103 | 1.418779 | -0.000088 |
| H | 2.622142 | -2.505061 | -0.000418 |
| H | 2.622145 | 2.505061 | 0.000460 |
| H | -2.622142 | 2.505061 | -0.000236 |
| S | 0.000000 | 1.695588 | -0.000014 |

S -0.000000 -1.695588 -0.000579

H 4.731459 -1.242223 0.000525

H 4.731460 1.242221 0.000896

H -4.731459 -1.242221 0.000928

H -4.731459 1.242222 0.000474

**TT•++4•+ E= -1529.567015 G= -1529.318170**

C 3.36164000 -1.27166400 0.88931100

C 2.23553900 -0.79790500 1.53250500

C 0.96436000 -0.97553100 0.95520700

C 0.85342400 -1.63201600 -0.28923900

C 2.01354900 -2.12037800 -0.92453500

C 3.24897500 -1.94102900 -0.34187800

C -1.87176900 -1.01668500 -0.36751500

C -1.76632700 -0.37992300 0.88774000

C -2.88052900 0.29949900 1.41666100

H -2.80146000 0.79748900 2.37890200

C -4.06295800 0.34452900 0.70920900

C -4.16785400 -0.28815200 -0.54181300

C -3.08986600 -0.96218200 -1.07396900

H 2.32963300 -0.28061000 2.48298800

H 1.92912200 -2.62593200 -1.88237500

H -3.17368300 -1.44595500 -2.04316600

S -0.61975000 -1.92363800 -1.15151100

S -0.35572300 -0.36291000 1.89861600

C 2.07833700 2.53079300 0.65642500

C 2.82899200 1.83777900 -0.33492000

C 2.21203600 1.36584000 -1.52288800

C 0.87669000 1.58268800 -1.71832200

C 0.10275400 2.28829000 -0.73316600

C 0.74323300 2.75060300 0.46249300

H 2.80656000 0.83851500 -2.26048800

H 3.89217100 1.67963500 -0.18453000

H 0.14805700 3.27810600 1.20122200

H 2.57675700 2.87542600 1.55588000

H 0.37721500 1.23506300 -2.61768300

C -1.32654500 2.56131000 -0.97677800

H -5.09938100 -0.24529900 -1.09619000

H -4.91367800 0.87692700 1.12141200

H 4.33708400 -1.12208000 1.34013000

H 4.13739600 -2.31177700 -0.84200300

H -1.38662300 3.44200700 -1.64017200

H -1.80739700 1.73907900 -1.51699700

H -1.86628600 2.79897700 -0.05776800

TT E= -1258.450189 G= -1258.319474

C 0.782781 -0.697281 3.582296

C 0.315291 -1.395815 2.471943

C -0.189998 -0.700090 1.370683

C -0.189998 0.700089 1.370683

C 0.315291 1.395816 2.471943

C 0.782781 0.697281 3.582296

C -0.189998 0.700089 -1.370683

C -0.189998 -0.700090 -1.370683

C 0.315291 -1.395815 -2.471943

H 0.335757 -2.482098 -2.462110

C 0.782781 -0.697281 -3.582296

C 0.782781 0.697281 -3.582296

C 0.315291 1.395816 -2.471943

H 0.335757 -2.482098 2.462110

H 0.335758 2.482098 2.462111

H 0.335758 2.482098 -2.462111

S -0.867998 1.611145 0.000000

S -0.867997 -1.611145 -0.000000

H 1.159780 1.244868 -4.441078

H 1.159779 -1.244868 -4.441078

H 1.159780 1.244868 4.441078

H 1.159779 -1.244868 4.441078

**TT•+ E= -1258.241651 G= -1258.112938**

C 3.81932000 0.70321800 0.00004100

C 2.63004600 1.39996600 0.00001000

C 1.40326700 0.70557700 -0.00001300

C 1.40326700 -0.70557700 -0.00000400

C 2.63004600 -1.39996600 0.00003300

C 3.81931900 -0.70321900 0.00005400

C -1.40326700 -0.70557700 -0.00001400

C -1.40326700 0.70557700 -0.00000300

C -2.63004600 1.39996600 0.00003500

H -2.63410600 2.48675600 0.00005400

C -3.81931900 0.70321900 0.00005500

C -3.81932000 -0.70321800 0.00003900

C -2.63004600 -1.39996600 0.00000700

H 2.63410600 2.48675600 0.00001000

H 2.63410600 -2.48675600 0.00004900

H -2.63410600 -2.48675600 0.00000400

S 0.00000000 -1.72351100 -0.00006100

S 0.00000000 1.72351100 -0.00005400

H -4.75837300 1.24659700 0.00008300

H -4.75837300 -1.24659700 0.00005400

H 4.75837300 1.24659700 0.00005600

H 4.75837300 -1.24659700 0.00008100

**TT+OH-H+-OEt2 E= -1568.127345 G= -1567.842043**

C 3.800533 -1.285870 0.685194

C 2.603721 -1.234763 1.354981

C 1.397866 -1.114901 0.616848

C 1.416409 -1.038646 -0.798316

C 2.655663 -1.093111 -1.452847

C 3.820973 -1.209689 -0.720232

C -1.358064 -1.069890 -0.824908

C -1.356326 -1.155022 0.591470

C -2.571280 -1.313723 1.311373

H -2.554714 -1.372277 2.396373

C -3.755134 -1.396057 0.623771

C -3.757029 -1.309337 -0.781983

C -2.585570 -1.154320 -1.497393

H 2.572070 -1.285369 2.439710

H 2.698867 -1.044134 -2.536938

H -2.614781 -1.098602 -2.581600

S 0.035425 -0.875603 -1.825575

S 0.007213 -0.971204 1.575447

O 0.126291 1.027549 1.905727

H -4.696731 -1.372923 -1.320767

H -4.686894 -1.526196 1.162479

| | | | |
|---|---|---|---|
| H | 4.769768 | -1.249788 | -1.245131 |
| H | 4.727309 | -1.382587 | 1.239321 |
| O | -0.067275 | 2.453590 | -0.104099 |
| C | 2.322219 | 2.387660 | -0.430484 |
| H | 2.223201 | 1.863921 | -1.386074 |
| H | 3.219777 | 3.013579 | -0.473383 |
| H | 2.465877 | 1.652630 | 0.368882 |
| C | 1.123607 | 3.272773 | -0.176383 |
| H | 0.980907 | 3.983629 | -0.996017 |
| H | 1.218537 | 3.831231 | 0.762757 |
| C | -1.291066 | 3.224337 | -0.072868 |
| H | -1.335598 | 3.768222 | 0.878857 |
| H | -1.240562 | 3.950158 | -0.889842 |
| C | -2.469511 | 2.294584 | -0.249020 |
| H | -3.390365 | 2.887240 | -0.243385 |
| H | -2.406587 | 1.765308 | -1.204400 |
| H | -2.541499 | 1.562467 | 0.562533 |
| H | 0.007397 | 1.635851 | 1.019062 |
| H | -0.552388 | 1.293282 | 2.561379 |

**TT+-OH E= -1334.053421 G= -1333.909460**

| | | | |
|---|---|---|---|
| C | 3.667686 | -0.487504 | -0.877218 |
| C | 2.507897 | -1.214594 | -0.655526 |
| C | 1.381594 | -0.559627 | -0.143116 |
| C | 1.383494 | 0.816916 | 0.124921 |
| C | 2.564948 | 1.528635 | -0.096511 |
| C | 3.693103 | 0.877139 | -0.584428 |
| C | -1.383490 | 0.816916 | 0.124919 |
| C | -1.381593 | -0.559627 | -0.143117 |
| C | -2.507896 | -1.214593 | -0.655528 |
| H | -2.474690 | -2.279444 | -0.868277 |
| C | -3.667685 | -0.487500 | -0.877220 |
| C | -3.693100 | 0.877143 | -0.584431 |
| C | -2.564944 | 1.528636 | -0.096513 |
| H | 2.474689 | -2.279446 | -0.868275 |
| H | 2.597143 | 2.597109 | 0.095733 |
| H | -2.597137 | 2.597111 | 0.095730 |
| S | 0.000002 | 1.713538 | 0.754728 |
| S | -0.000000 | -1.574369 | 0.210024 |
| O | -0.000025 | -1.599487 | 1.849964 |
| H | 0.000082 | -2.532298 | 2.149320 |
| H | -4.599074 | 1.449985 | -0.756305 |
| H | -4.548108 | -0.982711 | -1.272381 |
| H | 4.599078 | 1.449980 | -0.756303 |
| H | 4.548108 | -0.982715 | -1.272379 |

**TTO E= -1333.620734 G= -1333.487504**

| | | | |
|---|---|---|---|
| C | -0.469304 | -0.981295 | 3.602616 |
| C | -1.204653 | -0.695350 | 2.457080 |
| C | -0.578461 | -0.111124 | 1.354489 |
| C | 0.793430 | 0.165392 | 1.377640 |
| C | 1.524799 | -0.118597 | 2.534480 |
| C | 0.891469 | -0.677992 | 3.640971 |
| C | 0.793430 | 0.165392 | -1.377640 |
| C | -0.578461 | -0.111124 | -1.354489 |
| C | -1.204653 | -0.695350 | -2.457080 |
| H | -2.269434 | -0.911846 | -2.421780 |
| C | -0.469304 | -0.981295 | -3.602616 |

| | | | |
|---|---|---|---|
| C | 0.891469 | -0.677992 | -3.640971 |
| C | 1.524799 | -0.118597 | -2.534480 |
| H | -2.269434 | -0.911846 | 2.421780 |
| H | 2.592834 | 0.079259 | 2.564031 |
| H | 2.592834 | 0.079259 | -2.564031 |
| S | 1.664063 | 0.863640 | -0.000000 |
| S | -1.639609 | 0.391242 | 0.000000 |
| O | -1.694803 | 1.908880 | 0.000000 |
| H | 1.472991 | -0.896093 | -4.531861 |
| H | -0.956482 | -1.432090 | -4.461511 |
| H | 1.472991 | -0.896093 | 4.531861 |
| H | -0.956482 | -1.432090 | 4.461511 |

**TT+-TFA E= -1784.413058 G= -1784.262883**

| | | | |
|---|---|---|---|
| C | 3.015761 | -2.847307 | -1.054040 |
| C | 1.981237 | -2.034312 | -1.482406 |
| C | 1.563862 | -0.976743 | -0.658760 |
| C | 2.177664 | -0.704266 | 0.575481 |
| C | 3.213213 | -1.545631 | 0.988743 |
| C | 3.617816 | -2.604227 | 0.183399 |
| C | 1.009341 | 1.809332 | 0.606482 |
| C | 0.403351 | 1.541431 | -0.633279 |
| C | -0.136171 | 2.561165 | -1.434627 |
| H | -0.588069 | 2.325716 | -2.393773 |
| C | -0.086569 | 3.866481 | -0.978708 |
| C | 0.486904 | 4.142953 | 0.265685 |
| C | 1.034805 | 3.133745 | 1.049216 |
| H | 1.493635 | -2.214782 | -2.436134 |
| H | 3.717454 | -1.362034 | 1.932774 |
| H | 1.502480 | 3.378008 | 1.998254 |
| S | 1.713848 | 0.595701 | 1.666158 |
| S | 0.205542 | -0.070667 | -1.240809 |
| O | -2.632152 | 0.125308 | -1.428988 |
| C | -2.248252 | -0.494572 | -0.482622 |
| O | -0.967000 | -0.719875 | -0.142154 |
| C | -3.174221 | -1.164943 | 0.565774 |
| F | -2.935333 | -2.478251 | 0.624510 |
| F | -4.444680 | -0.970433 | 0.235382 |
| F | -2.953487 | -0.639335 | 1.775059 |
| H | 0.524233 | 5.166042 | 0.626114 |
| H | -0.497629 | 4.666168 | -1.584924 |
| H | 4.429767 | -3.240167 | 0.521816 |
| H | 3.350165 | -3.669438 | -1.677130 |

# SPECTROSCOPIC DATA

## $^1$H NMR of *tert*-butylbenzene derived thianthrenium salt 2, *para*-isomer

$CD_3CN$, 23 ºC

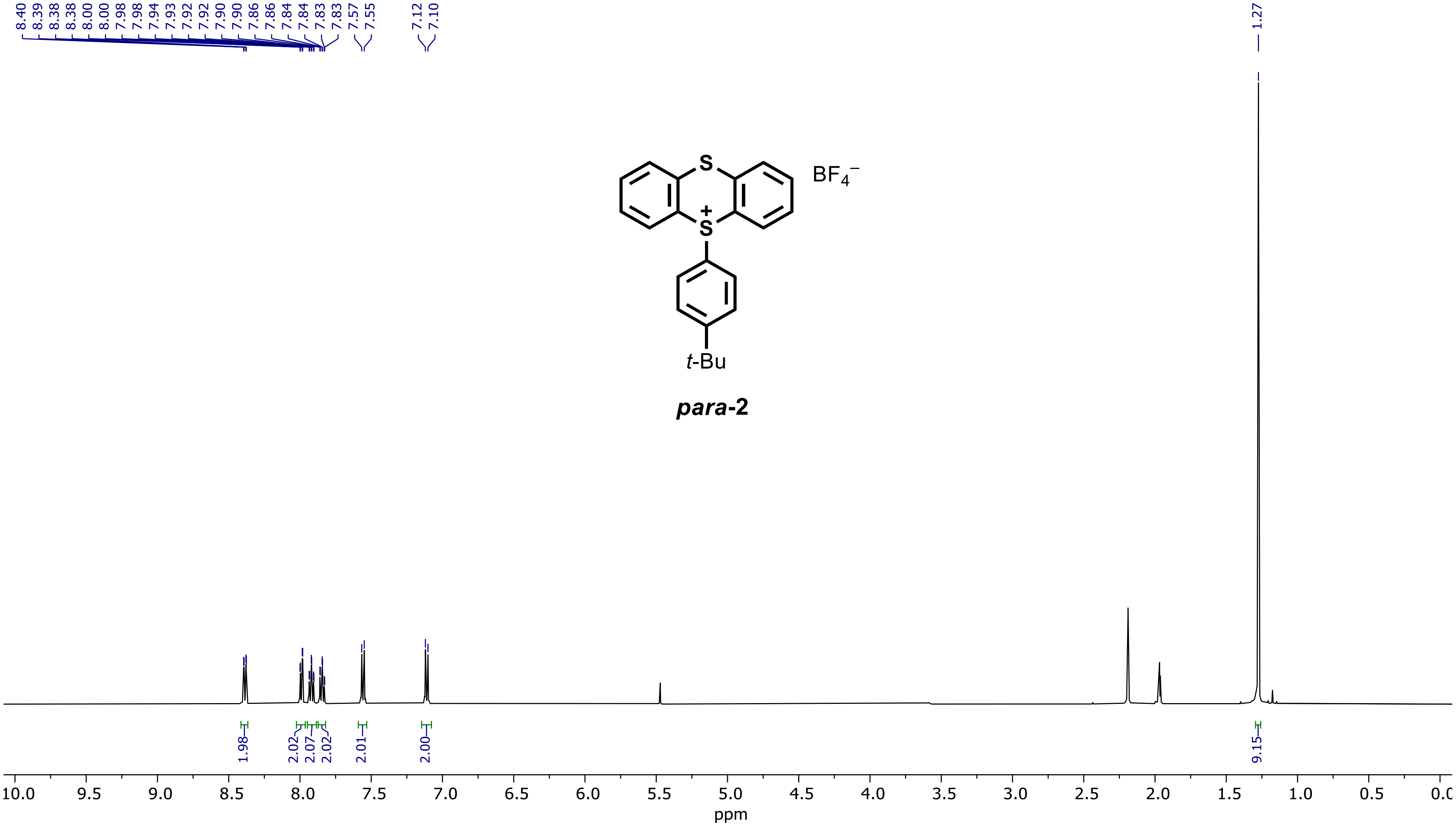

## $^{13}C$ NMR of *tert*-butylbenzene derived thianthrenium salt 2, *para*-isomer

CD$_3$CN, 23 ºC

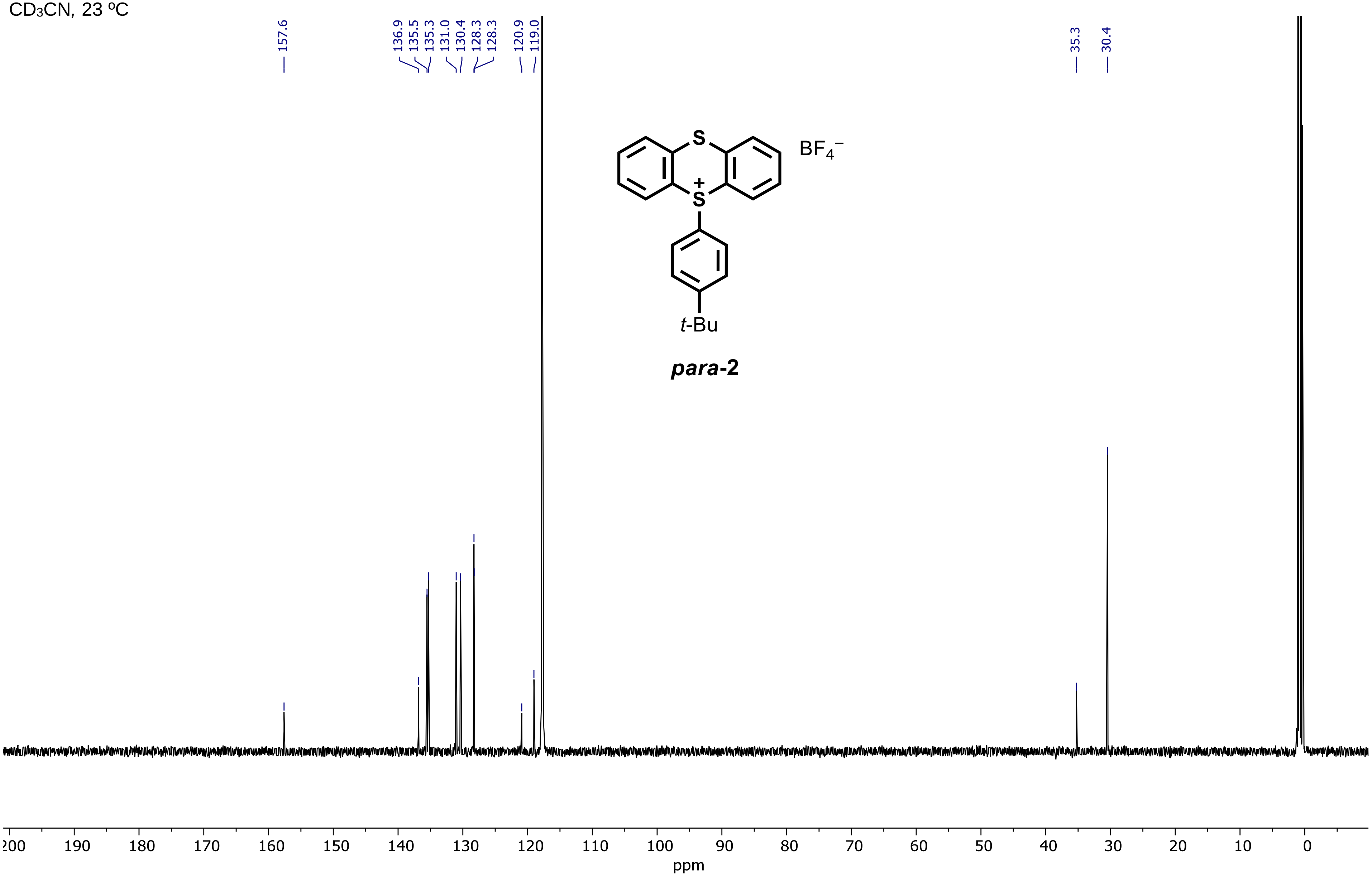

## $^{1}$H NMR of toluene derived thianthrenium salt 5, *para*-isomer

$CD_3CN$, 23 ºC

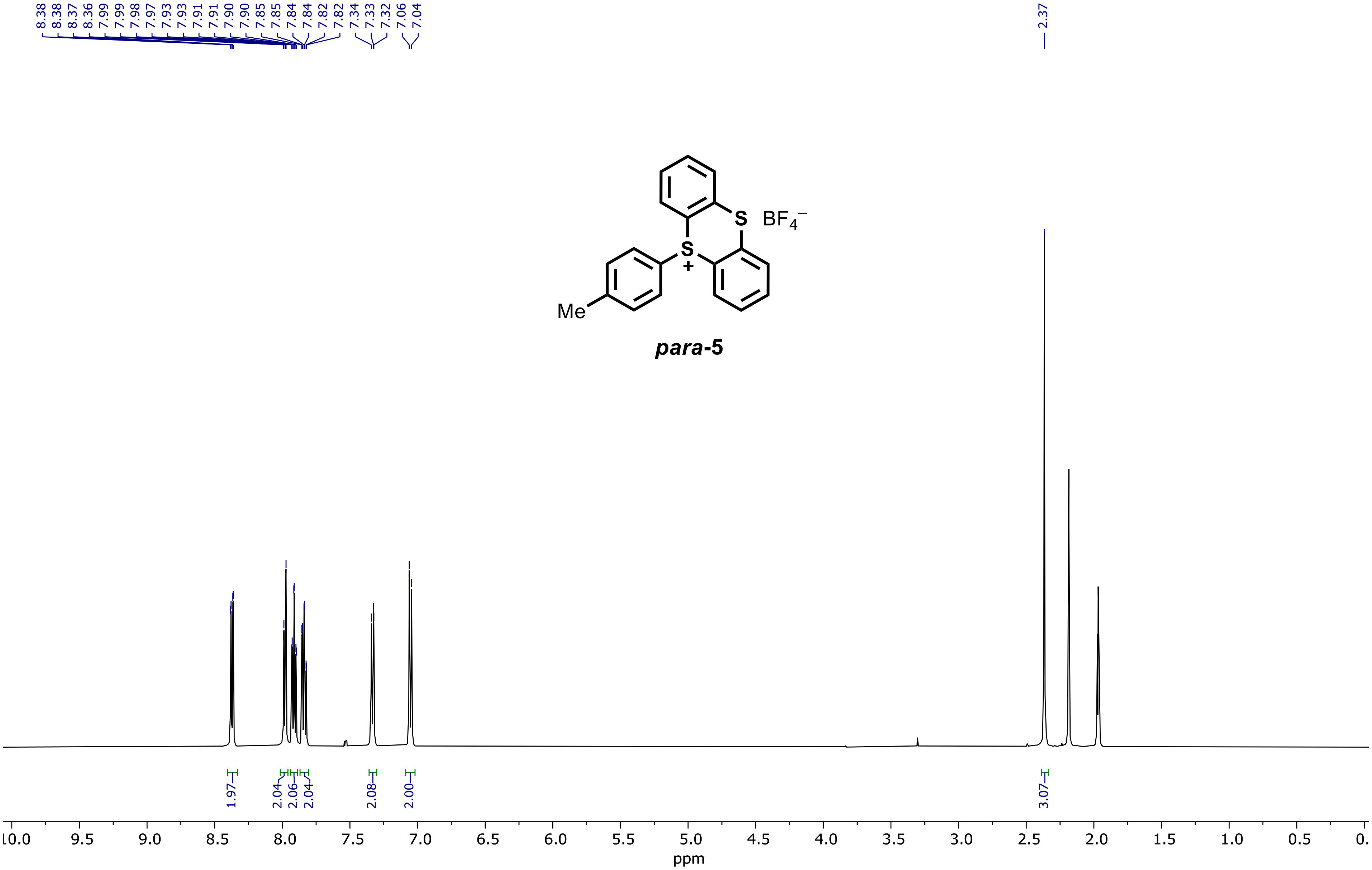

**$^{13}$C NMR of toluene derived thianthrenium salt 5, *para*-isomer**

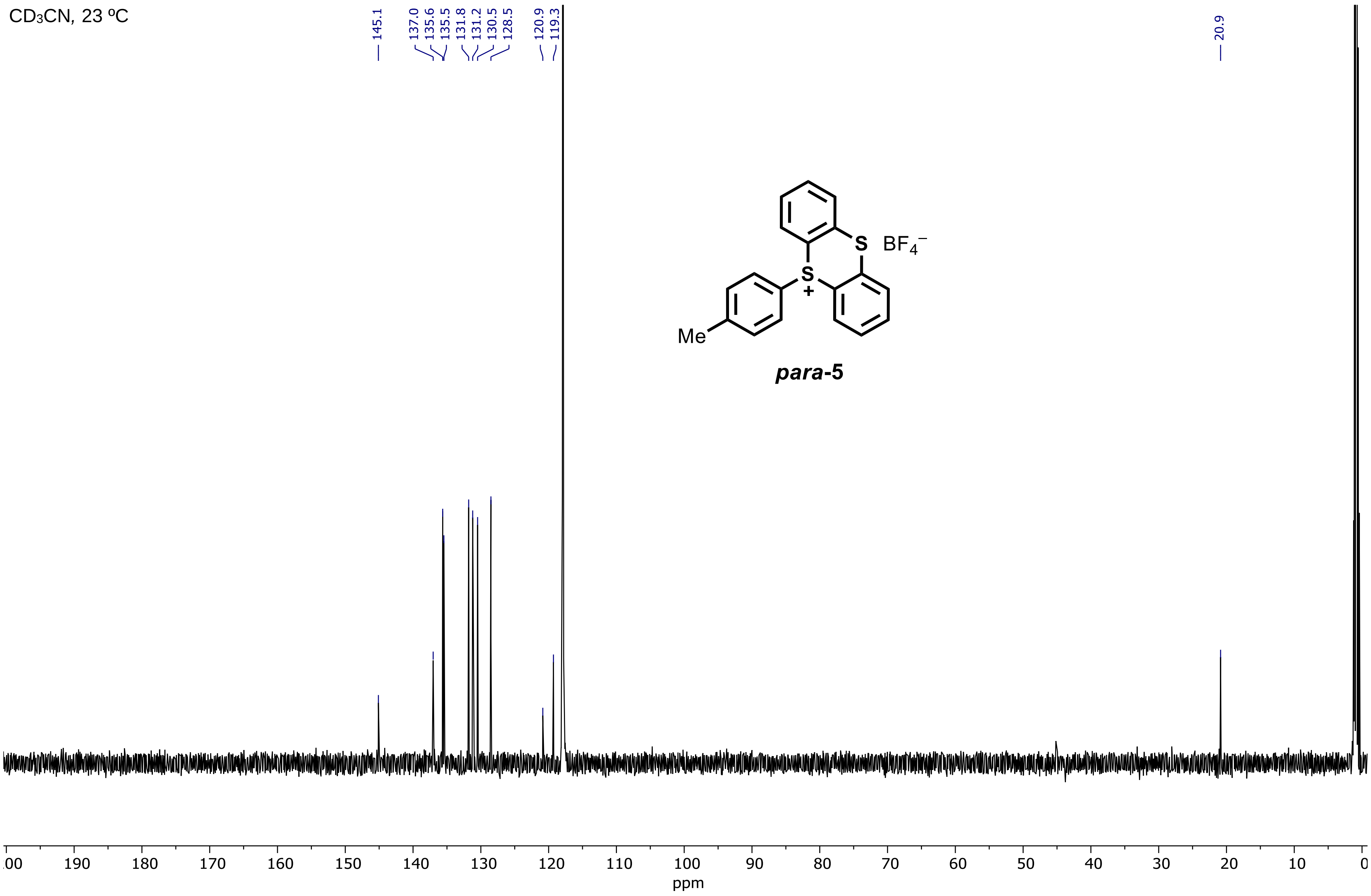

## $^1$H NMR of toluene derived thianthrenium salt 5, *meta*-isomer

CD3CN, 23 ºC

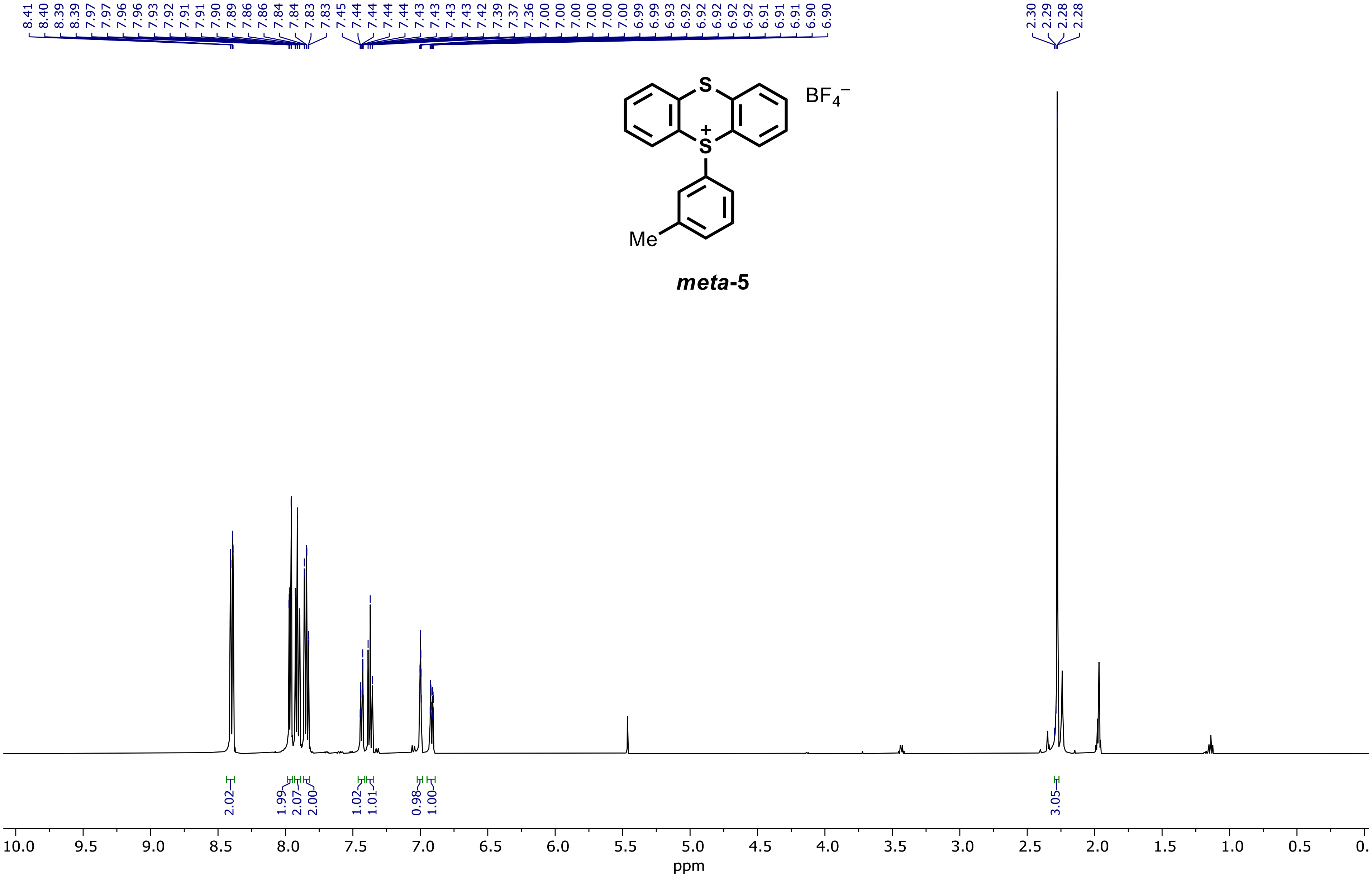


***meta*-5**

## $^{13}$C NMR of toluene derived thianthrenium salt 5, *meta*-isomer

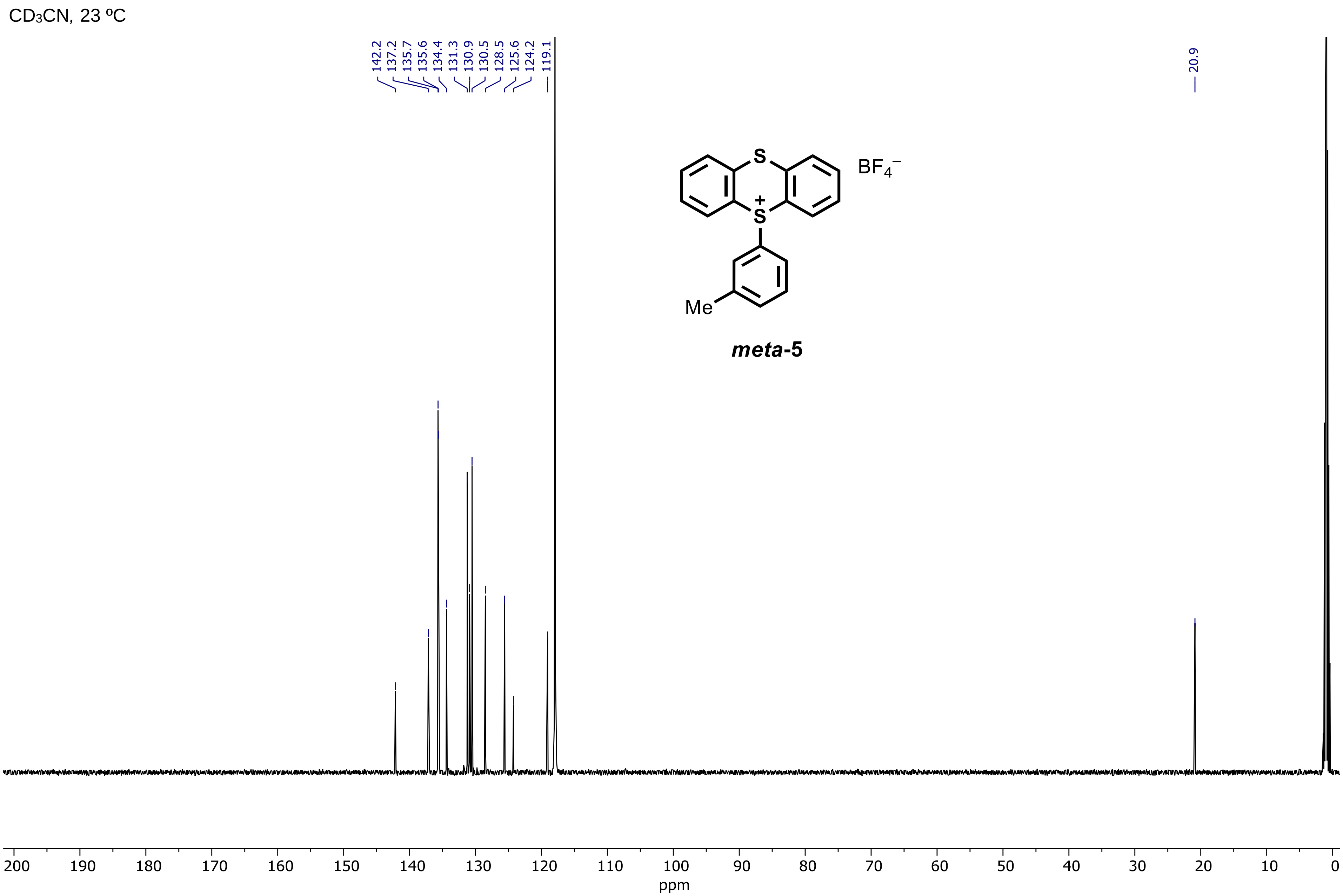


***meta*-5**

## $^{1}$H NMR of toluene derived thianthrenium salt 5, *ortho*-isomer

$CD_3CN$, 23 ºC

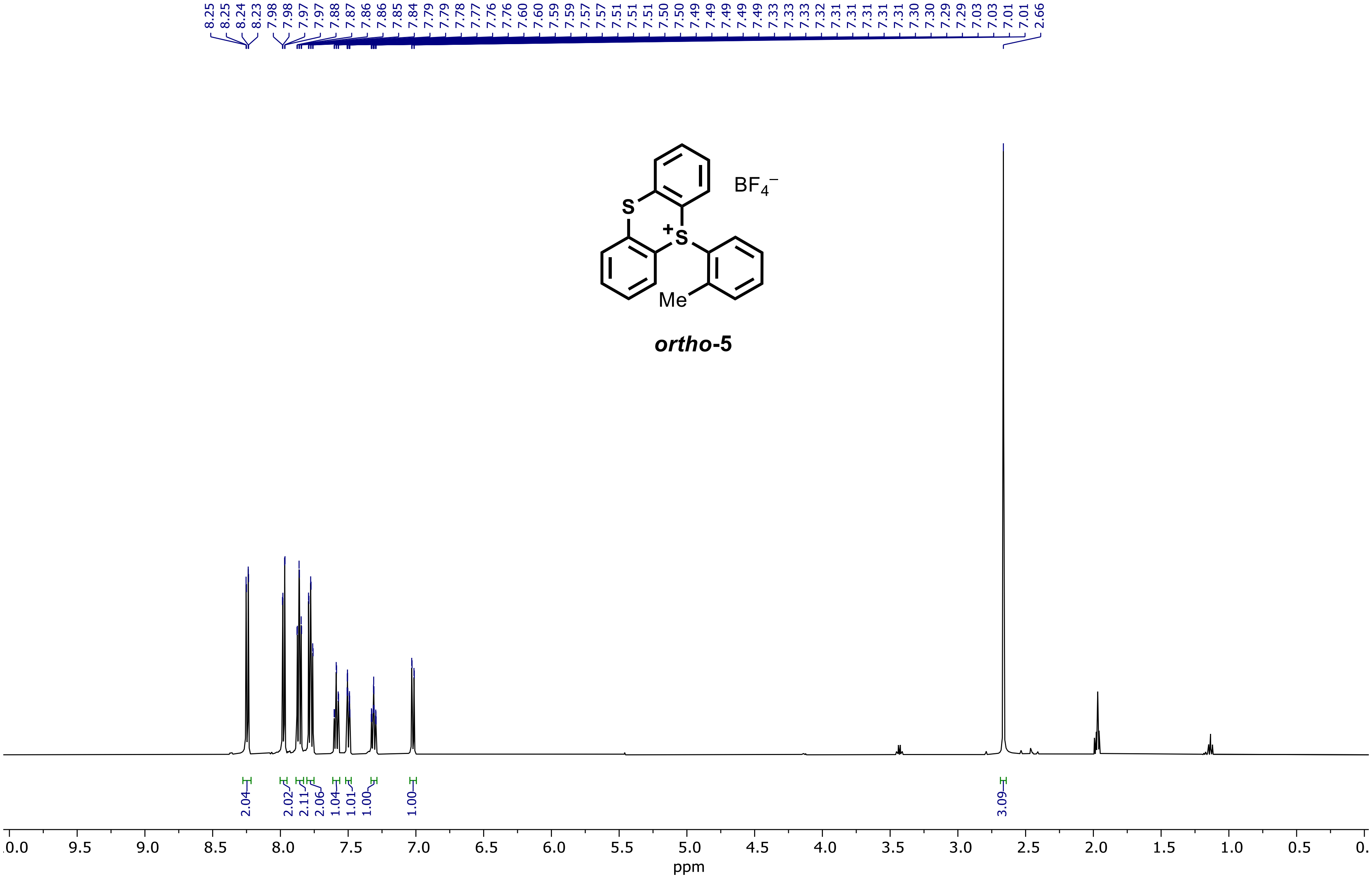

## $^{13}$C NMR of toluene derived thianthrenium salt 5, *ortho*-isomer

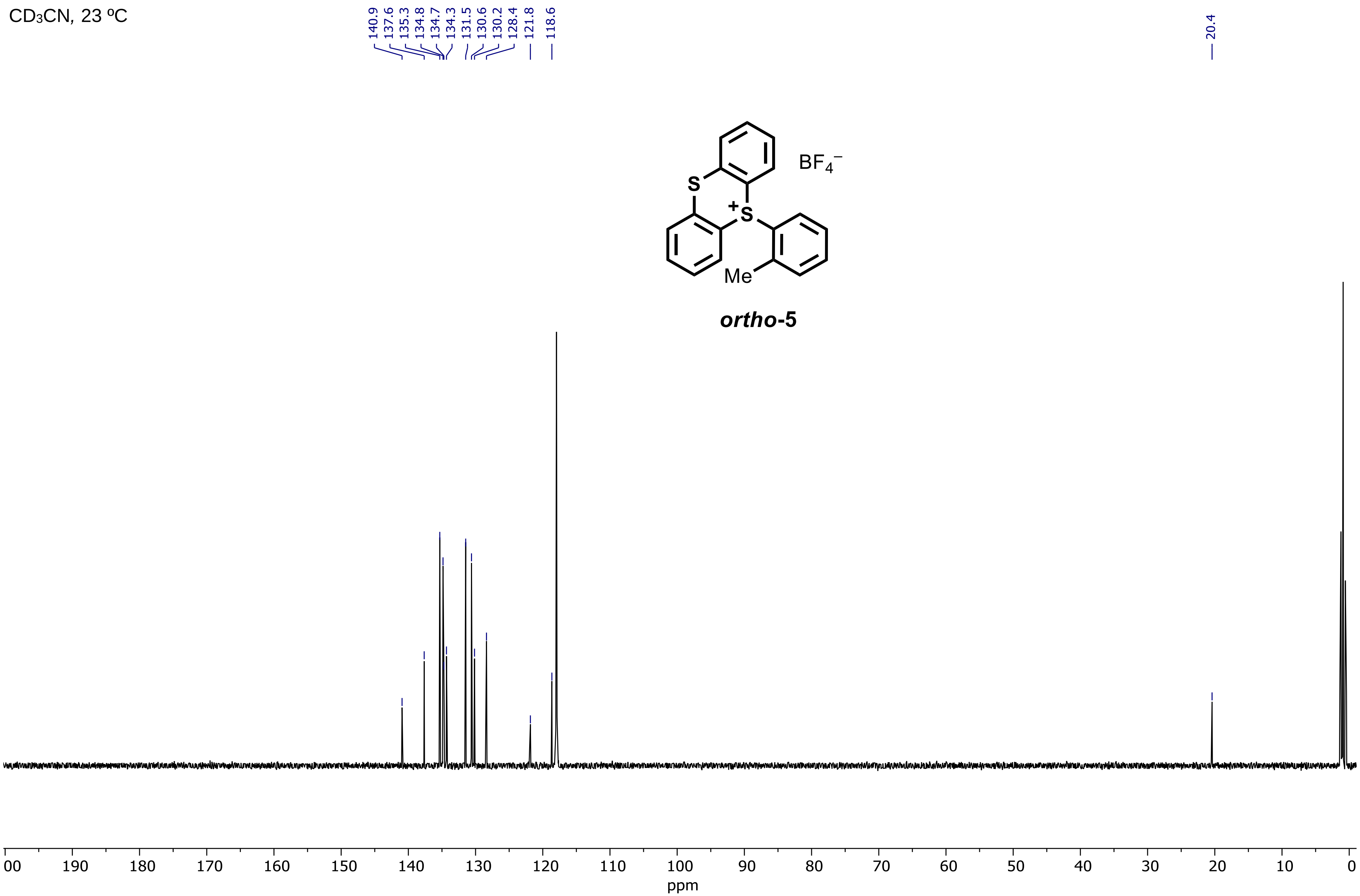

### [1]H NMR of indole derived thianthrenium salt S1

$CD_3CN$, 23 ºC

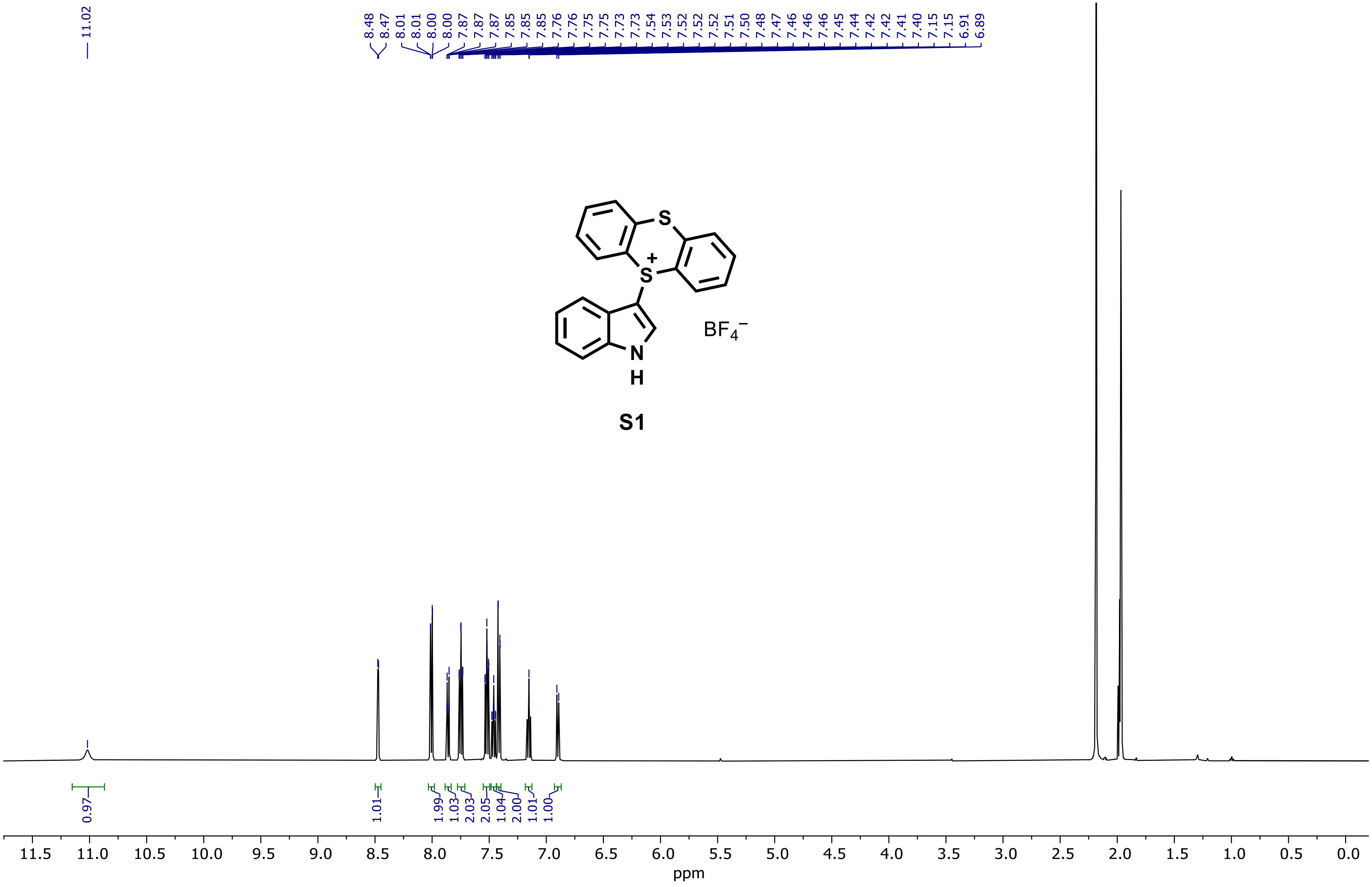

## $^{13}$C NMR of indole derived thianthrenium salt S1

$CD_3CN$, 23 ºC

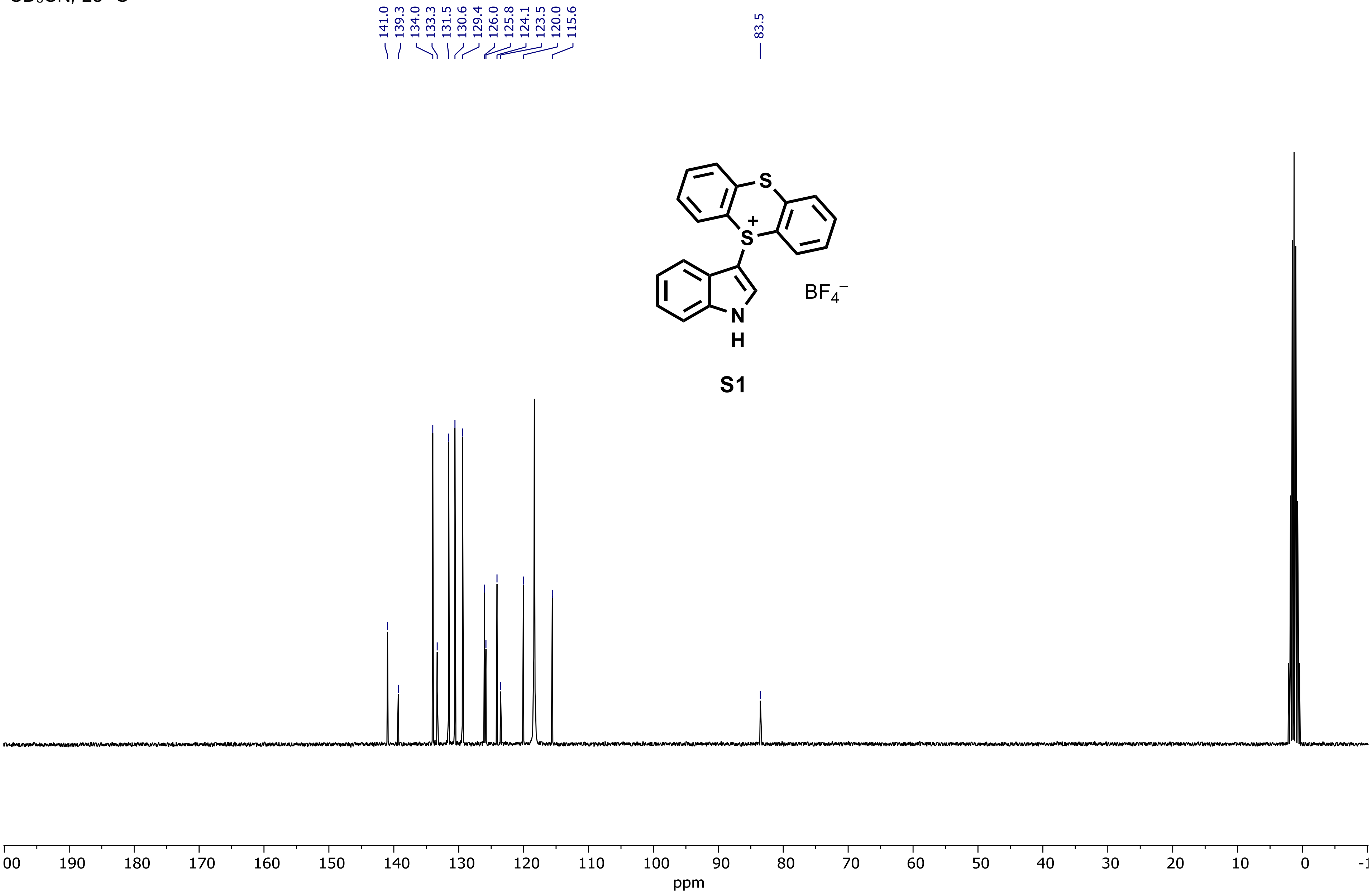

# $^1$H NMR of toluene derived tetrafluorothianthrenium salt S2, *para*-isomer

CD$_3$CN, 23 ºC

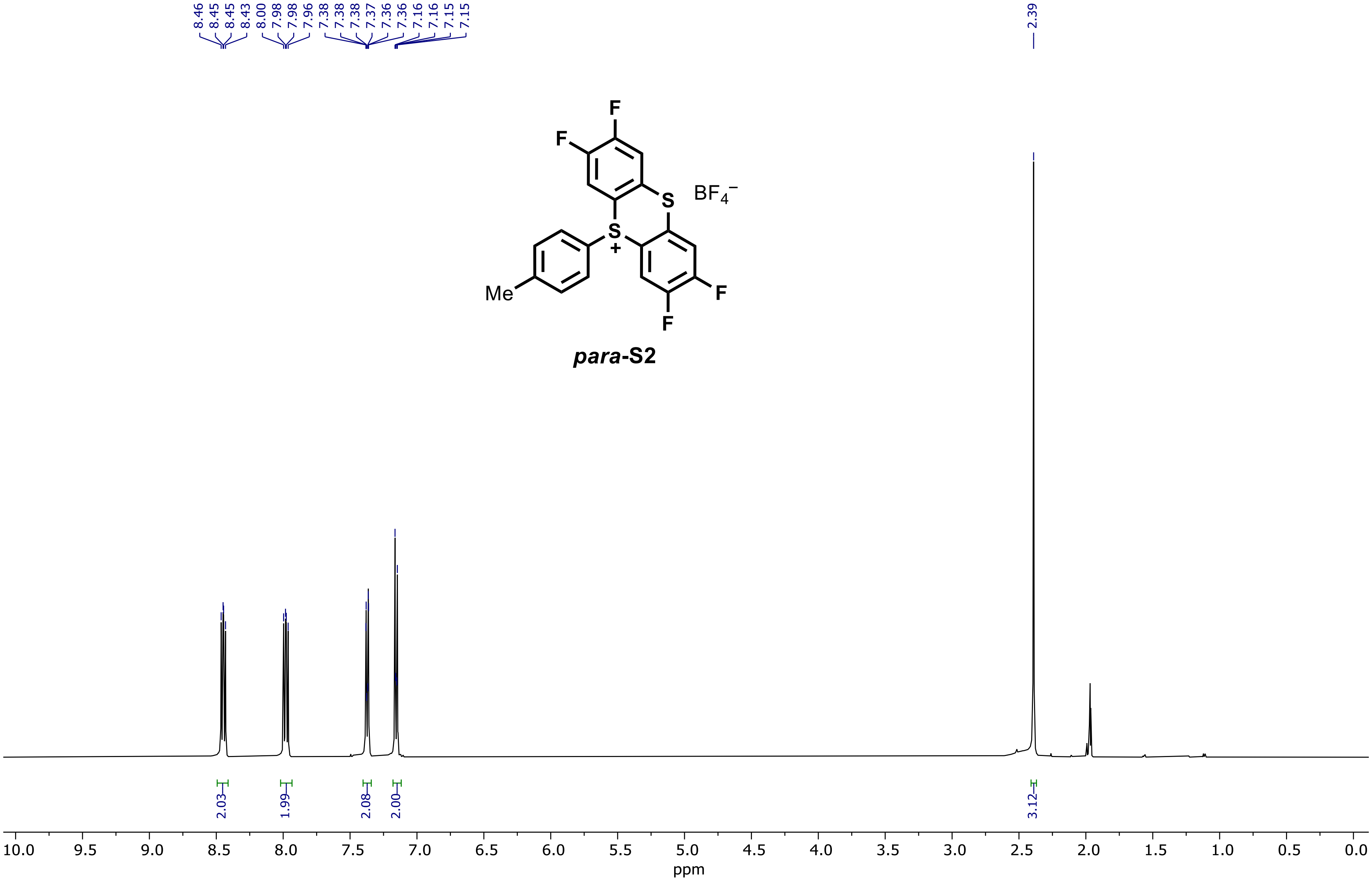


***para*-S2**

**[13]C NMR of toluene derived tetrafluorothianthrenium salt S2, *para*-isomer**

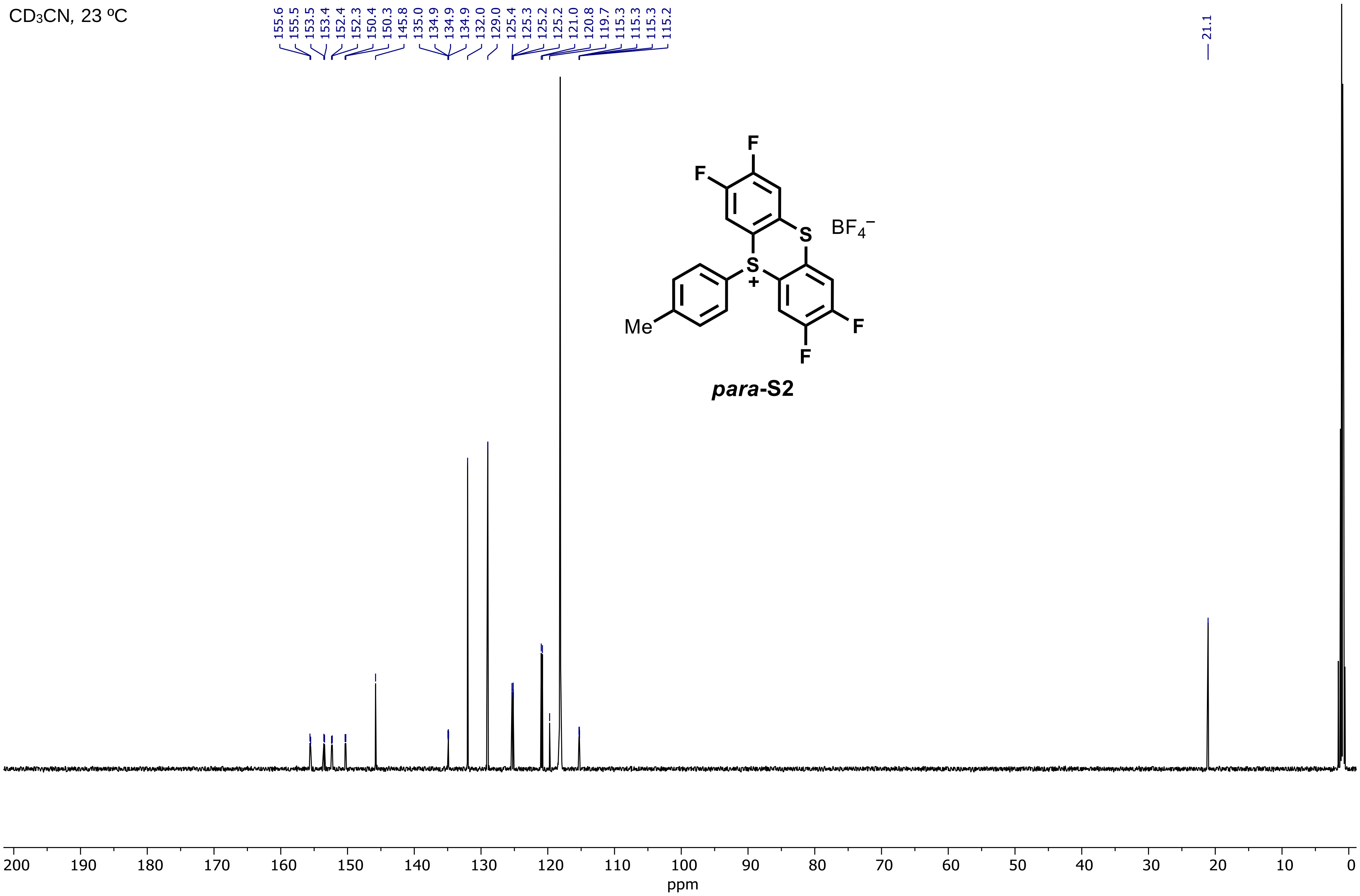


***para*-S2**

## $^{19}F$ NMR of toluene derived tetrafluorothianthrenium salt S2, *para*-isomer

$CD_3CN$, 23 ºC

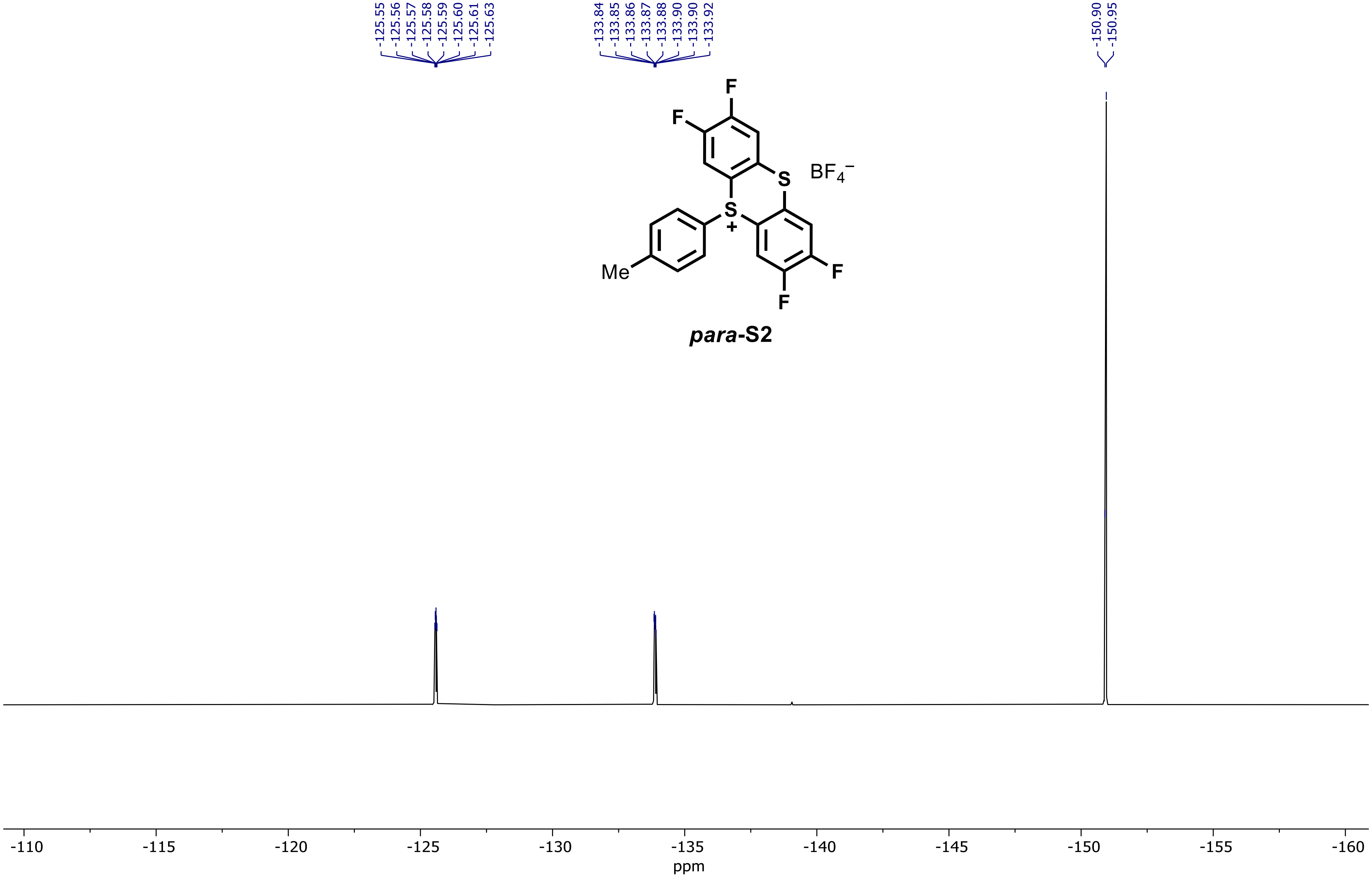


***para*-S2**

## $^{1}$H NMR of toluene derived tetrafluorothianthrenium salt S2, *meta*-isomer

CD3CN, 23 ºC

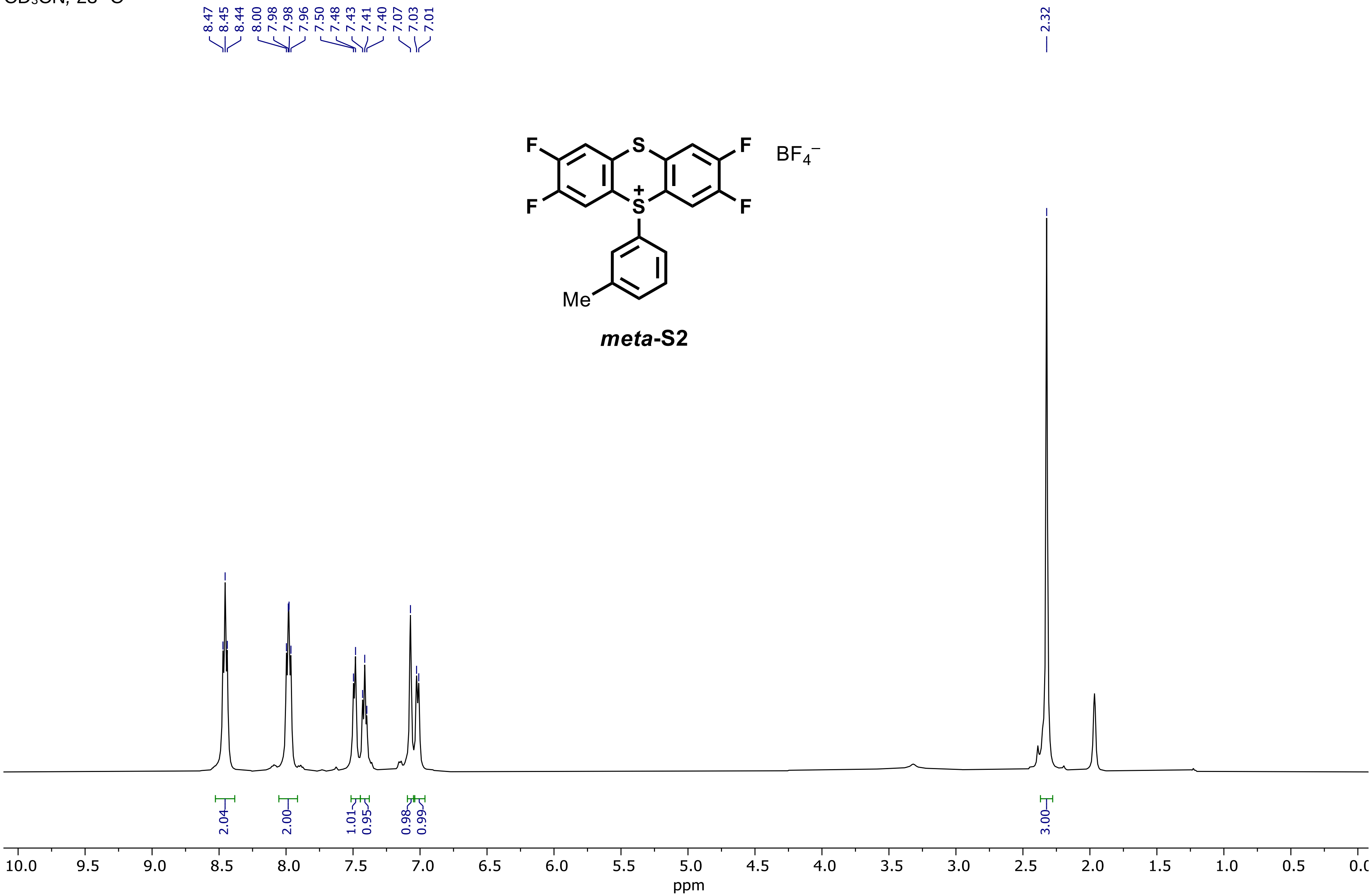


**_meta_-S2**

## $^{13}$C NMR of toluene derived tetrafluorothianthrenium salt S2, *meta*-isomer

$CD_3CN$, 23 ºC

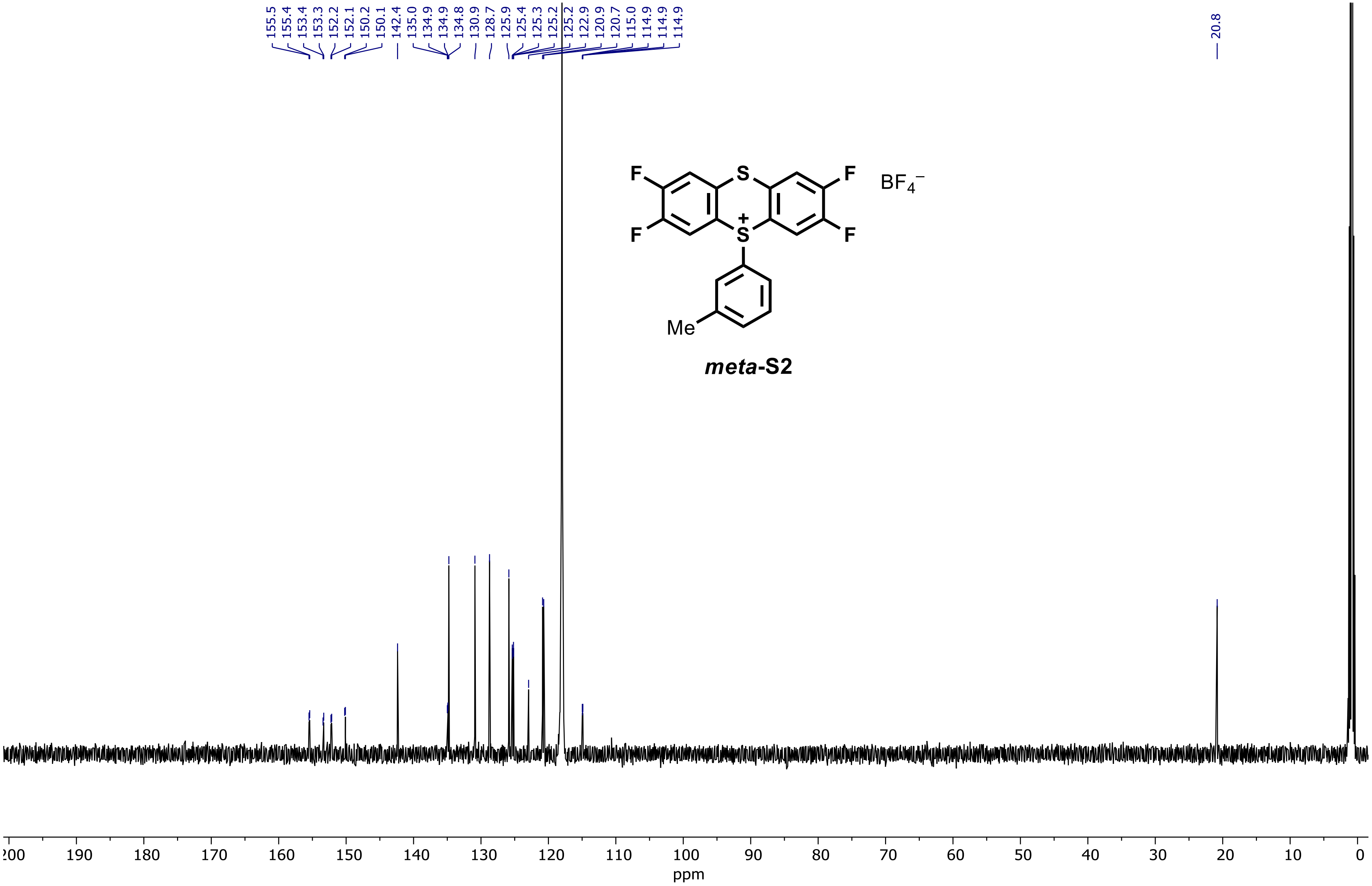

### $^{19}$F NMR of toluene derived tetrafluorothianthrenium salt S2, *meta*-isomer

$CD_3CN$, 23 ºC

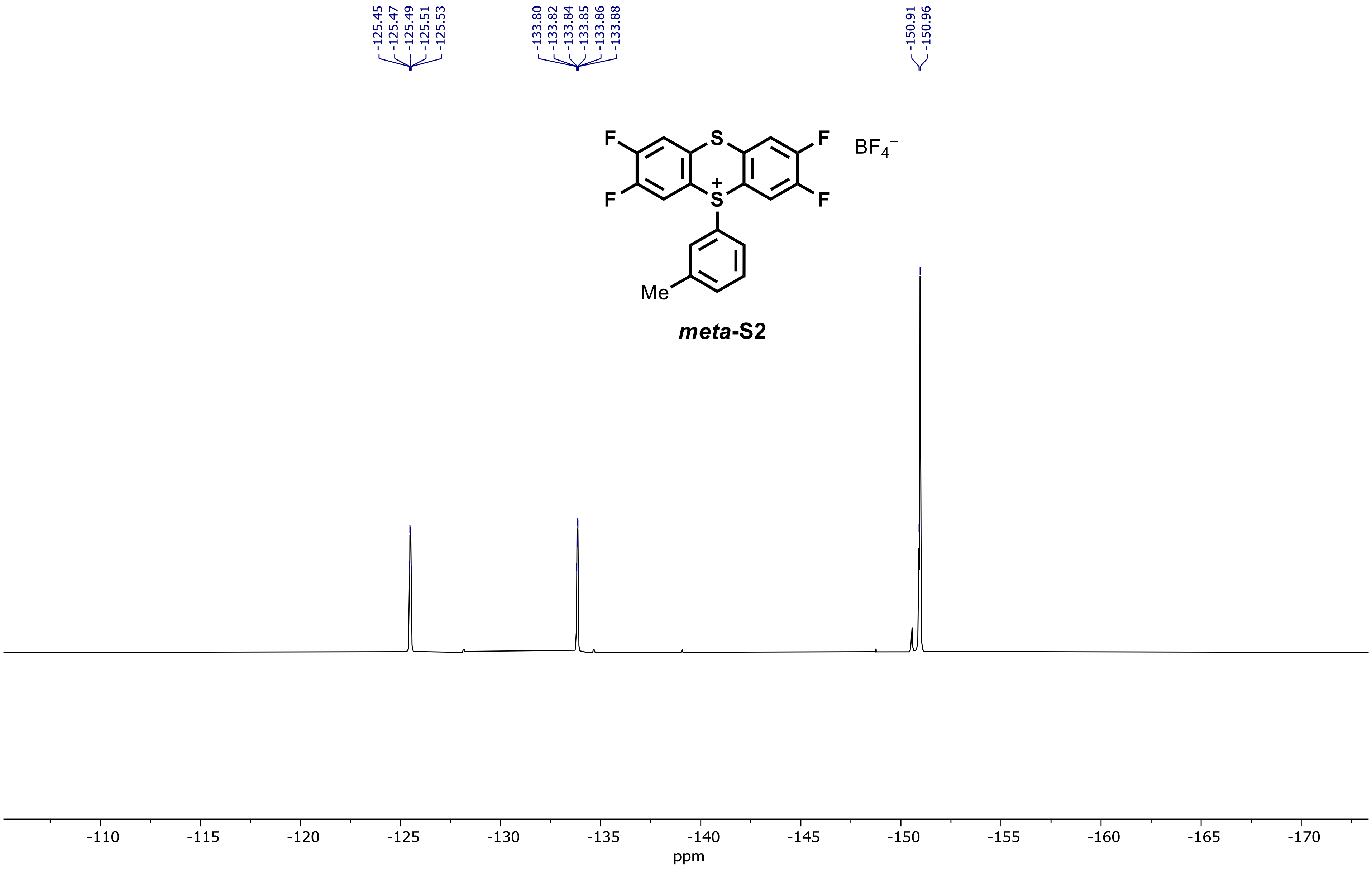


***meta*-S2**

### [1]H NMR of toluene derived tetrafluorothianthrenium salt S2, *ortho*- + *para*-isomer

$CD_3CN$, 23 ºC

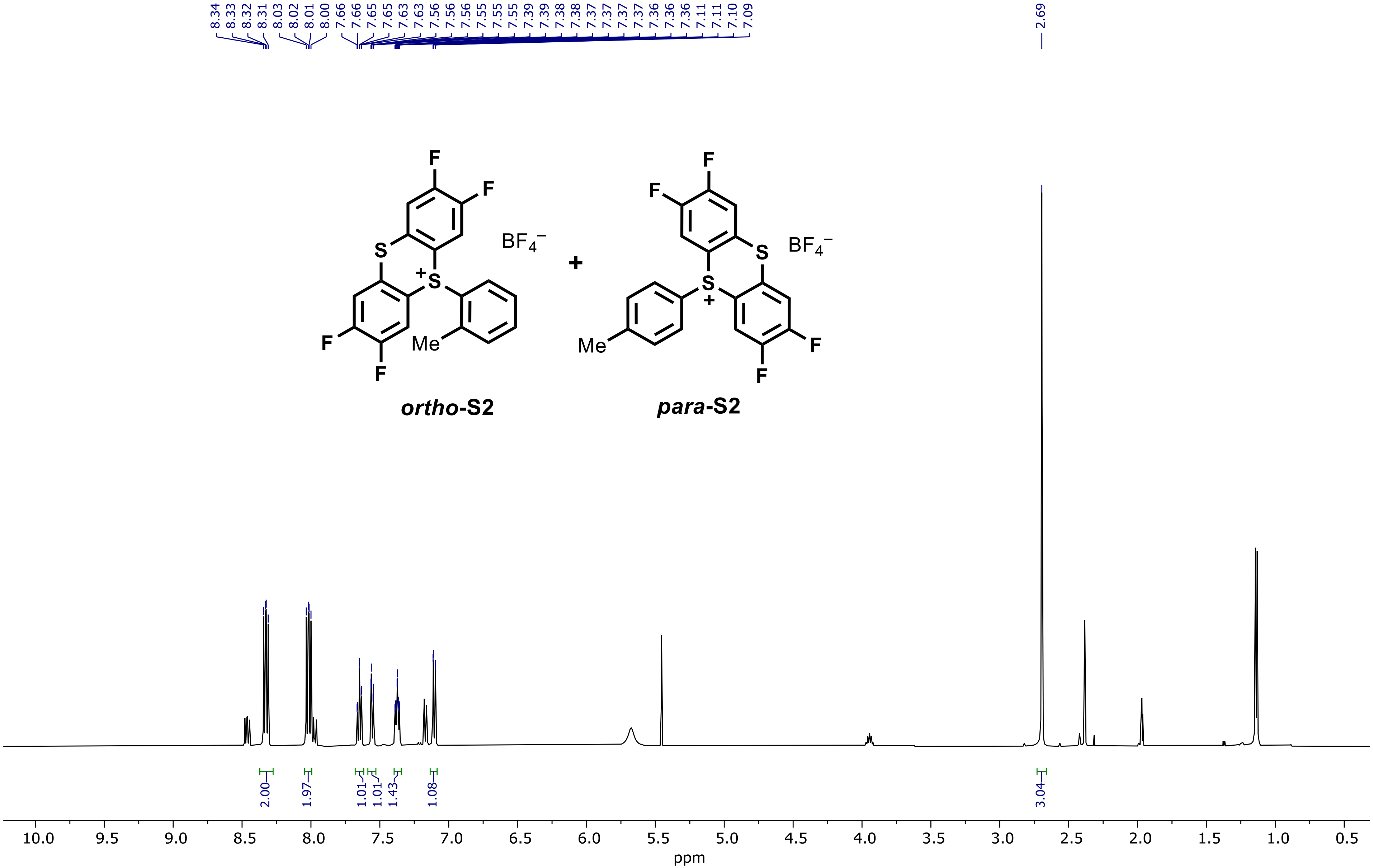

## [13]C NMR of toluene derived tetrafluorothianthrenium salt S2, *ortho*- + *para*-isomer

CD3CN, 23 ºC

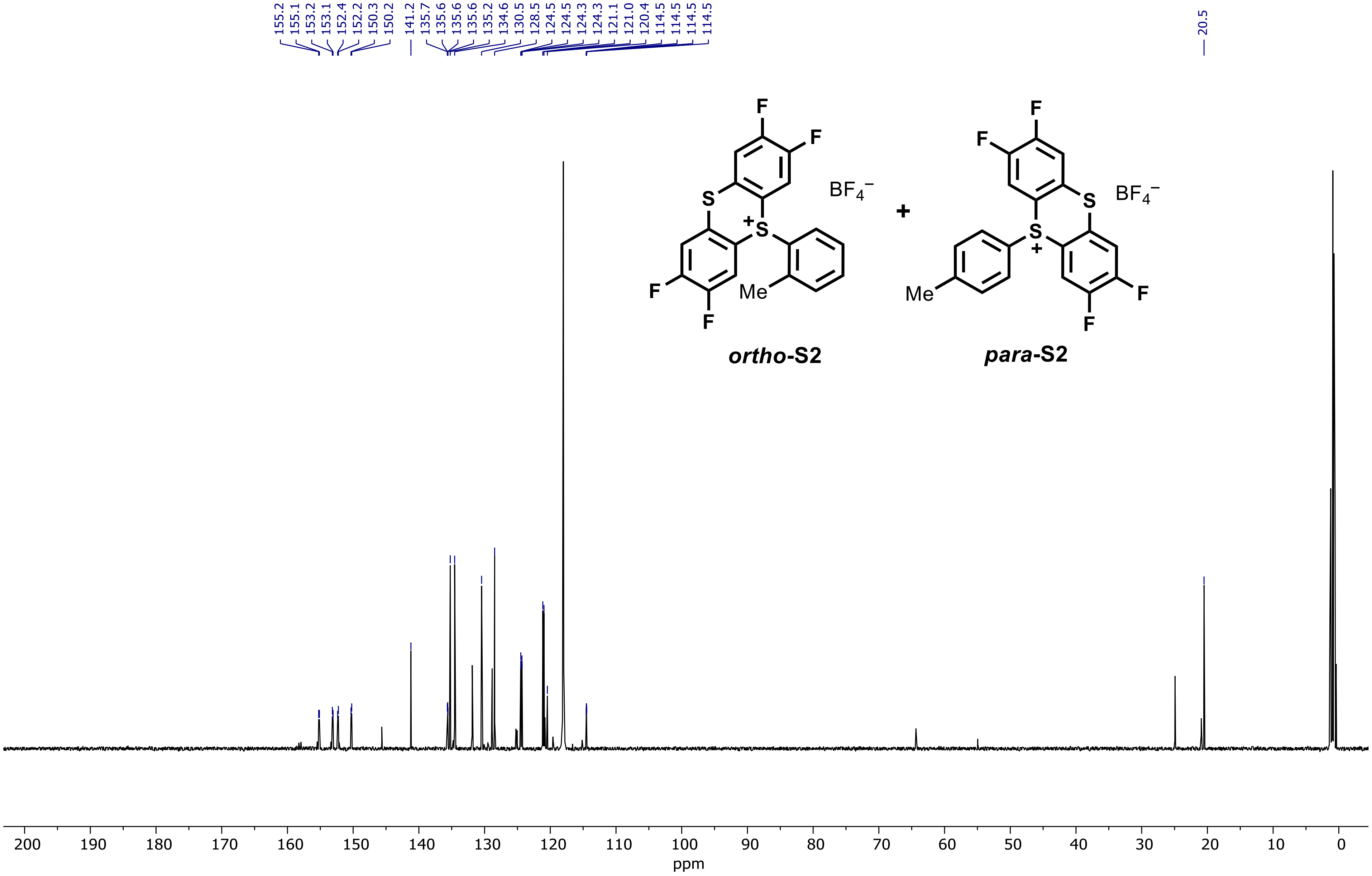

# $^{19}$F NMR of toluene derived tetrafluorothianthrenium salt S2, *ortho*- + *para*-isomer

CD3CN, 23 ºC

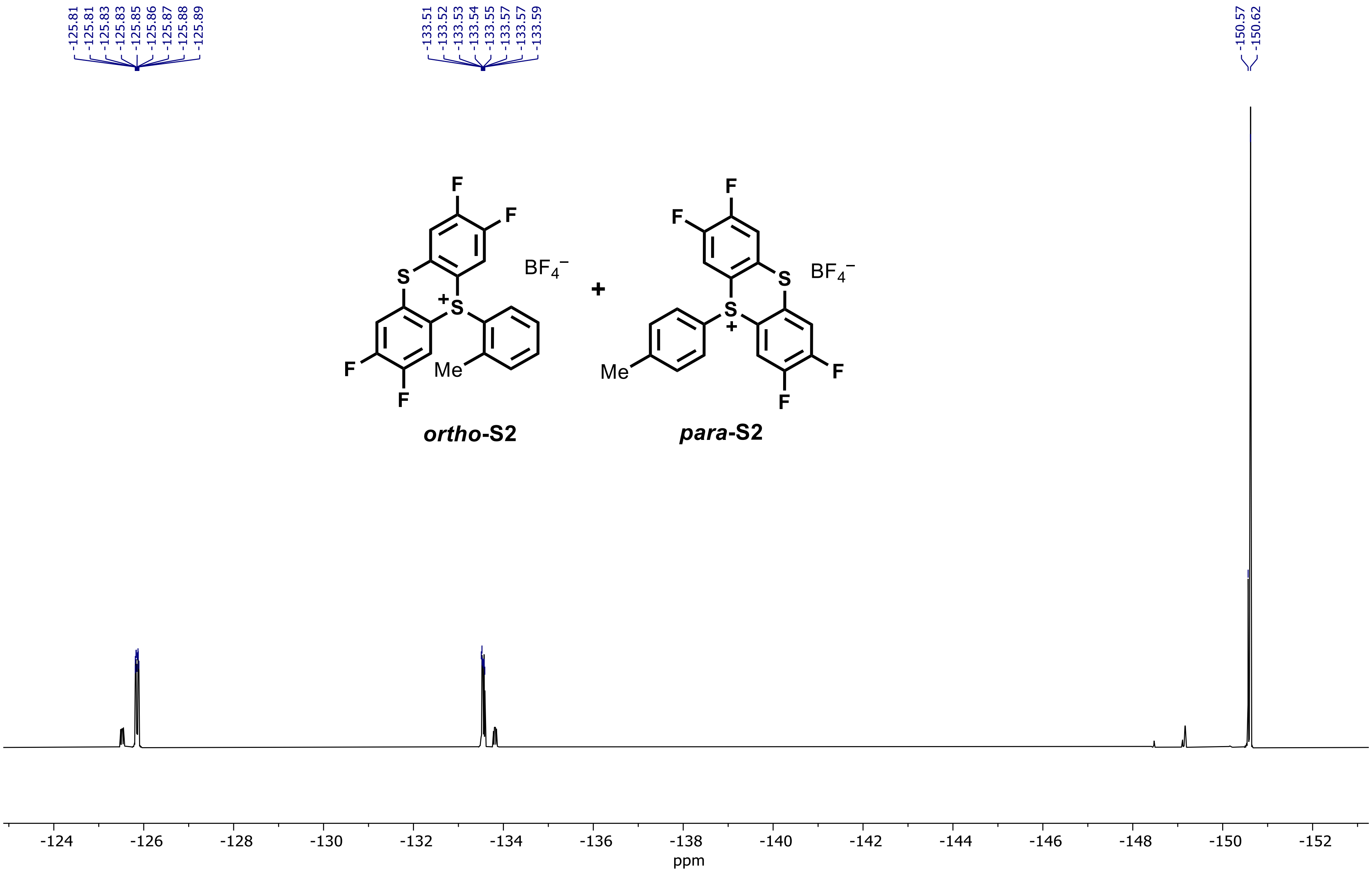

## $^1$H NMR of toluene derived phenoxathiinium salt S3, *para*-isomer

$CD_3CN$, 23 ºC

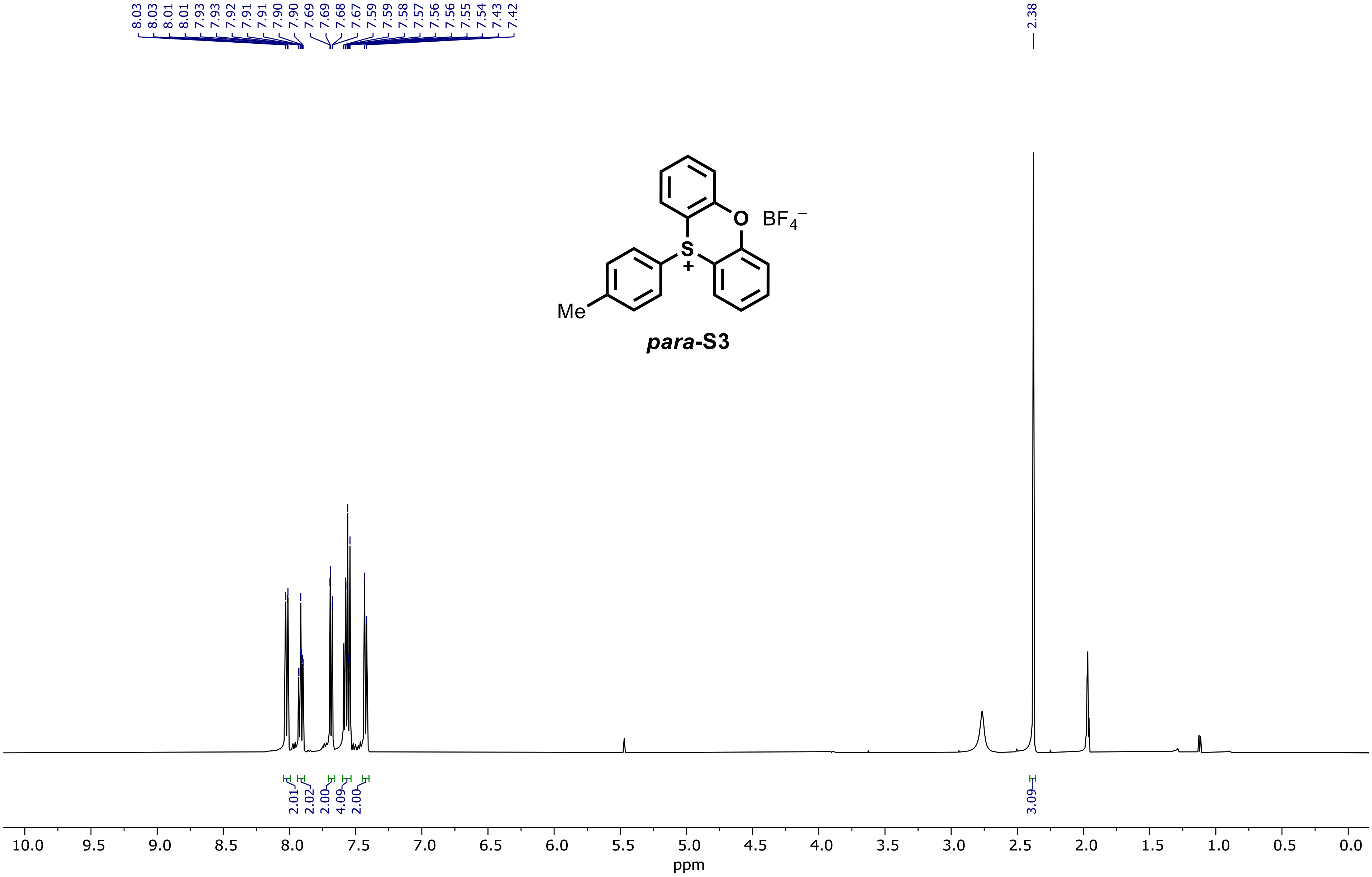

## $^{13}$C NMR of toluene derived phenoxathiinium salt S3, *para*-isomer

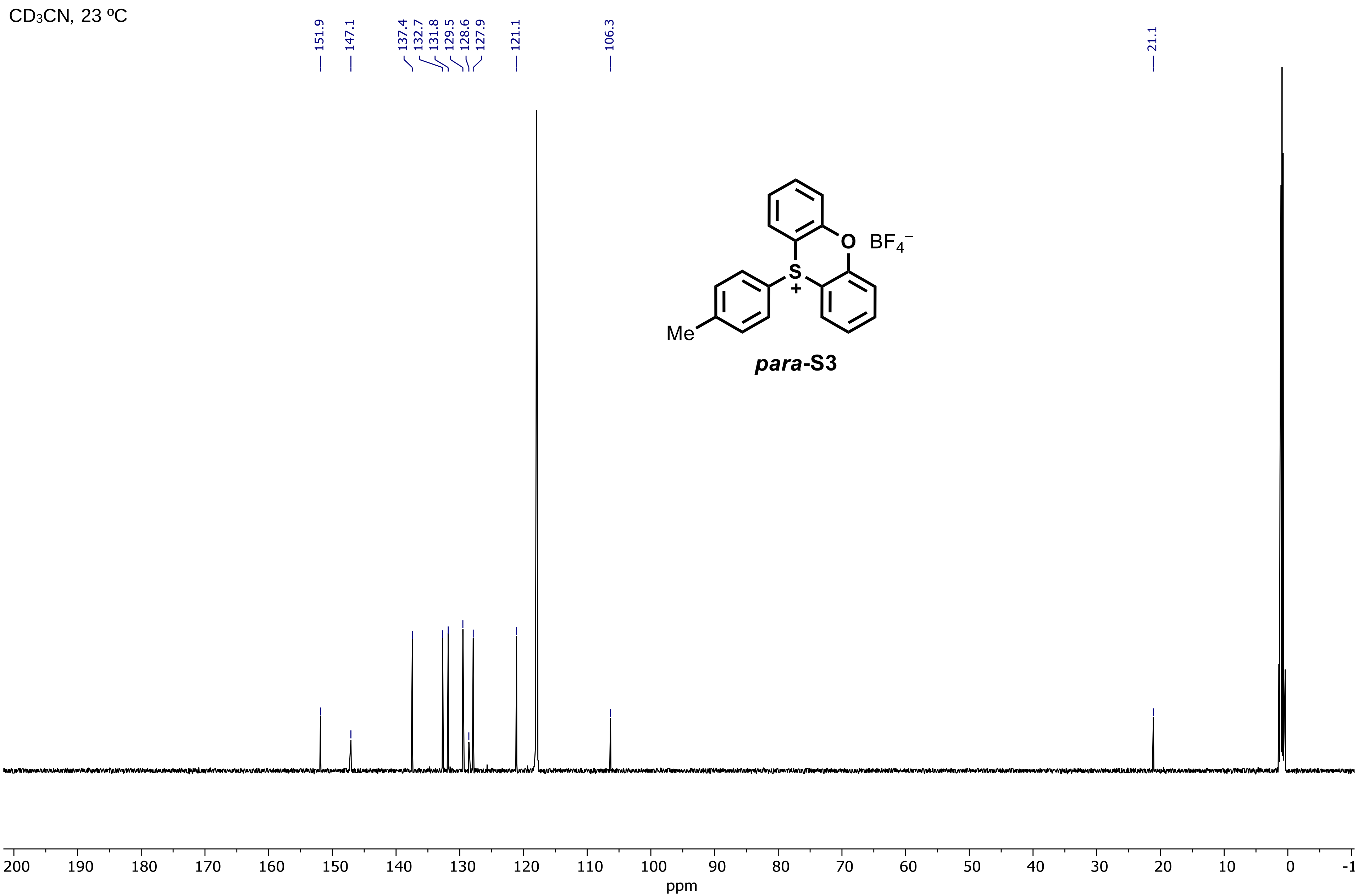

## $^{1}$H NMR of toluene derived phenoxathiinium salt S3, *meta*-isomer

$CD_3CN$, 23 ºC

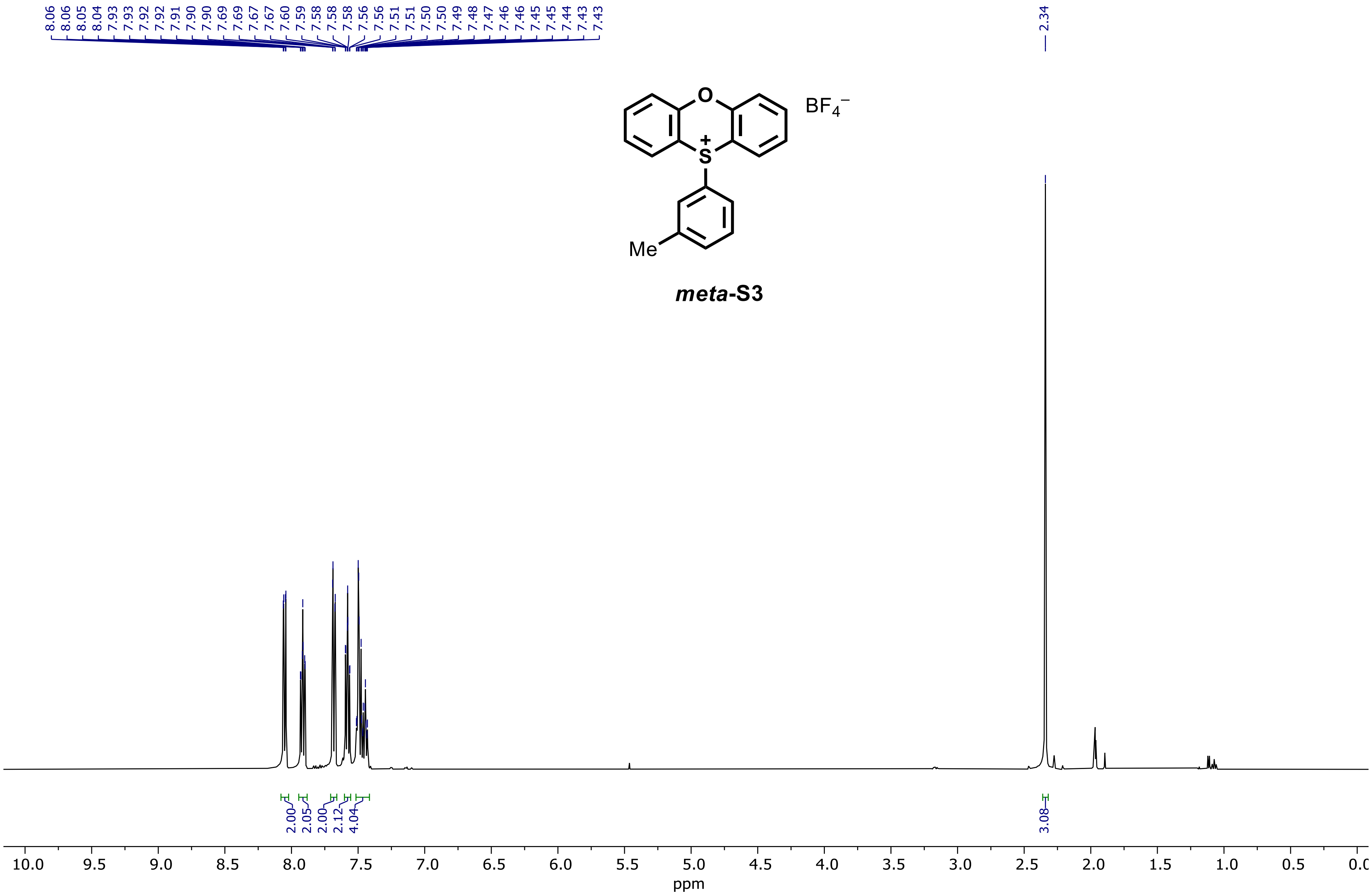

### $^{13}C$ NMR of toluene derived phenoxathiinium salt S3, *meta*-isomer

$CD_3CN$, 23 ºC

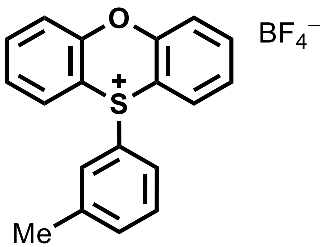


***meta*-S3**

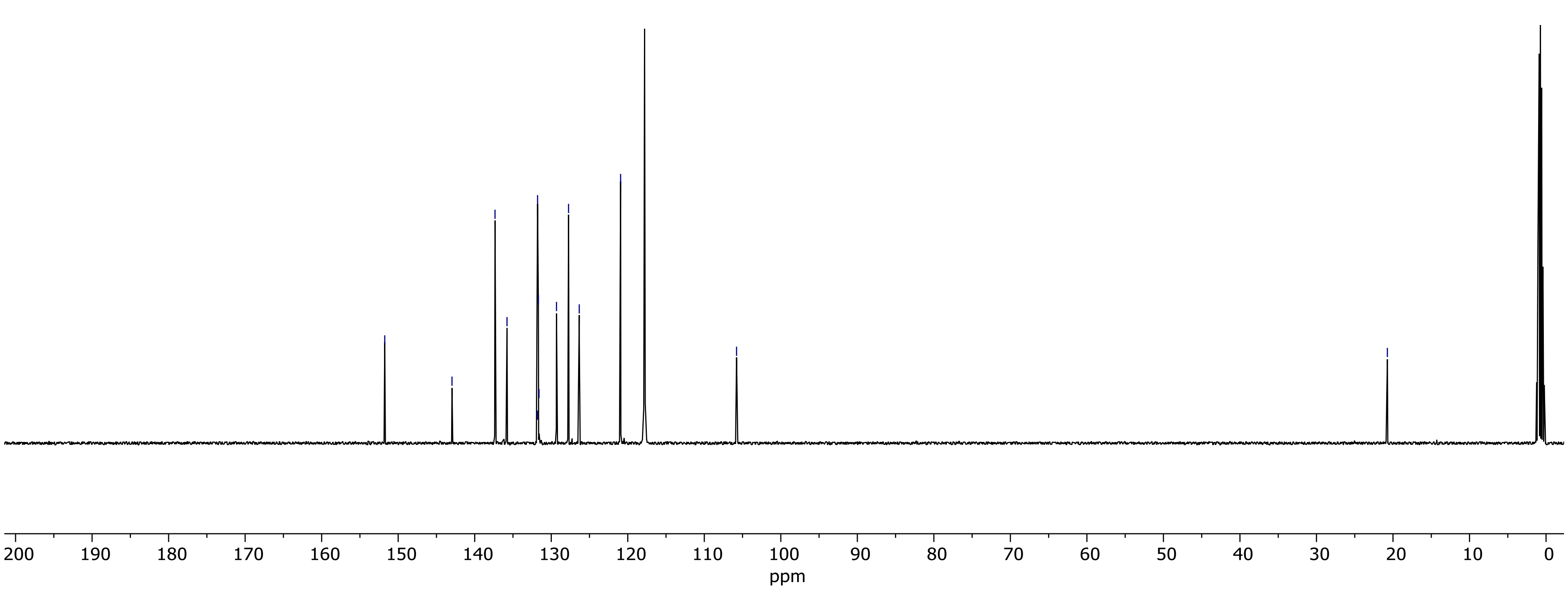

## $^{1}$H NMR of toluene derived phenoxathiinium salt S3, *ortho*-isomer

CD$_3$CN, 23 ºC

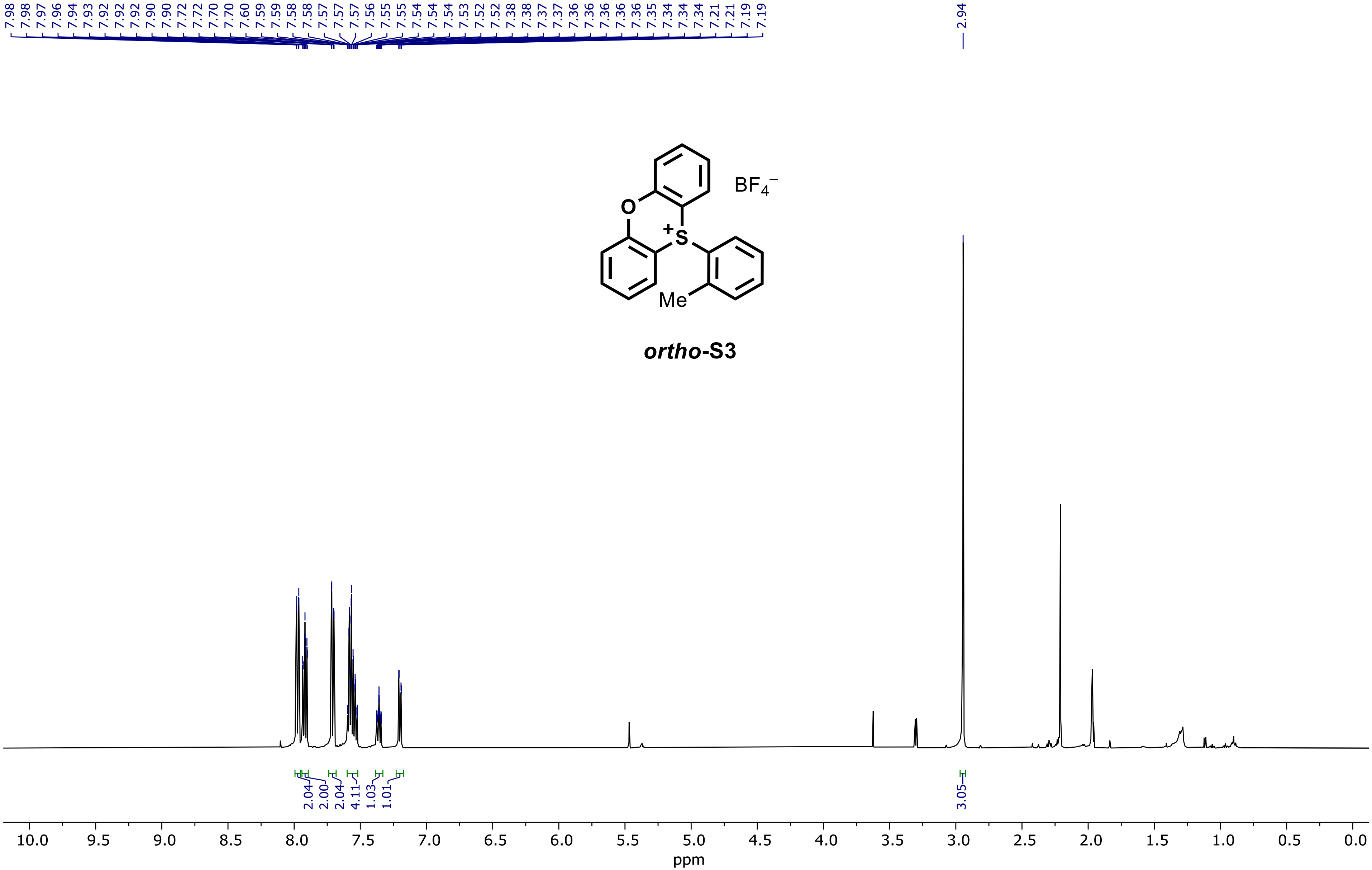

**$^{13}$C NMR of toluene derived phenoxathiinium salt S3, *ortho*-isomer**

CD$_3$CN, 23 ºC

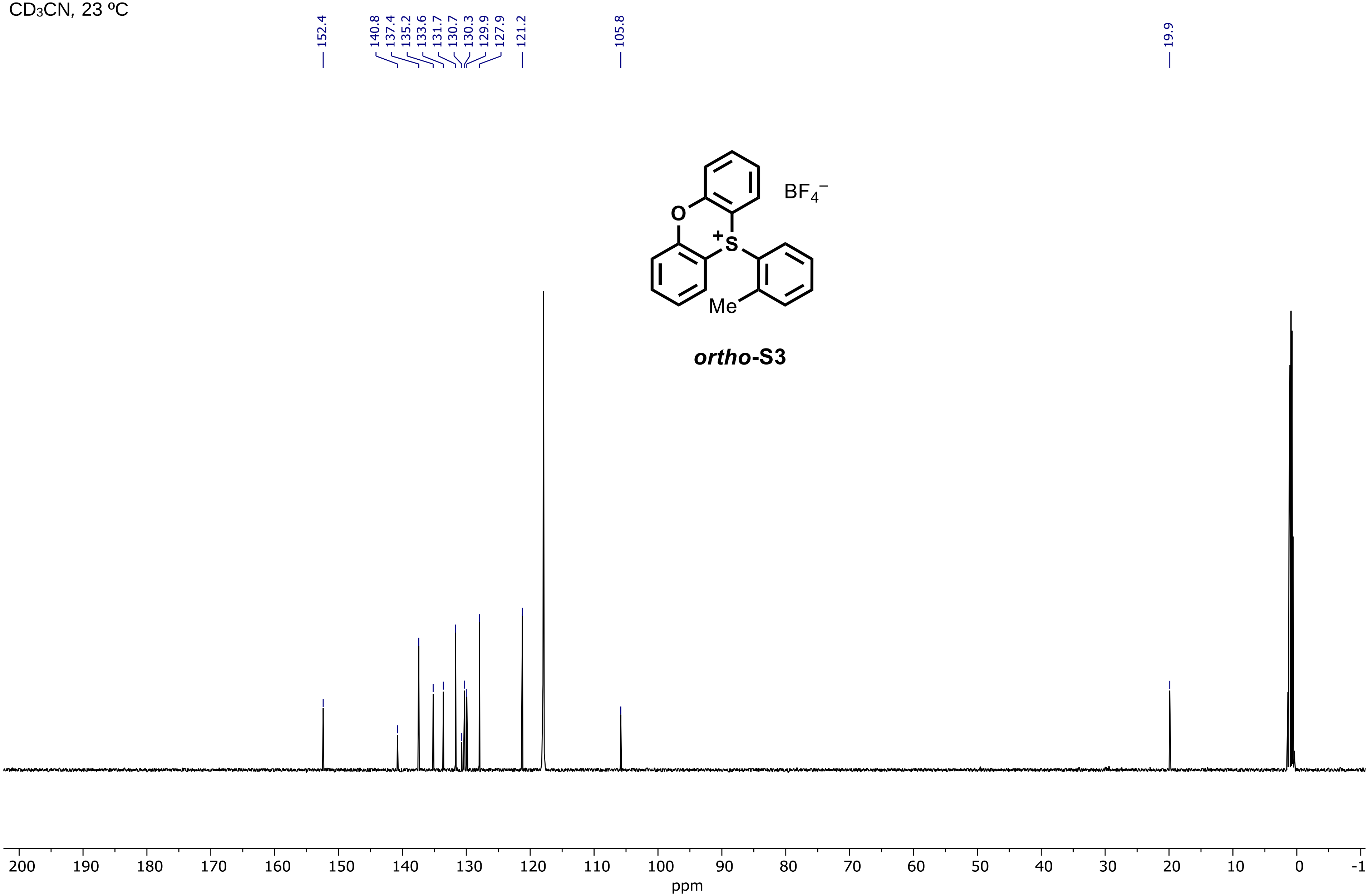

## $^{1}$H NMR of toluene derived dibenzothiophenium salt S4, *para*-isomer

CD$_3$CN, 23 ºC

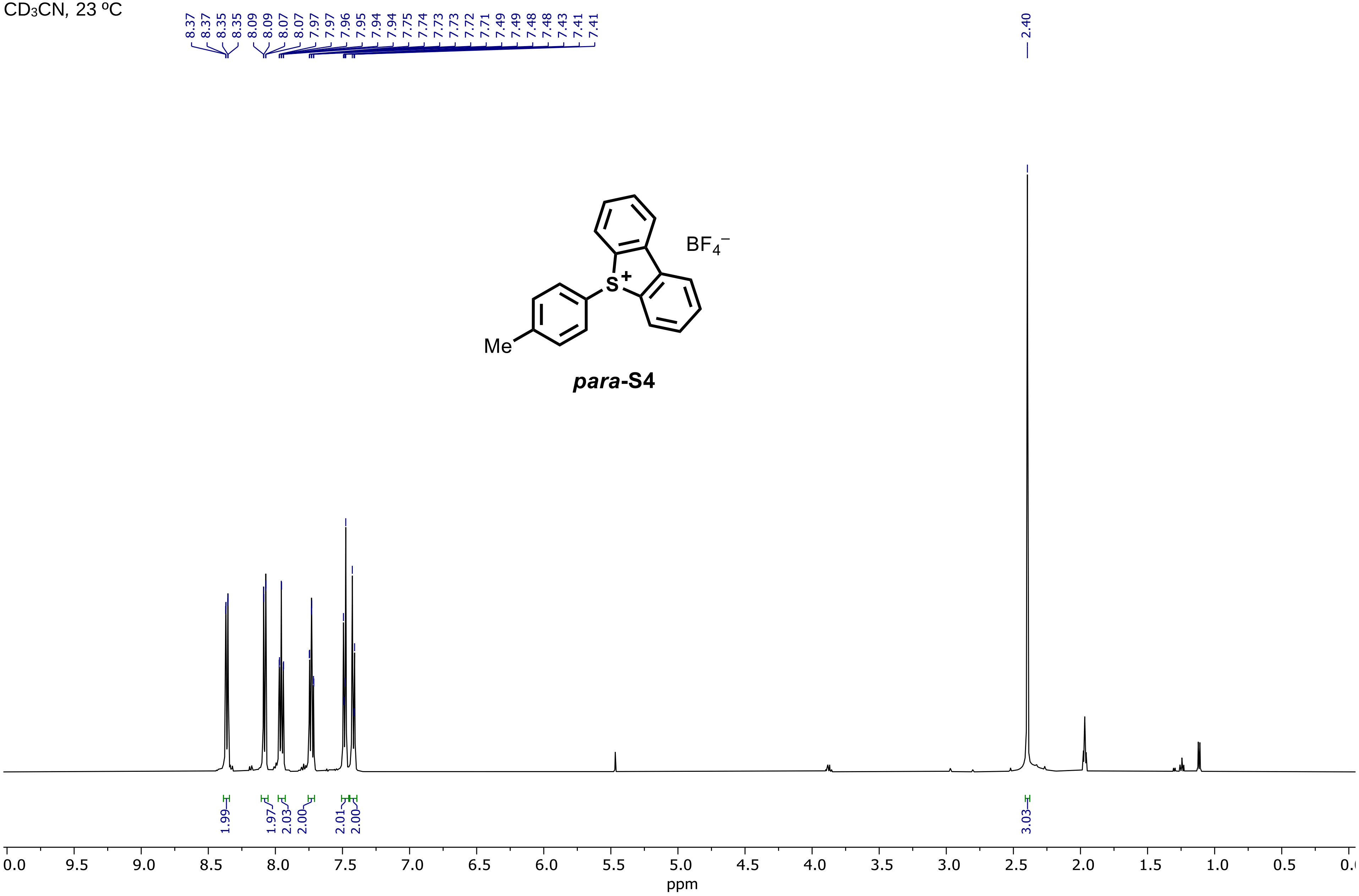

### $^{13}$C NMR of toluene derived dibenzothiophenium salt salt S4, *para*-isomer

$CD_3CN$, 23 ºC

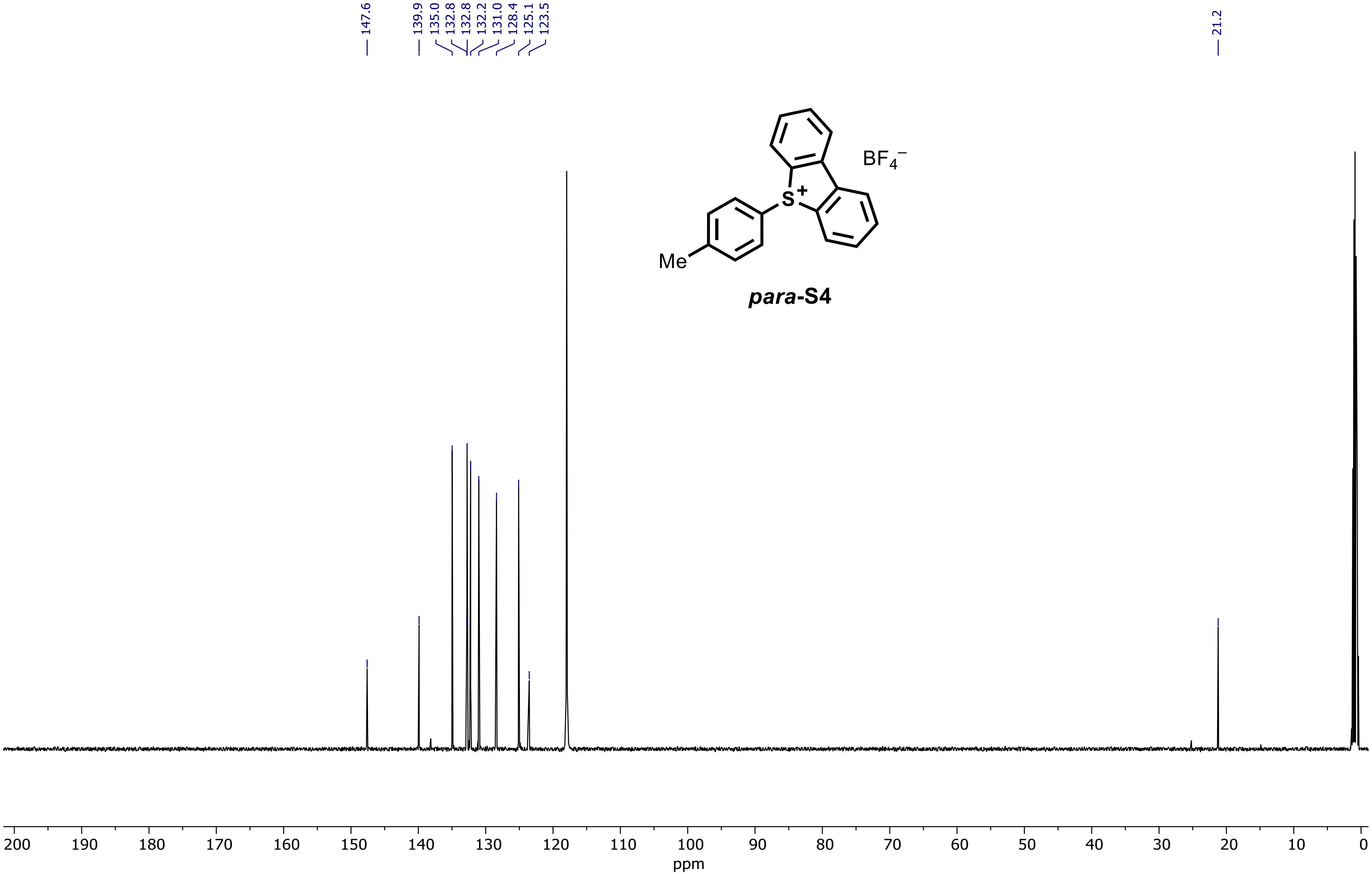

## [1]H NMR of toluene derived dibenzothiophenium salt S4, *meta*-isomer

$CD_3CN$, 23 ºC

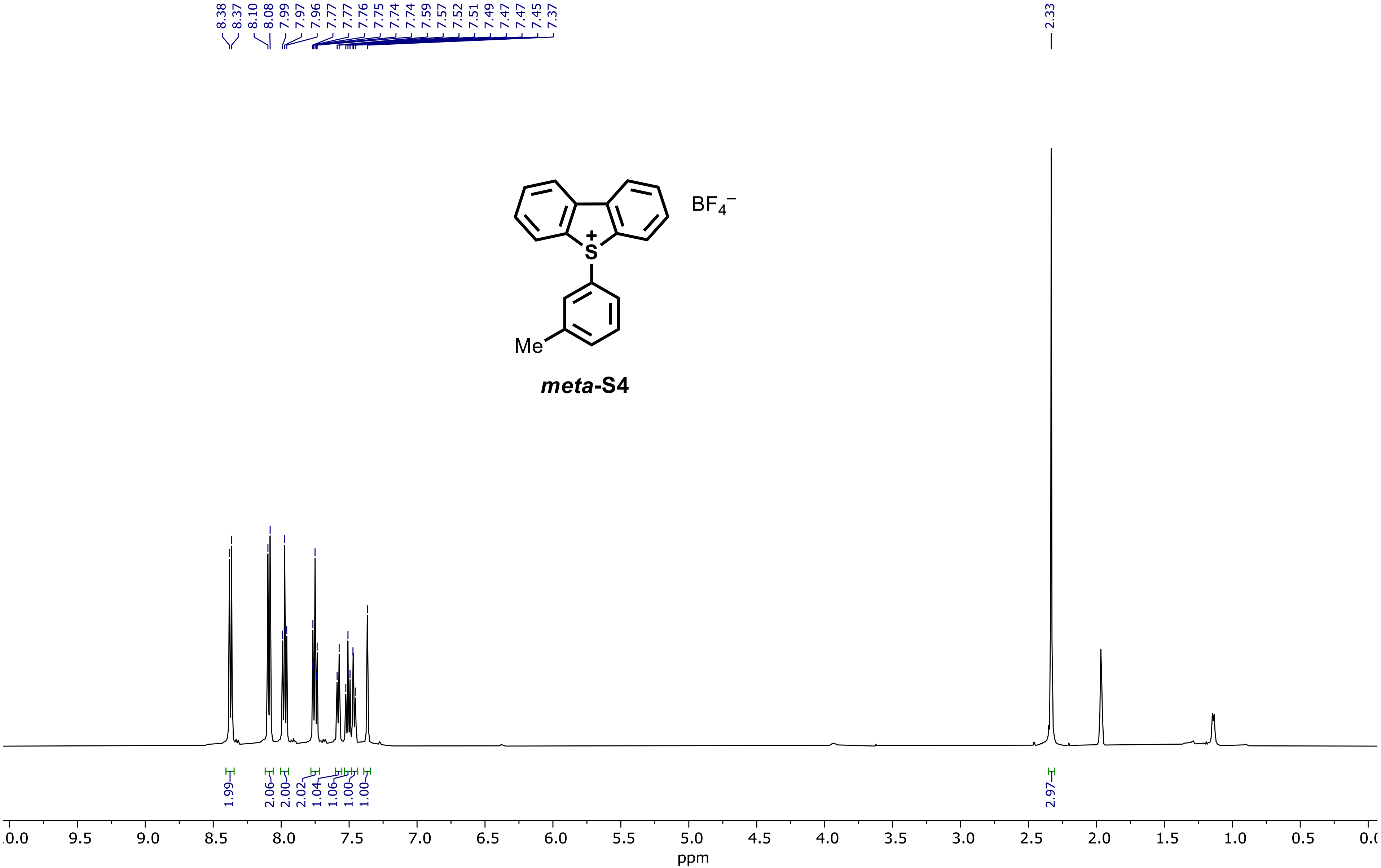


***meta*-S4**

### $^{13}$C NMR of toluene derived dibenzothiophenium salt S4, *meta*-isomer

CD$_3$CN, 23 ºC

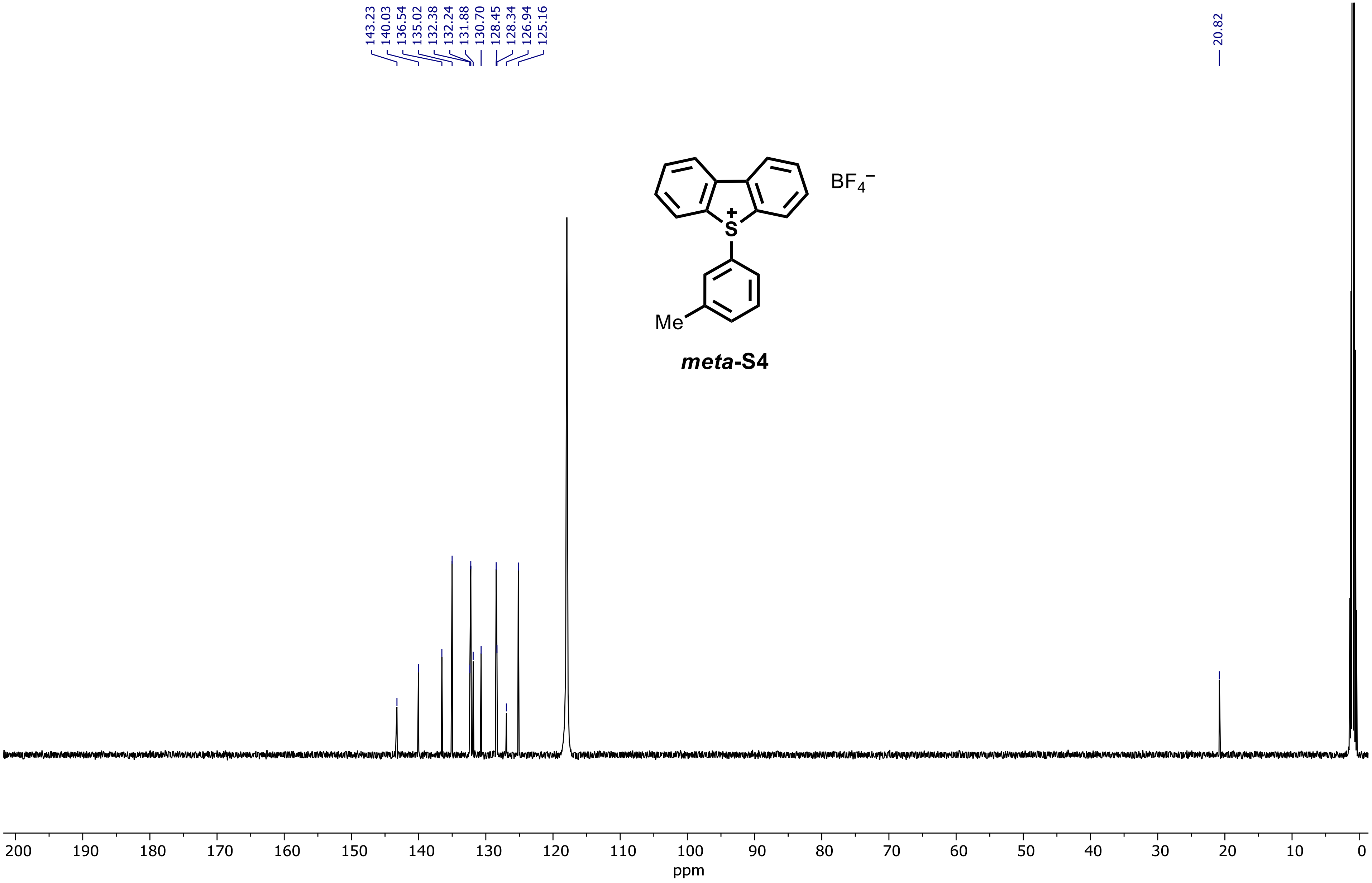

## $^1$H NMR of toluene derived dibenzothiophenium salt S4, *ortho*-isomer

$CD_3CN$, 23 ºC

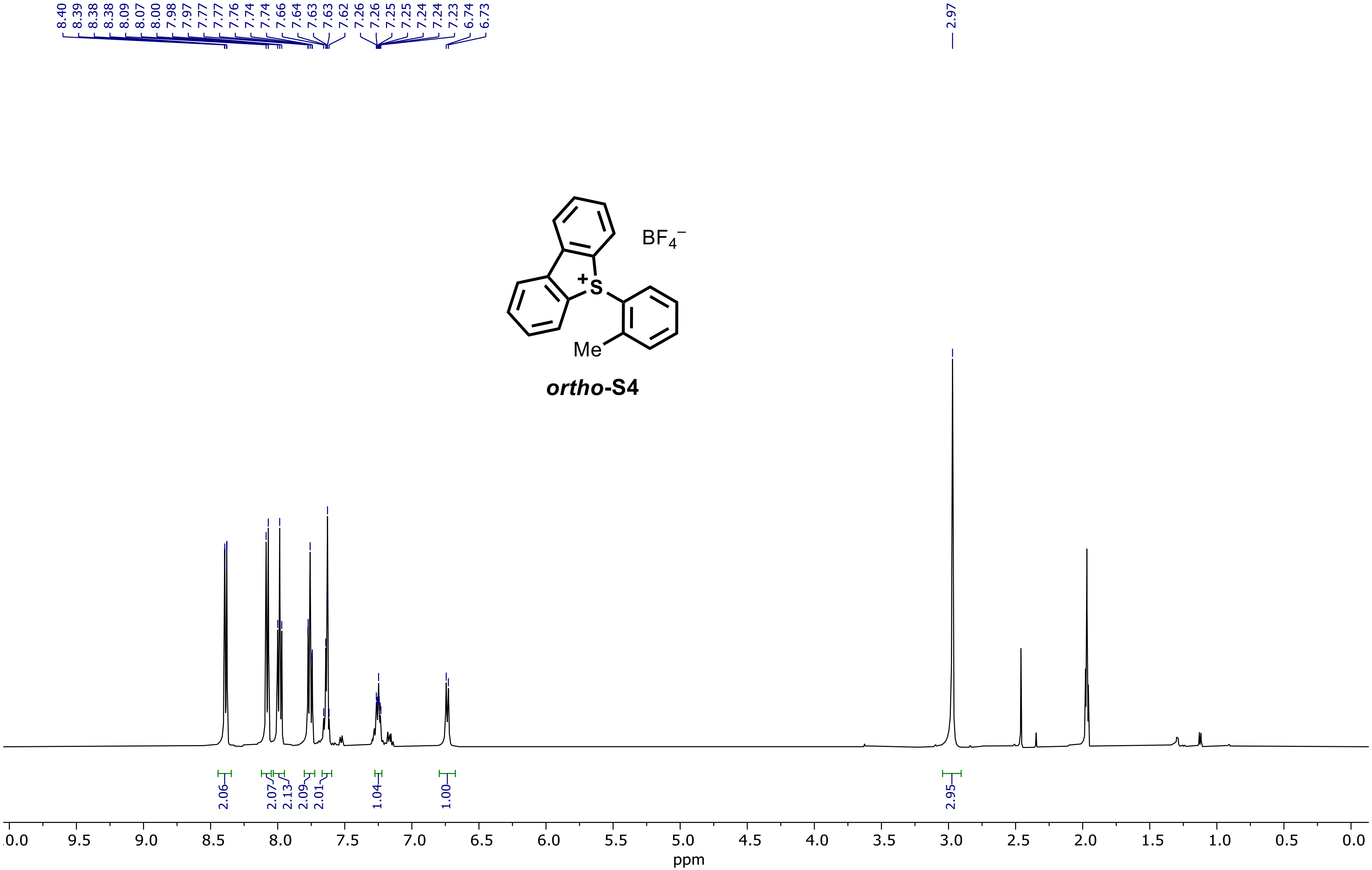

## $^{13}$C NMR of toluene derived dibenzothiophenium salt S4, *ortho*-isomer

CD$_3$CN, 23 ºC

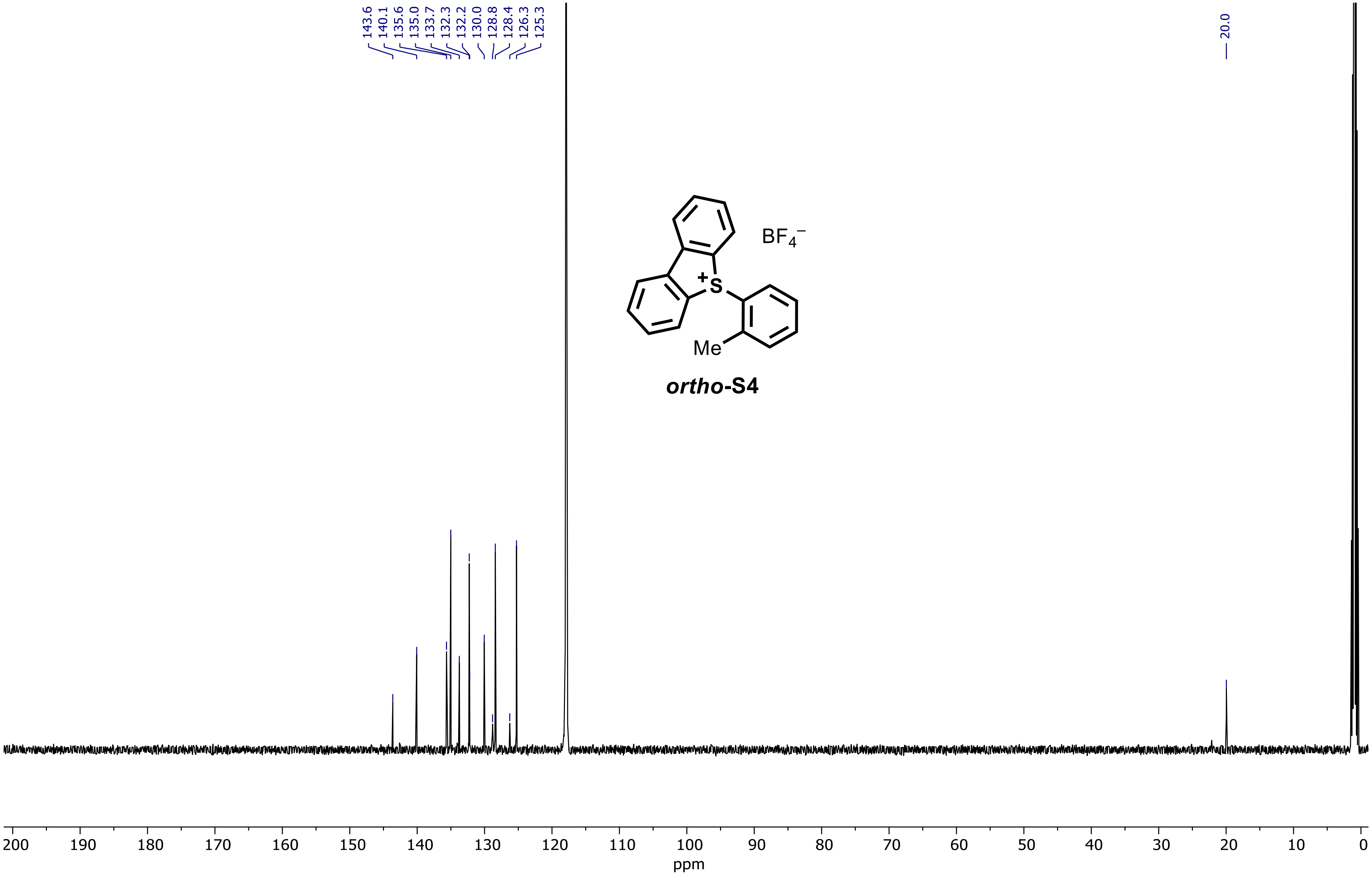

## $^1$H NMR of toluene derived diphenylsulfonium salt S5, *para*-isomer

$CD_3CN$, 23 ºC

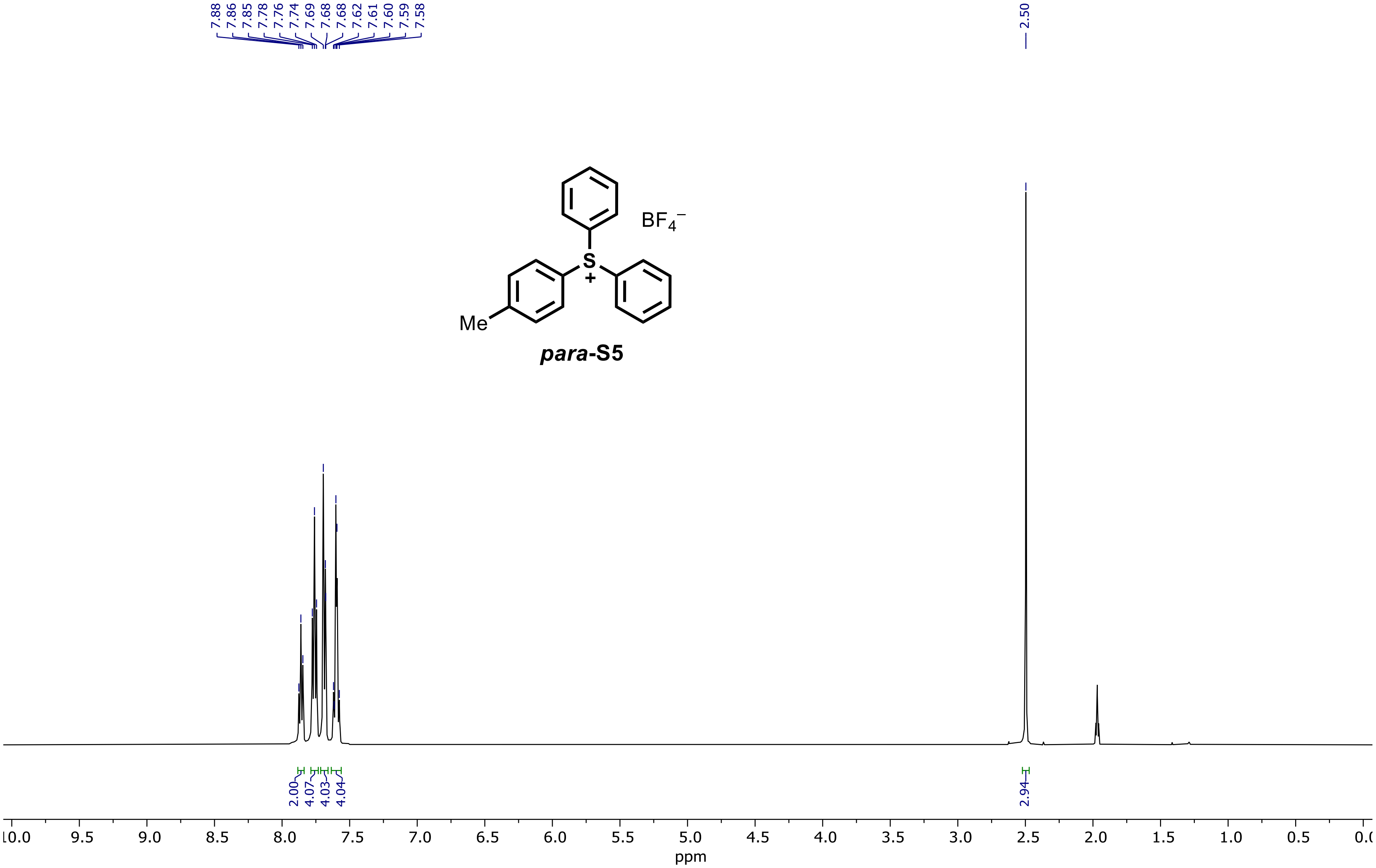

### $^{13}$C NMR of toluene derived diphenylsulfonium salt S5, *para*-isomer

$CD_3CN$, 23 ºC

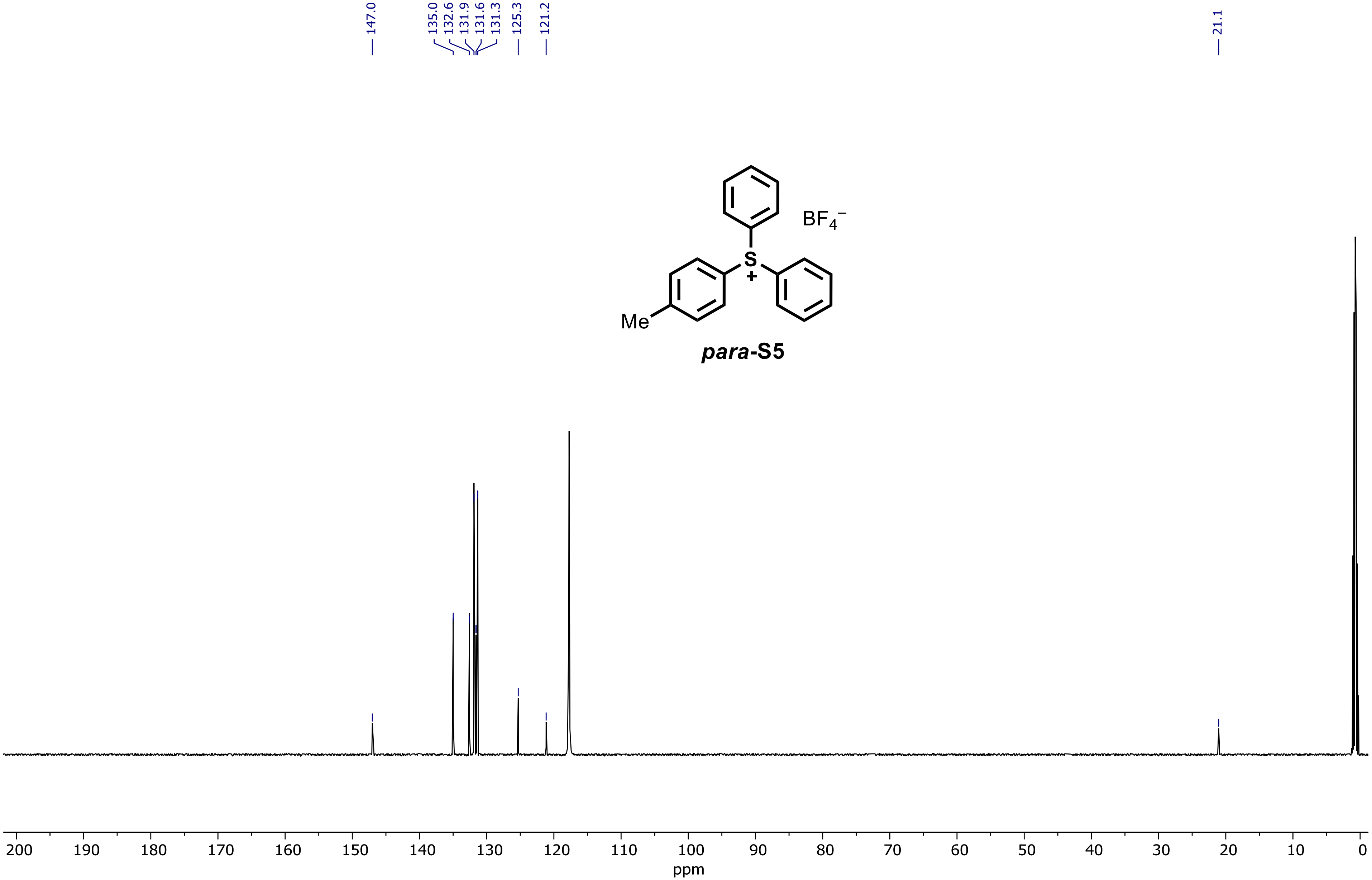


***para*-S5**

### [1]H NMR of toluene derived diphenylsulfonium salt S5, *meta*-isomer

$CD_3CN$, 23 ºC

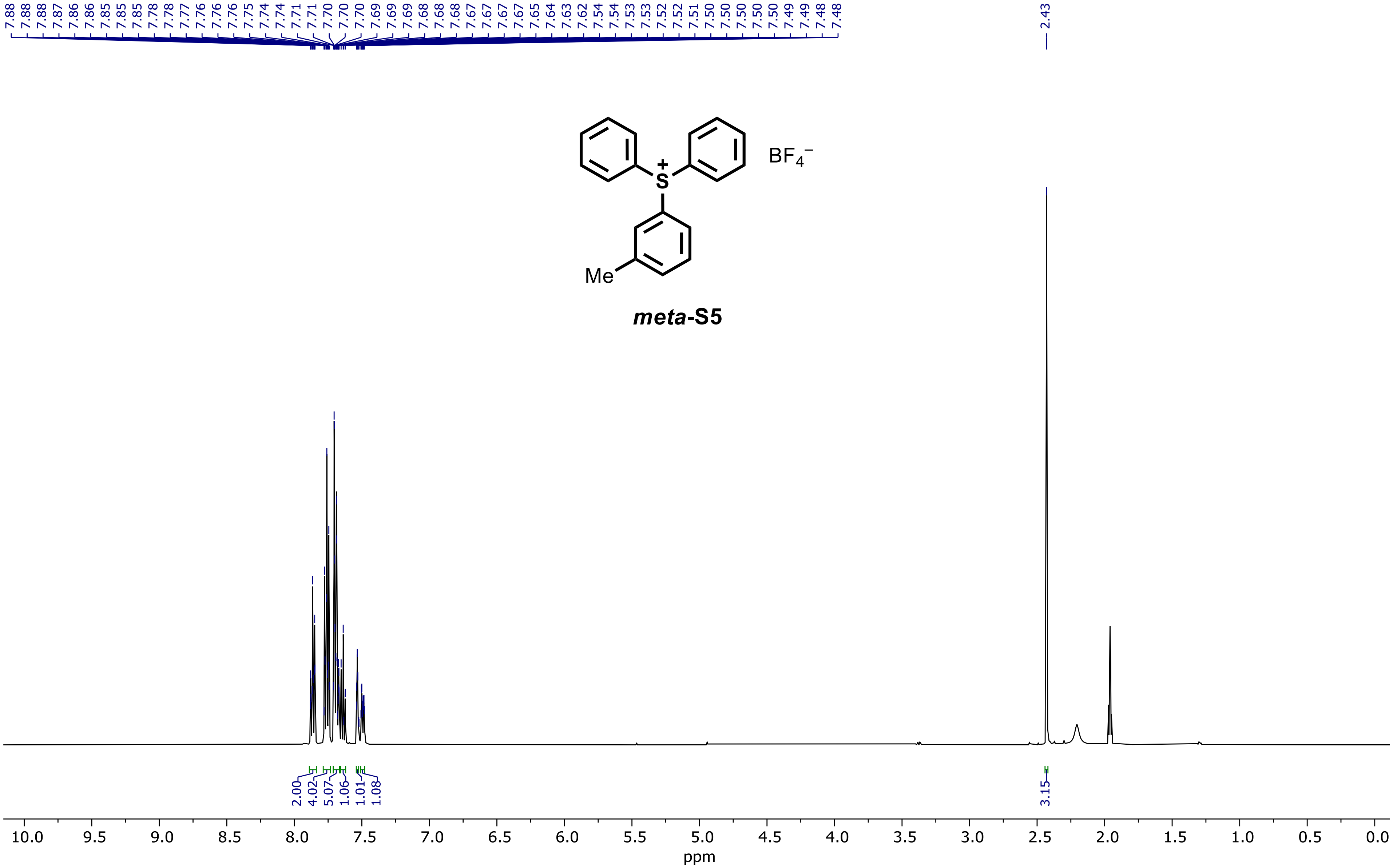

**$^{13}$C NMR of toluene derived diphenylsulfonium salt S5, *meta*-isomer**

CD₃CN, 23 ºC

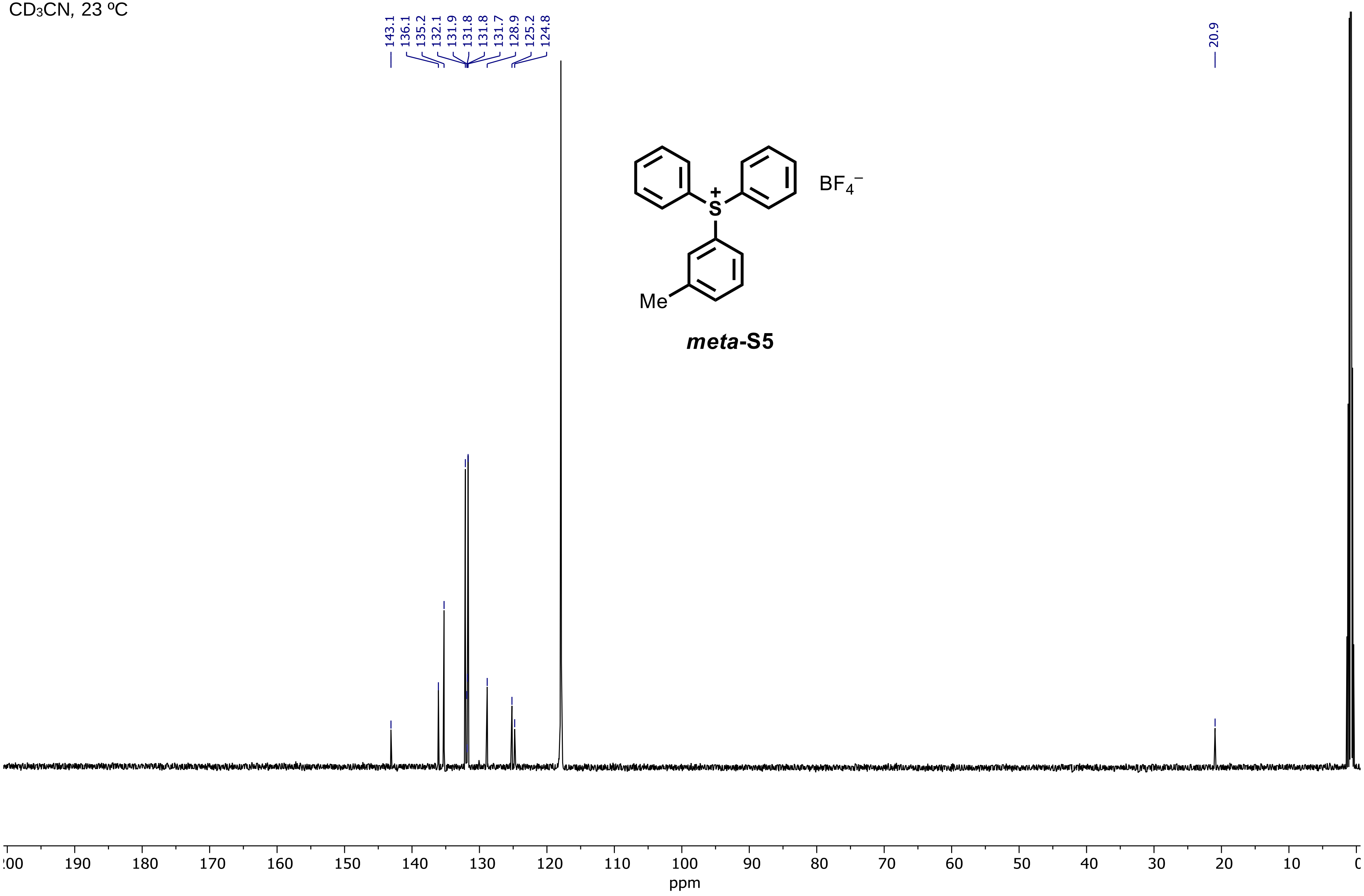

## $^1$H NMR of toluene derived diphenylsulfonium salt S5, *ortho*-isomer

$CD_3CN$, 23 ºC

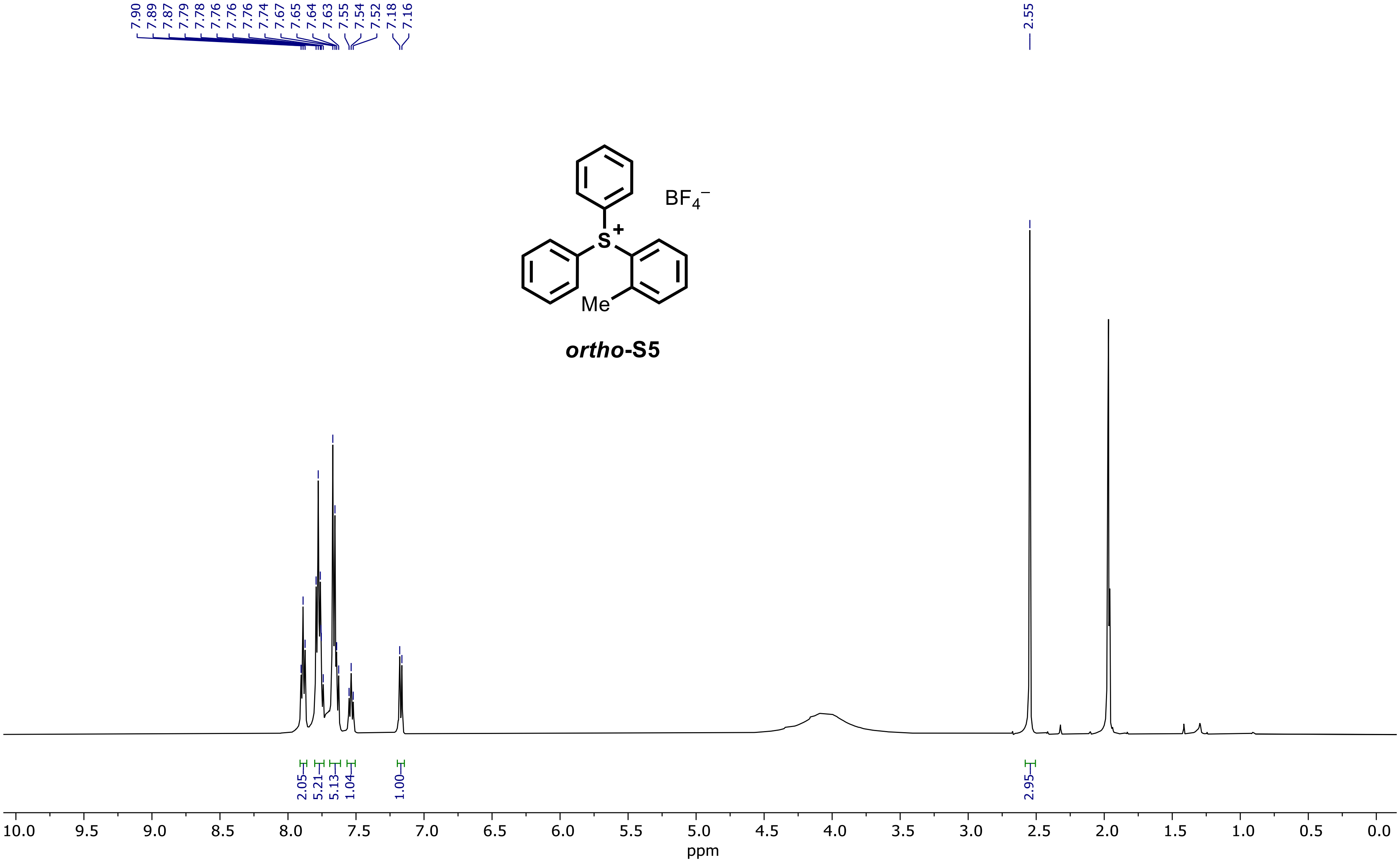

**$^{13}$C NMR of toluene derived diphenylsulfonium salt S5, *ortho*-isomer**

$CD_3CN$, 23 ºC

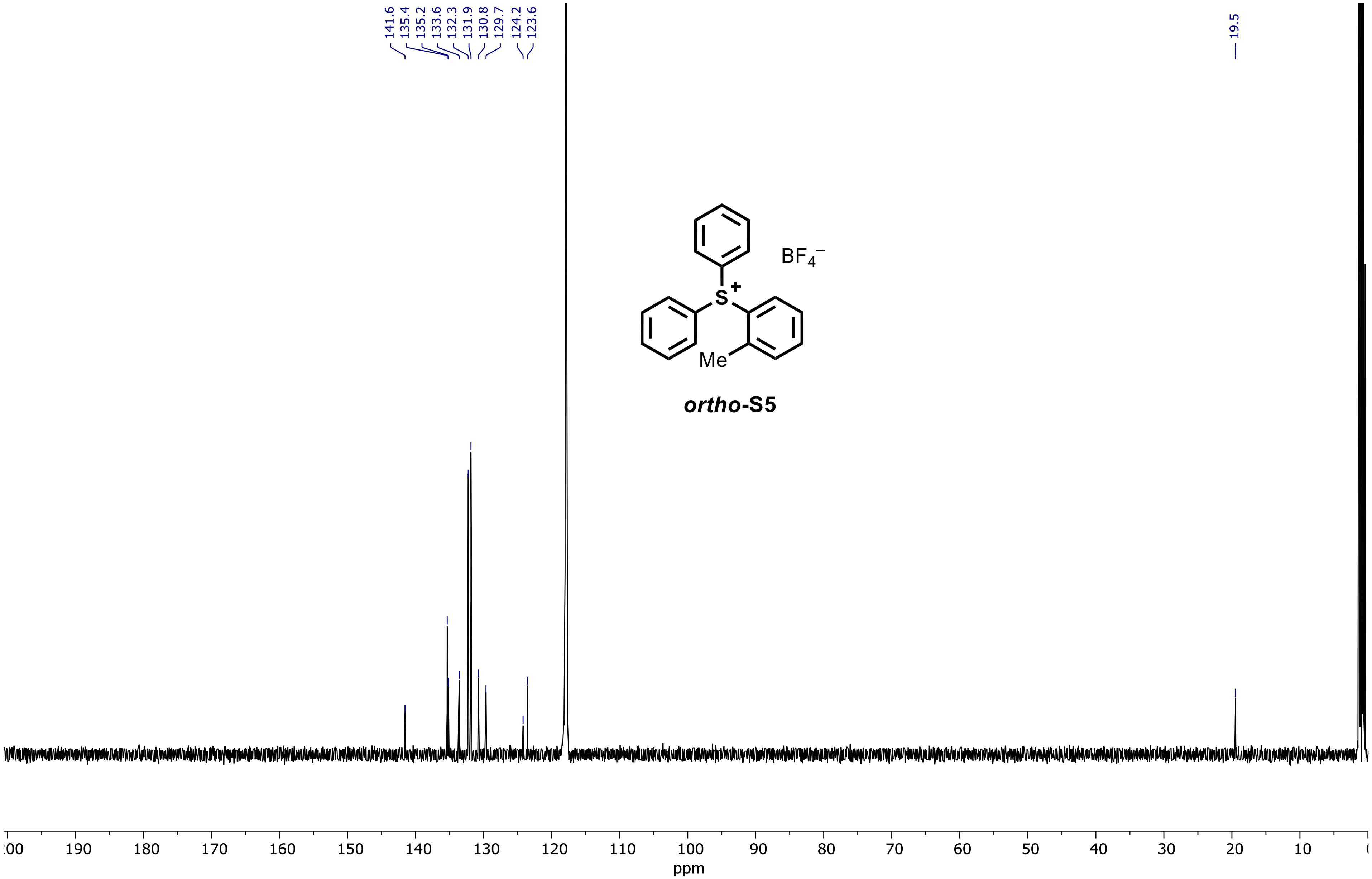

**$^1$H NMR of TTO-$d_2$**

$CDCl_3$, 23 ºC

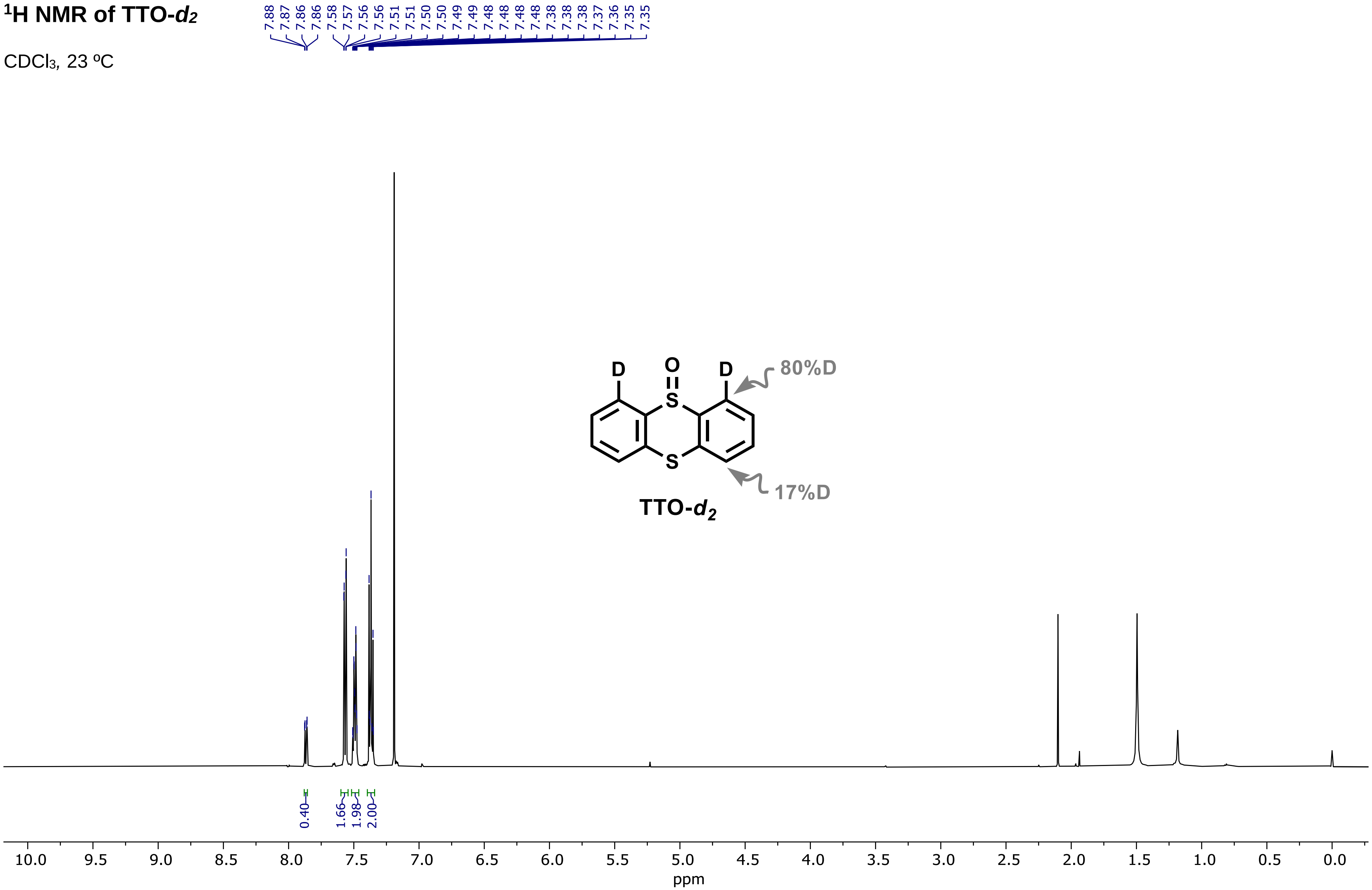

### $^{13}$C NMR of TTO-$d_2$

CDCl$_3$, 23 ºC

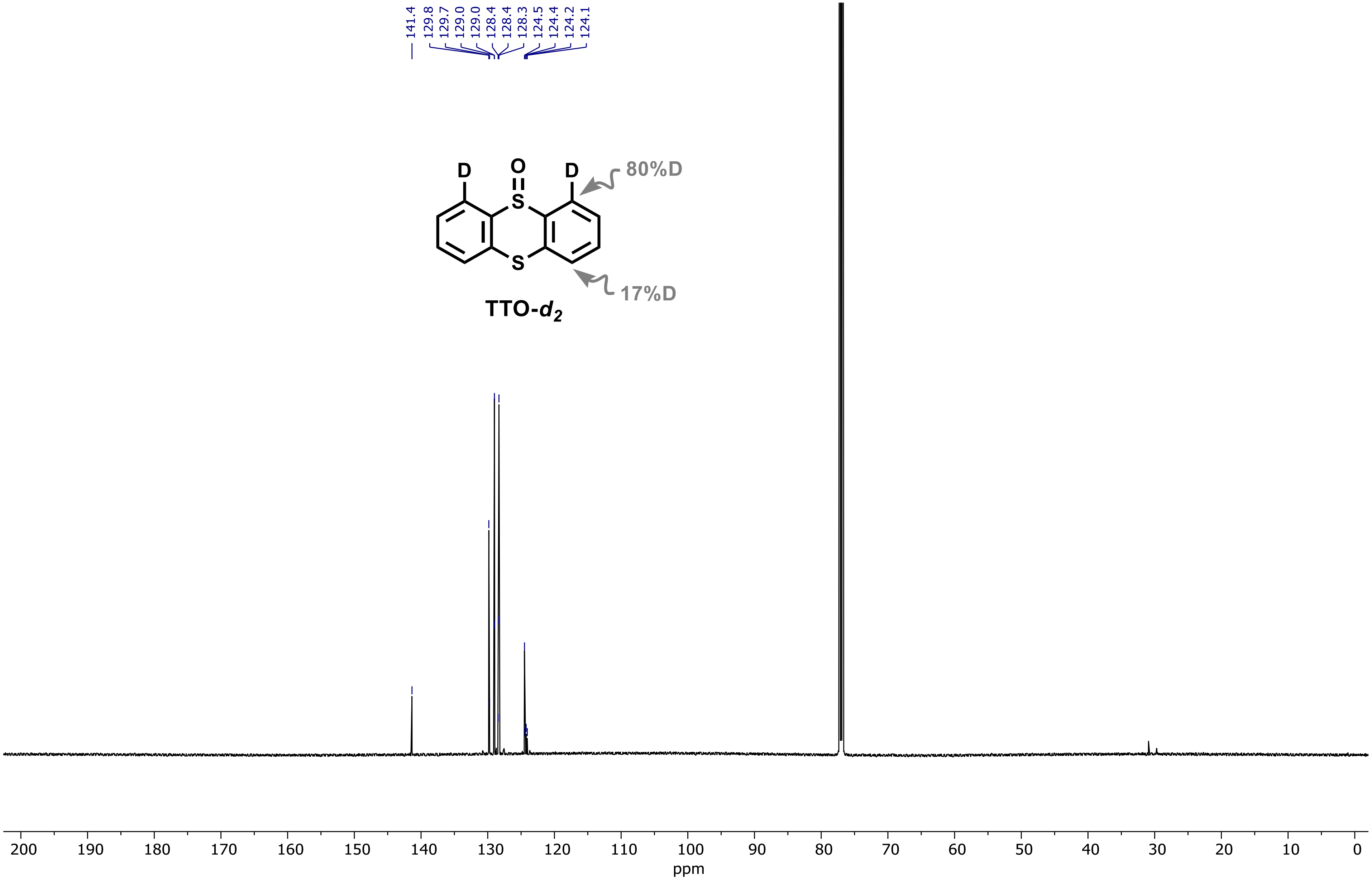

## $^2$H NMR of TTO-$d_2$

$CDCl_3$, 23 ºC

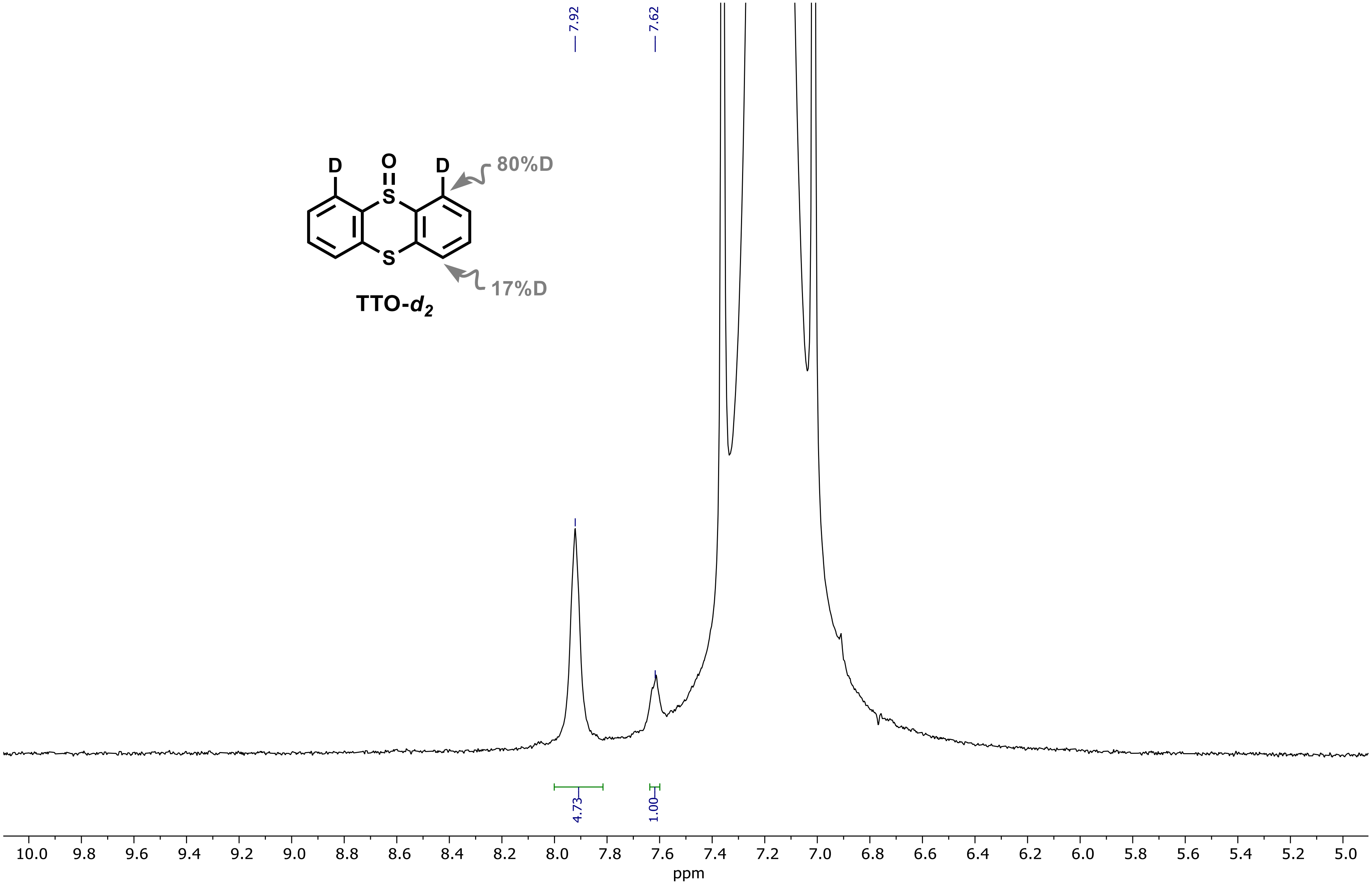

## [1]H NMR of *tert*-butylbenzene derived *d*$_2$-thianthrenium salt 2-*d*$_2$

CDCl$_3$, 23 ºC

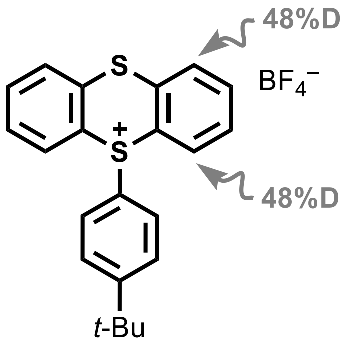


**2-*d*$_2$**

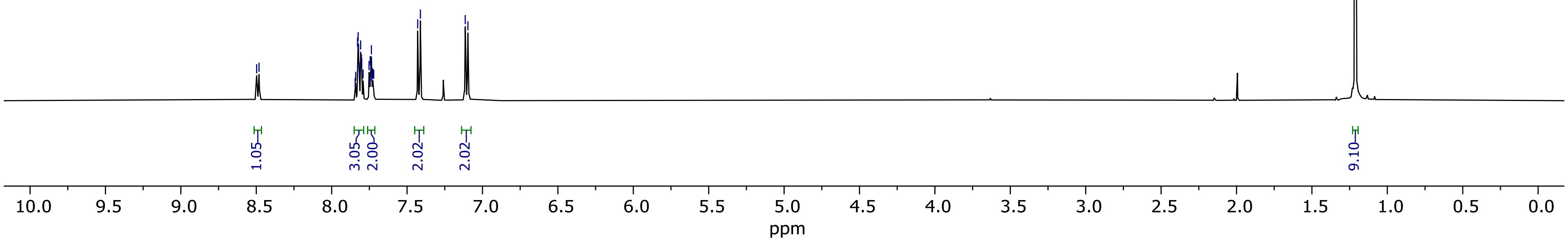

### $^{13}$C NMR of *tert*-butylbenzene derived *d*$_2$-thianthrenium salt 2-*d*$_2$

CDCl$_3$, 23 ºC

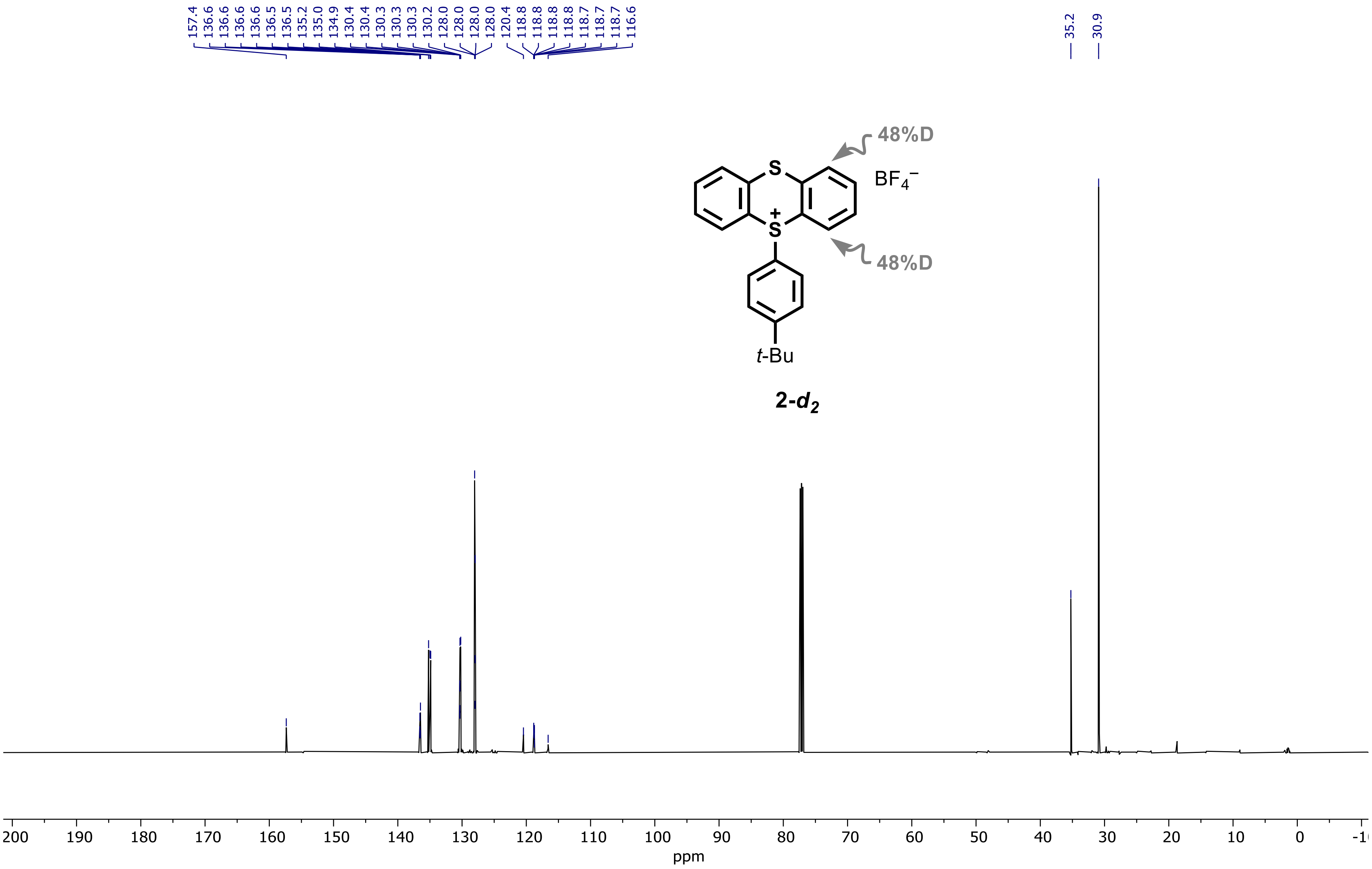

### $^2$H NMR of *tert*-butylbenzene derived $d_2$-thianthrenium salt 2-$d_2$

CDCl$_3$, 23 ºC

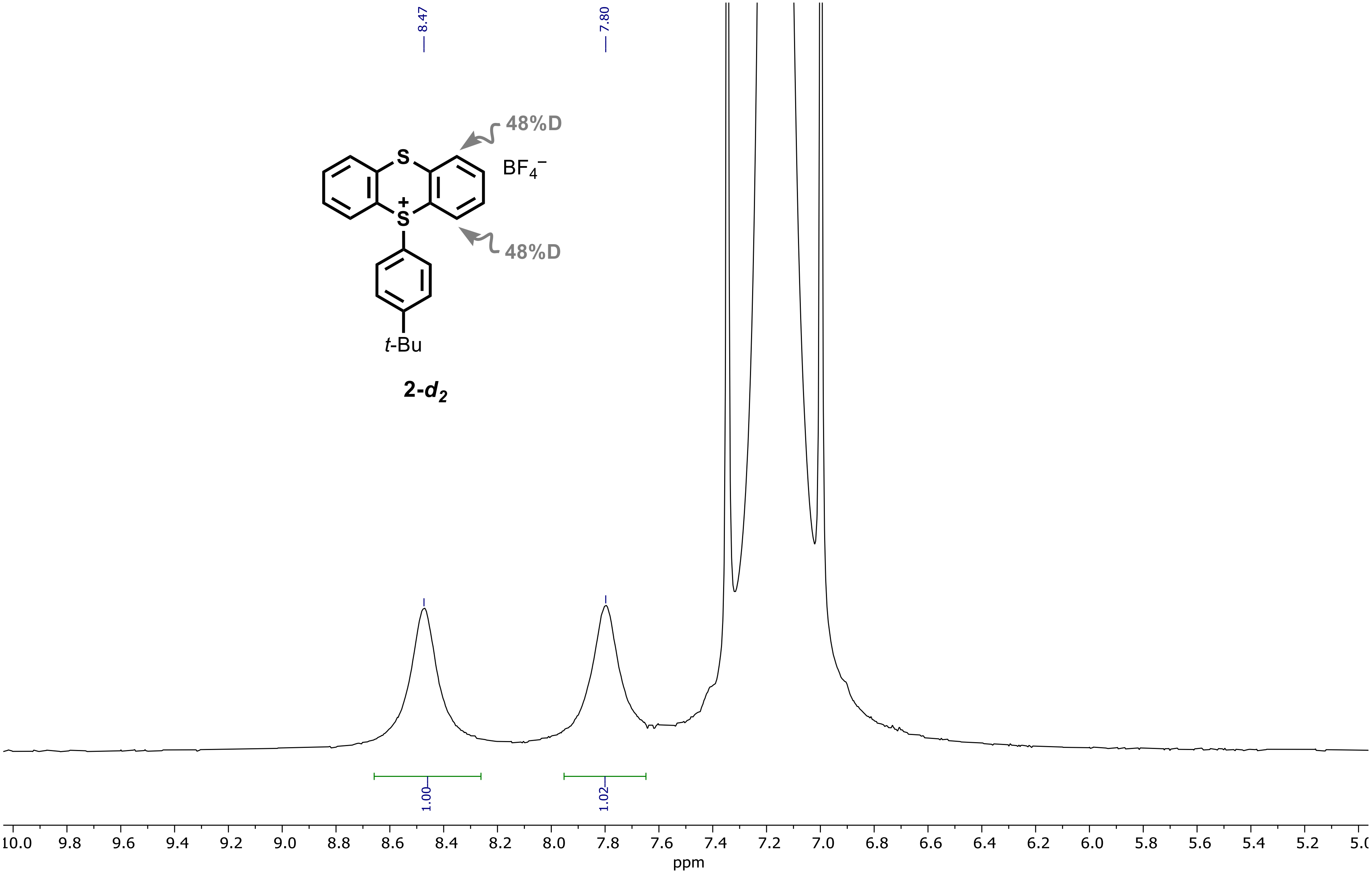


**2-$d_2$**

## References


[1] F. Berger, M. B. Plutschack, J. Riegger, W. Yu, S. Speicher, M. Ho, N. Frank, T. Ritter, *Nature* **2019**, *567*, 223.

[2] H. J. Shine, D. R. Thompson, *Tetrahedron Lett.* **1966**, *7*, 1591-1597.

[3] S. Imazeki, M. Sumino, K. Fukasawa, M. Ishihara, T. Akiyama, *Synthesis* **2004**, 10, 1648-1654.

[4] L. C. Stock, H. C. Brown, *A Quantitative Treatment of Directive Ef-fects in Aromatic Substitution*. In Advances in Physical Organic Chemistry, Gold, V., Ed. Academic Press: **1963**; Vol. 1, 35-154.

[5] F. A. Carey, R. J. Sundberg, *Aromatic Substitution*. In Advanced Organic Chemistry: Part A: Structure and Mechanisms, Springer US: Boston, MA, **2000**; 551-60.

[6] L. L. Miller, B. F. Watkins, *J. Am. Chem. Soc.* **1976**, *98*, 1515-1519.

[7] G. A. Olah, S. J. Kuhn, S. H. Flodd, S. H.; Hardie, B. A., *J. Am. Chem. Soc.* **1964**, *86*, 2203–2209.

[8] W. Kitching, M. Glenn, *Science of Synthesis* **2004**, 3: Category 1, Organometallics. Product Class 3: Organometallic Complexes of Mercury.

[9] H. G. Roth, N. A. Romero, D. A. Nicewicz, *Synlett* **2016**, *27*, 714.

[10] R. S. Glass, *Sulfur Radical Cations*. In Organosulfur Chemistry II, Ed. Springer Berlin Heidelberg: Berlin, Heidelberg, **1999**, 1-87.

[11] L. Eberson, M. P. Hartshorn, O. Persson, F. Radner, *Acta Chem. Scand.* **1997**, *51*, 592-500.

[12] M. J. Frisch *et al.* Gaussian 16 Revision A.03. **2016**.

[13] J. D. Chai, M. Head-Gordon, *Phys. Chem. Chem. Phys.*, **2008**, *10*, 6615–6620.

[14] A. V. Marenich, C. J. Cramer, D. G. Truhlar, *J. Phys. Chem. B*, **2009**, *113*, 6378–6396.

[15] G. Luchini, J. V. Alegre-Requena, Y. Guan, I. Funes-Ardoiz, R. S. Paton, GoodVibes: GoodVibes 3.0.1, **2019**, http://doi.org/10.5281/zenodo.595246.

[16] S. Grimme, *Chem. Eur. J.*, **2012**, *18*, 9955-9964.

[17] Y. Li, J. Gomes, S. M. Sharada, A. T. Bell, M. Head-Gordon, *Phys. Chem. C*, **2015**, *119*, 1840-1850.
